# ﻿Taxonomic revision of *Muhlenbergia* (Poaceae, Chloridoideae, Cynodonteae, Muhlenbergiinae) in Central America: phylogeny and classification

**DOI:** 10.3897/phytokeys.230.103882

**Published:** 2023-08-03

**Authors:** Paul M. Peterson, Yolanda Herrera Arrieta, Silvia Lobo Cabezas, Konstantin Romaschenko

**Affiliations:** 1 Department of Botany MRC-166, National Museum of Natural History, Smithsonian Institution, Washington, DC 20013-7012, USA National Museum of Natural History Washington, DC United States of America; 2 Instituto Politécnico Nacional, CIIDIR Unidad‐Durango‐COFAA, Durango, C.P. 34220, México Instituto Politécnico Nacional Durango Mexico; 3 Herbario Nacional, Museo Nacional de Costa Rica, Apartado Postal 749-1000, San José, Costa Rica Museo Nacional de Costa Rica San José Costa Rica

**Keywords:** Central America, classification, ITS, lectotypification, *Muhlenbergia*, phylogeny, plastid DNA sequences, Poaceae, systematics, taxonomy

## Abstract

A taxonomic treatment of 38 species of *Muhlenbergia*, a phylogeny based on analysis of six DNA sequence markers, and classification of *Muhlenbergia* for Central America (Belize, Costa Rica, El Salvador, Guatemala, Honduras, Nicaragua, Panama; and Campeche, Chiapas, Quintana Roo, Tabasco, and Yucatán, México) is given. With the support from a molecular phylogeny we describe Muhlenbergiasubg.Ramulosae**subgen. nov.** In our treatment we place *M.gigantea* (younger name) as a synonym of *M.mutica.* Lectotypes are designated for the names *Agrostismicrosperma* Lag., *Epicampesgigantea* E. Fourn., *Lamarckiatenella* DC., *Muhlenbergiaadspersa* Trin., *M.diversiglumis* Trin., *M.exilis* E. Fourn., *M.flabellata* Mez, *M.setarioides* E. Fourn., *Pereilemaciliatum* E. Fourn., P.crinitumvar.cirratum E. Fourn., *Podosemumciliatum* Kunth, *P.tenuissimum* J. Presl, and *Schellingiatenera* Steud.

## Abstract (it)

Brindamos un tratamiento taxonómico para 38 especies de *Muhlenbergia*, una filogenia basada en el análisis de seis marcadores de secuencia de ADN y la clasificación de *Muhlenbergia* para América Central (Belize, Costa Rica, El Salvador, Guatemala, Honduras, Nicaragua, Panama; y Campeche, Chiapas, Quintana Roo, Tabasco y Yucatán, México). Con el apoyo de una filogenia molecular describimos Muhlenbergiasubg.Ramulosae**subgen. nov.** En nuestro tratamiento colocamos a *M.gigantea* (nombre más joven) como sinónimo de *M.mutica*. Se designan lectotipos para los nombres *Agrostismicrosperma* Lag., *Epicampesgigantea* E. Fourn., *Lamarckiatenella* DC., *Muhlenbergiaadspersa Trin.*, *M.diversiglumis* Trin., *M.exilis* E. Fourn., *M.flabellata* Mez, *M.setarioides* E. Fourn., *Pereilemaciliatum* E. Fourn., P.crinitumvar.cirratum E. Fourn., *Podosemumciliatum* Kunth, *P.tenuissimum* J. Presl y *Schellingiatenera* Steud.

## ﻿Introduction

With the incorporation of molecular DNA studies, the classification of the grass family has improved, and we now recognize 12 subfamilies, seven supertribes, 54 tribes, five super subtribes, and 109 subtribes (Soreng et al. 2022). Many satellite genera have been subsumed within larger genera and new genera have been described to recognize monophyletic clades. Sequence-derived phylogenies are extremely useful for elucidating synapomorphies, and these are used to circumscribe a clade or lineage of closely related species.

The subtribe Muhlenbergiinae Pilg. (Cynodonteae Dumort.) is a diverse assemblage of 183 species represented by a single, monophyletic genus, *Muhlenbergia* Schreb. (Peterson et al. 2010a, b, 2016, 2018, 2021; Soreng et al. 2017, 2015, 2022). Species within *Muhlenbergia* are morphologically highly variable and are characterized in having membranous ligules (rarely a line of hairs); paniculate inflorescences that are rebranched or composed only of primary branches; spikelets that are usually solitary but sometimes in pairs or triads, with cleistogenes (self-pollinated flowers that do not open at maturity) occasionally present in the leaf sheaths; one floret (rarely more) per spikelet that is perfect, staminate, or sterile; glumes that are awned or unawned; lemmas 3-veined, awned or unawned; and a base chromosome number of *x* = 8–10 (Peterson et al. 1995, 1997, 2007a, b; Peterson 2000, 2003). Two subtypes of C_4_ photosynthesis based on nicotinamide adenine dinucleotide cofactor malic enzyme (NAD-ME) and phosphoenolpyruvate carboxykinase (PCK) have been identified anatomically in *Muhlenbergia*, with a few species verified by biochemical assay (Gutierrez et al. 1974; Brown 1977; Hattersley and Watson 1992).

Based on analysis of seven molecular markers (nuclear ITS and plastid *ndhA* intron, *ndhF*, *rps16-trnK*, *rps16* intron, *rps3*, and *rpl32-trnL* DNA sequences), Peterson et al. (2010b) provided a phylogeny and classification for 124 species (68%) of the Muhlenbergiinae. They recognized five subgenera within *Muhlenbergia: M.* subg. Bealia (Scribn.) P.M. Peterson, M.subg.Clomena (P. Beauv.) Hack., M.subg.Muhlenbergia, M.subg.Pseudosporobolus (Parodi) P.M. Peterson, and M.subg.Trichochloa (P. Beauv.) A. Gray. Formerly, subtribe Muhlenbergiinae included 10 genera but based on DNA-derived phylogenies nine of these genera were subsumed within *Muhlenbergia* (Giraldo-Cañas and Peterson 2009; Columbus and Smith 2010; Peterson et al. 2010b). The phylogeny of *Muhlenbergia* was revisited in Peterson et al. (2018, 2021) and was based on 150 of the 183 (82%) species in the genus. To show the affinities of the taxa treated in this revision we include a phylogenetic tree generated previously in Peterson et al. (2021).

Biogeographical reconstruction of *Muhlenbergia* suggests the genus originated 9.3 mya in the Sierra Madre (Occidental and Oriental) in México, splitting into six lineages, with *M.ramulosa* (Kunth) Swallen diverging 8.2 mya, M.subg.Muhlenbergia at 5.9 mya, M.subg.Pseudosporobolus at 5.9 mya, M.subg.Clomena at 5.4 mya, M.subg.Bealia at 4.3 mya, and M.subg.Trichochloa at 1 mya, each of these with a high probability of Sierra Madrean origin (Peterson et al. 2021). Founder‐event speciation from Sierra Madre to Central America occurred independently multiple times in four of the five subgenera during the Pleistocene and late Pliocene (Peterson et al. 2021).

The most comprehensive treatment of *Muhlenbergia* for Central America appears in Flora Mesoamericana where Reeder (1994) recognized 36 species. In addition, *Aegopogon* Humb. & Bonpl. ex Willd. with two species (Pohl 1994a), *Lycurus* Kunth with a single species (Davidse 1994), and *Pereilema* J. Presl with three species (Pohl 1994b) appear in Flora Mesoamericana, all now included within *Muhlenbergia* (Peterson et al. 2010a, b). All of these genera, as then understood, were treated in the subtribe Sporobolinae Benth. (Davidse and Pohl 1994).

Here we present a phylogeny, classification, and a taxonomic revision of 38 species of *Muhlenbergia* for Central America Central (Belize, Costa Rica, El Salvador, Guatemala, Honduras, Nicaragua, Panama; and Campeche, Chiapas, Quintana Roo, Tabasco, and Yucatán, México). Since the Flora Mesoamericana (Davidse and Pohl 1994) included the Yucatán Peninsula and Chiapas in addition to Central America (political region), we also include in our treatment Campeche, Chiapas, Quintana Roo, Tabasco, and Yucatán, a region that harbors species of *Muhlenbergia*.

## ﻿Materials and methods

### ﻿Phylogenetic analyses

**Figure 1.**
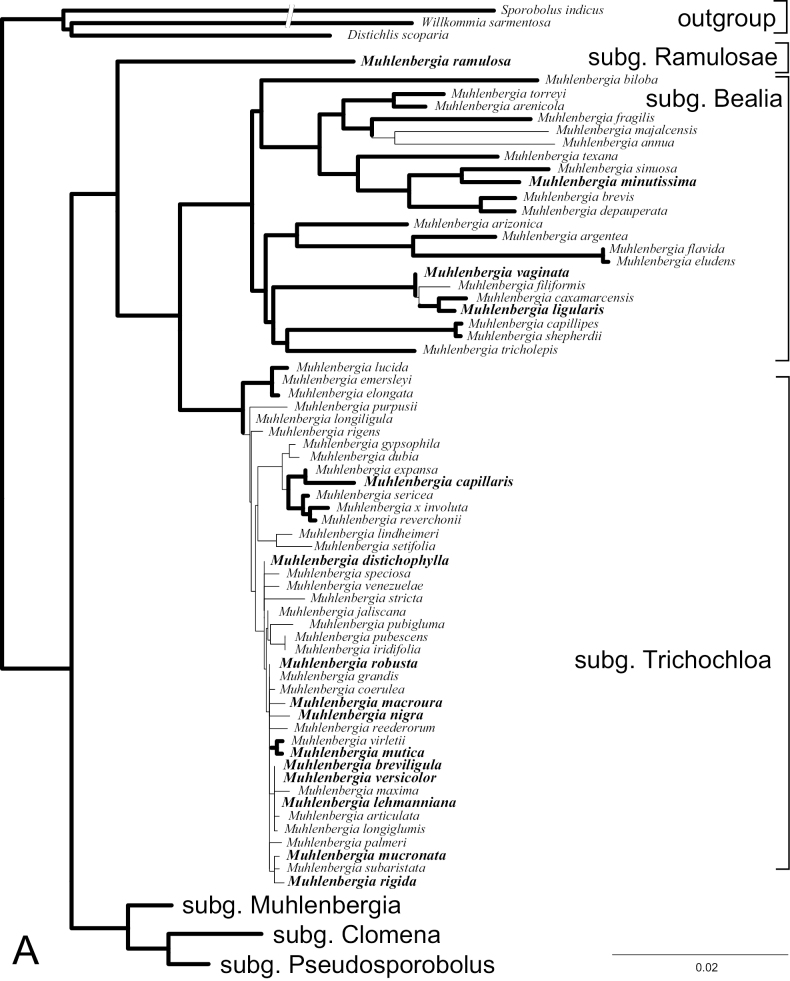
**A, B** Bayesian tree inferred from combined plastid (*ndhA* intron, *rps16-trnK*, *rps16* intron, *rps3*, and *rpl32-trnL*) and ITS sequences. Thick branches indicate posterior probabilities of 0.95–1; species in bold occur in Central America; Scale bar: 2%.

The phylogram (Fig. 1) was generated using existing data from Peterson et al. (2021). The methods for DNA extraction, primers, amplification, sequencing, and phylogenetic analysis are given in Peterson et al. (2010b, 2016, 2018, 2021). We estimated the phylogeny among members of *Muhlenbergia* based on the analysis of six DNA sequence markers (ITS 1&2 and plastid *ndhA* intron, *rpl32-trnL*, *rps3*, *rps16* intron, and *rps16-trnK*).We sampled 150 species of *Muhlenbergia* (82%) within subtribe Muhlenbergiinae, and included outgroups: *Distichlisscoparia* (Nees ex Kunth) Arechav. (Monanthochloinae Pilg. ex Potztal), *Willkommiasarmentosa* Hack. (Traginae P.M. Peterson & Columbus), and *Sporobolusindicus* L. (Zoysieae Benth., Sporobolinae Benth.). Voucher information with GenBank numbers and characteristics of the six regions along with parameters used in Bayesian analyses can be found in Peterson et al. (2021) or are available upon request.

### ﻿Taxonomy

Specimens of *Muhlenbergia* from Central America were reviewed in the following herbaria: CAS, CIIDIR, CR, DS, ENCB, INB (now part of CR), ITC, LAGU, MEXU, MHES, MICH, MO, NY, SLPM, TAES, TEFH, US, and USJ (Thiers 2023). Distribution information for each species is based on a review of the literature and on specimens cited in this treatment. Additional synonyms accepted by us can be found in the Catalogue of New World Grasses web site (http://www.tropicos.org/Project/CNWG) that is continually updated within TROPICOS (http://www.tropicos.org).

When counting culm nodes it is best to start counting 1 cm above the base. Blade width is measured from margin to margin on a flat blade but not when the blade is tightly involute. Glabrous refers to without pubescence. Smooth indicates no prickle-hairs with broad bases and/or hooked or pointed apices (i.e. pubescence can occur on a smooth surface, and a scabrous surface can be glabrous). Excluded species and an infrageneric classification of the accepted species of *Muhlenbergia* in Central America are presented at the end prior to the references.

## ﻿Results and discussion

### ﻿Phylogeny

The Bayesian tree from the combined analysis of ITS and five plastid regions is well resolved with strong support for the monophyly of *Muhlenbergia*, including *M.ramulosa* (Kunth) Swallen sister to M.subg.Bealia + M.subg.Trichochloa, these all in one clade that is sister to M.subg.Clomena + M.subg.Pseudosporobolus sister to M.subg.Muhlenbergia. (Fig. 1; posterior probability, PP = 0.95–1, shown with thick branches). Each of the six major clades, corresponding to the six subgeneric divisions, five of these clades recognized previously (Peterson et al. 2010b; 2018), include species that occur in Central America (shown in **bold**). The species in each of these subgenera share salient morphological characteristics or trends.

Species of Muhlenbergiasubg.Bealia are strongly caespitose, never rhizomatous, annuals or perennials with pubescent margins or midveins at least on the lower ½ of the lemma (only *M.ligularis* is without pubescence), and round, equal primary, secondary, and tertiary vascular bundles without well-developed sclerenchyma (Peterson and Herrera Arrieta 2001; Peterson 2003; Peterson et al. 2010b). *Muhlenbergiaminutissima* (Steud.) Swallen and *M.vaginata* Swallen (two species included in our revision) are members of this subgenus.

Although the clade of species representing *Muhlenbergiasubg.Trichochloa* is strongly supported in our analyses (Fig. 1), there is little resolution among members, indicative of low levels of genetic divergence in the studied markers among the species in this subgenus. The low level of divergence may be a consequence of rapid speciation events. Within *Muhlenbergia*, species within this group are by far the most difficult to determine because there are few morphological differences among the taxa and discrete (nonplastic) characteristics are few. Species of *M.subg.Trichochloa* consist of robust perennials up to three meters tall with compressed-keeled or rounded basal sheaths, 1-veined glumes, and unequal rectangular or obovate/elliptic secondary and tertiary vascular bundles with well-developed sclerenchyma girders, these usually with sclerosed phloem (Peterson and Herrera Arrieta 2001; Herrera Arrieta and Peterson 2007, 2017, 2018; Peterson et al. 2010b). Thirteen of the species in Central America are placed in *M.* subg. *Trichochloa: M.aurea* Swallen (endemic to Guatemala), *M.breviligula* Hitchc., *M.capillaris* (Lam.) Trin., *M.distichophylla* J. Presl) Kunth, *M.lehmanniana* Henrard, *M.macroura* (Kunth) Hitchc., *M.mucronata* (Kunth) Trin., *M.mutica* (Rupr. ex E. Fourn.) Hitchc., *M.nigra* Hitchc., *M.rigida* (Kunth) Kunth, *M.robusta* (E. Fourn.) Hitchc., *M.versicolor* Swallen, and *M.xanthodas* Soderstr.

Species of Muhlenbergiasubg.Clomena have 3-veined upper glumes that are often 3-toothed, densely caespitose non rhizomatous culms with lower leaf sheaths that often flat and somewhat papery at maturity, and lemmas with flexuous awns [only *M.jonesii* (Vasey) Hitchc., an endemic to California, lacks an awn but the apex is mucronate] (Reeder and Reeder 1995; Herrera Arrieta 1998; Peterson 2003; Peterson et al. 2010b). Of the Central America species, *Muhlenbergiaflabellata* Mez (endemic to Costa Rica and Panama), *M.montana* (Nutt.) Hitchc., *M.peruviana* (P. Beauv.) Steud., and *M.quadridentata* (Kunth) Trin. are members of this subgenus.

Members of Muhlenbergiasubg.Pseudosporobolus usually have plumbeous spikelets, well-developed adaxial and abaxial sclerenchyma in their primary vascular bundles, narrow to loosely open panicles, unawned, mucronate or short-awned lemmas [long-awned in *M.implicata* (Kunth) Trin.], and the plants are rhizomatous when perennial (Peterson and Herrera Arrieta 2001; Peterson 2003; Peterson et al. 2010b). In Central America, *M.implicata*, *M.phalaroides* (Kunth) P.M. Peterson, *M.plumbea* (Trin.) Hitchc., *M.tenuissima* (J. Presl) Kunth, and *M.utilis* (Torr.) Hitchc. are placed in M.subg.Pseudosporobolus.

Morphologically, species of the Muhlenbergiasubg.Muhlenbergia clade have broad, flat leaf blades, most have well-developed, scaly, and creeping rhizomes, and panicles that are usually narrow at maturity (Peterson et al. 2010b). This is the only subgenus where the PCK subtype of C_4_ photosynthesis has been found. PCK species contain chlorenchyma composed of tabular cells that are indistinctly radiate and continuous between bundles [PCK type, defined as centrifugal/evenly distributed photosynthetic carbon reduction (PCRD) cell chloroplasts (with grana). The major veins are surrounded by two bundle sheaths, an inner mestome sheath of elongate nonchlorenchymatous cells and an outer chlorenchymatous sheath of shorter PCRD cells (designated XyMS+structural type; Hattersley and Watson 1976, 1992; Dengler et al. 1986). In addition, the leaf blades of these species contain fan- to shield-shaped bulliform cells that do not form a column of colorless cells from the adaxial to the abaxial surface, and they generally have four or more secondary and/or tertiary vascular bundles between consecutive primary vascular bundles (Gutierrez et al. 1974; Brown 1977; Peterson and Herrera Arrieta 2001). In Central America, *M.cenchroides* (Humb. & Bonpl. ex Willd.) P.M. Peterson, *M.ciliata* (Kunth) Trin., *M.diandra* (R.W. Pohl) Columbus (endemic to Costa Rica), *M.diversiglumis* Trin., *M.microsperma* (DC.) Kunth, *M.pereilema* P.M. Peterson, *M.plumiseta* Columbus, *M.setarioides* E. Fourn., *M.spiciformis* Trin., *M.tenella* (Kunth) Trin., *and M.uniseta* (Lag.) Columbus are placed in M.subg.Muhlenbergia.

In our study, and all previous molecular studies, *M.ramulosa* does not align within an existing subgenus of *Muhlenbergia.* Instead, it is sister to M.subg.Bealia and M.subg.Trichochloa in plastid and combined phylograms and sister to all Muhlenbergiinae in ITS phylograms (Columbus et al. 2010; Peterson et al. 2010b, 2018, 2021). Morphologically, *M.ramulosa* is similar to many of the small delicate annual or perennial species classified in M.subg.Bealia. *Muhlenbergiaramulosa* differs from most species of *M.* subg, *Bealia* in having awnless, mottled (greenish-black and greenish-white areas) lemmas, obtuse to subacute and glabrous glumes that are shorter than the lemma, panicles that are exserted and loosely contracted, ovoid or deltoid in outline 0.6–2.7 cm wide, and individuals with erect or spreading culms that do not root at the lower nodes (Peterson and Annable 1991; Herrera Arrieta and Peterson 2018). We describe a new subgenus below to include this enigmatic species.

### ﻿Taxonomic treatment

#### ﻿ Muhlenbergia

Taxon classificationPlantaePoalesPoaceae

Schreb., Gen. Pl. 1: 44. 1789.

898BC280-1645-5C86-A461-FF6EA26A755C

Dilepyrum Michx., Fl. Bor.-Amer. 1: 40. 1803. Type: Dilepyrumminutiflorum Michx. (= Muhlenbergiaschreberi J. F. Gmel.). (M.subg.Muhlenbergia).
Aegopogon Humb. & Bonpl. ex Willd., Sp. Pl. 4 (2): 899. 1805 [1806]. Type: Aegopogoncenchroides Humb. & Bonpl. ex Willd. [≡ Muhlenbergiacenchroides (Humb. & Bonpl. ex Willd.) P.M. Peterson]. (M.subg.Muhlenbergia).
Podosemum Desv., Nouv. Bull. Sci. Soc. Philom. Paris 2: 188. 1810. Type: Podosemumcapillare (Lam.) Desv. [≡ Muhlenbergiacapillaris (Lam.) Trin.]. (M.subg.Trichochloa).
Clomena P. Beauv., Ess. Agrostogr. 28. 1812. Type: Clomenaperuviana P. Beauv. [≡ Muhlenbergiaperuviana (P. Beauv.) Steud.]. (M.subg.Clomena).
Tosagris P. Beauv., Ess. Agrostogr. 29. 1812. Type: Tosagrisagrostidea P. Beauv. [= Muhlenbergiacapillaris (Lam.) Trin.]. (M.subg.Trichochloa).
Trichochloa P. Beauv., Ess. Agrostogr. 29. 1812. Type: Trichochloapurpurea P. Beauv. [= Muhlenbergiaexpansa (Poir.) Trin.]. (M.subg.Trichochloa).
Podosaemum Kunth, Mem. Mus. Hist. Nat. 2: 72. 1815; orth. var. Podosemum. (M.subg.Trichochloa).
Hymenothecium Lag., Gen. Sp. Pl. 4. 1816. Lectotype: Cynosurustenellus Cav. ex DC., designated by Hitchcock 1920: 169 [≡ Lamarckiatenella DC. ≡ Hymenotheciumtenellum (Cav. ex DC.) Lag. = Muhlenbergiauniseta (Lag.) Columbus]. (M.subg.Muhlenbergia).
Lycurus Kunth, Nov. Gen. Sp. 1: 141. 1815 [1816]. Lectotype: Lycurusphleoides Kunth, designated by Hitchcock 1920: 139 [≡ Muhlenbergiaphleoides (Kunth) Columbus]. (M.subg.Pseudosporobolus).
Anthipsimus Raf., J. Phys. Chim. Hist. Nat. Arts 89: 105. 1819. Type: Anthipsimusgonopodus Raf. (= Muhlenbergiaschreberi J.F. Gmel.). (M.subg.Muhlenbergia).
Sericrostis Raf., Neogenyton 4. 1825. Lectotype: Stipasericea Michx, designated by Pfeiffer 1874: 1142 [≡ Muhlenbergiasericea (Michx.) P.M. Peterson]. (M.subg.Trichochloa).
Pereilema J. Presl, Reliq. Haenk. 1 (4–5): 233.1830. Type: Pereilemacrinitum J. Presl (≡ Muhlenbergiapereilema P.M. Peterson). (M.subg.Muhlenbergia).
Epicampes J. Presl, Reliq. Haenk. 1 (4–5): 235. 1830. Type: Epicampesstricta J. Presl [= Muhlenbergiarobusta (E. Fourn.) Hitchc.]. (M.subg.Trichochloa).
Dactylogramma Link, Hort. Berol. 2: 248. 1833. Type: Dactylogrammacinnoides Link [= Muhlenbergiaglomerata (Willd.) Trin.]. (M.subg.Muhlenbergia).
Calycodon Nutt., Proc. Acad. Nat. Sci. Philadelphia 4: 23. 1848. Type: Calycodonmontanum Nutt. [≡ Muhlenbergiamontana (Nutt.) Hitchc.]. (M.subg.Clomena).
Pleopogon Nutt., Proc. Acad. Nat. Sci. Philadelphia 4: 25. 1848. Type: Pleopogonsetosum Nutt. [≡ Lycurussetosus (Nutt.) C.G. Reeder = Muhlenbergiaalopecuroides (Griseb.) P.M. Peterson & Columbus]. (M.subg.Pseudosporobolus).
Schedonnardus Steud., Syn. Pl. Glumac. 1: 146. 1854. Type: Schedonnardustexanus Steud. [= Lepturuspaniculatus Nutt. ≡ Rottboelliapaniculata (Nutt.) Spreng. ≡ Schedonnarduspaniculatus (Nutt.) Branner & Coville ≡ Spirochloepaniculata (Nutt.) Lunell ≡ Muhlenbergiapaniculata (Nutt.) Columbus]. (M.subg.Pseudosporobolus).
Vaseya Thurb., Proc. Acad. Nat. Sci. Philadelphia 15: 79. 1863. Type: Vaseyacomata Thurb. [= Muhlenbergiaandina (Nutt.) Hitchc.]. (M.subg.Muhlenbergia).
Chaboissaea E. Fourn., Mexic. Pl. 2: 112. 1886. Type: Chaboissaealigulata E. Fourn. [≡ Muhlenbergialigulata (E. Fourn.) Scribn. & Merr.]. (M.subg.Pseudosporobolus).
Crypsinna E. Fourn., Mexic. Pl. 2: 90. 1886. Lectotype: Crypsismacroura Kunth, designated by Hitchcock 1920: 144. [≡ Crypsinnamacroura (Kunth) E. Fourn.≡ Muhlenbergiamacroura (Kunth) Hitchc.]. (M.subg.Trichochloa).
Redfieldia Vasey, Bull. Torrey Bot. Club 14: 133. 1887. Type: Graphephorumflexuosum Thurb. ex A. Gray [≡ Redfieldiaflexuosa (Thurb. ex A. Gray) Vasey ≡ Muhlenbergiamultiflora Columbus]. (M.subg.Pseudosporobolus).
Bealia Scribn., True Grasses 104, f. 45a. 1890. Type: Bealiamexicana Scribn. (≡ Muhlenbergiabiloba Hitchc.). (M.subg.Bealia).
Blepharoneuron Nash, Bull. Torrey Bot. Club 25(2): 88. 1898. Lectotype: Vilfatricholepis Torr., designated by Peterson and Annable 1990: 522 [≡ Blepharoneurontricholepis (Torr.) Nash ≡ Muhlenbergiatricholepis (Torr.) Columbus]. (M.subg.Bealia)
Schaffnerella Nash, N. Amer. Fl. 17(2): 141. 1912. Type: Schaffneragracilis Benth. [≡ Schaffnerellagracilis (Benth.) Nash ≡ Muhlenbergiaspatha Columbus]. (M.subg.Pseudosporobolus).

##### Description.

Plants ***annual*** or ***perennial***; usually synoecious, infrequently andromonoecious; sometimes rhizomatous, often cespitose, sometimes mat-forming, rarely stoloniferous. ***Culms*** 2–300 cm, erect, geniculate, or decumbent, usually herbaceous, sometimes becoming woody. ***Sheaths*** open; ***ligules*** membranous or hyaline (rarely firm or coriaceous), acuminate to truncate, sometimes minutely ciliolate, sometimes with lateral lobes longer than the central portion; ***blades*** narrow, flat, folded, or involute, sometimes arcuate. ***Inflorescences* (synflorescence)** terminal, sometimes also axillary, open to contracted, racemelike or spikelike panicles; **disarticulation** usually above the glumes, occasionally below the pedicels. ***Spikelets*** mostly perfect with 1 (2–6) florets, sometimes staminate or sterile, occasionally paired or in groups of threes then the central spikelet perfect and the lateral ones staminate or sterile; chasmogamous, rarely cleistogamous; ***glumes*** usually (0)1(2–3)-veined, apices entire, erose, or toothed, truncate to acuminate, sometimes mucronate or awned from the midvein, occasionally awned from the lateral veins; ***lower glumes*** sometimes rudimentary or absent, occasionally bifid; ***upper glumes*** shorter than to longer than the florets; ***callus*es** poorly developed, glabrous or hairy, the hairs up to 3.5 mm long, straight; ***lemmas*** glabrous, scabrous, or with short hairs, 3-veined (rarely appearing 5-veined), apices awned from the midvein, mucronate, or unawned; **awns**, if present, straight, flexuous, sinuous, or curled, sometimes borne between 2 minute teeth, lateral veins occasionally extended into awns; ***paleas*** shorter than or equal to the lemmas, 2-veined, apices; ***lodicules*** 2, cuneate, fleshy, sometimes with a narrow membranous apex, apex truncate and slightly lobed, glabrous; ***anthers*** (1–2) 3, purple, orange, yellow, olivaceous, or whitish; ***ovary*** with 2 styles (rarely 1), glabrous, stigmas plumose. ***Caryopses*** an achene,elongate, fusiform or elliptic, slightly dorsally compressed, rarely laterally compressed, free threshing; ***grain*** solid without lipid. ***Cleistogamous panicles*** sometimes present in the axils of the lower cauline leaves, enclosed by a tightly rolled, somewhat indurate sheath. ***Chromosome base number*** is *x* = (8 or 9) 10, and these are relatively small in size when compared to chromosomes in the Pooideae.

##### Distribution.

The genus is primarily distributed in the Western Hemisphere in North, Central, and South America (Peterson 2003; Peterson and Giraldo-Cañas 2012; Peterson et al. 2018, 2021). There are also eight species known to occur in southeastern Asia; six of these are found in China (Wu and Peterson 2006) and five in India (Tiwari et al. 2023).

##### Ecology.

The species occur in open habitats in deserts, grasslands, sclerophyllous scrubland, and margins of forests often in xeric to meso-xeric habitats from near sea level to more than 4000 m.

##### Etymology.

Named for Gotthilf Henry Ernest Muhlenberg (1753–1815), a Lutheran minister and pioneer botanist of Pennsylvania, USA.

### ﻿Key to the species of *Muhlenbergia* in Central America

**Table:** 

1	Spikelets in clusters of three to five or more; lemmas usually with lateral veins excurrent into awns	**2**
–	Spikelets not in clusters of three to five, or if in clusters, then in pairs; lemmas with lateral veins not excurrent into awns	**6**
2	Spikelets subtended by an involucre of bristles; leaf blades with prominent auricles	**3**
–	Spikelets not subtended by an involucre of bristles; leaf blades without auricles	**5**
3	Involucres deciduous with feathery bristles; panicles 0.2–0.6 cm wide	**25. *Muhlenbergiaplumiseta* Columbus**
–	Involucres persistent with scabrous bristles, panicles 1–3 cm wide	**4**
4	Leaf blades 5–9 mm wide; lemma awns straight; stamens 2, anthers 0.7–1 mm long, purple	**6. *M.diandra* (R.W. Pohl) Columbus**
–	Leaf blades 2–3(–5) mm wide; lemma awns flexuous; stamens 3, anthers 0.4–0.7 mm long, yellow	**21. *M.pereilema* P.M. Peterson**
5	Glumes with acute lobes, central vein extending as an awn 2–4 mm long; plants perennial usually caespitose	**4. *M.cenchroides* (Humb. & Bonpl. ex Willd.) P.M. Peterson**
–	Glumes with obtuse or rounded lobes, central vein usually extending as a mucro 0.2–1 mm long; plants annual sprawling or caespitose	**34. *M.uniseta* (Lag.) Columbus**
6	Annual plants	**7**
–	Perennial plants	**17**
7	Glumes and lemmas awnless, the lemma sometimes mucronate with a mucro up to 1.2 mm long	**8**
–	Glumes and/or lemmas awned, the awns (1.5–)5–30 mm long	**11**
8	Glumes sparsely short pilose at least near the apex; pedicels 2–7 mm long, longer than the spikelets; panicles open, ovate with spreading branches, often capillary	**15. *M.minutissima* (Steud.) Swallen**
–	Glumes glabrous; pedicels 1–3 mm long, mostly shorter than the spikelets; panicles contracted, narrow	**9**
9	Panicles contracted, narrow, linear, usually partially included in the uppermost sheath, 0.3–0.7 cm wide; branches ascending or appressed; culms geniculate or decumbent, often rooting at the lower nodes	**36. *M.vaginata* Swallen**
–	Panicles loosely contracted, exerted, 0.5–2.7 cm wide; branches usually open, extended or reflexed to closely appressed; culms erect or decumbent, not rooting in the lower nodes	**10**
10	Lemmas 0.8–1.3 mm long, oval, plump; ligules 0.2–0.5 mm long; anthers 0.2–0.3 mm long	**27. *M.ramulosa* (Kunth) Swallen**
–	Lemmas 1.5–3.0 mm long, lanceolate, slender; ligules 0.6–2.5 mm long; anthers 0.8–1.1 mm long	**12. *M.ligularis* (Hack.) Hitchc.**
11	Upper glumes apex wide and truncate, usually 2 or 3-toothed; lemma awns irregularly flexuous, purple	**22. *M.peruviana* (P. Beauv.) Steud.**
–	Upper glumes apex acuminate, acute or obtuse but never 2 or 3-toothed; lemma awns straight or flexuous, greenish to purplish	**12**
12	Lemmas up to 2.5(–2.7) mm long	**13**
–	Lemmas 2.5–7.6 mm long	**15**
13	Ligules acute or rounded, sometimes lacerate, membranous, 0.6–1.5 mm long; lemmas indistinctly 3-veined with no intermediate veins visible	**33. *M.tenuissima* (J. Presl) Kunth**
–	Ligules truncate, a ciliate membrane, 0.2–0.9 mm long; lemmas prominently 3-veined with the appearance of 5 veins, these intermediate veins are rows of short barbs on top of folded epidermal ridges	**14**
14	Primary panicle branches spreading or reflexed at maturity up to 90° from the culm axis; awns of lemma (1.5–)5–11(–18) mm long; leaf blades alternately inserted along the culm; plants found in moist to dry habitats usually beneath taller vegetation	**5. *M.ciliata* (Kunth) Trin.**
–	Primary panicle branches tightly appressed and ascending; awns of the lemma 12–26 mm long; leaf blades commonly secund or lying to one side of the culm; plants restricted to moist drainages or perennially wet, rocky cliffs	**32. *M.tenella* (Kunth) Trin.**
15	Glumes dimorphic, those from proximal spikelets of each branch, often awnless, those of distal spikelets often one or both awned; ligules up to 0.8 mm long; primary panicle branches appearing secund or lying to one side of the culm axis	**8. *M.diversiglumis Trin.***
–	Glumes similar in all spikelets, awnless; ligules 1.0–2.5(–3) mm long; primary panicle branches alternately inserted along the culm	**16**
16	Panicles diffuse at maturity; pedicels capillary and flexuous, smooth; cleistogamous spikelets absent; lemmas pubescent only on the callus	**10. *M.implicata* (Kunth) Trin.**
–	Panicles not diffuse; pedicels stiff and stout, antrorsely scabrous; cleistogamous spikelets usually present in the axils of the lowermost culm branches; lemmas pubescent along margins, midvein, and callus	**14. *M.microsperma* (DC.) Kunth**
17	Plants rhizomatous, rhizomes slender or stout, scaly and creeping	**18**
–	Plants not rhizomatous	**20**
18	Upper glumes (3–)3.2–4 mm long, apex truncate, obtuse or acute, often with 3 or 4 small teeth less than 1/6^th^ the length; ligules 2–8 mm long, apex acuminate often lacerate; leaf sheaths 10–30 cm long, basal sheaths becoming flattened with age; spikelets 3.4–4.7 mm long, plumbeous; rhizomes short and stout	**26. *M.quadridentata* (Kunth) Trin.**
–	Upper glumes 0.5–1.6(–1.8) mm long, apex acute without any teeth; ligules 0.2–0.8 mm long, apex truncate; leaf sheaths 0.3–5 cm long, basal sheaths not becoming flattened with age; spikelets 1.4–2.4(–3.5) mm long, plumbeous, green or purplish; rhizomes slender and scaly	**19**
19	Panicles open, usually well exerted from upper sheaths, 4–9(–14) cm long, 0.5–4(–8) cm wide; primary branches 2–8 cm long, ascending to spreading up to 50° from the rachis; spikelets 2.5–3.2(–3.5) mm long; paleas 2–2.8(–3.1) mm long, apex acuminate; anthers 1.4–2 mm long	**24. *M.plumbea* (Trin.) Hitchc.**
–	Panicles narrow, contracted, partially included in the upper sheaths, 1–5 cm long, 0.1–0.4 cm wide; primary branches 0.1–1.2 cm long, appressed, rarely spreading 30° from the rachis; spikelets 1.4–2.4 mm long; paleas 1–2 mm long, apex acute; anthers 0.7–1.4 mm long	**35. *M.utilis* (Torr.) Hitchc.**
20	Upper glumes 3-veined; old sheaths flattened below and sometimes spirally twisted near base	**21**
–	Upper glumes 1-veined, rarely 2 or 3-veined; old sheaths not flat and/or spirally twisted near base	**23**
21	Upper glume apices 3 or 4-toothed, the teeth 1/6^th^ the length of the glume; internodes mostly scabrous	**26. *M.quadridentata* (Kunth) Trin.**
–	Upper glume apices usually 3-toothed, occasionally 2-toothed, the teeth about 1/3 the length of the glume; internodes smooth	**22**
22	Sheaths much overlapping or flabellately arranged below; leaf blades mainly basal 2–5 cm long; leaf blades mainly basal; glumes unawned	**9. *M.flabellata* Mez**
–	Sheaths not overlapping nor flabellately arranged below; leaf blades basal and cauline, 5–30 cm long; glumes mucronate or short-awned	**15**. ***M.montana* (Nutt.) Hitchc.**
23	Panicles diffuse, branches capillary; pedicels 4–40(–50) mm long, much longer than the spikelets	**24**
–	Panicles not diffuse, branches not capillary branches; pedicels ≤ 3 mm long, shorter than the spikelets or equal in length	**26**
24	Culms 12–30 cm tall; leaf blades 5–8 cm long; spikelets 3–3.5 mm long, plumbeous to dark-green turning golden brown with age; ligules 0.3–0.5(–1) mm long, truncate, membranous	**20. *M.orophila* Swallen**
–	Culms 40–100 (–150) cm tall; leaf blades 10–35(–80) cm long; spikelets 3–5 mm long, usually purple to reddish-purple, occasionally green, brown or stramineous; ligules (1–) 1.8–6 (–8) mm long, obtuse to acute, firm below	**25**
25	Panicles 8–30 (–41) cm wide; pedicels 10–40(–50) mm long; primary branches widely divergent, diverging up to 100° from the culm axis	**3. *M.capillaris* (Lam.) Hitchc.**
–	Panicles (2–)3–5(–12) cm wide; pedicels ≤ 10 mm long; primary branches ascending and spreading, diverging up to 80° from the culm axis	**28. *M.rigida* (Kunth) Kunth**
26	Culms rounded near base, the base appearing terete in transverse section	**27**
–	Culms compressed-keeled near base, appearing elliptic in transverse section	**32**
27	Lower glumes 2 or 3-veined, usually 2-awned, the awns 1–3 mm long; upper glumes usually 1-awned, the awns 1–2.5 mm long; spikelets usually in pairs, the lower spikelet short- pedicelled perfect, staminate or sterile and the upper spikelet longer-pedicelled and usually perfect; culms 10–30 cm tall and decumbent sprawling near base	**23. *M.phalaroides* (Kunth) P.M. Peterson**
–	Lower glumes 1-veined and unawned; upper glumes unawned; spikelets not in pairs and all spikelets perfect; culms 30–200 cm tall and erect, rarely geniculate near base	**28**
28	Plants sprawling along the ground, culms geniculate and rooting at the lower nodes; lemmas with 3 prominent, green veins; anthers yellowish	**30. *M.setarioides* E. Fourn.**
–	Plants with culms caespitose and rooting only at the base; lemmas 3-veined, the veins not green nor prominent; anthers reddish-purple, purplish, greenish, or greenish-gray to whitish-gray	**29**
29	Glumes 3.4–8 mm long, usually as long or longer than the lemmas, strongly laterally compressed; ligules (5–)8–40(–50) mm long, strongly decurrent, often splitting into broad auricles 10–35(–50) mm long; panicles dense and spikelike, 5–12 mm wide, plumbeous with a hint of green or greenish–gray, with primary branches 1–12 mm long	**30**
–	Glumes ≤ 2 mm long, ½ as long or less than the lemmas, not laterally compressed; ligules 1–8 mm long, not decurrent and without auricles; panicles narrow and contracted to loosely contracted and spreading, (0.6–)1–5(–12) cm wide, purple, reddish-purple or purplish-green, with primary branches 0.4–10 cm long	**31**
30	Spikelets 3.4–5.6(–6) mm long; lemmas 3.4–5 mm long; glumes 3.4–5.6 mm long, unawned and without a mucro; basal leaf sheaths keeled; anthers 1.5–2.2 mm long	**13. *M.macroura* (Kunth) Hitchc.**
–	Spikelets (5.3–)6–8 mm long; lemmas 5–6.5 mm long; glumes (5.3–)6–8 mm long, sometimes mucronate or short-awned; basal leaf sheaths flattened; anthers 2.5–3.2 mm long	**19. *M.nigra* Hitchc.**
31	Culms with 4–8 nodes per culm; leaf blades 2–12 cm long; glumes 0.3–1 mm long; anthers 0.9–1.6 mm long	**31. *M.spiciformis* Trin.**
–	Culms with 1 or 2 nodes per culm; leaf blades 12–40 cm long; glumes 1–2 mm long; anthers 1.7–2.3 mm long	**32**
32	Lemmas awned, awns 10–22 mm long; glumes 1–1.7(–2) mm long, apex obtuse to subacute, sometimes erose	**28. *M.rigida* (Kunth) Kunth**
–	Lemmas mucronate or shortly awned, awns or mucros 0.5–1(–2) mm long; glumes 1.5–2 mm long, apex acute, scabrous	**17. *M.mucronata* (Kunth) Trin.**
33	Panicles and spikelets golden yellow to yellowish-brown	**34**
–	Panicles dark green, plumbeous, greenish-gray, silvery-gray, greenish-brown, purple, purplish-brown, purplish-green, reddish-purple or brownish-purple but never yellowish	**37**
34	Lemmas 1.8–2 mm long, hyaline, indistinctly 3-veined; paleas 1.7–1.8 mm long, glabrous between the veins; glumes 1.7–2.2 mm long, mucronate, the mucro up to 0.6 mm long; known only from Guatemala	**1. *M.aurea* Swallen**
–	Lemmas (2–)2.1–3.3 mm long, membranous, distinctly 3-veined; paleas 2–2.9 mm long, hairy between the veins; glumes 2–3.8 mm long, sometimes mucronate; more wide-ranging in Central America	**35**
35	Glumes unveined, smooth, translucent, somewhat lustrous and shining, apex acute without a mucro; ligules hyaline, delicate, frayed in age, becoming somewhat firm at base (3–)6–13 mm long	**38. *M.xanthodas* Soderstr.**
–	Glumes 1-veined, occasionally 2-veined or rarely 3-veined; apex acute to acuminate, usually mucronate; ligules membranous to chartaceous 6–25 mm long or 0.1–2 mm long and truncate on culm leaves	**36**
36	Ligules 0.1–2 mm long on culm leaves, usually 6–13 mm long on the innovations, apex truncate; basal sheaths becoming brown, curled and fibrillose or shredded; leaf blades usually abruptly narrowed at base (sheath and blade junction)	**2. *M.breviligula* Hitchc.**
–	Ligules 10–25 cm long on the culm leaves and lower innovations, apex acuminate; basal sheaths golden yellow with age and not fimbrillose or basally shredded; leaf blades not abruptly narrowed at the based (sheath and blade junction)	**11. *M.lehmanniana* Henrard**
37	Lemmas unawned or mucronate, the mucro, if present, less than 1 mm long; culms 100–300 cm tall	**38**
–	Lemmas awned, the awns 4–20(–25) mm long; culms 70–150(–167) cm tall	**39**
38	Panicles (8–) 15–30 cm wide, purple or brown-purplish; auricles absent; primary panicle branches pendulous to flexuous spreading, 6–25 cm long, usually 15–20 cm long below	**18. *M.mutica* (Rupr. ex E. Fourn.) Hitchc.**
–	Panicles (2–)3–8 cm wide, greenish-gray to silvery-gray or purplish; auricles present, (1–)2–4(–10) mm long; primary panicle branches closely appressed to spreading 40° from the axis, 1–15(–17) cm long	**29. *M.robusta* (E. Fourn.) Hitchc.**
39	Sheath auricles lacking or rudimentary, less than 0.5 mm long	**40**
–	Sheath auricles present, 1.5–64 cm long	**41**
40	Ligules 0.1–2 mm long on culm leaves, usually 6–13 mm long on the innovations, apex truncate; basal sheaths becoming brown, curled and fimbrillose or shredded; leaf blades usually abruptly narrowed at base (sheath and blade junction)	**2. *M.breviligula* Hitchc.**
–	Ligules 10–25 mm long on the culm leaves and lower innovations, apex acuminate; basal sheaths golden yellow with age and not fimbrillose or basally shredded; leaf blades not abruptly narrowed at the based (sheath and blade junction)	**11. *M.lehmanniana* Henrard**
41	Lemmas villous on lower 1/2–2/3 and pilose along the margins near base, (2.5–)3–3.5 mm long; panicles dark-greenish to plumbeous; upper glumes mostly awned, the awns 1–1.2 mm long; culms strigulose below the nodes	**37. *M.versicolor* Swallen**
–	Lemmas glabrous with short pubescence along the margins and midvein on the lower 1/2–2/3, 1.4–2.8 mm long; panicles greenish-brown, purplish-brown to reddish-purple; upper glumes unawned or mucronate, the mucro up to 0.4 mm long; culms glabrous, puberulent or pubescent below the nodes	**42**
42	Ligules 0.1–2 mm long on the culm leaves, apex truncate; sheath auricles 1.5–6 mm long; leaf blades usually abruptly narrowed at base (sheath and blade junction), 2–3 mm wide	**2. *M.breviligula* Hitchc.**
–	Ligules 4–15 mm long on culm leaves, apex lacerate; sheath auricles (6–)10–26 mm long below and up to 64 mm long above; leaf blades not abruptly narrowed at the base (sheath and blade junction), 2–7 mm wide	**7. *M.distichophylla* (J. Presl) Kunth**

#### ﻿1. Muhlenbergia aurea

Taxon classificationPlantaePoalesPoaceae

Swallen, Contr. U.S. Natl. Herb. 29(9): 411. 1950.

90576265-77DB-564A-9F36-129BDE55BAF1

Fig. 2A–E

##### Type.

Guatemala. Quezaltenango, in thickets at base of vertical slopes along railroad, Finca Pirineos, lower S facing slopes of Volcán Santa María, between Santa Maria de Jesus and Calahuache, 1300–1500 m, 31 Dec 1939, *J.A. Steyermark 33175* (holotype: F-1057948 [image 64109!]; isotype: US-2236470 fragm. ex F!).

##### Description.

Strongly caespitose ***perennials*. *Culms*** 70–80 cm tall, stout, compressed-keeled near base, glabrous below the nodes; ***internodes*** glabrous. ***Leaf sheaths*** glabrous below becoming scabrous near the collar; ***ligules*** 3–10 mm long, hyaline, apex attenuate, appearing shredded and withering with age; ***sheath auricles*** 4–8 mm long, often rudimentary and withering with age; ***blades*** 25–42 cm long, 2.5–5 mm wide, flat, conduplicate near base, scaberulous above, scabrous below, margins saw-toothed. ***Panicles*** 28–36 cm long, 5–10 cm wide, nodding, dense, golden yellow; ***primary branches*** 10–17 cm long, ascending, appressed to spreading up to 30° from the culm axis, without spikelets on the lower half; pedicels 1.5–2 mm long, scabrous. ***Spikelets*** 1.7–2.2 mm long, erect, yellow; ***glumes*** 1.7–2.2 mm long, as long as the floret, the upper glumes slightly longer than the lower, apex acute, scabrous, mucronate, the mucro up to 0.6 mm long; ***lemmas*** 1.8–2 mm long, hyaline, indistinctly 3-veined, pubescent on the lower 1/3 of the margins and midvein, awned, awns 10–20 mm long, flexuous borne just below the apex; ***paleas*** 1.7–1.8 mm long, a little shorter than the lemma, hyaline, glabrous; ***anthers*** not seen. ***Caryopses*** not seen.

##### Phenology.

Flowering December and January.

##### Distribution.

Known only from two collections in Guatemala, the type locality on the south facing slopes of Volcán Santa Maria in Departamento Quezaltenango between the cities of Quezaltenango and Retalhuleu (Peterson et al. 2001).

##### Ecology.

*Muhlenbergiaaurea* occurs on slopes between 1300 and 1500 meters.

##### Comments.

Swallen (1950) suggested *M.aurea* is related to *M.scoparia* Vasey “which differs in having narrower sheaths and blades, an elongate ligule, a narrow, less densely flowered, purple panicle, and longer awns.” Not much is known about *M.aurea* and currently it has not been investigated using molecular methods. However, based on morphology it is placed in M.subg.Trichochloa.

**Figure 2.**
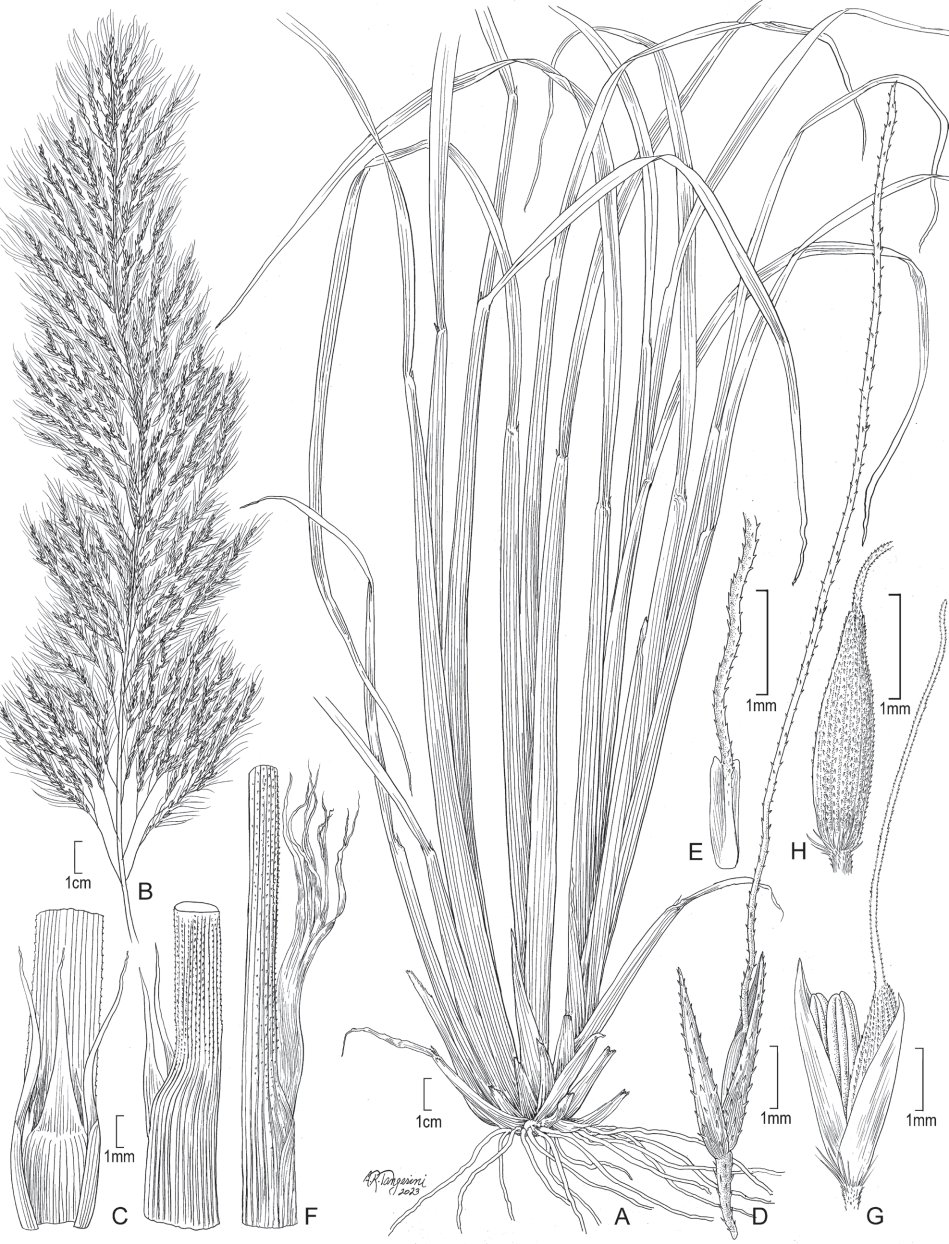
**A–E***Muhlenbergiaaurea* Swallen **A** habit **B** inflorescence **C** ligule **D** Spikelet **E** immature lemma **F–H***Muhlenbergiaxanthodas* Soderstr. **F** ligule **G** spikelet **H** lemma. **A** drawn from *J.A. Steyermark 33175* (F-1057948) **B–E** drawn from *M. de Koninck 241* (US-2182672) **F–H** drawn from *E. Matuda 4003* (US-1817864).

##### Specimens examined.

Guatemala. **Quetzaltenango**: Finca Pirineos, lower south-facing slopes of Volcán Santa María, between Santa María de Jesús and Calahuaché, *J.A. Steyermark 33175* (MO); Quezaltenango–Retalhuleu, 1480 m, 1954, *M. de Koninck 241* (US-2182672).

#### ﻿2. Muhlenbergia breviligula

Taxon classificationPlantaePoalesPoaceae

Hitchc., N. Amer. Flora 17(6):458. 1935.

F839A21F-8995-5EBB-96A3-C03CED6496B7

Fig. 3A–E

##### Type.

Guatemala, Guatemala City, collected on clay hill, 1500 m, 2 Dec 1911, *A.S. Hitchcock 9063* (holotype: US-995888!).

##### Description.

Densely caespitose ***perennials*. *Culms*** 70–140 tall, erect, stout, compressed-keeled near base, puberulent to glabrous below the nodes; ***internodes*** glabrous. ***Leaf sheaths*** 8–26 cm long, mostly longer than the lower internode, scaberulous to glabrous, basally becoming brown, curled and fibrillose or shredded, margins entire, apex pubescent on abaxial surface; ***ligules*** 0.1–2 mm long on the culm leaves, of innovations 6–13 mm long, membranous to chartaceous with a rim of hairs, lacerate, apex truncate; ***sheath auricles*** 1.5–6 mm long, whitish when present, often lacking; ***blades*** 20–50 cm long, 2–3 mm wide, usually abruptly narrowed at base (sheath and blade junction), flat to folded, scabrous above, sometimes pubescent near throat and scaberulous below. ***Panicles*** 30–51 cm long, 3–10 cm wide, nodding, somewhat dense, yellowish to purplish brown or purplish green; ***primary branches*** mostly 3–12 cm long, ascending, lax and nodding, appressed to spreading up to 40° from the culm axis; ***pedicels*** 0.5–3 mm long, scaberulous. ***Spikelets*** 2–3.5 mm long, erect, yellowish to purplish-brown; ***glumes*** 2–3.5 mm long, equal or a little longer than the floret, equal in length, lanceolate to oblong-lanceolate, 1-veined, occasionally the upper 2-veined, apex acute to acuminate, often scaberulous, sometimes mucronate, the mucros up to 0.2 mm long; l**emmas** 2.1–2.8 mm long, lanceolate to oblong-lanceolate, awned, midvein and margins pubescent to sparsely pilose on the proximal 2/3, the hairs up to 0.3 mm long, apex acute to acuminate; awns 6–18 mm long, straight or flexuous, borne just below the apex; ***callus*** sparsely pilose; ***paleas*** 2–2.7 mm long, oblong, the proximal 2/3 pubescent to sparsely pilose between the veins, apex acute; **anthers** 1–1.7 mm long, purple or yellow. ***Caryopses*** 1–1.3 mm long, fusiform, light brownish.

**Figure 3.**
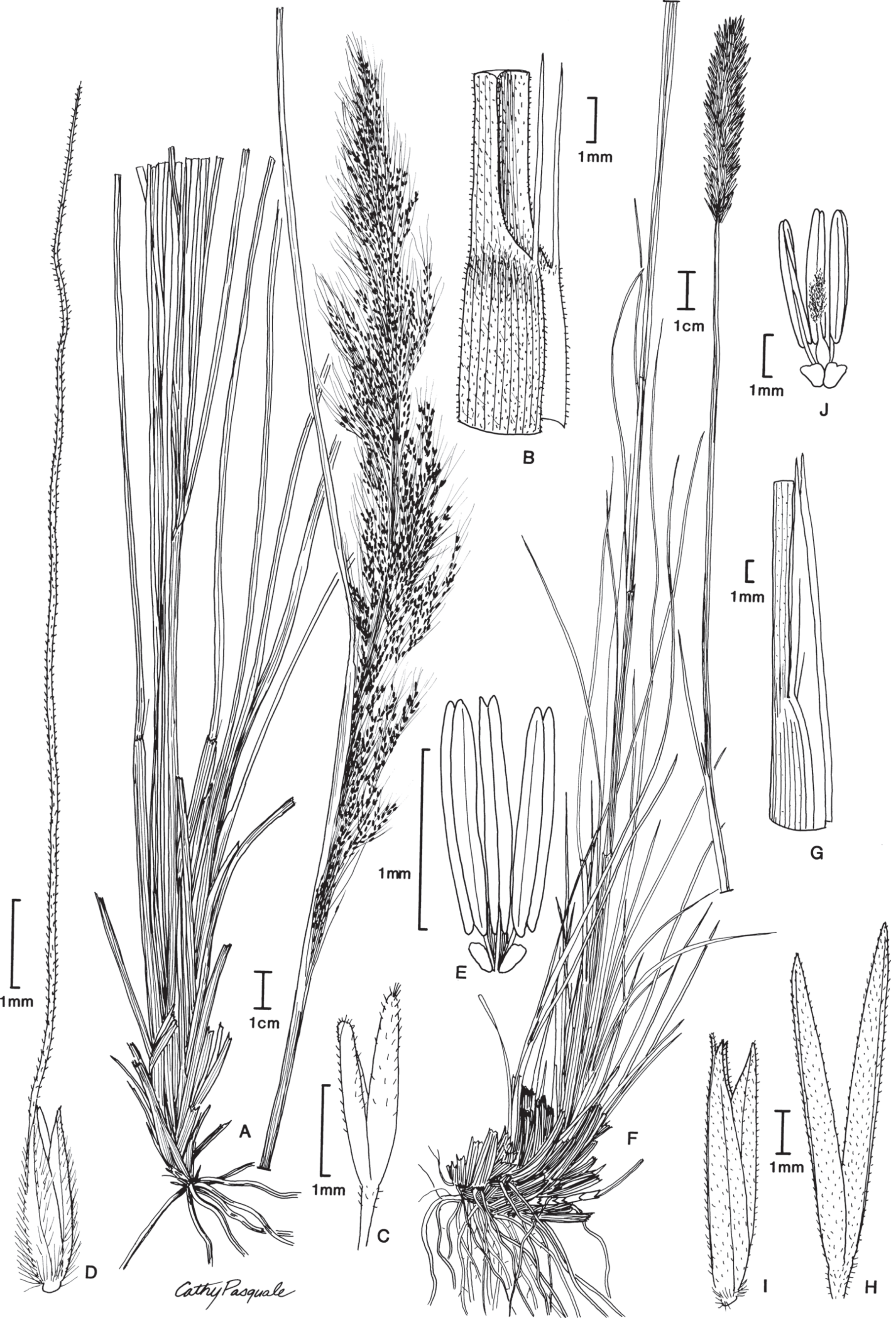
**A–E***Muhlenbergiabreviligula* Hitchc. **A** habit **B** ligule with auricles **C** glumes **D** floret **E** stamens and lodicules **F–J***Muhlenbergianigra* Hitchc. **F** habit **G** ligule **H** glumes **I** floret **J** stamens, pistil, and lodicules. **A–E** drawn from *A. Molina R. 25227* (US-2942492) **F–J** drawn from *S.D. Koch 76255* (US-2824590).

##### Phenology.

Flowering October through December.

##### Distribution.

Chiapas, México, southeast to Guatemala, Honduras, El Salvador, and Nicaragua (Peterson et al. 2001; Menjívar Cruz et al. 2021).

##### Ecology.

*Muhlenbergiabreviligula* occurs on rocky slopes, along drainages, and in open pine-oak woodlands; 650–2530 m.

##### Comments.

This species is morphologically similar to *M.emersleyi* Vasey, a species common in southwestern USA and northern México. However, *M.breviligula* differs in having culms with a short ligule 0.2–2 mm long (ligules 10–25 mm long in *M.emersleyi*), leaf blades 2–3 mm wide (2–6 mm wide in *M.emersleyi*), and persistent fimbrillose basal sheaths (not as persistent in *M.emersleyi*) [Soderstrom 1967; Peterson 2003]. *Muhlenbergiabreviligula*, a member of M.subg.Trichochloa, has been found in an unsupported clade with three other species, distributed in North America (México), Central America, and South America (Columbia, Ecuador, and Peru) [*M.lehmanniana*, *M.versicolor*, and *M.maxima* Lægaard & Sanchez Vega] (Fig. 1A; Peterson et al. 2021).

##### Specimens examined.

El Salvador. **Chalatenango**: San Fernando, creciendo a orilla de la calle, *J. González 398* (MO). Honduras. **Choluteca**: San Marcos de Colón, pine forest area near San Marcos [de Colón], *L.O. Williams & A. Molina R. 10921* (MO). **El Paraíso**: Guinope, Galeras, along road ca 10 km S of El Zamorano, open pine forest. [Originally reported from Francisco Morazán], *R.W. Pohl 12527* (MO); Alauca, Las Manos, 5 km N of Las Manos, near Los Limones, *R.W. Pohl & M. Gabel 13430* (MO). **Francisco Morazán**: Tatumbla, 24 km E de Tegucigalpa, bosque húmedo subtropical, *I. Cruz P. 75* (MO); In mountains along highway, 30 km W of Tegucigalpa, *W.A. Archer 3846* (US); Drainage of the Rio Yaguare, floresta de pino roble, acueducto de la EAP, Zamorano, *A. Molina R. 1593* (US); foothills of Mt. Uyuca, beyond Las Floras, overhanging steep bank in pine forest, *J.R. Swallen 11323* (US); San Antonio de Oriente, El Zamorano, *R.W. Pohl 12509* (MO); San Antonio del Oriente, overhanging bank, small shady canyon, about 2 km above San Antonio, *J.R. Swallen 10968* (US); Distrito Central, El Picacho, carretera en el Picacho, *Villatoro et al. 137* (MO). **Lempira**: Gracias, Río Mejocote [Rio Grande de Mejocote], 9 km de Gracias, pinares, *C. Nelson et al. 252* (MO). **Ocotepeque**: Santa Fe, about 8 km southwest of Santa Fé, near the Guatemala border, *R.A. Molina et al. 31271* (MO). **Olancho**: Jutiapa, mountain above a fire tower, Jutiapa Forest Station, between Concordia and Salamáa, pine forest on a steep slope, *R.W. Pohl & M. Gabel 13493* (MO). **Santa Bárbara**: Quimistán, Cofradia, ca. 8 km S of Cofradia, along Highway 18, open pine savana on a hill, *R.W. Pohl & M. Gabel 13392* (MO). **El Paraíso**: Dry hillsides along the Quebrada de Dantas, Rio Choluteca Valley about 16 km north of Yuscaran, *L.O. Williams & R.P. Williams 18652* (US). Guatemala. **Chiquimula**: along Rio Taco, between Chiquimula and Montana Barriol, 3–15 mi NW of Chiquimula, *J.A. Steyermark 30649* (US). Alta Verapaz: vicinity of Secanquim, *O.F. Cook & C.B. Doyle 60* (US). **Guatemala**: Guatemala City, *A S. Hitchcock 9063* (MO), *A.S. Hitchcock 9064* (US). *A.S. Hitchcock 9109* (US). **Huehuetenango**: Mountains wet of Aguacatan, on the road to Huehuetenango, open bank, *P.C. Standley 81190* (US). **Zacapa**: 13 km east of El Lobo, North-facing slope of open field and thickets, Quercus and Pinus on drier slopes, *W.E. Harmon & J.A. Fuentes 1851* (MO). Mexico. **Chiapas**: **Arriaga**: At La Mina Microwave Station, *D.E. Breedlove 56328* (CAS, MO); **Chiapa de Corso**: Above El Chorreadero, *D.E. Breedlove & R.F. Thorne 20499* (CAS, MO); 30 miles W of San Cristobal de las Casas on Mex 190 *J. N. Brunken & C. H. Perino 354* (MEXU); **Cintalapa**: Crest of the Sierra, near the microwave station of La Mina, 12 km S of Mexican Highway 190, near Rizo de Oro, *D.E. Breedlove & R.F. Thorne 20555* (CAS, MO); **Comitán de Domínguez**: 6 km N of Comitán along Mexican Highway 190, *D.E. Breedlove & G. Davidse 54872* (CAS, MO); 6 km N of Comitán along Mexican Highway 190, *D.E. Breedlove & G. Davidse 54870* (CAS, MO); **Huistán**: 10 km E of Huistán. [Tenejapa Mpo. (Huistan), *D.E. Breedlove & G. Davidse 55186* (CAS, MO); Comitán de Domínguez: 6 km N of Comitán along Mexican Highway 190, *D.E. Breedlove & G. Davidse 54872* (CAS, MO); 6 km N of Comitán along Mexican Highway 190, *D.E. Breedlove & G. Davidse 54870* (CAS, MO); **Ixtapa**: near the Zinacantán Paraje of Muctajoc. Slope with tropical deciduous forest, *D.E. Breedlove & G. Davidse 54005* (MEXU, CAS, MO), *54006* (CAS, MO), *54008* (MEXU, CAS, MO), *54009* (MEXU, CAS, MO), *54011* (CAS, MO); Intersection of the Tuxtla Gutiérrez-San Cristobal de las Casas and the Villahermosa highways, *G. Davidse et al. 30101* (MO); along road from Zinacantán center to Ixtapa near Paraje Vo Bits, *D.E. Breedlove 40709* (CAS, MO); **Jiquipilas**: Ejido Tierra y Libertad, *A. Reyes-García 5556* (MEXU); **La Independencia**: 6–10 km NNE of La Soledad along logging road from Las Margaritas to Campo Alegre, *D.E. Breedlove & B.M. Bartholomew 55670* (CAS, MO); **La Trinitaria**: a 12 km al S de la Trinitaria, camino a Cd. Cuauhtemoc, *E.M. Martínez S. & W.D. Stevens 23905a* (MEXU); 6–7 km S of La Trinitaria, *G. Davidse et al. 29938* (MO); 6 km south of La Trinitaria on Mexican Highway 190, *D.E. Breedlove & G. Davidse 55074* (CAS, MO); 20 km south of La Trinitaria, *D.E. Breedlove & G. Davidse 55067* (CAS, MO); *D.E. Breedlove 4216* (CAS, MO); **Oxchuc**: 5 km east of Oxchuc, *D.E. Breedlove & F. Almeda 57120* (CAS, MO); **San Cristóbal las Casas**: NE edge of San Cristóbal las Casas, *D.E. Breedlove 54721 & G. Davidse* (CAS, MEXU, MO); Northeast edge of San Cristóbal Las Casas, *D.E. Breedlove & G. Davidse 54746* (CAS, MEXU, MO); San Fernando: Parque Nacional del Sumidero, 20–22 km NW of Tuxtla Gutiérrez, along the road to the canyon Outlook, *G. Davidse et al. 29764* (MO); Northeast edge of San Cristóbal Las Casas, *D.E. Breedlove & G. Davidse 54728* (CAS, MO); **San Juan Chamula**, *C. Santíz Ruíz 222* (MEXU); **Tuxtla Gutiérrez**: 22 km north of Tuxtla Gutiérrez. Cliff faces and limestone bluffs with Seasonal Evergreen Forest, Calycophyllum, Zanthoxylum, Bursera, Quercus, Ficus and Erythrina at El Sumidero, *D.E. Breedlove & B.M. Bartholomew 55482* (CAS, MO); 16 km north of Tuxtla Gutiérrez on road to El Sumidero, *D.E. Breedlove & Bruce M. 55494* (CAS, MO); t El Sumidero, 22 km north of Tuxtla Gutiérrez, *D.E. Breedlove & A.R. Smith 21557* (CAS, MO); **Venustiano Carranza**: Wooded slope near the town, along the road to Pugiltic, *R. M. Laughlin 2702* (ENCB); 3 mi S of Aguacatenango along road to Pinola Las Rosas, *D.E. Breedlove & P.H. Raven 13135* (ENCB); **Villa Corzo**: Above Colonia Vincente Guerrero on road to Finca Cuxtepec [Custepec], *D.E. Breedlove 54599 & G. Davidse* (CAS, MO); near Paraje Navenchauk, *D.E. Breedlove & G. Davidse 53899* (CAS MO); Near Colonia Vincente Guerrero, *D.E. Breedlove 48584* (CAS, MO); **Zinacantán**: near Paraje Zinacantán, *D.E. Breedlove 53901 & G. Davidse* (MEXU); near the Zinacantán Paraje of Muctajoc, *D.E. Breedlove & G. Davidse*, (MEXU); *D.E. Breedlove 13807* (DS, MO). Nicaragua. **Nueva Segovia**: Santa María, km 242, 1 km al W de Santa María, *P.P. Moreno 25214* (MO).

#### ﻿3. Muhlenbergia capillaris

Taxon classificationPlantaePoalesPoaceae

(Lam.) Trin., Gram. Unifl. Sesquifl. 191–192, 296, t. 5, f. 15. 1824.

E9FD0089-5929-5814-8DAE-1EDC265D4E1C

Fig. 4A–D

Stipa capillaris Lam., Tabl. Encycl. 1: 158. 1791. Type: USA, E. Carolina, *D. Fraser s.n.* (holotype: P-LAM!; isotypes: MPU-026956 [image!], US-A866136 fragm. ex P-LAM!). ≡ Podosaemumcapillare (Lam.) Desv., Nouv. Bull. Sci. Soc. Philom. Paris, sér 2 2: 188. 1810. ≡ Trichochloacapillaris (Lam.) DC., Cat. Pl. Horti Monsp. 152. 1813. Basionym.

##### Description.

Caespitose ***perennials*. *Culms*** 60–100(–150) cm tall, erect from the base, not conspicuously branched; ***internodes*** mostly glabrous, sometimes puberulent below the nodes. ***Leaf sheaths*** glabrous or puberulent, basal sheaths terete, often becoming fibrous, but never spirally coiled, at maturity; ***ligules*** 1.8–5(–10) mm long, membranous, firm, strongly decurrent, obtuse; ***blades*** 10–35(–80) cm long, 2–4 mm wide, flat or involute, smooth abaxially, scabrous adaxially. ***Panicles*** 15–50(–60) cm long, 8–30(–41) cm wide, longer than wide, diffuse; ***primary branches*** 2–20 cm long, capillary, diverging 30–100° from the culm axis, naked basally, lower branches with 5–20 spikelets; ***pedicels*** 10–40(–50) mm long, longer than the spikelets, capillary, flexible. ***Spikelets*** 3–5 mm long, usually purple, occasionally green, brown, or stramineous; ***glumes*** (0.3–)1–1.5(–2) mm long, usually less than 1/2 as long as the lemmas, subequal, glabrous; ***lower glumes*** 1-veined, usually unawned, rarely awned, awns 1–3 mm long; ***upper glumes*** 1-veined, rarely 3-veined, acute to acuminate, often erose, usually unawned, rarely awned, awns 1–3(–5) mm long; ***lemmas*** 3–5 mm long, lanceolate, not shiny, calluses short pubescent, apices scabrous, acuminate, sometimes with 2 setaceous teeth, teeth to 1 mm long, unawned or awned, awns 2–13(–18) mm long, clearly demarcated from the lemma bodies; ***paleas*** 2–4.5 mm long, lanceolate, acuminate, usually unawned; ***anthers*** 1.5–2 mm long, purple. ***Caryopses*** 2–2.5 mm long, narrowly elliptic, brownish.

##### Distribution.

*Muhlenbergiacapillaris* ranges from the southeastern United States, the Caribbean coast of México (Quintana Roo), extending to Guatemala, Bahamas, and various Caribbean islands (Peterson et al. 2001). It is also grown as an ornamental. This species was reported from the Yucatán (Dávila et al. 2018) but we have been unable to locate a specimen to verify this record.

##### Ecology.

*Muhlenbergiacapillaris* occurs in open woodlands, pine-oak forests, savannahs and on rock outcrops; 0–2020 m.

##### Comments.

*Muhlenbergiacapillaris* can be separated morphologically from *M.rigida* in having panicles 8–30(–41) cm wide with open, diffuse branches that are strongly divergent, whereas *M.rigida* has loosely contracted panicles 2–5(–12) cm wide with appressed to ascending branches spreading up to 80° from the culm axis. *Muhlenbergiacapillaris*, a member of M.subg.Trichochloa, is found in a strongly supported clade sister to *M.expansa* (Poir.) Trin., a species from the southeastern USA (Fig. 1; Peterson et al. 2021).

##### Specimens examined.

Guatemala. **Guatemala**: Guatemala City, open prairie, *A.S. Hitchcock 9141* (US-995855); **Huehuetenango**: Rocky dry slopes above San Ildefonso, Ixtahuacan, *J.A. Steyernmark 50673* (US-1935074, US-2208677); **Quiché**: Chichicastenango, 1 km north of Chichicastenango. Small “prairie” next to a milpa, shallow soil on sandstone, *W.E. Harmon 4364* (MO), mts. E of Quiche, *V. Grant 645* (US-1818233). Mexico. **Chiapas**: near ranch house on S edge of Teopisca, *D.E. Breedlove & P.H. Raven 13097* (MICH); Marsh near Teopisca, *D.E. Breedlove & G. Davidse 54803* (MICH, CAS, MO); Teopisca, slope at W edge of Teopisca, *D.E. Breedlove & J.L. Strother 46373* (CAS, MO). **Quintana Roo**: **José María Morelos**: Lake Chichancanab (Laguna Chan-kabnab), 28–29 July 1932, *J.R. Swallen 2726* (MO, US-1537112, US-3090503).

**Figure 4.**
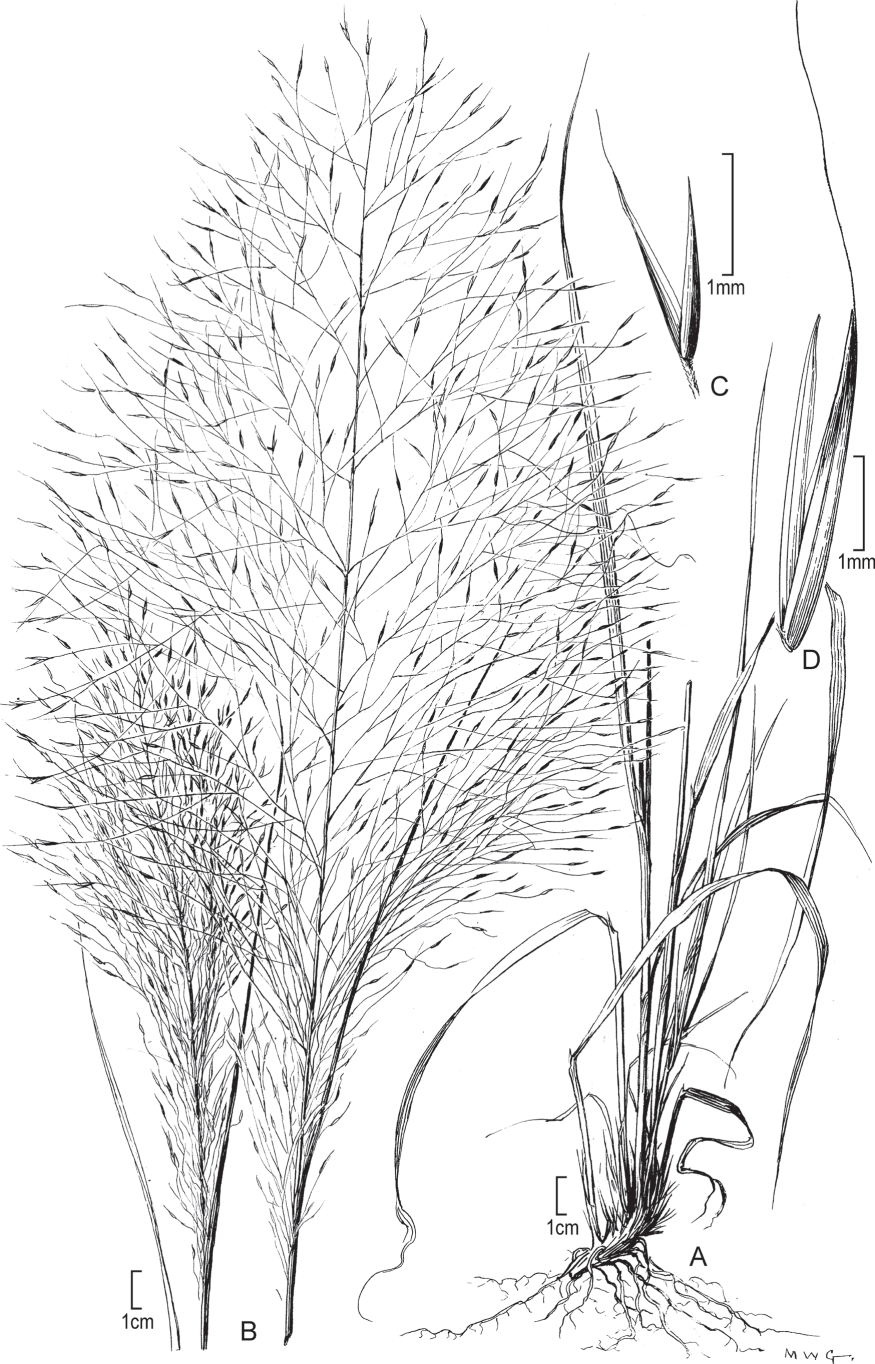
**A–C***Muhlenbergiacapillaris* (Lam.) Trin. **A** habit **B** glumes **C** floret. **A–C** drawn from *F. Lamson Scribner s.n.* (US-746068) used in Hitchcock (1935).

#### ﻿4. Muhlenbergia cenchroide

Taxon classificationPlantaePoalesPoaceae

s (Humb. & Bonpl. ex Willd.) P.M. Peterson, Caldasia 31(2): 280, f. 2 C–D. 2009.

0AC9F3A4-EC78-5863-83A6-203FE95D3814

Fig. 5A, B

Aegopogon cenchroides Humb. & Bonpl. ex Willd., Sp. Pl. 4(2): 899. 1806. Type: Venezuela, Sucre, Cumaná, *F.W.H.A. Humboldt & A.J.A. Bonpland 3002* (holotype: B-W-01637-020 [image!]). Basionym.
= Hymenothecium quinquesetum Lag., Gen. Sp. Pl. 4. 1816. Aegopogonquinquesetus (Lag.) Roem. & Schult., Syst. Veg. 1:805. 1817. Type: México, México Iperio, *Ludovicus Nee* (holotype: MA; isotype: BAA-00002156 [image!]).
= Hymenothecium trisetum Lag., Gen. Sp. Pl. 4. 1816. Aegopogontrisetus (Lag.) Roem. & Schult., Syst. Veg. 2:805. 1817. Aegopogoncenchroidesvar.trisetus (Lag.) E. Fourn., Mexic. Pl. 2:72. 1886. Type: México, México Imperio (holotype: MA; isotype: BAA-00002158 [image!]).
= Aegopogon setifer Nees, Linnaea 19(6):691. 1847. Type: México, *A. Aschenborn 132* (holotype: B; isotypes: FR-0036375 [image!], FR-0036376 [image!], US-75953 fragm. ex B!).
= Aegopogon cenchroides var. multisetus E. Fourn., Mexic. Pl. 2: 72. 1886. Type: México, Moran. in rupibus, 1840, *H. Galeotti 5808* (lectotype: BR! designated by Peterson et al., Contr. U.S. Natl. Herb. 41: 10. 2001; isolectotypes: P!, US-75958 fragm. ex P!).

##### Description.

Caespitose ***perennials*** often sprawling, occasionally with stolons. ***Culms*** (10–)25–55 cm tall, glabrous below the nodes; ***internodes*** glabrous. ***Leaf sheaths*** mostly 0.8–8 cm long, shorter than the internodes, glabrous; ***ligules*** 1–2 mm long, apex acute, lacerate; ***blades*** 1.5–6 cm long, 0.5–2 mm wide, flat, scaberulous above, smooth beneath. ***Panicles*** 2–8 cm long, 0.5–1.2 cm wide, open, loosely-flowered with recemosely arranged branches; ***primary branches*** 2–4 mm long, excluding the awns, one per node, often purplish. ***Spikelet*** fascicles of three with one sessile perfect spikelet (lateral staminate or sterile), the ***pedicels*** less than 0.2–0.5 mm long and the other two spikelets short-pedicelled, the ***pedicels*** about 0.7–1.2 mm long; ***glumes*** (1–)1.5–2.8 mm long, oblong and wider distally, 1-veined, apex deeply notched, awned, the awns 2–4 mm long, lobes triangular, acute; ***lemmas*** 2.5–3 mm long, fusiform, 3-awned, the central awns 5–13 mm long, lateral awns 2–3 mm long; ***paleas*** 2.5–3 mm long, puberulent, apex awned, the awns 1–2 mm long; ***anthers*** 1.6–1.8 mm long, yellowish to purplish. ***Caryopses*** 1–1.4 mm long, fusiform. 2*n* = 40, 60, 80.

##### Distribution.

*Muhlenbergiacenchroides* ranges from throughout México, throughout Central America (Costa Rica, El Salvador, Guatemala, Honduras, México, Nicaragua, and Panama) to South America in Bolivia, Brazil, Colombia, Ecuador, Peru, Guyana, and Venezuela (Pohl 1994a; Peterson et al. 2001).

**Figure 5.**
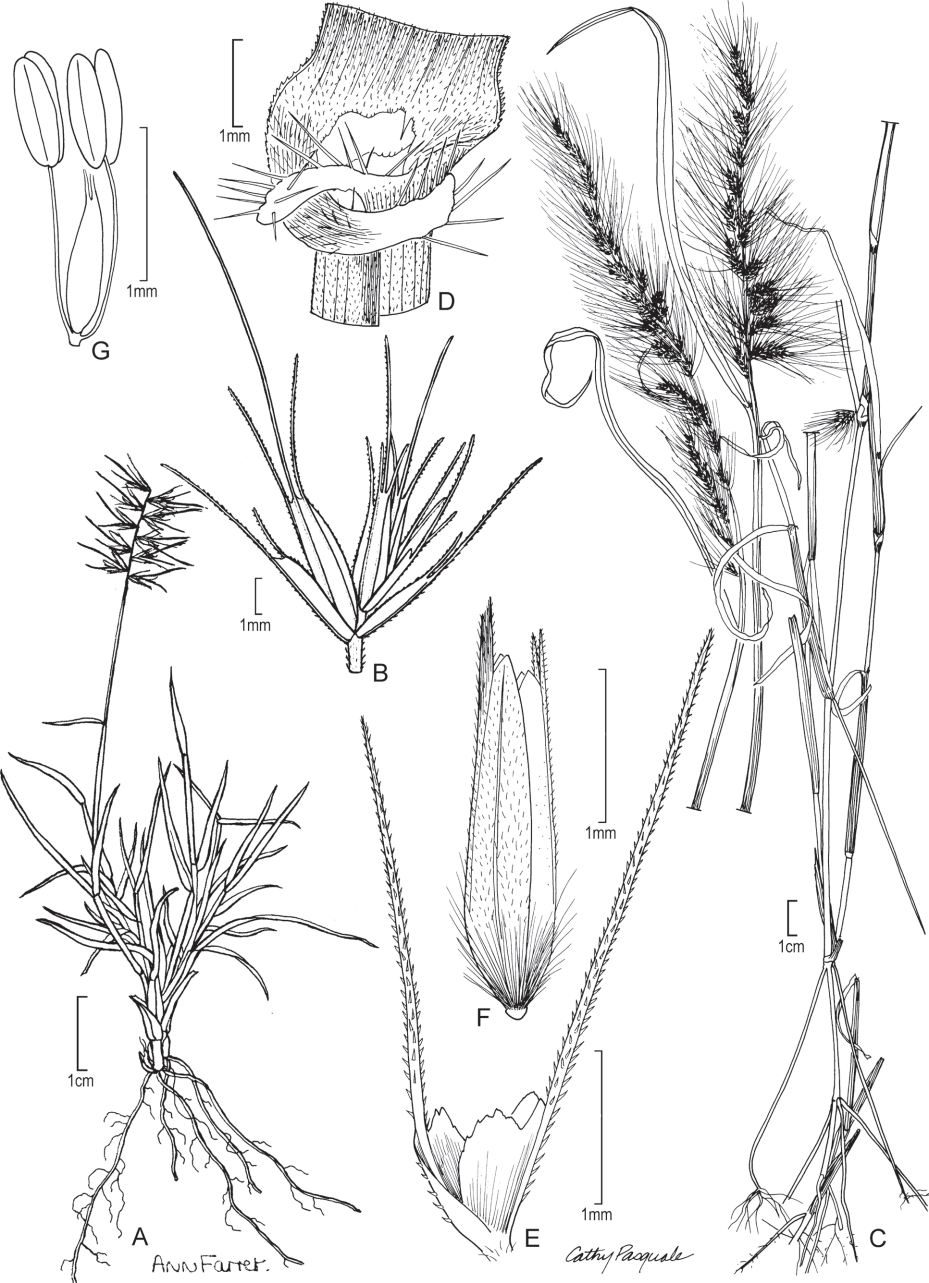
**A, B***Muhlenbergiacenchroides* (Humb. & Bonpl. ex Willd.) P.M. Peterson **A** habit **B** spikelets **C–G***Muhlenbergiadiandra* (R.W. Pohl) Columbus **C** habit **D** ligule with auricles **E** glumes **F** floret **G** stamens and pistil. **A, B** drawn from *S. Beck 7464* used in Renvoize (1998)**C–G** drawn from *P.C. Standley 44066* (US-1307162)

##### Ecology.

*Muhlenbergiacenchroides* occurs on rocky slopes, canyons, cliffs, roadcuts, arroyos, seeps, and meadows, often associated with *Baccharis* ssp., *Salvia* ssp., *Eupatorium*, *Festuca*, *Schizachyrium*, *Muhlenbergia* ssp., *Hyptis*, *Oxalis*, *Aristida*, *Bidens*, *Sporobolus*, *Carex*, *Eragrostis*, *Lupinus*, *Lycopodium*, *Jarava*, *Nassella*, *Agave*, *Thalictrum*, and *Chusquea*; 1430–3850 m.

##### Comments.

*Muhlenbergiacenchroides* can be separated from its South American sister, *M.bryophilus* (Döll) P.M. Peterson (South American) in having anthers 1.6–1.8 mm long, the perennial habit with (10–)25–55 cm tall culms, and sessile or inconspicuously pedicelled perfect spikelets usually associated with two staminate or sterile pedicelled spikelets (Tovar 1993; Giraldo-Cañas and Peterson 2009). In addition to being perennial, *Muhlenbergiacenchroides* differs from *M.uniseta* in having glumes with acute lobes and central veins extending as an awn 2–4 mm long.

*Muhlenbergiacenchroides*, a member of M.subg.Muhlenbergia, lies in a strongly supported trichotomy with *M.bryophilus* and *M.uniseta*, and this trichotomy is sister to *M.tarahumara* P.M. Peterson & Columbus (Sierra Madre Occidental, México) in M.subg.Muhlenbergia (Peterson et al. 2010b; Peterson et al. 2021).

##### Specimens examined.

Costa Rica. **Alajuela**: SW of Palmira, *A. Weston 3076* (USJ); 1 km S of Carrizal, *R.W. Pohl & G. Davidse 11499* (CR); 6.5 km W of Varablanca, *R.W. Pohl & C. Calderón 10278* (CR); 0.5 km W of Varablanca, roadside, *R.W. Pohl & C. Calderón 10257* (CR). **Cartago**: Reserva Biológica Tres de Junio, *J. Gómez-L. & D. Rivera 1221* (USJ); Interamerican Highway, about km 82, *L. Clark et al. 1570* (USJ, MO, US); Cordillera Central, lower slopes of Volcán Irazú, 1 km below San Juan de Chicoa, *R.W. Pohl & G. Davidse 11421* (CR); Reserva Forestal Río Macho, Cuenca del Savegre, Cerro de la Muerte, Carretera Interamericana, entre km 93/94, *A. Rodríguez 4227* (CR, INB); Estación Cuericí, camino a la Auxiliadora, 2 km E de Villa Mills, *B. Gamboa R. 753* (INB); Salsipuedes, bosque secundario y turberas en las cabeceras del rio Humo, *J.F. Morales 6223* (INB, MO); Santa Rosa, *E. Alfaro 5132* (INB); San Ramón de Tres Ríos, Westl. Talhang des Río Tiribí, untere Nebelwaldregion, Weg Einschmitt, vollsonniger Lehmhang ohne Gehölze, *H. Kuhbier 214* (CR); **Heredia**: *P. Döbbeler 2010* (MO); San José de la Montaña, *M. Montiel s.n.* (USJ); Monte de la Cruz, R. Ocampo 1251 (CR); 2 km N of Porrosatí, *R.W. Pohl & M. Gabel 13673* (CR); San José de la Montaña, D. Santamaría 3092 (INB); Parque Nac. Braulio Carrillo Porrosatí, Río Ciruelas, *R. Rivera 457* (MO). **San José**: Santa Maria de Dota, *J. Bustamante 218* (INB); Hda. Tiquires, Bosque primario, robledales y potreros en la Fila Aguabuena cerca de la Laguna, *J.F. Morales 4232* (MO, INB); Bosque secundario en la cima del Cerro Pico Alto, cabeceras del rio Poas, El Cidral, *J.F. Morales 1725* (INB); Copey, Cerro Vueltas, bosques enanos transición a páramo y páramos en la cima, *L. G. Clark 1570* (INB); Carretera Interamericana, ruta al Cerro de la Muerte, 2 km después de los Chespiritos dirección a Pérez Zeledón, entre km 80–81, *A. Rodríguez 3276* (INB, MO); Dota, Reserva Ftal Los Santos, Estación Ojo de Agua, Calle a Providencia, *E. Alfaro 2223* (INB); along Carretera Interamericana between km 103 and km 106, ca. 7.7–9.7 km beyond La Georgina toward San Isidro de El General, *M. Grayum 8154* (INB); Bajo Gamboa, 1 km sobre el camino que conduce al Cerro Caraigres, *J.F. Morales 6718* (CR, INB); R.F. Los Santos. Providencia Dota, Fila Cerro Vueltas, *J.F. Morales 8412* (INB); Salsipuedes, km 69, Carretera Interamericana, *B. Hammel 5132* (INB); Direct line from Hotel La Georgina to Cerro Frío of the Cerro Buenavista complex (Cerro de la Muerte), area with television and radio towers, *G. Davidse 25028* (MO); along Carretera Interamericana between km 103 and km 106, ca. 7.7–9.7 km beyond La Georgina toward San Isidro de El General, *M. Grayum & J. Affolter 8154* (MO); Z.P. Cerros de Escazú, Cerros Escazú-La Carpintera, *J.F. Morales 1725* (MO); Cuenca Térraba-Sierpe. Estación Cuericí, camino a la Auxiliadora, bosque secundario en orillas del camino, *B. Gamboa R. 753* (MO); Los Santos, Est. Ojo de Agua, colectando en bosque y orillas del tendido eléctrico, *E. Alfaro & M. Alfaro 2223* (MO); Cerro León, camino hacia Fila Aguabuena, *A. Quesada et al. 751* (CR); Cerro León, camino hacia Fila Aguabuena, *A. Quesada et al. 755* (CR); Los Cuadros, O. Jiménez 710 (CR); Cordillera de Talamanca, 26 km N of San Isidro del General along the Carretera Interamericana, *R.W. Pohl & G. Davidse 10755* (CR); along carretera Interamericana, ca. 10 km N of San Isidro del General roadsides, *R.W. Pohl & C. Calderón 10064* (CR); Montane forest formations with open landslides and road cuts and swamps, about 22 km SE of Empalme, along the Interamericcan Highway, *G. Davidse 6452* (CR); Z.P. Cerros de Escazú Río Londres y Río Agres, bosque ripario secundario, *G. Vargas & J. Sánchez 940* (CR); Los Santos Ca. 4 km due S of La Georgina at Villa Mills off the Interamerican Hwy., *F. Almeda & K. Nakai 4824* (CR); R.F. Los Santos Camino entre San Gerardo y Carretera Interamericana, *A. Estrada et al. 2782* (CR); Cerro León, camino hacia Fila Aguabuena, *R. Chacón et al. 188* (CR); San Antonio, *S. Lobo 908* (CR); Rivas, *A. Rodríguez 6509* (INB); Páramo, *D. Santamaría 3151* (INB); Páramo, *S. Lobo 1656* (CR); Carretera interamericana Sur, *J. Gómez. 6342* (CR); 71 km from San Isidro del General on Cartago road, *H.S. McKee 11221* (US). El Salvador: **Santa Ana**, Cerro Monte Cristo, NE of Metapan, *R.W. Pohl 12581* (CR); Montecristi, *R. Villacorta & J. González 1158* (MO); Volcán de Santa Ana, *G. Davidse & R.W. Pohl 2050* (MO); Parque Nacional Montecristo, camino a Miramundo, *R.A. Carballo & J. Aldana RAC00590* (MO). **Usulutan**: Laguna de Alegría, por la entrada, *D. Williams & R.W. Williams 356* (MO). Guatemala. **Alta Verapaz**: *H. von Turckheim s.n.* (MO).). **Chimaltenango**: Vegetación del volcán de Acatenango, *M. Vélz 93.2751* (MO). **Guatemala**: Guatemala city, *A.S. Hitchcock 9108* (US). **Huehuetenango**: Carretera a la Sierra de los Cuchumatanes, M. Véliz, 96.5778 (MO); Dry oak-pine forest and ravines about 6 km S of Huehuetenango, *L.O. Williams et al. 22038* (US). **Izabal**: Between Los Amates and Izabal, Sierra del Mico, *W.A. Kellerman*, *6230* (MO). **Quetzaltenango**: Mountains above Ostucalco, *P. C. Standley 66407* (US); vicinity of Zunil, dry brushy hillside, *P.C. Standley 83211* (US).**Sololá**: Volcan Atitlán, *J. Viñals*, *s.n.* (MO). Honduras. **Comayagua**: vicinity of Siguatepeque, *P.C. Standley 56214* (US). **El Paraíso**: Mansaragua, *G. Davidse et al. 35031* (MO). **Intibucá**: Cerro San Cristóbal, *C. Nelson & R. Andino 10605* (MO). **Ocotepeque**: Nueva Ocotepeque, *W.E. Harmon & J.D. Dwyer 4123* (MO). Mexico. **Chiapas**: **Cintalpa**: Hacienda Monserrat, *C.A. Purpus 467* (MO). Larráinzar, Muctahuitz. Región Los Altos, *L. Soto-Pinto 1543* (MEXU). **Huixtlán**: Chilil, *B.Y. López-Santos 217*, *218* (MEXU). **Tenajapa**: In the Paraje of Pahal Ton, *D.E. Breedlove 12591* (MEXU). **Mapastepec**: Reserva El Triunfo, polígono 1, Dry area above Cañada Honda, *M. Heath & A. Long 1043* (MEXU). **San Cristóbal**: 10 km de la carr. San Cristóbal-Ocosingo, *Borrego 6* (MEXU); entrada a Zacualpa, 500 m carr San Cristóbal-Comitán, *S. Ochoa-Gaona et al. 4258* (MEXU). **San Juan Chamula**: Los Altos, Bautista Chico, *L. Soto-Pinto 1236*, *1264* (MEXU); *Yalchín*, *B.Y. López-Santos & F. Martínez 735* (MEXU). **Unión de Juárez**: en el camino de Talquián a Chiquihuite, *E. Martínez et al. 193976* (MEXU). **Venustiano Carranza**: Ejido “Laja tendida”, km 17 de carr. Venustiano Carranza-Tuxtla Gutíerrez, aprox. 2 km a Flores Magón, *A. Miranda S. 1237* (MEXU); *G. Davidse 9449* (MO). Nicaragua. **Chontales**: 3 miles southeast of Juigalpa, route 7, roadside and swampy woods [Seymour series], *D.A. Dudey 1602* (MO). **Estelí**: Mechapa, *D.A. Hamblett 992* (MO). **Rivas**: Southwest of La Virgen and northeast of San Juan del Sur, route 16 where road crossed brook, km 136 [Seymour series], *F.C. Seymour1228* (MO); Peñas Blancas, *F.C. Seymour1853* (MO). Panama. **Chiriquí**: Volcán Barú, on road to towers at top; near towers at summit, *G. McPherson 15064* (MO); 3 km S of Boquete along Rio Caldera, *P.M. Peterson & C.R. Annable 7387* (MO); Steep forested slope to W of Río Caldera, ca. 2 km NW of Bajo Mono (Boquete region), *M. Grayum et al. 6450* (MO); Camino de acceso al Parque Nacional Volcán Barú (vertiente oriental), *M. Vega & R. Rincon 194* (MO); Baru/Potrero Muleto, *G. Davidse & W.G. D’Arcy 10257* (MO); Volcán Chiriquí, *A. Weston 12375A* (CR); Volcán Chiriquí, *A. Weston 6216* (CR); Side of Barú Mt., *J.S. McCorkle 156* (US); 8 km NW of Boquete on road to Volcán Barú, slopes above Quebrada Grande, *P.M. Peterson & C.R. Annable 7361* (US); Chiriqui Volcano, *A.S. Hitchcock 8218* (US); Chiriquí Volcano, *A.S. Hitchcock 8206* (US).

#### ﻿5. Muhlenbergia ciliata

Taxon classificationPlantaePoalesPoaceae

(Kunth) Trin., Gram. Unifl. Sesquifl. 193. t.5, f.16. 1824.

3AB3A75F-C24F-5CD4-8FD2-C468DCB57E25

Fig. 6A–D

Podosemum ciliatum Kunth, Nov. Gen. Sp. (quarto ed.) 1:128–129. 1816. Type: México, Michoacán, Volcán de Jorullo, Sep, *F.W.H.A. Humboldt & A.J.A. Bonpland s.n.* (**lectotype, designated here**: P-00077293 [image!]; isolectotypes: BAA-1619 ex P!, BM!, P-00129654 [image!], US-91918 fragm. ex P!). ≡ Trichochloaciliata (Kunth) Roem. & Schult., Syst. Veg. 2:386. 1817. ≡ Polypogonciliatus (Kunth) Spreng., Syst. Veg. 1:243. 1825. Basionym.
= Muhlenbergia adspersa Trin., Mém. Acad. Imp. Sci. Saint-Pétersbourg, Sér. 6, Sci. Math., Seconde Pt. Sci. Nat. 4(3-4):291. 1841. Type: Peru, Lima, ex herb. *C.H. Mertens s.n.* (**lectotype, designated here**: LE-TRIN-1486.01 fragm. ex LE herb. Mertens!; isolectotype: US-87236 fragm. ex LE herb. Mertens!).

##### Description.

Sprawling, slender ***annuals***. ***Culms*** 8–30(–50) cm tall, glabrous, filiform, often tufted, freely branching at lower nodes; 0.2–0.5 mm diameter just below the inflorescence; ***internodes*** 6–42 mm long. ***Leaf sheaths*** (8–)20–44 mm long, glabrous or sparsely pilose along the margins, shorter than the internodes; ***ligules*** 0.2–0.8 mm long, a ciliate membrane; apex truncate; margin with a tuft of hairs up to 1 mm long; ***blades*** 1–4 cm long, 0.6–1.4 mm wide, flat or loosely involute, often sparsely pilose above, glabrous below. ***Panicles*** 4–12 cm long, 1.8–5.0 cm wide, terminal, densely flowered; ***primary branches*** 1.5–3.7 cm long spreading and reflexed at maturity up to 90° from the rachises, one per node; ***pedicels*** 0.5–3 mm long, glabrous, appressed, erect; nodes 6–13 per panicle. ***Spikelets*** appressed to the branches, overlapping; ***glumes*** 0.7–1.7 mm long, subequal, glabrous, 1-veined; apex acuminate, often mucronate; the mucro up to 0.5 mm long; ***lower glumes*** 0.7–1.5 mm long; ***upper glumes*** 0.8–1.7 mm long; ***lemmas*** 1.8–2.5 mm long, lanceolate, slender, awned, strongly 3-veined but appearing five-veined, the intermediate “nerves” actually rows of short barbs on top of folded epidermal ridges, sometimes with prominent short hairs (scabers) along the lateral veins, often appearing glabrous without magnification, awns (1–)5–11(–18) mm long, flexuous; ***callus*** minutely short pubescent; ***paleas*** 1.6–2.4 mm long, narrowly lanceolate, glabrous; ***anthers*** 0.3–0.5 mm long, yellowish. ***Caryopses*** 0.8–1.8 mm long, narrowly fusiform, brownish. 2*n* = 20.

**Figure 6.**
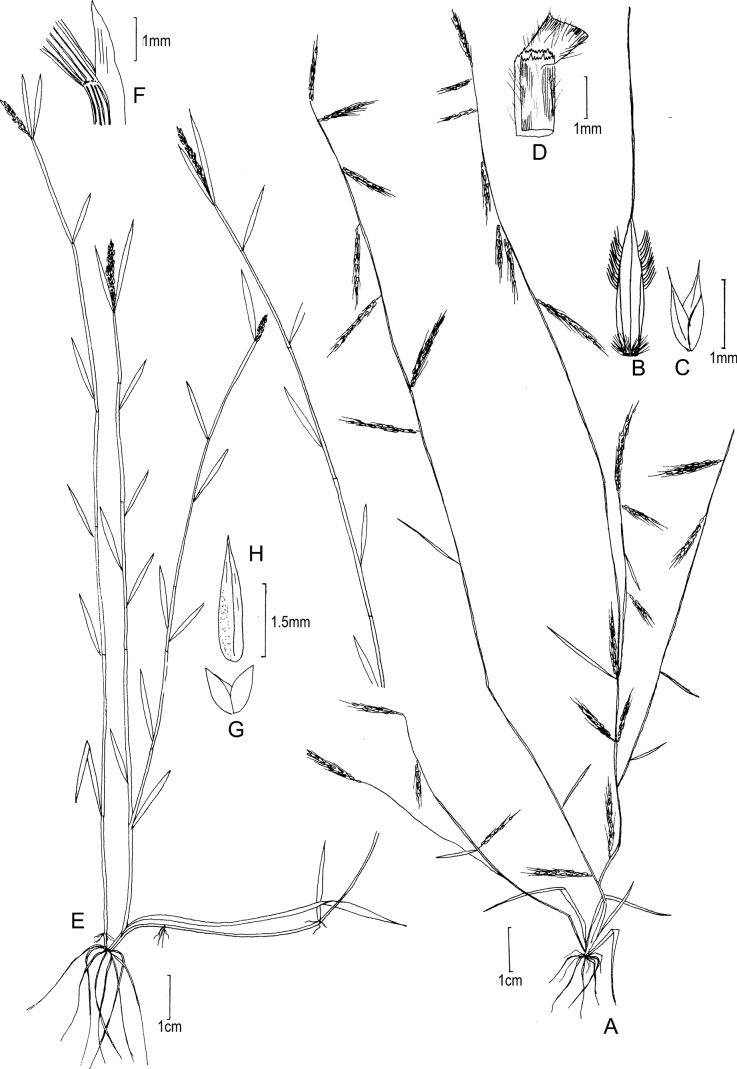
**A–D***Muhlenbergiaciliata* (Kunth) Trin. **A** habit **B** floret **C** glumes **D** ligule **E–F***Muhlenbergiavaginata* Swallen **E** habit **F** ligule **G** glumes **H** floret. **A–D** drawn from *P.M. Peterson & C.R. Annable 4541* (US, WS) **E–F** drawn from *P.M. Peterson & C.R. Annable 4111* (US, WS) both used in Peterson and Annable (1991).

##### Distribution.

*Muhlenbergiaciliata* is found throughout México and Central America (Costa Rica, El Salvador, Guatemala, Honduras, México, Nicaragua, and Panama) to Ecuador, Peru, Brazil, Bolivia, and Argentina (Peterson and Annable 1991; Peterson et al. 2001).

##### Ecology.

*Muhlenbergiaciliata* is found on moist to dry soils usually beneath taller vegetation, sandy drainages, steep rocky slopes, rock outcrops, and disturbed roadsides in woodlands with *Acacia*, *Agave*, *Andropogon*, *Bidens*, *Baccharis* ssp., *Bothriochloa*, *Eupatorium*, *Melinusminutiflora* P. Beauv., *Muhlenbergiabryophilus*, *M.flexuosa*, *M.rigida*, *Puya*, and *Salvia*; 1000–2400 m.

##### Comments.

*Muhlenbergiaciliata* can be differentiated from *M.tenella* in having lemmas with ciliate margins (not ciliate in *M.tenella*), reflexed and spreading panicles branches (tightly appressed panicle branches in *M.tenella*), regular, alternating leaf blade insertion (secund in *M.tenella*) [Peterson and Annable 1991; Peterson and Giraldo-Cañas 2011, 2012].

*Muhlenbergiaciliata* is closely related to *M.pectinata* C.O. Goodd. (North America) and *M.tenella* (Kunth) Trin. (North America, Central America, and Colombia) [Peterson and Annable 1991]. These three species form a strongly supported clade in M.subg.Muhlenbergia (Fig. 1; Peterson et al. 2010b; Peterson et al. 2021).

##### Specimens examined.

Costa Rica. **San José**: *J.F. Morales 6707A* (MO); Cerro de Piedra Blanca, above Escazú, *P.C. Standley 32486* (US); Cord. Talamanca, 14 km S of Division along the Interamerican Highway, roadside through oak forest, road fill, *R.W. Pohl & G. Davidse 11616* (US). El Salvador. **San Salvador**: Volcano of San Salvador, *A.S. Hitchcock 8938* (US). Guatemala. **Alta Verapaz**: Coban, Unter Kiefernwald, *H. Von Turckheim 3989* (US). **Guatemala**: Guatemala city, dry hill, *A.S. Hitchcock 9061* (US). Quetzaltenango: km 15.5, *M. de Koninck*, *M. 227* (US). **Huehuetenango**: San Juan Ixcoy, Sierra de los Cuchumatanes, 3 mi N of San Juan Ixocy on Hwy 9N, *P.M. Peterson & C.R. Annable 4689* (MO); Huehuetenango, *P.M. Peterson & C.R. Annable 4681* (MO). **Sacatepequez**: Magdalena, *W.A. Archer 3865* (US); Volcano Agua, open ground near Antigua, *A.S. Hitchcock 9135* (US); Volcano Agua, *A.S. Hitchcock 9136* (US). **Totonicapán**: San Francisco El Alto, *W.E. Harmon 4555* (MO). **Zacapa**: Forested slopes, Sierra de las Minas, near summit of mountain, between Rio Hondo and Finca Alejandria, *J.A. Steyermark 29683* (US). Honduras. **Copan**: Sta. Rosa de Copan, *W.A. Archer 3838* (US). **Francisco Morazán**: Santa Lucía, Quebrada Hierba Buena, *R. Clotter 87* (MO); Tatumbla, Cerro Uyuca, *I. Cruz 93* (MO); Tatumbla, Cerro Uyuca, *R.W. Pohl & M. Gabel 13414* (MO); Distrito Central, El Hatillo, *R.W. Pohl & M. Gabel 13463* (MO); Distrito Central, between El Hatillo and Los Jutes, *R.W. Pohl & M. Gabel 13790* (MO); Valle Angeles, *R. Ramos 99* (MO); Valle de los Angeles, El Balmoral, *C. Román 23* (MO); Drainage of the Rio Yeguare, *L. O. Williams 17001* (US); Cerro de Uyuca, along trail from Las Flores to La Labranza, *P.C. Standley 27380* (US); San Antonio del Oriente, *J.R. Swallen 10916* (US); Mt. Uyuca, *J.R. Swallen 11177* (US). Mexico. **Chiapas: Cacahoatán**: 3 km NE of Huixtla on hwy to Motozintla, *W.D. Stevens & E.M. Martínez 25671* (MO). **Ocozocoautla de Espinosa**: 13 km E of Ocozocoautla on Rte. 190, then N on road to Aguacera, *M.J. Huft el al. 2252* (MO). **Ixtapa**: 1 km W of Ixtapa, *F.W. Gould 12713* (ENCB). **Jitotol**: about 10 mi NE of Bochil on Hwy 195, *J.R. Reeder & C.G. Reeder 6075* (ARIZ, ENCB, MO); 4 km SE of Jitotol along road to Bochil, *G. Davidse et al. 29663* (MEXU); Slope, 10 km N of Jitotol near Rio Hondo, *D.E. Breedlove & G. Davidse 55155* (CAS); Santa Isabel near Jitotol, *A. A. Beetle M-4091* (MEXU). **Ruíz**: camino de El Zopilote a San Juan Corapan, *A. Ramos 291* (MEXU). **Teopisca**: Belem, 8 km NW of Teopisca along hwy. to San Cristobal de las casas, *G. Davidse et al. 29771* (MEXU); 8.2 mi SE de San Cristóbal de las Casas, *P.M. Peterson & C.R. Annable 4679* (US); 10.5 mi SE of San Cristobal de las Casas, *P.M. Peterson & C.R. Annable 4717* (ENCB, MEXU, US); **Venustiano Carranza**: 3 mi S of Aguacatenango along rd to Pinola Las Rosas, *D. E. Breedlove & P. H. Raven 13458* (US). Nicaragua. **Estelí**: Darailí, *W.D. Stevens 15913* (MO). Panama. **Chiriquí**: vicinity of Boquete, from Boquete to 3mi N, *W.H. Lewis et al. 332* (MO).

#### ﻿6. Muhlenbergia diandra

Taxon classificationPlantaePoalesPoaceae

(R.W. Pohl) Columbus, Aliso 28: 66. 2010 (21 May).

04C94E4D-0E5B-5A4C-A6D3-48103D07F354

Fig. 5C–G

Pereilema diandrum R.W. Pohl, Novon 2(2): 102. 1992. Type: Costa Rica, Heredia: Puente Mulas, S of San Antonio, canyon of Río Virilla, 850 m, 28 Nov 1968, *R.W. Pohl & G. Davidse 11482* (holotype: ISC-277884!; isotypes: CR-46688!, K-000308951 [image!], MO-356265 fragm. ex ISC!, US-3054594!). ≡ Muhlenbergiadiandra (R.W. Pohl) P.M. Peterson, Amer. J. Bot. 97(9): 1543. 2010 (1 Sep), isonym. Basionym.

##### Description.

Caespitose ***annuals*. *Culms*** 35–90 cm tall, erect to somewhat decumbent and rooting at the lower nodes, smooth and glabrous below the nodes; internodes 1–2 mm diameter, glabrous or slightly scabrous, often reddish. ***Leaf sheaths*** shorter than the internodes, scabrous or smooth; prophyll**s** 2–3 cm long, prominent, bifid; ***ligules*** 0.7–1 mm long, membranous, thick, apex truncate; ***blades*** 9–22 cm long, 5–9 mm wide, flat, scabrous, auriculate near base, the **auricles** 1–2 mm long, ciliate, clasping. ***Panicles*** 10–22 cm long, 1–2.5 cm wide, cylindrical, solitary, exserted or included in the sheaths below; ***primary branches*** 1–3.5 cm long, ascending mostly appressed, spreading up to 70° from the culm axis with dense fascicles of florets; ***pedicels*** 0.1–0.3 mm long, the spikelets arising just above the sterile spikelets or bristles, the **bristles** 3–5 mm long. ***Spikelets*** (fertile) 1.8–2.8 mm long; ***glumes*** 0.7–1.5 mm long, subequal, oblong to ovate, 1-veined, awned, the awns 1.5–4 mm long; ***lemmas*** 1.8–2.6 mm long, lanceolate, scabrous 3-veined, awned, the awns 10–24 mm long, straight, callus hairy, the hairs 0.6–1 mm long; ***paleas*** 1.8–2.8 mm long, slightly longer than the lemma, apex bidentate; ***stamens*** 2, ***anthers*** 0.8–1 mm long, often purple. 2*n* = 80 (Pohl and Davidse 1971).

##### Distribution.

The species is endemic to Costa Rica and is known from Provincias Alajuela, Heredia, Puntarenas, and San José (Peterson et al. 2001; Morales 2003).

##### Ecology.

*Muhlenbergiadiandra* occurs on dry to moist roadsides and disturbed sites on the margins of humid forests; 500–1800 m.

##### Comments.

*Muhlenbergiadiandra* differs from *M.pereilema* in having wide leaf blades 4–9 mm long (2–3 mm wide in *M.pereilema*), lemmas with straight awns (flexuous in *M.pereilema*), and chromosome number (Davidse and Pohl 1992; Morales 2003). This species probably forms a clade with *M.beyrichiana* Kunth, *M.pereilema*, and *M.plumiseta* Columbus within M.subg.Muhlenbergia (Peterson et al. 2021).

*Muhlenbergiabeyrichiana* is only known to occur in Brazil, Ecuador, and Peru as determined here. Earlier, it was reported in México and Central America but these are in error (Espejo Serna et al. 2000; Lægaard and Peterson 2001; Peterson et al. 2001, 2018).

##### Specimens examined.

Costa Rica. *Hoffmann 465* (US); Río Tiliri, *A. Tonduz 3121* (MO, CR). **Alajuela**: Poás, Carrillos de Poás, *A.M. Brenes 14606* (CR, US); Naranjo, puente sobre el Rio Colorado donde el Tropical Bungee, *B. Hammel 20587* (INB, CR); Naranjo, open areas on upper slopes of Cerro Espíritu Santo, 1–3 km SW of Naranjo, *A.S. Weston et al. 3852* (CR). **Heredia**: Puente de Mulas, S of San Antonio, Canyon of the Río Virilla, *R.W. Pohl & G. Davidse 11482* (MO, CR). **Puntarenas**: Monteverde, 10 km S Monteverde on road to Inter-American Highway, area of spring, *W. Haber & W. Zuchowski 9639* (MO, INB); Cordillera de Tilarán, 10 km SW Santa Elena on road from Monteverde to Inter American Highway, dry ridges and cut banks, *W. Haber & W. Zuchowski 10305* (MO, INB, US). **San José**: San Francisco de Guadalupe, *A. Tonduz 9817* (CR, MO, US); Mora, Llano Grande Puriscal, *O. Jiménez 890* (US, CR); Orillas del camino a San Juan, *O. Jiménez 161* (US); vicinity of Santa Maria de Dota, *P.C. Standley 41769* (US), *43224* (US), *44066* (US); Bordes del rio Torres, San Francisco de Guadalupe, *A. Tonduz 7198* (US).

#### ﻿7. Muhlenbergia distichophylla

Taxon classificationPlantaePoalesPoaceae

(J. Presl) Kunth, Enum. Pl. 1: 202. 1833.

11082E31-423A-5A4F-A946-A5A0D96683DD

Fig. 7F–I

Podosemum distichophyllum J. Presl, Reliq. Haenk. 1(4–5): 231. 1830. Type: México, 1831, *T.P.X. Haenke* s.n. (holotype: PRC-450429 [image!]; isotypes: MO-1837831!, US-90711 fragm ex PR!, W-0002571!). ≡ Epicampesstrictavar.distichophylla (J. Presl) M.E. Jones, Contr.W. Bot. 14: 6. 1912. Basionym.
= Muhlenbergia angustifolia Swallen, N. Amer. Fl. 17(6): 457. 1935. Type: México, near Guadalajara, on rocky hills, 11 Nov 1889, *C.G. Pringle 2346* (holotype: US-822822!; isotypes: BR-0000006863357 [image!], BR-0000006884116 [image!], CM-2819 [image!], KFTA-0002830 [image!], LE!, MEXU-00005188 [image!], MO-1837815!, UC-122455 [image!], US-995828!, US-3274342!, W-18900000582 [image!], W-19160027683 [image!]).

##### Description.

Caespitose ***perennials*. *Culms*** 100–180 cm tall, erect, glabrous to pubescent below the nodes; ***internodes*** glabrous. ***Leaf sheaths*** 8–42 cm long, longer than basal internodes, glabrous, the keels prominent, sometimes coiled to shredded below, basal sheaths compressed-keeled; ***sheath auricles*** 6–26 mm long, on lower portions and to 6.4 cm above, apex acuminate; ***ligules*** 4–15 mm long, membranous, apex finely lacerate sometimes almost to the base; ***blades*** 18–90 cm long, 2–7 mm wide, flat or folded, scaberulous to scabrous on both sides, the margins and keel saw-toothed. ***Panicles*** 35–70 cm long, 4–15 cm wide, densely-flowered, oblong, sometimes lax near apex, greenish-brown, sometimes reddish-purple; ***primary branches*** 2–15 cm long, without spikelets near the base, appressed to loosely spreading up to 60° from the rachises; ***pedicels*** 0.2–4 mm long, glabrous to scaberulous. ***Spikelets*** 1.5–2.8(–3) mm long, erect, greenish-brown, to reddish-purple; ***glumes*** 1.2–2.8 mm long, longer, as long or a little shorter than the lemma, subequal, oblong to narrowly-oblong, faintly 1-veined, hyaline, glabrous to scaberulous, usually with faint, widely scattered hairs, the hairs less than 0.1 mm long, apex acute to acuminate; ***upper glumes*** rarely mucronate, the mucro to 0.4 mm long; ***lemmas*** 1.4–2.7 mm long, lanceolate to linear-lanceolate, awned, glabrous or sometimes the margins on the lower 1/3 pubescent, the hairs to 0.2 mm long, rarely the lower 1/3 with scattered hairs, apex acute, minutely bifid, the teeth to 0.5 mm long, the awn 4–16 mm long, flexuous, often reddish-purple near base; ***callus*** usually short pilose; ***paleas*** 1.3–2.7 mm long, glabrous or with few hairs between the veins on the lower 1/3, apex acute; ***anthers*** 1.2–1.5 mm long, yellowish, sometimes reddish tinged. ***Caryopses*** not seen.

**Figure 7.**
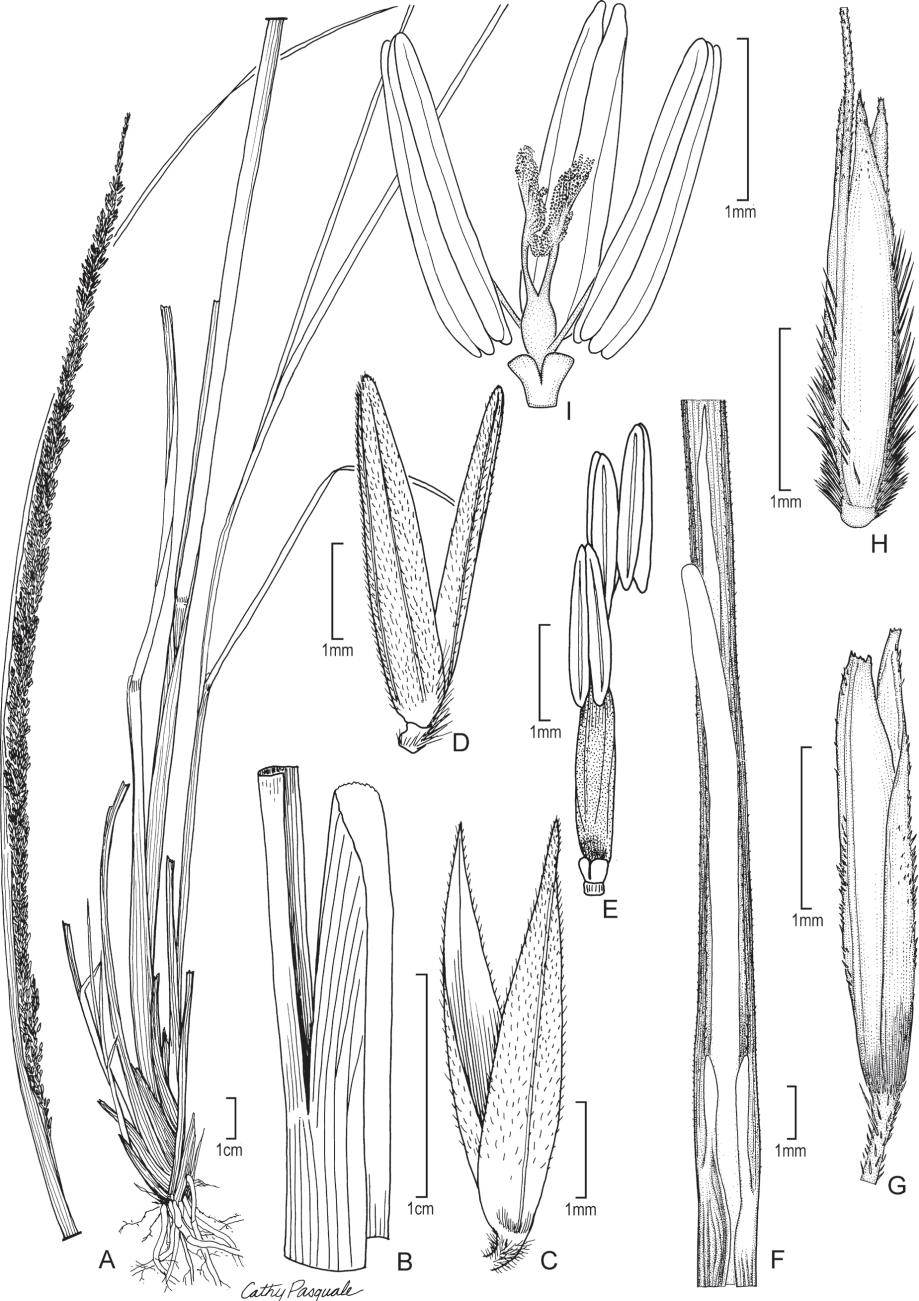
**A–E***Muhlenbergiamacroura* (Kunth) Hitchc. **A** habit **B** ligule **C** glumes **D** floret **E** stamens, pistil, and lodicules **F–I***Muhlenbergiadistichophylla* (J. Presl) Kunth **F** ligule **G** glumes **H** floret **I** stamens pistil, and lodicules. **A–E** drawn from *P.M. Peterson & C.R. Annable 5970* (US) **F–I** drawn from *L.O. Williams*, *A. Molina R. & T.P. Williams 22309* (US) and *F.W. Gould 12666* (US).

##### Distribution.

The species ranges from central México in Jalisco, Guerrero, México, Oaxaca, and Chiapas to Guatemala (Peterson et al. 2001).

##### Ecology.

*Muhlenbergiadistichophylla* occurs in open pine-oak forests and tropical deciduous forests on rocky slopes, canyons, and ravines; 400–2000 m.

##### Comments.

This species can be separated from other members of M.subg.Trichochloa in having long, acuminate sheath auricles up to 6.4 cm long on the culm, usually greater than 2 cm long on the lower innovations (Herrera Arrieta and Peterson 2018). *Muhlenbergiadistichophylla*, a member of M.subg.Trichochloa, has been found in an unsupported clade with nine other species, distributed in North America (Eastern USA, western USA, México) and Central America [*M.lindheimeri* Hitchc., *M.setifolia* Vasey, *M.dubia* E. Fourn., *M.gypsophila* Reeder & C. Reeder, *M.* x *involuta* Swallen, *M.reverchonii* Vasey & Scribn., *M.sericia* (Michx.) P.M. Peterson, *M.expansa* (Poir.) Trin. and *M.capillaris*] (Fig. 1; Peterson et al. 2021).

##### Specimens examined.

Guatemala. **Escuintla**: Rio Coyolate, near Highway CA-2, thickets on banks of Río Coyolate, *W.E. Harrmon & J.A. Fuentes 4721* (MO). **Huehuetenango**: On dry bank about 5 km. west of Huehuetenango, *L.O. Williams*, *A. Molina R. & T.P. Williams 22309* (US); Sierra de los Cuchumatanes, *P.C. Standley 81480* (US). Mexico. **Chiapas**: Escuintla, *E. Matuda 319* (MEXU); Monte Ovando, *E. Matuda 322* (MO).

#### ﻿8. Muhlenbergia diversiglumis

Taxon classificationPlantaePoalesPoaceae

Trin., Mém. Acad. Imp. Sci. Saint-Pétersbourg, Sér. 6, Sci. Math., Seconde Pt. Sci. Nat. 6,4(3–4):298. 1841.

0D866556-64D1-5E1B-B9D6-237F76597CE1

Fig. 8A–D

##### Type.

México, Porto Pedro, *Karwinsky 1393* (**lectotype, designated here**: LE-TRIN-1497.01!; isolectotypes: LE-TRIN-1497.02!, LE-TRIN-1497.03!, US-84831 fragm. ex LE-TRIN!, W-0002564!).

##### Description.

Sprawling ***annuals*. *Culms*** 16–50 cm tall, decumbent, rooting at the lower nodes; **nodes** retrorsely pilose; ***internodes*** smooth or scabridulous. **Leaf** s**heaths** 1.5–8.5 cm long, sparsely or densely pilose, hairs to 3 mm long, papillose-based; ***ligules*** 0.5–0.8 mm long, membranous, apex truncate, erose; ***blades*** 2–6 cm long, 1.5–4 mm wide, flat, bases distinctly narrowed to the junction with the sheath, surfaces scabridulous and sparsely pilose, hairs papillose-based. ***Panicles*** 6–10.5 cm long, 2.0–4.5 cm wide, open; ***primary branches*** 0.8–3.5 cm long, secund, spreading at right angles or somewhat reflexed usually lying to one side with 2–5 spikelets; ***secondary branches*** not developed; ***pedicels*** 1–5 mm long, scabrous or shortly pilose, hairs papillose-based; **disarticulation** at the base of the primary branches where there is a weak and contorted stipe. ***Spikelets*** 4–8 mm long, dimorphic with respect to the glumes, proximal spikelets on each branch almost sessile; ***glumes* of proximal *spikelets*** on each branch subequal, 0.2–0.7 mm long, orbicular, truncate, often erose or irregularly toothed, unawned; ***glumes* of distal *spikelets*** on each branch markedly unequal; ***lower glumes*** to 8 mm long, 1-veined, acute, usually awned, awns 0.5–3 mm; ***upper glumes*** orbicular, acute, sometimes awn-tipped; ***lemmas*** 4.0–7.6 mm long, linear to broadly lanceolate, light greenish, smooth or scabrous, usually with greenish veins, apices acuminate, awned, awns 6–19 mm long, usually straight, scabrous; ***paleas*** 3.7–6.8 mm long, narrowly lanceolate, coarsely papillate or almost smooth, 2-keeled, the veins prominent, scabrous, greenish, sometimes extending as minute awns, acuminate; ***anthers*** 0.4–0.8 mm long, yellowish. ***Caryopses*** 1.8–3 mm long, oblong-ovoid, flattened, brownish. 2*n* = 20.

**Figure 8.**
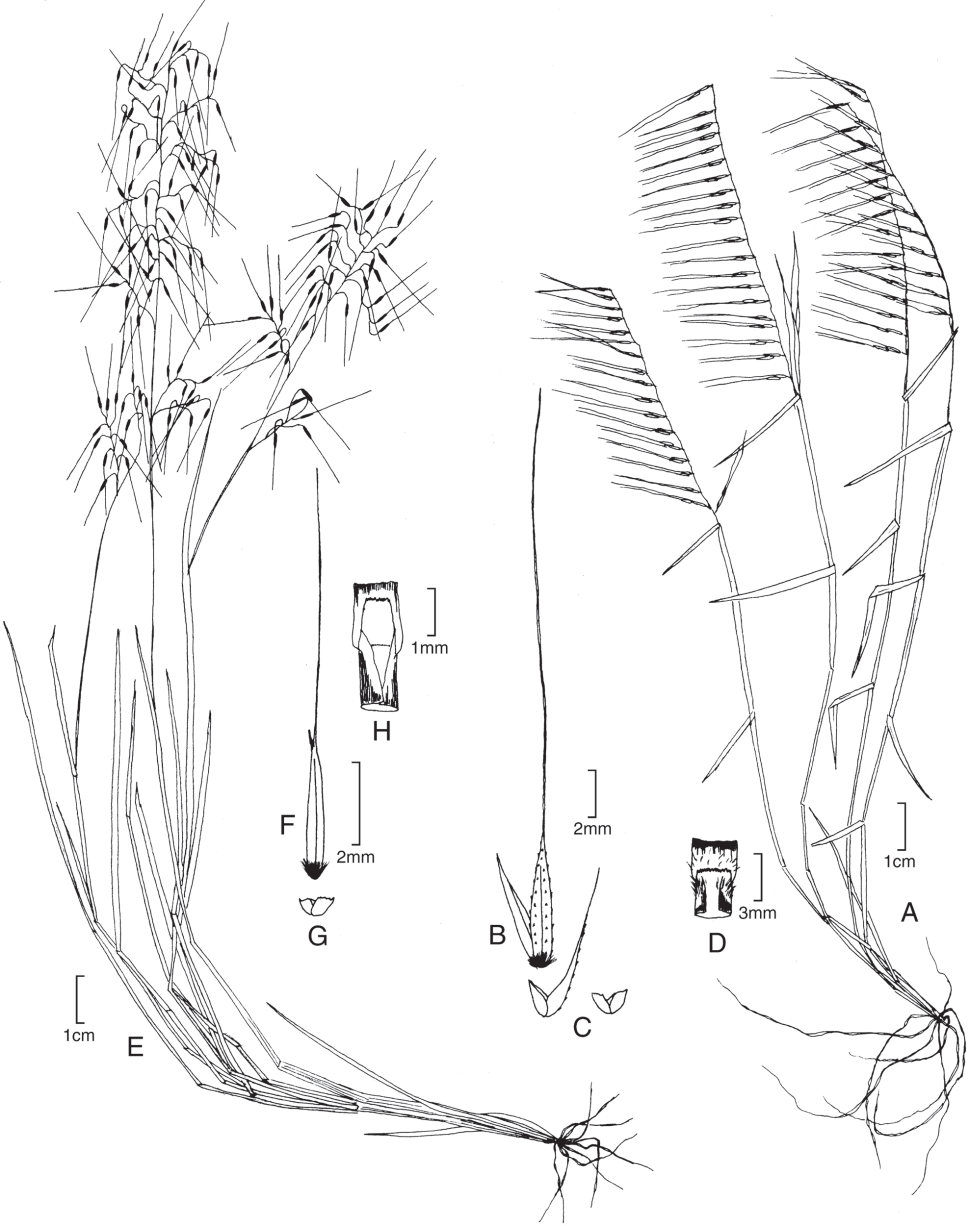
**A–D***Muhlenbergiadiversiglumis* Trin. **A** habit **B** floret **C** glumes **D** ligule **E–H***Muhlenbergiaimplicata* (Kunth) Kunth **E** habit **F** floret **G** glumes **H** ligule. **A–D** drawn from *P.M. Peterson & C.R. Annable 4158* (US, WS). **E–H** drawn from *P.M. Peterson & C.R. Annable 4598* (US, WS).

##### Distribution.

The species is native to North America, Central America, Colombia, Venezuela, Ecuador, Peru, and Argentina (Peterson and Annable 1991; Peterson et al. 2001).

##### Ecology.

*Muhlenbergiadiversiglumis* grows on moist cliffs, along water courses, sandy slopes, and road cuts, primarily in moist shaded environments of broadleaf evergreen forests and pine-oak forests; 600–2500 m.

##### Comments.

*Muhlenbergiadiversiglumis* can be differentiated from *M.ciliata*, *M.microsperma*, and *M.romaschenkoi* in having secund panicles (versus not secund in the latter three species) with each primary branch consisting of 2–5 dimorphic spikelets where the proximal spikelets have short orbicular glumes less than 1 mm long, and the distal spikelets have glumes up to 8 mm long (Peterson and Annable 1991; Giraldo-Cañas and Peterson 2009).

*Muhlenbergiadiversiglumis* is a member of M.subg.Muhlenbergia and is sister to *M.alamosae* Vasey, a species from México (Peterson et al. 2021).

##### Specimens examined.

Costa Rica. **Alajuela**: 1 km S of Carrizal, *R.W. Pohl & G. Davidse 11500* (US). **Cartago**: San Ramón, E of San José, open grassy roadside, *R.W. Pohl & Mark Gabel 13678* (MO); Road crossing of Río Reventado between Llano Grande and Tierra Blanca, gravelly river banks, *R.W. Pohl & M. Lucas 13092* (MO). **Heredia**: Porrosati, 2 km by road N of Porrosati, *R.W. Pohl & M. Gabel 13672* (MO); Carrizal, *H. Pittier 786* (US); Barba, cultures a La Esmeralda, *A. Tonduz 1692* (US). **Puntarenas**: Cordillera de Tilarán, *W. Haber & W. Zuchowski 10891* (MO, INB). **San José**: Aserri, Cuenca del Pirris-Damas, Cerros Caraigres, Falda S, Quebrada Concha, en el camino viejo a Bijagual, *J.F. Morales 5913* (MO, INB); León Cortés Castro, Z.P. Caraigres, Cuenca del Pirris-Damas, Fila El Alto. *J.F. Morales 5933* (MO, INB), Pérez Zeledón, P. N. Chirripó camino a Chirripó a orilla del sendero, Cuenca Térraba- Sierpe, *E. Alfaro*, *et al. 966* (CR, INB); Hacienda La Esperanza, La Palma, *O. Jiménez 963* (CR). San Francisco de Guadalupe, cultivos, *O. Jiménez s.n.* (US); Jardines de San Francisco de Guadalupe, *H. Pittier 9068* (US); 11 km N of San Isidro de El General along the Carretera Interamericana, *R.W. Pohl & G. Davidse 11570* (US); Between Aserrí and Tarbaca, *P.C. Standley 41352* (US); vicinity of Santa María de Dota, moist forest, *P.C. Standley 41831* (US); vicinity of Santa María de Dota, *P.C. Standley & J. Valerio 43215* (US); La Verbena de Alajuelita, *A. Tonduz 9084* (US). El Salvador. **Ahuachapán**: Parque Nacional El Imposible: San Benito, al S del enganche de los ríos Venado y Escalares, *E. Sandoval & F. Pérez 1470* (MO). **San Salvador**: Volcán San Salvador, el Boqueron, disturbed secondary forest bordering pasture and cafetale, *A. Monro et al. 2179* (MO); Volcano of San Salvador, *A.S. Hitchcock 8929* (US); Volcano of San Salvador, *A.S. Hitchcock 8939* (US); Volcano of San Salvador, *A.S. Hitchcock 8940* (US). Guatemala. **Alta Verapaz**: near San Cristóbal Verapaz, wet thickets and second growth forest, *L.O. Williams et al. 42228* (MO); Coban, *H. Von Turckheim s.c.* (US); Coban, Maisfeldem. unter Kiefernald, *H. Von Turckheim 3988* (US). **Guatemala**: Guatemala City, *A.S. Hitchcock 9049* (US). **Huehuetenango**: 21 mi NW of Huehuetenango on Pan American Hwy. 1, *P.M. Peterson & C.R. Annable 4682* (MO). **Jalapa**: Mountains along the road between Jalapa and Paraiso, *P.C. Standley 77358* (US). **Jutiapa**: Volcan Chingo, *W.C. Shannon 3699* (US). **Quiche**: Chichicastenango, 4 km S of Chichicastenango, steep wooded hillside dominated by pine and oak, *W.E. Harmon 4613* (MO). **San Marcos**: Wet mountain forest near Aldea Fraternidad, between San Rafael Pie de la Cuesta and Palo GPoales, west facing slope of the Sierra Madre Mountains, *L.O. Williams et al. 25993; L.O. Williams et al. 25971* (US); Montane cloud forest area on outer slopes of Tajumulco Volcano, Sierra Madre Mountains about 8–10 km west of San Marcos, *L.O. Williams et al. 26928* (US). Honduras. **Francisco Morazán**: Tatumbla, Cerro Uyuca, *R.W. Pohl 12493* (MO); San Antonio del Oriente, *J.R. Swallen 10975* (US); forest with with *Pinus* and *Liquidambar* entre Peña Blanca y Lo de Ponce, *L.O. Williams & A. Molina R. 17126* (US). Mexico. **Chiapas. Angel Albino Corzo**: slopes of Río Cuxtepec. along stream below Finca Cuxtepec, *D.E. Breedlove & J.L. Strother 46692* (CAS, MO). **Motozintla**: Sierra Madre de Chiapas 5 mi NW of Motozintla de Mendoza on road to El Porvenir, *P.M. Peterson & C.R. Annable 4707* (ENCB, MEXU, US, MO); W side of Cerro Mozotal, 11 km NW of the junction of the road to Motozintla along the road to El Porvenir and Siltepec, *D.E. Breedlove & B.M. Bartholomew 55714* (CAS, MO). **Zinancantán**: Hwy 190, 10 mi SE of the road to Simojovel paraje of Granadia, *D.E. Breedlove 7276* (ENCB).

#### ﻿9. Muhlenbergia flabellata

Taxon classificationPlantaePoalesPoaceae

Mez, Repert. Spec. Nov. Regni Veg. 17: 213. 1921.

42CEBE07-DF65-5C87-B0F8-138C11194726

Fig. 9A–E

##### Type.

Costa Rica, San José, Cerro de Buena Vista, 3000m, 19 Jan 1891, *H. Pittier 3372* (**lectotype, designated here**: G-00192109 [image!]; isolectotypes G-00192054 [image!], US-577110!).

##### Description.

Caespitose and sprawling ***perennials*. *Culms*** 25–45 cm long, densely branched near base, decumbent and rooting below; ***internodes*** glabrous. ***Leaf sheaths*** much overlapping, flabellately arranged, glabrous, margins membranous, old sheaths flattened and papery; ***ligules*** (2.5–)3–8 mm long, membranous, hyaline, margins entire, decurrent, apex acute; ***blades*** 2–5 cm long, 1–2 mm wide, flattened to folded or involute, mainly basal, strongly ridged above with short stiff hairs, scabrous below. ***Panicles*** 3–9(–15) cm long, 2–3 cm wide, few-flowered, narrow, short-exserted, dark green; ***primary branches*** mostly 1–2 cm long, sometimes purplish with loosely appressed and ascending branches, spreading up to 30° from the culm axis, central axis slightly flattened, 2-ribbed, scabrous; ***pedicels*** 0.2–1 mm long, scabrous. ***Spikelets*** 3–4 mm long, erect, plumbeous to dark olivaceous; ***glumes*** 1.2–2 mm long, subequal, scabrid, apex obtuse to truncate, unawned; ***lower glumes*** 1.2–1.6 mm long, ovate, usually 1-veined, sometimes unveined; ***upper glumes*** 1.6–2 mm long, half as long as the lemma, oblong-obovate, usually 3-veined, but sometimes very faint, 2 or 3-toothed, the teeth 0.3–0.6 mm long, about 1/3 the length of the glume; ***lemmas*** 3–4 mm long, lanceolate, pilose below and along margins, the hairs mostly less than 1 mm long, the callus hairy, apex awned, the awns 4–8 mm long, flexuous, scabrous, olive-green, arising from a bifid apex, the teeth up to 0.5 mm long; ***paleas*** 3–4 mm long, as long as the lemma, olive-green, scabrous; ***anthers*** 1.8–2.1 mm long, fusiform, purple.

**Figure 9.**
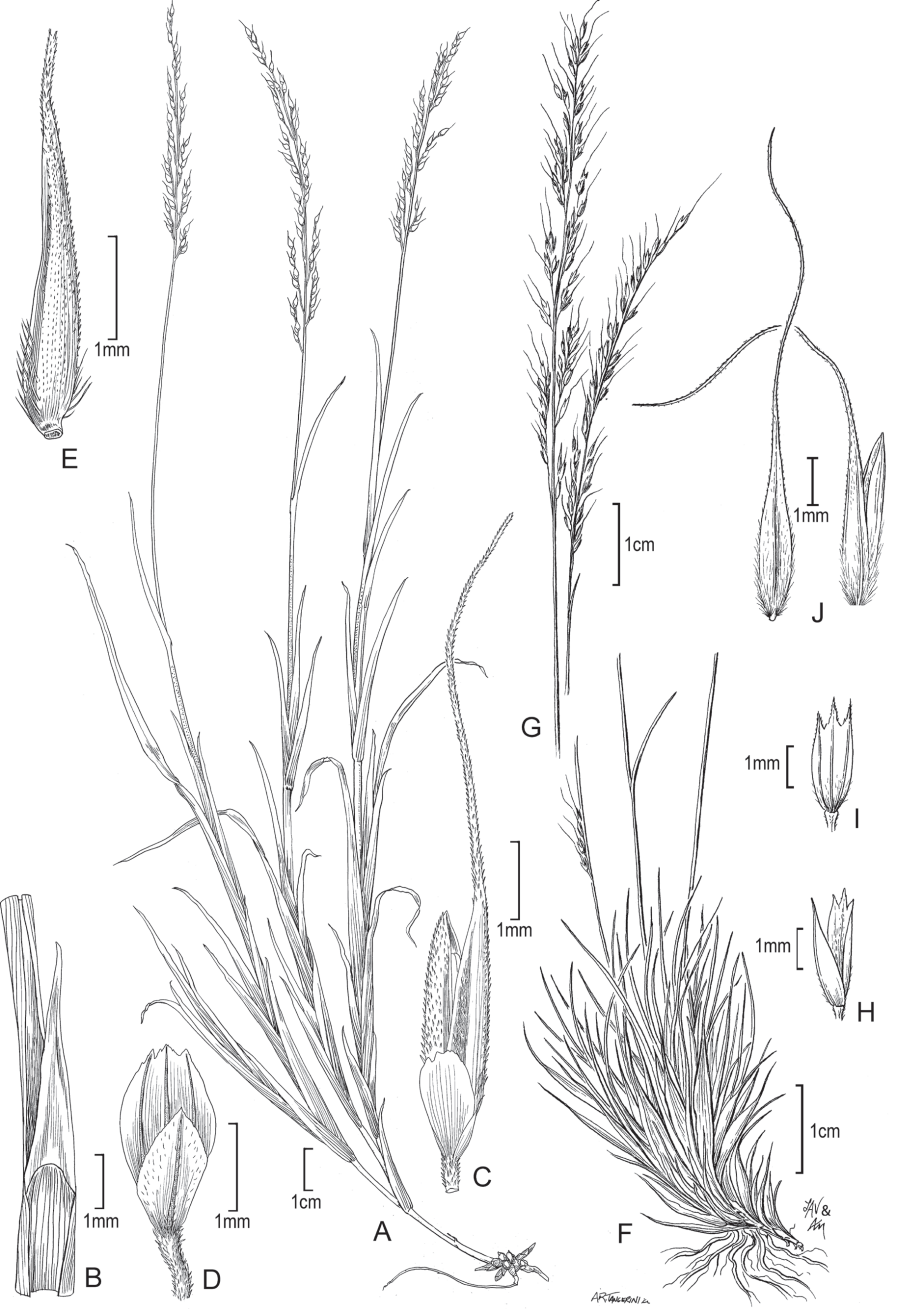
**A–E***Muhlenbergiaflabellata* Mez. **A** habit **B** ligule **C** glumes **D** floret **E** floret **F–J***Muhlenbergiamontana (Nutt.) Hitchc***F** habit **G** inflorescence **H** glumes **I** upper glume **J** florets. **A, B, D, E** drawn from *W.C. Burger & L. Gomez P. 8309* (US-2695054) **C** drawn from *G. Davidse 24971* (US-3014559) **F–J** drawn from *A.S. Hitchcock 3143* (US-995116).

##### Distribution.

The species is endemic to Costa Rica and Panama (Peterson et al. 2001).

##### Ecology.

*Muhlenbergiaflabellata* occurs in páramos between 3100–3500 m often associated with *Chusqueasubtessellata* Hitchc., *Hypericum*, ssp., *Comaristaphylosarbutoides* Lindl., *Garryalaurifolia* Hartw. ex Benth., and *Buddlejanitida* Benth.

##### Comments.

*Muhlenbergiaflabellata* is a member of M.subg.Clomena and within this clade it is sister to *M.quadridentata*, a species primarily restricted to higher elevations that is common in México and extends into Guatemala (Peterson et al. 2021). *Muhlenbergiaflabellata* can be separated from *M.quadridentata* in having short leaf blades 2–5 cm long (5–15 cm long in *M.quadridentata*), short upper glumes 1.6–2 mm long [versus (3–)3.2–4 mm long in *M.quadridentata*], and paleas scabrous throughout (versus pilose on the proximal ½ in *M.quadridentata*) [Peterson et al. 2007b; Herrera Arrieta and Peterson 2018].

##### Specimens examined.

Costa Rica. **Cartago**: Paraíso, Cerro de la Muerte, Cordillera de Salamanca, near summit of Cerro Sátira, *S. Horn 35* (CR); Paraíso, Cerro de la Muerte, *M. Kappelle et al. 2377* (CR). **Pérez Zeledón**: R. F. Los Santos, Cerro Bubis, *A. Estrada et al. 2769* (CR); P. N. Chirripó, parte superior (norte) del Valle de los Conejos, *J. Gómez 5339* (CR); R.F. Los Santos Alrededores de las torres de TV, cerro Buenavista, *L. Gómez 6354* (CR); P. N. Chirripó, alrededores de refugio, parte inferior del valle de los Conejos, *J. Gómez-L. 4503* (USJ); P. N. Chirripó, Cordillera de Salamanca, S facing slope of the Valle de los Conejos, about 1 km S of Cerro Nuevo, *S. Horn 59* (CR); P.N. Chirripó, Valle de los Conejos, *R. Ocampo 1466* (CR); P.N. Chirripó, Valle de los Conejos, *R. Ocampo 1489* (CR); Rivas, *A. Rodríguez 6422* (INB); Pérez Zeledón, Rivas, *A. Rodríguez 6541* (INB); P.N. Chirripó, refugio Los Crestones, *G. Vargas et al. 340* (USJ); disturbed paramo, Cerro de la Muerte, *R. Chazdon 447* (CR); Cordillera de Talamanca, Cerro de la Muerte, summit of Cerro Buvis, *R.W. Pohl & G. Davidse 11621* (US); Direct line from Hotel La Georgina to Cerro Frio of the Cerro Buenavista complex (Cerro de la Muerte), area with television and radio towers, G. *Davidse 24971* (US, MO); Open paramo formation with stands of *Chusquea* bamboo 1–2.5 m tall and areas of short-burned forest and original forest 5–15 m tall in protected sites, along the trail to the Valle de los Leones and the lower part of theValle de los Conejos along the upper Rio Talari, *W. Burger & L.D. Gomez 8309* (US). **San Jose**: *W.C. Burger & R. Liesner 7467* (MO); Chirripó, *G. Davidse & R. W. Pohl 1541* (MO); Buenavista, *A. Jiménez 2666* (MO); Parque Nacional Chirripó, Páramo near Albergues de los Crestones, along Río Talari near trail to Valle de los Conejos, *J. G*. *Pruski et al. 3904* (MO); Buenavista, *A. Weston 5846* (MO); Cerro Buenavista, *P.M. Peterson*, *S. Lobo*, *J. Sánchez & R. Chacón 22855* (CR, US); P. N. Chirripó Open paramo formation with stands of Chuquea bamboo 1–2.5 m tall on slopes and in the valley, short grasses and very short (30 cm)shrubs on the exposed ridges, Valle de los Conejos (upper río) Talari and trails to Cerro Chirripó and the Valle de Los Lagos, *W.C. Burger & R. Liesner 7353* (CR); P. N. Chirripó, *A. Chaverri et al. 1026* (CR); P. N. Chirripó Valle de los Conejos, *A. Chaverri et al. 1172* (CR), Panama. **Bocas del Toro**: Fábrega, A. *Weston 10190* (MO).

#### ﻿10. Muhlenbergia implicata

Taxon classificationPlantaePoalesPoaceae

(Kunth) Trin., Gram. Unifl. Sesquifl. 193: t. 5a, f. 26. 1824.

4F0B9218-88B8-53A0-86AF-1BB2AD92FA3C

Fig. 8E–H

Podosemum implicatum Kunth, Nov. Gen. & Sp. 1: 127. 1815 (1816). Type: México, Michoacán, near Lake Cuiseo and Puerto de Andaracuas, *F.W.H.A. Humboldt & A.J.A. Bonpland s.n*. (holotype: B-W!; isotype: P!). ≡ Trichochloaimplicata (Kunth) Roem. & Schult., Syst. Veg. 2: 385. 1817 ≡ Agrostisimplicata (Kunth) Spreng., Syst. Veg. 1: 262. 1825. Basionym.
= Muhlenbergia erecta J. Presl, Reliq. Haenk. 1:231. 1830, nom illeg. hom., non Muhlenbergiaerecta Schreb, 1807. Type: México, *Haenke s.n.* (holotype: PR!).

##### Description.

Caespitose slender ***annuals*. *Culms*** 15–50(–70) cm tall, mostly branched below and lax spreading or erect, scaberulous to short-pubescent below the nodes, the nodes 0.4–0.6 mm in diameter just below the inflorescence; ***internodes*** 3–9.5 cm long. ***Leaf sheaths*** 1.4–9.1 mm long, mostly shorter than the internodes, glabrous to scaberulous; ***ligules*** 1–2.5(–3) mm long, membranous to hyaline; ***blades*** 3–5(–10) cm long, 1–2.5 mm wide, flat or loosely involute, short pubescent above and mostly glabrous below, margins scabrous. ***Panicles*** 7–12(–26) cm long, 3–5(–9.2) cm wide, open, diffuse, with the peduncle included in the sheath, axis scaberulous; ***primary branches*** ascending and spreading up to 90° from the culm axis; ***pedicels*** 7–11 mm long, capillary, flexuous, delicate, smooth, nodding to reflexed, mostly purplish, thickened just below the spikelet. ***Spikelets*** 2.5–3(–4) mm long, purple; ***glumes*** 0.2–0.6 mm long, unequal, glabrous, apex truncate to broadly obtuse, often erose; ***lower glumes*** 0.2–0.4 mm long, veinless; ***upper glumes*** 0.3–0.6 mm long, 1-veined; ***lemmas*** 2.5–3(–4.5) mm long, 3-veined, appearing 5-veined because the margins are folded with rows of barbs that resemble extra veins, narrowly lanceolate, scabrous, apex awned and 2-toothed, the teeth to 1 mm long, the awns 8–26 mm long; ***callus*** with whitish appressed or spreading pubescence, the hairs up to 0.7 mm long; ***paleas*** 2.9–4 mm long, narrowly lanceolate, glabrous, apex acuminate; ***anthers*** 0.4–0.9 mm long, purple. ***Caryopses*** 1.8–2.7 mm long, narrowly fusiform, brownish. *2n* = 20 (Peterson and Annable 1991).

##### Distribution.

*Muhlenbergiaimplicata* ranges from México to Central (Costa Rica, El Salvador, Guatemala, Honduras, México, Nicaragua, and Panama) and South America (Peterson and Annable 1991; Peterson et al. 2001). In Mexico, *M.implicata* is known from Chiapas and Campeche (Sanchez-Ken 2018).

##### Ecology.

This species grows on cliffs, canyon walls, and dry to rocky roadsides in open vegetation associated with oak-pinyon-juniper woodlands; (5–) 600–2550 m.

##### Comments.

*Muhlenbergiaimplicata* is a member of M.subg.Pseudosporobolus and is sister to a central Mexican endemic, *M.seatonii* Scribn. (Fig. 1; Peterson et al. 2021).

##### Specimens examined.

Costa Rica. **Alajuela**: San Ramón, San Pedro de San Ramón, *A.M. Brenes 14966* (CR); San Ramón, Colinas de San Pedro de San Ramón, *A.M. Brenes 16704* (CR); San Ramón, San Juan cerca de San Ramón, *A. M. Brenes 16852* (CR); Orillas de camino, *O. Jiménez 44* (US); Canoas, *O. Jiménez s.n.* (US); 4 km NW of San Jose, Dry road bank in coffee plantation, *R.W. Pohl & G. Davidse 11366* (US); 1 km S of Carrizal. Open roadside in coffee plantation, *R.W. Pohl & G. Davidse 11501* (US). **Cartago**: *R.W. Pohl & M. Lucas 13152* (MO). **Heredia**: *M. Grayum 9600* (MO). **San José**: *R.W. Pohl & M. Lucas 12997* (MO); Puriscal, 10 km antes de Puriscal, *R. Ocampo1123* (CR); San José, *J.J. Cooper 5996* (US); along railway, *A.S. Hitchcock 8511* (US); San José, *A.S. Hitchcock 8459* (US); San Francisco de Guadalupe, sobre paredón, *O. Jiménez 5* (US); 13 km N of San Isidro de El General along the carretera interamericana, busy roadside in forest, *R.W. Pohl & G. Davidse 11628* (US); vicinity of Santa Maria de Dota, in potrero, *P.C. Standley 41586* (US); vicinity of Santa Maria de Dota, in cafetal, *P.C. Standley 41608* (US); vicinity of Santa Maria de Dota, brushy slope, *P.C. Standley & J. Valerio 43218* (US). El Salvador. **La Libertad**: Volcán San Salvador, *P. Bernhardt & E.A. Montalvo 79* (MO); Volcano of San Salvador, *A.S. Hitchcock 8948* (US). Guatemala. **Alta Verapaz**: Coban, *H. Von Turckheim 3990* (US); Coban, *H. Von Turckheim s.n.* (US). **Chimaltenango**: along road from Chimaltenango to San Martin Jilotepeque, *P.C. Standley 57892* (US); along road between Chimaltenango and San Martin Jilotepeque, oak forest, *P.C. Standley 80877* (US). **Guatemala**: Guatemala City, *A. S. Hitchcock 9030* (US); Guatemala City, *A.S. Hitchcock 9013* (US); Barranca north of Guatemala City, *W. Popenoe 736* (US). **Huehuetenango**: Sierra los Cuchumatanes. 8 mi N of Huehuetenango on Pan American hwy CA 1, slopes above Rio, Quercus forest, *P.M. Peterson & C.R. Annable 4701* (MO, US); About Laguna de Ocubila, east of Huehuetenango, dry open bank, *P.C. Standley 82631* (US); Pine-oak forest region, canyon at head of Rio Chixoy, about 10 km southwest of Huehuetenango, *L.O. Williams et al.22548* (US); Barranco in oak forest near Ocubila, 10 km west of Aguacatan, *L.O. Williams & T.P. Williams 21785* (US); Open, wet, boggy meadow 3 km S of Huehuetenango, *L.O. Williams et al. 22105* (US). **Jalapa**: Jalapa, *P.C. Standley 76829* (MO); vicinity of Jalapa, damp thicket, *P.C. Standley 76399* (US); vicinity of Jalapa, *P.C. Standley 76410* (US). **Sacatepequez**: Magdalena, *W. A*. *Archer 3867* (US); Above Pastores, sand along stream, *P.C. Standley 60846* (US). Mountains near Santa Maria, *Weatherwax 173* (US). Honduras. **El Paraíso**: Guinope, drainage of the Rio Yeguare, en el pantano, llanos y potreros empantanados de Galeras y Llano de Lizapa, *A. Molina 3367* (MO); Cumbre on Yuscaran road, rocky slope in pine forest, *P.C. Standley 29360* (US). **Francisco Morazán**: Distrito Central, Colonia Miraflores, *A. Díaz 244A* (MO); San Antonio de Oriente, Las Mesas, *R.W. Pohl 12521* (MO); Distrito Central, between El Hatillo and Los Jutes, *R.W. Pohl & M. Gabel 13791* (MO); Las Casitas, L. *Villela 129* (MO); Cerro la Uyuca, La Labranza and vicinity, along trail to summit, moist thicket, trail to La Labranza, *P.C. Standley 28299* (US); vicinity of Suyapa, *J.R. Swallen 11271* (US); San Antonio del Oriente, *J.R. Swallen 10915* (US); Drainage of the Rio Yeguare, *L.O. Williams 16905* (US). Mexico. **Campeche**: **Campeche**: 2.5 km al oeste de San Francisco Kobén, 5m, 20 Sep 2003, *C. Gutiérrez Báez 7909* (MEXU-1338596). **Chiapas**: 10.5 mi SE of San Cristobal de las Casas, *P.M. Peterson & C.R. Annable 4721* (ENCB, MEXU, US); near San Cristóbal, *A.A. Beetle 3963* (MEXU); W edge of San Cristóbal de las Casas, *D.E. Breedlove 53975* (NY); 2 mi S of Tuxtla Gutiérrez along road to Villa Flores, *D.E. Breedlove & P.H. Raven 13357* (US). **La Trinitaria**: 20 km S of La Trinitaria, *D.E. Breedlove & G. Davidse 55055* (CAS, MO); 6–7 km S of La Trinitaria, *G. Davidse et al. 29972* (MO). **Ixtapa**: near the Zinacantán Paraje of Muctajoc, *D.E. Breedlove & G. Davidse 54021* (CAS, MO); near Ixtapa, *D.E. Breedlove & G. Davidse 54298* (CAS, MO); near the Zinacantán Paraje of Muctajoc, *D.E. Breedlove & G. Davidse 53991* (CAS, MO). **Pueblo Nuevo Solistahuacán**: Clínica Yerba Buena, 2 km NW de Pueblo Nuevo Solistahuacán, *D.E. Breedlove & P.H. Raven 19825* (ENCB, TAES, US). **San Cristobal de las casas**: S end of the valley of San Cristóbal Las Casas, *D.E. Breedlove & G. Davidse 54374* (CAS, MO). **Teopisca**: N of Teopisca, *D.E. Breedlove & G. Davidse 54770* (CAS, MO); 7 km NW of Teopisca along hwy. to San Cristobal de las Casas, *G. Davidse*, *M. Sousa*, *O. Téllez*, *E. Martinez & J. Davidse 29826* (MO, US); 6 km W of Teopisca, *F.W. Gould & S.L. Hatch 14420* (US). Nicaragua. **Estelí**: Las Pilas, *P.P. Moreno 22341* (MO). **Jinotega**: Mountain slopes and wooded ravines of Cordillera Central de Nicaragua, 2–4 km S of Jinotega, grass in clearing, *L.O. Williams et al. 23554* (MO); Jinotega, *L. O. Williams et al. 27924* (MO). Panama. **Chiriquí**: *G. Davidse & W.G. D´Arcy 10382* (MO); *J.A*. *Duke 9197* (MO); *M. Nee 14135* (MO).

#### ﻿11. Muhlenbergia lehmanniana

Taxon classificationPlantaePoalesPoaceae

Henrard, Meded. Rijks-Herb. 40: 49. 1921.

1A7FF59C-F456-514D-A7FE-954A9590FEAE

Fig. 10G–K

= Muhlenbergia attenuata Swallen, Ann. Missouri Bot. Gard. 30 (2): 138. 1943. Type: Panamá, Chiriquí, El Boquete, foothills, 1000–1300 m, *A.S. Hitchcock 8174* (holotype: US-995843!).
= Muhlenbergia multinodis Aspl., Bot. Not. 1939: 796. 1939. Type: Colombia, Cauca, Popayán, El Tambo, 1700 m, 22 Jun 1938, *Sneidern 1323* (holotype: S-R-3665 [image!]; US-2383833 fragm. ex S!).

##### Type.

Colombia, Cauca, Popayán, *F.C. Lehmann 1267* (holotype: L-0044745 [image!]; isotypes: K-000308934 [image!], NY-00381489 [image!], US-72979 fragm. ex L!, US-72977 fragm. ex L!, US-72978 fragm. ex K!).

##### Description.

Caespitose ***perennials*** covered in old sheaths. ***Culms*** (72–)90–130(–167) cm tall, erect, compressed-keeled near base, glabrous to scaberulous below the nodes; ***internodes*** glabrous. ***Leaf sheaths*** tightly imbricate below, golden yellow with age, not basally shredded; ***ligules*** 10–25 mm long, membranous, somewhat firm below; apex acuminate; ***sheath auricles*** lacking or rarely rudimentary up to 0.1 mm long; ***blades*** 20–40 cm long, 1–4 mm wide, flat to folded, scaberulous to scabrous, apex becoming narrow and threadlike. ***Panicles*** 19–45 cm long, 3–6 cm wide, dense and narrow, golden, golden-brown or purplish-green; ***primary branches*** mostly 3.5–14 cm long, and up to 19 cm long below, ascending and appressed, naked below; ***pedicels*** 0.7–3.5 mm long, scaberulous. ***Spikelets*** 2.5–3.8 mm long, erect; ***glumes*** 2.5–3.8 mm long, about equal, sometimes the upper glume, both longer than the lemmas, 1-veined, occasionally 2 or 3-veined, hispid; apex obtuse to dentate, usually mucronate, the mucro up to 0.3 mm long; ***lemmas*** 2–3.3 mm long, pilose along the veins and margin on lower 2/3, awned from just below the apex; apex obtuse, the awns 10–20 mm long, flexuous and minutely scabrid; ***callus*** with short hairs; ***paleas*** 2.3–3.2 mm long, usually shorter than the lemma, hairy between the veins on the lower ½; ***anthers*** 1.4–1.6 mm long. ***Caryopses*** 1.2–1.3 mm long, fusiform, glabrous, light brown. 2*n* = 20 +2b (Reeder 1994).

**Figure 10.**
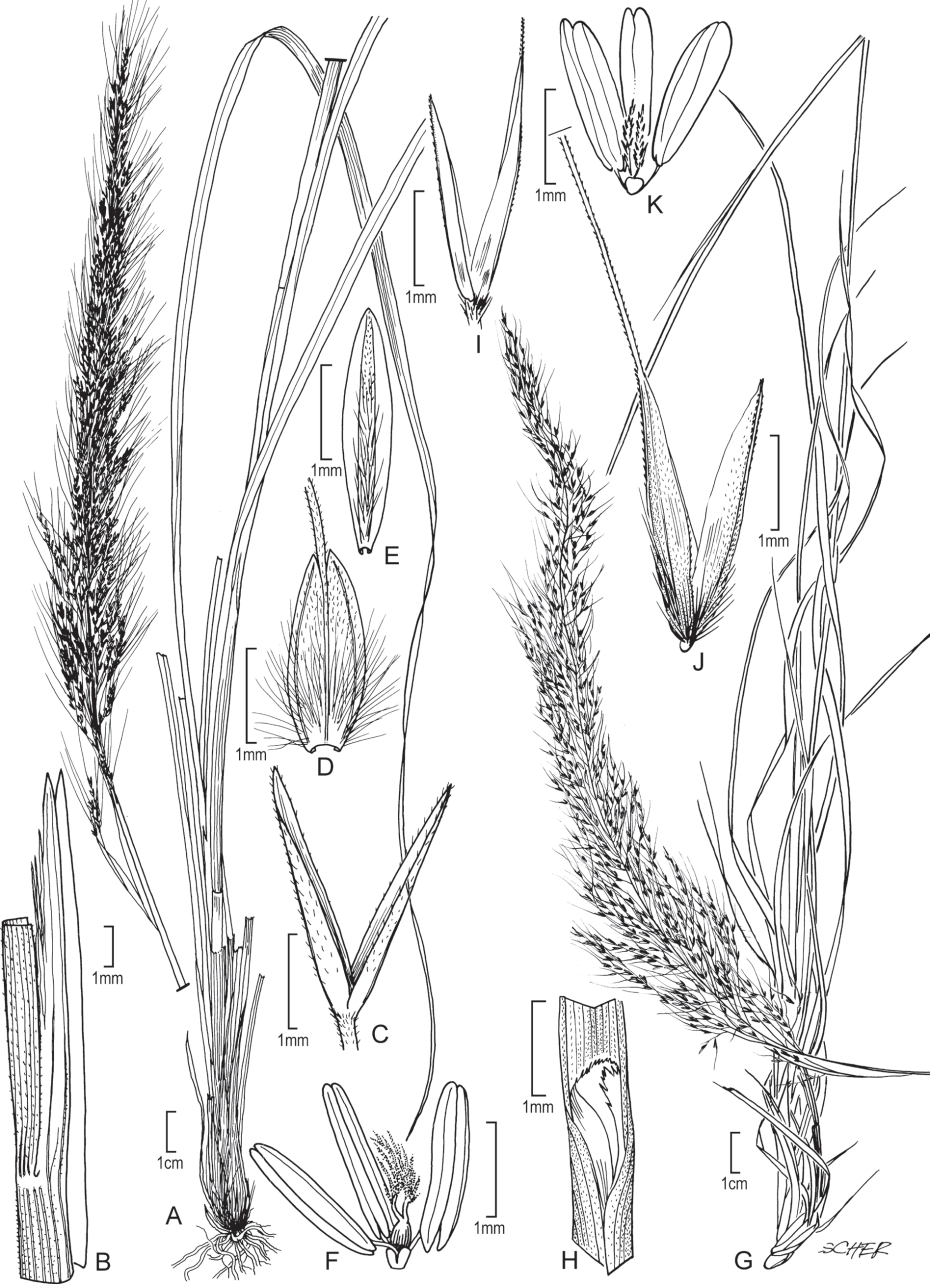
**A–F***Muhlenbergiaversicolor* Swallen **A** habit **B** ligule **C** glumes **D** lemma **E** palea **F** stamens, pistil, and lodicules **G–K***Muhlenbergialehmanniana* Henrard **G** habit, **H** ligule **I** glumes **J** floret **K** stamens, pistil, and lodicules. **A** drawn from *G.B. Hinton 2324* (US-1840807) **B–F** drawn from *G.B. Hinton 14808* (US-1842654) **G–K** drawn from *H. Pittier 1468* (US).

##### Distribution.

*Muhlenbergialehmanniana* ranges from Central America in Costa Rica, Honduras, and Panama to South America in Columbia, Ecuador, and Venezuela (Giraldo-Cañas and Peterson 2009).

##### Ecology.

This species occurs on gentle to steep slopes, ravines, gravelly sites, and disturbed sites in humid tropical forests with *Pinus* and *Quercus*; 600–2200 m.

##### Comments.

*Muhlenbergialehmanniana* is morphologically similar to *M.máxima* Lægaard & Sánchez Vega, a Peruvian endemic. However, *M.lehmanniana* differs from *M.máxima* in having longer lemmatal awns (12–20 mm long versus 4–9 mm long in *M.máxima*) and longer ligules (10–25 mm long versus 3–5 mm long in *M.máxima*) [Peterson et al. 2018].

*Muhlenbergialehmanniana* is a member of M.subg.Trichochloa (Peterson et al. 2010b; 2021). There is not much genetic variation among all members of M.subg.Trichochloa in Peterson et al. (2018, 2021).

##### Specimens examined.

Costa Rica. Basamento de Fila Lleskila, *L.D. Gómez et al. 23244* (MO). **Alajuela**: San Ramón, San Pedro de San Ramón, cerca del Río Barranca, *A.M. Brenes 21415* (CR); 6 km S of Los Cartagos by road, *R.W. Pohl & G. Davidse 11504* (US); 12 km SW of San Ramon along the Carretera Interamericana, *R.W. Pohl & G. Davidse 11511* (US). **Cartago**: El Guarco, *F. Solís 618* (CR); Oreamuno, in Monte Irazú, *A.S. Oersted 14012* (US), *14471* (US); Paraíso, Las Concavas, *C.H. Lankester 676* (US); 5 km E of Paraíso, *R.W. Pohl & G. Davidse 11390* (US). **Puntarenas**: Coto Brus, Z.P. Las Tablas. Cuenca Terraba-Sierpa, el tajo, colectando a orillas del camino, *E. Alfaro 2593* (MO); Coto Brus, Z.P. Las Tablas, Cuenca Térraba-Sierpe, Cerro Pando, colecta en bosque y orillas de potreros *E. Alfaro et al. 925* (MO); Coto Brus, Z.P. Las Tablas. Cuenca Térraba-Sierpe, Cerro Pando, colecta en bosque y orillas de potreros, *E. Alfaro et al. 931* (MO); Coto Brus, Z.P. Las Tablas, *E. Alfaro 925* (INB); Coto Brus, Z.P. Las Tablas, *E. Alfaro 931* (INB); Coto Brus, Finca Cafrosa, 2.5 km al E del Progreso, Cerro Pelón, *E. Alfaro 2593* (INB); Buenos Aires, Ujarras, El Carmen, Sabanas de Murur Bisuk, estribaciones de Cerro Amu, *G. Herrera 3593* (INB); Buenos Aires, P.N. La Amistad, Cuenca térraba-Sierpe, Violey, Sabanas Esperanzas, *L. González & A. Garita 1196* (MO); Buenos Aires, R.I. Ujarrás-Salitre-Cabagra, Cuenca Térraba-Sierpa, Salitre, Cerro Sipar, *L. González & A. Garita 1215* (MO); Monteverde, 10 km S Monteverde on road to InterAmerican Highway, area of spring, *W. Haber & W. Zuchowski 9660* (MO); Golfito, P.N. Corcovado, Península de Osa, Dos Brazos de Río Tigre, Jiménez, Cuenca superior del Río Madrigal, margen derecha, *G. Herrera & C. Fallas 4710* (MO, INB); Buenos Aires, Ujarrás, El Carmen, Sabanas de Murur Bisuk, estribaciones de Cerro Amú, *G. Herrera & W. Gamboa 3593* (MO); Buenos Aires, Violey, Sabanas Esperanzas, *L. González 1196* (INB); Buenos Aires, Salitre, Cerro Sipar, *L. González 1215* (INB); Parrita, Cuenca del Naranjo y Paquita, Fila Chonta, La Virgen, sector SE, Fila entre la *vuelta del Pallo*, cabeceras Río Palo Seco y Fila Chonta, *J. F. Morales & R.J. Abarca 6294* (MO); Buenos Aires, Potrero Grande, *A. Rodríguez 9744* (INB); Buenos Aires, Potrero Grande, *A. Rodríguez 9835* (INB); Boruca, Savanes de Boruca, *Soc. ex. San José 449* (CR); Buenos Aires, Potrero Grande, *D. Solano 2871* (INB); Buenos Aires, *M. Valerio 873* (CR); Monteverde, 10 km S Monteverde on road to Inter American Highway, area of spring, *W. Haber 9960* (INB); Coto Brus, Savanas de Cañas Gordas, *H. Pittier 7355* (US); Coto Brus, Savanes de Cañas Gordas, *H. Pittier 11019* (MO); Coto Brus, Savanas de Cañas Gordas, *H. Pittier 7358* (US); Borde de chemin de Mano de Tigre, *H. Pittier 4630* (US); **San José**: Perez Zeledón, along Carretera Interamericana, S slope Cerro de la Muerte, between km 125 and km 117, *M. Grayum & B. Hammel 9580* (MO, INB); Aserri, bosque primario y tacotales en la Fila El Alto, *J.F. Morales 3390* (INB); Aserri, Faldas SE Fila Aguabuena, entre Quebrada Ceniza y Quebrada Sopapo, camino a Bijagual, *J.F. Morales 6863* (INB, CR); Acosta, Alto Reflis, Falda NE, Fila de Cal, *J.F. Morales 8764* (INB); Escazú, San Antonio, *J. González 2886* (INB); Aserri, Cuenca del Pirris-Damas, Cerros Caraigres, Falda S, Quebrada Concha, en el camino viejo a Bijagual, *J.F. Morales 5915* (MO); Aserri, Cuenca del Pirris-Damas. Ceiba Alta, Cuest Pacayas, Quebrada Pacayas, charrales residuales, *J.F*. *Morales 6759* (MO, INB); 6 km by road N of San Pablo, N of San Marcos, open dry pasture on a hilltop, *R.W. Pohl & M. Lucas 13142* (MO); Acosta, Cerro León, camino hacia Fila Aguabuena, *R. Chacón et al. 190* (CR); Santa Ana, Z.P. Cerros de Escazú, Alto Caña Quemada, *A. Estrada et al. 3241* (CR); Cercanías de División, Carretera Interamericana Sur, *J. Gómez-L. 3460* (USJ); Acosta, Cuenca del Río Pírris-Damas, Fila del Naranjal, sendero a la Escuadra, *J.F. Morales 7459* (CR, INB, MO); Acosta, Cerro León. Camino hacia Fila Aguabuena, *A. Quesada et al. 741* (CR); Acosta, Cerro León. Camino hacia Fila Aguabuena, *J. Sánchez et al. 1170* (CR); 3 km SSE of Villa Colon, *R.W. Pohl & G. Davidse 11399* (US); 13 km N of San Isidro de El General along the Carretera Interamericana, *R.W. Pohl & G. Davidse 11631* (US). Honduras. **Comayagua**: Ojo de Agua, orilla Río Humuya, 30 km N de ciudad Comayagua, bosque de vega tropical rodeado de pinares, *C. Nelson et al. 6818* (MO). Panama. **Chiriquí**: In bare clay of steep artificial roadside bank below coffee finca. Western slope of first ridge east of Quebrada Zumbona, opposite east side of Cerro Pando, 7 km (by air) northwest of El Hato Del Volcán, *T.S. Cochrane et al. 6327* (MO); Boquete, ca. 26 km N of David along the road to Boquete, Curatella-Byrsonima-Trachypogon savanna on old lava flow, *G. Davidse & W.G. D’Arcy 10138* (MO); Boquete, eastern slope of Volcán de Chiriquí (Barú), WNW of Boquete. Partially cleared slopes with patches of original oak forest and mostly secondary growth, *G. Davidse & W.G. D’Arcy 10170* (MO); Large old lava flow ca. 3 km NE of El Hato del Volcán at base of Volcán de Chiriquí (Barú), 1–3 km E of highway, *G. Davidse & W.G. D’Arcy 10332* (MO); Llanos E of El Hato de Volcán, savannah and woods on lava flow, *B. Hammel et al. 6811* (MO); Grassy slopes on lava flow about 16 km above town at Volcan, *B. Hammel 1585* (MO); Volcán Barú, summit to llanos at base of W slope, along trail, *B. Hammel et al. 6576* (MO); Lava fields near the town of Volcan, *J.A. Duke 9143* (MO); Western slopes of Volcan de Chiriqui (Baru), on lava flow, *S. Mori & J. Kallunki 5714* (MO); Bambito, in savanna 1 mile south, *M. Partch 69-31* (MO); Alto Boquete, savanna, *M. Partch 69-62* (MO); 2 km S of Boquete above the Río Caldera near a small flood control dam. Disturbed roadside, *P.M. Peterson & C.R. Annable 7349* (MO, US); Between Río Quebrado El Velo and Río Caldera, W of San Ramon and NW of Boquete, gravel pit with very steep sandy slopes, *P.M. Peterson & C.R. Annable 7372* (MO, US); 1 km NW of Boquete on road towards Volcan Baru, dry roadcut in bare soil, *P.M. Peterson & C.R. Annable 7376* (MO, US); S end of Boquete, dry open slopes, *P.M. Peterson & C.R. Annable 7385* (MO, US); NW of Boquete, between Finca Lerida and San Ramon, *P.M. Peterson & C.R. Annable 7371* (MO, US); S end of Boquete, *P.M. Peterson & C.R. Annable 7386* (MO, US); Ca 1.5 mi. northeast of El Hato del Volcan, grassy plain with occasional patches of forest, *S. McDaniel 10199* (MO); Foothills, vicinity of El Boquete, *A.S. Hitchcock 8241* (US); foothills, vicinity of El Boquete, *A.S. Hitchcock*, *8242* (US); Cerro Vaca, eastern Chiriqui, in savannas, *H. Pittier 5360* (US); Cerro Vaca, eastern Chiriqui, in savannas, *H. Pittier 5362* (US). **Cocle**: hills S of El Valle de Antón, *P.H. Allen 2812* (MO);vicinity of Ola, *H. Pittier 5062* (US), *H. Pittier 5042* (US). **Veraguas**: Cerro Campana, savannas S of radio tower, *B. Hammel 5528* (MO).

#### ﻿12. Muhlenbergia ligularis

Taxon classificationPlantaePoalesPoaceae

(Hack.) Hitchc., Contr. U.S. Natl. Herb. 24(8):388. 1927.

31A51EDE-7EA1-568E-8C8E-20312CD46CA7

Fig. 11F–J

Sporobolus ligularis Hack., Oesterr. Bot. Z. 52(2):57. 1902. Type: Ecuador, Pichincha, 23 Jan 1899, *Sodiro 23/1* (holotype: W-19160026304 [image!]; isotypes: BAA-2905! ex W, US-3274313! ex W, US-1163183!). Basionym.
= Muhlenbergia calcicola Swallen, Contr. U.S. Natl. Herb. 29(9):407. 1950. Type: Guatemala, Huehuetenango, Chemal, Sierra de los Chuchumatanes, 3300 m, 31 Dec 1940, *P.C. Standley 81703* (holotype: US-1910686!; isotypes: F-1200274 [image!], US-2236500US!).
= Muhlenbergia breviculmis Swallen, Contr. U.S. Natl. Herb. 29(9):408. 1950. Type: Guatemala, Huehuetenango, Cerro Chemalito, Sierra de los Cuchumatanes, 3.5 mi W of Santa Eulalia, 3100–3150 m, 2 Aug 1942, *J.A. Steyermark 49905* (holotype: US-1935054!; isotypes: F, US-2208654!).
= Muhlenbergia minuscula H. Scholz, Willdenowia 14:393. 1984. Type: Bolivia, Canton Ulla-Ulla, Pampa von Ulla-Ulla, Apolobamba Cordillera, 4450 m, 26 Feb 1983, *X. Menhofer X-1974* (holotype: B-10-0249104!; isotype: LPB-0000293 [image!]).

##### Description.

Loosely tufted ***annuals*** to short-lived ***perennials*. *Culms*** 2–12 cm tall, 0.2–0.4 mm diameter just below the panicle, erect or decumbent, slender, glabrous, sometimes flowering the first year, up to 15 cm broad, dying in the center, profusely branched below, a short branchlet with fascicled leaves borne at each node, with 4–6 nodes; ***internodes*** 2–20 mm long. ***Leaf sheaths*** 2–20 mm long, generally shorter than the internodes, glabrous, ridged, flattened by the densely fascicled branches; ***ligules*** 0.6–2.5 mm long, membranous to hyaline, apex truncate to rounded; ***blades*** 0.3–2.2 cm long, 0.8–1.5 mm wide, flat or folded, prominently veined, thick, firm, usually with whitish-thickened midvein and margins, conspicuously crystalline or spiculate on both surfaces, otherwise glabrous below, sparsely scaberulous above and along margins, tapering to a boat shaped tip. ***Panicles*** 1.0–3.0 cm long, 0.3–1.4 cm wide, long exerted or included in the uppermost sheath, loosely contracted; ***primary branches*** 5–9 mm long, one per node, appressed or reflexed at maturity up to 70° from the culm axis; ***pedicels*** 1–3 mm long, stiff, densely scabrous, spiculate, erect. ***Spikelets*** 1.5–3.0 mm long, often plumbeous to reddish-purple; ***glumes*** 1.0–1.9 mm long, subequal, glabrous, apex acute to obtuse, often minutely erose, greenish-gray; ***lower glumes*** 1.0–1.7 mm long, 1-veined; ***upper glumes*** 1.1–1.9 mm long, 1-veined or occasionally 3-veined; ***lemmas*** 1.5–3.0 mm long, lanceolate, 3-veined, keeled, glabrous, mottled with greenish-black areas or dark greenish mottles on a pale background, apex minutely scaberulous, acuminate, entire or mucronate; mucro rarely more than 1(–1.2) mm long; ***paleas*** 1.4–2.9 mm long, lanceolate, glabrous; ***anthers*** 0.8–1.1 mm long, purplish becoming pale. ***Caryopses*** 0.8–1.2 mm long, elliptic to fusiform, brownish.

**Figure 11.**
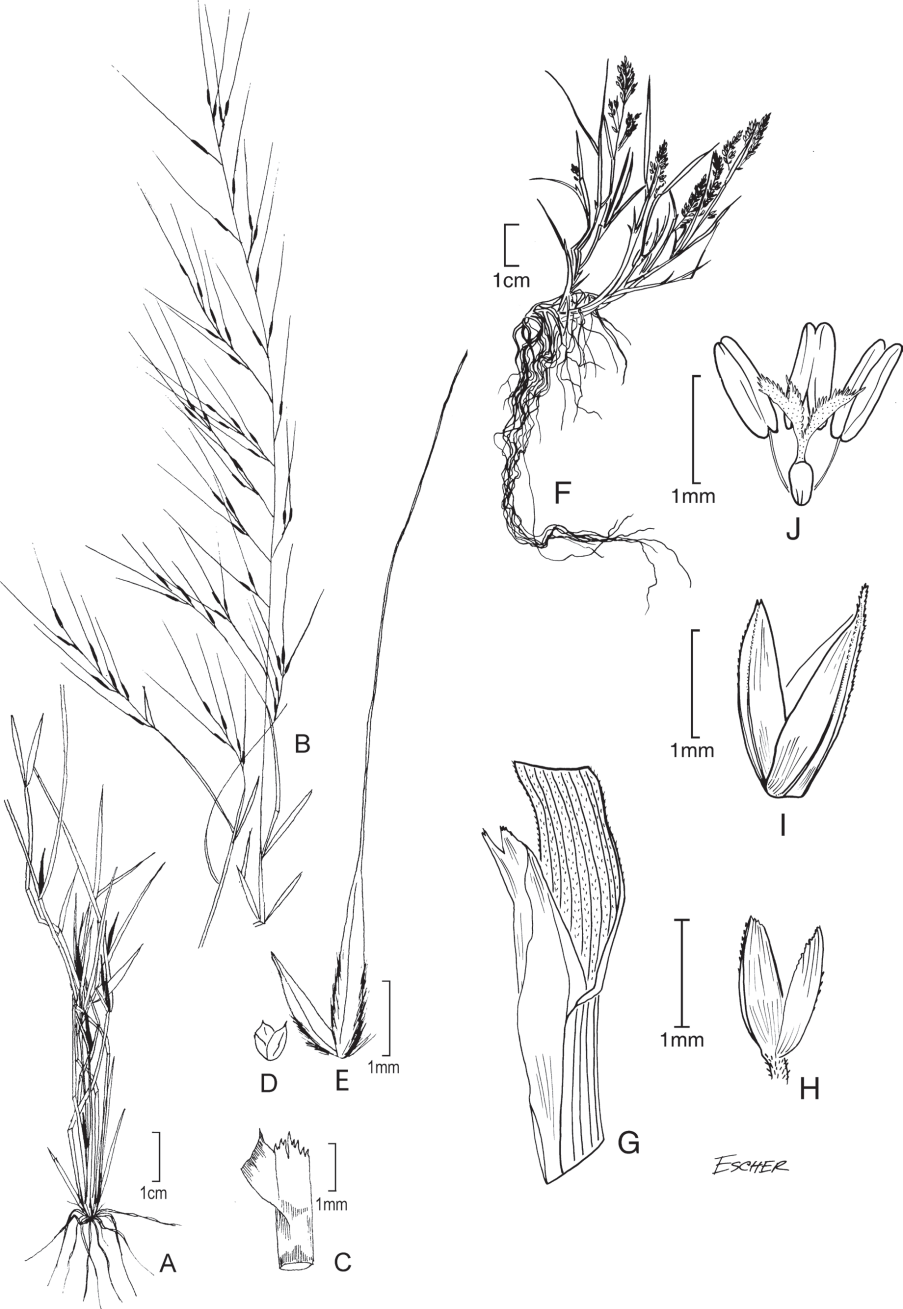
**A–E***Muhlenbergiamicrosperma* (DC.) Kunth **A** habit **B** inflorescence **C** ligule **D** glumes **E** floret **F–J***Muhlenbergialigularis* (Hack.) Hitchc. **F** habit **G** ligule **H** glumes **I** floret **J** stamens and pistil. **A–E** drawn from *P.M. Peterson 4185* (US, WS) **F–J** drawn from *P.M. Peterson*, *C.R. Annable*, *S. Lægaard & R.J. Soreng 12684* (US-3275569).

##### Distribution.

This species ranges from Guatemala and Costa Rica to Colombia, Venezuela, Ecuador, Bolivia, Peru, and Argentina (Pohl 1980; Peterson and Annable 1991; Peterson et al. 2001; Giraldo-Cañas and Peterson 2009).

##### Ecology.

*Muhlenbergialigularis* occurs in grassy flats, moist depressions, wet meadows, gravelly banks, ridgetops, and gravelly roadsides often derived from calcareous substrates, associated with *Achnatherum*, *Aciachne*, *Agrostis*, *Alnus*, *Anatherostipa*, *Baccharis*, *Berberis*, *Bidens*, *Buddleja*, *Caiophora*, *Carex*, *Cenchrusclandestinus* (Hochst. ex Chiov.) Morrone, *Colletiaspinosissima* J.F. Gmel., *Eleocharis*, *Festuca*, *Gaultheria*, *Hypericum*, *Jarava*, *Juncus*, *Lepidophyllum*, *Lupinus*, *Margyricarpus*, *Muhlenbergia*, *Nassella*, *Plantago*, *Poa*, *Puya*, *Rumex*, *Salvia*, and *Senecio*; 2320–4650 m.

##### Comments.

*Muhlenbergialigularis* is morphologically similar to the widespread South American, *M.fastigiata* (J. Presl) Henrard. It can be separated from the latter by possessing flat leaf blades, 0.8–1.5 mm wide, and a rather loosely tufted habit without wiry creeping rootstocks and scaly rhizomes. Morphologically, *M.ligularis* differs from the Peruvian endemic *M.caxamarcensis* Lægaard & Sánchez Vega in having glabrous lemmas (sericeous hairs on lower 1/2–3/4 of the lemma in the latter) [Peterson et al. 2018].

Molecular DNA sequence analysis indicates *M.ligularis* falls within the M.subg.Bealia clade in a subclade with *M.filiformis* (Thurb. ex S. Wats.) Rydb. and *M.vaginata* (Fig. 1; Peterson et al. 2021).

##### Specimens examined.

Costa Rica. **San José**: Valle de los Conejos (upper Río Talari) and trails to Cerro Chirripó and the Valle de los Lagos, open paramo formation with stands of *Chusquea* bamboo 1–2.5 m tall on slopes and in the valley, short grasses and very short (30 cm), shrubs on the exposed ridges, *W.C. Burger & R.L. Liesner 7470* (CR); Perez Zeledón, Paramo en el Sendero al Valle de los Conejos, *E. Alfaro 415* (INB, MO); Perez Zeledon, Rivas, sendero Valle Los Conejos y Cerro Chirripo en Paramo, *E. Alfaro 572* (INB); Perez Zeledón, Valle de los Conejos, *E. Alfaro 3873* (INB); P. N. Chirripó Alrededores del refugio, parte inferior (sur) del Valle de los Conejos, *J. Gómez 5344* (CR); P.N. Chirripó A la vera del Río Talari, parte inferior (Sur) del Valle de los Conejos, *J. Gómez 5382* (CR); P.N. Chirripó Valle de los Conejos, *R. Ocampo 1500* (CR); Pérez Zeledón, Rivas, *A. Rodríguez 6414* (INB); P.N. Chirripó Valle de los Conejos, *R. Soto s.n.* (CR). Guatemala. **Huehuetenango**: along road in region of Chémal, Sierra de los Cuchumatanes, at km 36, *P.C. Standley 81703* (MO); Sierra de los Cuchumatanes, 6.6 mi NW of Santa Eulalia on road to San Mateo Ixtatán, *P.M. Peterson & C.R. Annable 4691* (ARIZ, ENCB, GH. MEXU, MICH, MO, NMC, NY, RSA, TAES, UC, UNLV, US, UTC, WIS, WS); 3.6 mi NW of Paguix on hwy 9N and 16.2 mi S of San Juan Ixcoy, *P.M. Peterson & C.R. Annable 4686* (GH, MO, NY, US, WS); 15.1 mi S of San Juan Ixcoy on hwy. 9N, *Peterson & Annable 4688* (GH, MO, NY, RSA, US, WS); Meadow at Tojiah on hwy. 9N, *P.M. Peterson & C.R. Annable 4695* (GH, MO, NY, RSA, UC, US, WS); 13 mi NW of Santa Eulalia on road to San Mateo Ixtatán, *P.M. Peterson & C.R. Annable 4692* (ARIZ, ENCB, GH, MEXU, MICH, MO, NMC, NY, RSA, TAES, UC, UNLV, US, UTC, WIS, WS); Meseta alta Sierra de los Cuchumatanes, *R. López s.n.*, (MO); Cerro Chemalito, Sierra de Cuchumatanes, 3.5 miles W of Santa Eulalia, *J.A. Steyermark 49905* (MO); Sierra de los Cuchumatanes, at Chemal at km 318 on Ruta Nacional 9N, *J.H. Beaman 3068* (US); Sierra de los Cuchumatanes, immediately north of Tojiah at km 322 on Ruta Nacional 9N, *J.H. Beaman 3920* (US); Region of Chemal, Sierra de los Cuchumatanes, *P.C. Standley 81115* (US); Sierra de los Cuchumatanes, 3.6 mi NW of Paguix on hwy 9N and 16.2 mi S of San Juán Ixcoy, *P.M. Peterson & C.R. Annable 4686* (GH, MO, NY, RSA, US, WS); 15.1 mi S of San Juan Ixcoy on hwy 9N, *P.M. Peterson & C.R. Annable 4688* (GH, MO, NY, RSA, US, WS); 6.6 mi NW of Santa Eulalia on road to San Mateo Ixtatán, *P.M. Peterson & C.R. Annable 4691* (ARIZ, ENCB, GH, MEXU, MICH, MO, NMC, NY, RSA, TAES, UC, UNLV, US, UTC, WIS, WS); 13 mi NW of Santa Eulalia on road to San Mateo Ixtatán, *P.M. Peterson & C.R. Annable 4692* (ARIZ, ENCB, GH, MEXU, MICH, MO, NMC, NY, RSA, TAES, UC, UNLV, US, UTC, WIS, WS); Meadow at Tojiah on hwy 9N, *P.M. Peterson & C.R. Annable 4695* (GH, MO, NY, RSA, UC, US, WS); SW of Tojiah on Hwy 9N, *P.M. Peterson & C.R. Annable 4700* (NY, US, WS). **Chimaltenango**: Cerro Chichoy near Chichoy, *L.O. Williams & A. Molina R.15317* (US). **Totonicapan**: On the Tecum Uman Ridge at km 154 on Ruta Nacional N1, ca 20 km east of Totonicapan, *J.H. Beaman 4156* (UC, US); Desconsuelo, potrero natural, Flora alpine, *M. de Koninck 116* (US); Region of Desconsuelo, *P.C. Standley 62736* (US); Region of Chiu Jolom, mountains above Totonicapan, on road to Desconsuelo, *P.C. Standley 84418* (US); Totonicapan, En pastizal dominado por *Agrostisexserta* y *Geraniumalpicola*, muy sobrepastoreado; plano; plena sol, *Smith & Nelson 768* (MO).

#### ﻿13. Muhlenbergia macroura

Taxon classificationPlantaePoalesPoaceae

(Kunth) Hitchc., N. Amer. Fl. 17(6): 468. 1935.

DF61A073-D4DE-5529-B22A-71FBEFAC2281

Fig. 7A–E

Crypsis macroura Kunth, Nov. Gen. Sp. (quarto ed.) 1: 140–141. 1816 Type: México. Toluca, in apricis montanis regio Mexicane, 1760 m, *F.W.H.A. Humboldt & A.J.A. Bonpland s.n*. (holotype: P-00669403[image!]; isotypes: BAA-00003297 [image!], P-00077291 [image!]). ≡ Cinnamacroura (Kunth) Kunth, Révis. Gramin. 1: 67. 1829. ≡ Phleummacrourum (Kunth) Willd. ex Steud., Nomencl. Bot. (ed. 2) 1: 365. 1840. ≡ Epicampesmacroura (Kunth) Benth., J. Linn. Soc., Bot. 19: 87. 1881. ≡ Crypsinnamacroura (Kunth) E. Fourn., Mexic. Pl. 2: 90. 1886. Basionym.

##### Description.

Caespitose ***perennials*. *Culms*** 75–200 cm tall, erect, terete near base, forming dense clumps of 100 culms or more and up to 1 m in diameter, pubescent below the nodes, usually 1 or 2 nodes per culm; ***internodes*** mostly glabrous. ***Leaf sheaths*** 15–40 cm long, shorter than the internodes, glabrous to scaberulous, the basal persistent and keeled with age; ***ligules*** (5–)8–40(–50) mm long, strongly decurrent, splitting into broad **auricles** 10–35(–50) mm long, membranous to chartaceous above, brownish, firm, the veins evident below and near margins, apex truncate to obtuse; ***blades*** 20–60 cm long, 2–5 mm wide, mostly flat and apically involute, scabrous above and below. ***Panicles*** (15–)20–40 cm long, 5–12 mm wide, dense, spikelike, erect, exserted and surpassing the blades in height, greenish to greenish-gray; ***primary branches*** 0.1–1.2 cm long, ascending and tightly appressed, unexposed, imbricate; ***pedicels*** 0.1–1.7 mm long, shorter than the spikelets, scaberulous to hispidulous. ***Spikelets*** 3.4–5.6(–6) mm long, erect, strongly laterally compressed, greenish-gray; ***glumes*** 3.4–5.6 mm long, linear-elliptic to linear-ovate, usually longer than the lemma, 1-veined, scabrous along the keel, subequal, awnless, the upper slightly longer, apex acute to acuminate, scabrous; ***lemmas*** 3.4–5 mm long, elliptic to linear-elliptic, scabrous, greenish-gray; callus pilose, the hairs 0.1–0.3 mm long, apex acute, rarely mucronate, the mucro less than 0.4 mm long; ***paleas*** 3.4–5 mm long, about as long as the lemma, scabrous, apex acute; ***anthers*** 1.5–2.2 mm long, pale greenish. ***Caryopses*** 2–3 mm long, fusiform, brownish. 2*n* = 20, 24, 28.

##### Distribution.

*Muhlenbergiamacroura* occurs in the Sierra Madre Occidental in northern México from Chihuahua to Chiapas and Guatemala (Herrera Arrieta and Peterson 2018).

##### Ecology.

This species can be found growing on upland slopes, mountain meadows, in pine or pine-oak forests often in deep humid soils; 1500–3400 m.

##### Comments.

*Muhlenbergiamacroura* is morphologically similar to *M.nigra* but differs in having spikelets 3.4–5.6(–6) mm long [(5.5–)6–8 mm long in *M.nigra*] and greenish to greenish-gray panicles (15–)20–40 cm long [dark green to blackish panicles 6–15(–17) cm long in *M.nigra*]. *Muhlenbergiamacroura* is a member of M.subg.Trichochloa, and in a recent study was found to be sister to *M.rigida* (Peterson et al. 2021).

##### Specimens examined.

Guatemala. **Chimaltenango**: Plains near Tecpam, *A.F. Skutch 610* (US). **Guatemala**: Guatemala City, *A.S. Hitchcock 9140* (US). **Huehuetenango**: La Sierra (Tujimach), across river from San Juan Atitlan, Sierra de los Cuchumatanes, *J.A. Steyermark 51968* (US); Paquix, San Juan Ixcoy, Sierra de los Cuchumatanes between Paquix and San Juan Ixcoy, *A.A. Molina et al. 30017* (MO); San Juan Ixcoy, Sierra de los Cuchumatanes, along road to Huehuetenango, 5 miles S of San Juan Ixcoy, *D.E. Breedlove 8569* (MO, DS); San Juan Ixcoy, Jolomhuitz, Aldea Jolomhuitz, *M. Véliz 95.4470* (MO). **Quetzaltenango**: Mountains near Santa Maria, near Quetzaltenango, *Weatherwax 177* (US); La Esperanza, *M. de Koninck 18* (US); Cuesta El Caracol, Sierra Madre Mountains, about 5–8 km N of San Juan Ostuncalco, *L.O. Williams et al. 22770* (US); Oak pine forest, above Los Vahos, Cerro Quemado, *P.C. Standley 86171* (US). **Quiche**: Chichicastenango, 1 km N of Chichicastenango, *W.E. Harmon 4369* (MO). **Sacatepequez**: Santa Maria de Jesús, Mal Paso, Volcán de Agua, Mal Paso, *M. Véliz et al. 8529* (MO); Volcan Agua, *W.A. Kellerman 4764* (US). **San Marcos**: Tajumulco, Volcan Tajumulco, *M. Véliz et al. 10622* (MO), *M. Véliz et al. 10638* (MO); El Boqueron, in the mountains at the summit of the road between San Antonio Sacatepequez and Palestina, *P.C. Standley 85307* (US). **Solola**: Volcan Atitlan, near summit of mountain, *J. H. Beaman 4053* (US); NW of Los Encuentros, *L.O. Williams*, *A. Molina R. & T.P. Williams 25404* (F, US). **Totonicapán**: near San Francisco El Alto, *P.C. Standley 83123* (US); along road between San Francisco El Alto and Momostenango, *P.C. Standley 84011* (US); *O. F. Cook 35* (US); Hochland von Calel, *s.n. 2703* (US). Mexico. **Chiapas**: Vol. Tacana, Chiquihuite, *E. Matuda 2829* (ARIZ, MO); Mt. Tacana, *E. Matuda 2420* (DS). Amecatlán, Paraje Navenchauk, along mexican Hwy 190, *R. M. Laughin 1506* (ENCB). **La Grandeza**: La Grandeza to Ojo de Agua, *E. Hernandez X-1445* (US). **Larráinzar**: Muctahuitz. Región Los Altos, *L. Soto s/n* (MEXU). **Motozintla**: On the N and W slope of Cerro Mozotal below the microwave tower along the road from Huixtla to El Porvenir and Siltepec, *D.E. Breedlove & R.F. Thorne 31173* (MO). **San Juan Chamula**: near school house of yal Ichin, *D.E. Breedlove 7147* (ENCB, DS); Yut Bax, *C. Santoz Ruíz 179* (ENCB). Motozintla, NW slope of cerro Mozotal, along the road from Huixtla to El Porvenir and Siltepec, *D.E. Breedlove 31175* (NY); Slope near the school house of Yal Ichin, *D.E. Breedlove 10471* (DS). **Tenajapa**: Paraje Matsbad, *Alush Shilon Ton 999*, (ENCB, DS); Paraje Shohieh, *Alush Shilon Ton 548* (ENCB, DS); near Tenejapa Center, *Alush Shilon Ton 63* (DS). **Venustiano Carranza**: Ejido “Laja Tendida” km 17 carr. Venustiano Carranza-Tuxtla Gutiérrez, aprox. 2 km a Flores Magón, *A. Miranda S. s/n* (MEXU). **Zinacantán**: near Paraje Nachij [Nachig], *D.E. Breedlove & G. Davidse 53871* (CAS, MO); Steep NE slope of Zontehuitz near Summit, *D.E. Breedlove 12348* (DS); near Zinacantán Center, *R.M. Laughlin 2241* (DS); at paraje Navenchauk along Mexican Highway 190, *R.M. Laughlin 1506* (DS); alley floor in Zinacantán Center, *R.M. Laughlin 630* (DS).

#### ﻿14. Muhlenbergia microsperma

Taxon classificationPlantaePoalesPoaceae

(DC.) Kunth, Révis. Gramin. 1:64. 1829.

1FAEADD4-C2C6-5DB5-9923-8AACC85102A7

Fig. 11A–E

Trichochloa microsperma DC., Cat. Pl. Horti Monsp. 151. 1813. Type: México, cultivated at botanical garden at Montpellier from seeds collected in México and distributed by the Botanical Garden of Madrid, M. Sésse & J.M. Mociño s.n. (holotype: MPU; isotypes: G-00099434 [image!], P!, US fragm. ex P!). ≡ Muhlenbergiamicrosperma (DC.) Trin., Gram. Unifl. Sesquifl. 193. 1824, *nom. inval*. Basionym.
= Agrostis microsperma Lag., Gen. Sp. Pl. 2. 1816. Type: México, plants grown at H.R. Matritensis (= Herbario del Real Jardín Botánico de Madrid) from seeds collected by M. Sessé & J.M. Mociño in Nueva Espania, Oct, 1806, M. Sessé & J.M. Mociño s.n. (**lectotype, designated here**: SEL-H10620 [image!]).
= Podosemum debile Kunth, Nov. Gen. Sp. (quarto ed.) 1: 128. 1816. Type: Ecuador, Prov. Pichincha, Quito, *F.W.H.A. Humboldt & A.J.A. Bonpland s.n.* (holotype: P-Bonpl!; isotypes: B-W, P!, US-91924 fragm. ex P-Bonpl!). ≡ Trichochloadebilis (Kunth) Roem. & Schult., Syst. Veg. 2:385. 1817. ≡ Muhlenbergiadebilis (Kunth) Trin., Gram. Unifl. Sesquifl. 193, t. 5, f. 18. 1824.
= Podosemum setosum Kunth, Nov. Gen. Sp. (quarto ed.) 1:129. 1816. Type: México, between Gueguetoque and Tula, Aug, *F.W.H.A. Humboldt & A.J.A. Bonpland 4174* (holotype: P-Bonpl!; isotypes: B-W, US-91917 fragm. ex P-Bonpl!). ≡ Trichochloasetosa (Kunth) Roem. & Schult., Syst. Veg. 2:386. 1817. ≡ Agrostissetosa (Kunth) Spreng., Syst. Veg. 1:262. 1825. ≡ Muhlenbergiasetosa (Kunth) Trin., Gram. Unifl. Sesquifl. 193, t. 5, f. 22. 1824. ≡ Muhlenbergiasetosa (Kunth) Kunth, Révis. Gramin. 1:63. 1829, *isonym*.
= Muhlenbergia purpurea Nutt., J. Acad. Nat. Sci. Philadelphia, ser. 2, 1:186. 1848. Type: USA, California, Santa Barbara Co., Santa Barbara and Santa Catalina Island, *Gambel s.n.* (holotype: K!).
= Muhlenbergia ramosissima Vasey, Bull. Torrey Bot. Club 13(12):231. 1886. Type: México, Chihuahua, SW Chihuahua, Aug–Nov 1885, *E. Palmer 158* (lectotype: NY! designated by Hitchcock, N. Amer. Fl. 27:441. 1935, but without indicating the specific specimen; Peterson and Annable, Syst. Bot. Monogr. 31:61. 1991, indicated the specific specimen; isotypes: LE!, MO-2974152!, P!, US-995580!).

##### Description.

Caespitose ***annuals***, sometimes appearing as short-lived perennials. ***Culms*** 10–80 cm tall, often geniculate at the base, slender, often striate, much branched near the base, scaberulous below the nodes; ***internodes*** 1.8–8.6 mm long, mostly scaberulous or smooth. ***Leaf sheaths*** 2.2–6.6 mm long, commonly shorter than the internodes, glabrous, smooth or scaberulous; ***ligules*** 1–2 mm long, membranous to hyaline, decurrent, margins often extended, apex truncate to obtuse; ***blades*** 3–8.5(–10) cm long, 1–2.5 mm wide, flat or loosely involute, scabrous below, strigulose above, often deciduous with age. ***Panicles*** 6.5–13.5 cm long, 1–6.5 cm wide, open and not densely flowered, often purplish; ***primary branches*** 1.6–4 cm long, ascending or diverging up to 80° from the rachises, spikelet-bearing to the base; ***pedicels*** 2–6 mm long, appressed to divaricate, antrorsely scabrous. ***Cleistogamous panicles*** with 1–3 spikelets present in the axils of the lower sheaths. ***Spikelets*** 2.5–5.3 mm long; ***glumes*** 0.4–1.3 mm long, exceeded by the florets, 1-veined, obtuse, often minutely erose; ***lower glumes*** 0.4–1 mm long; ***upper glumes*** 0.6–1.3 mm long; ***lemmas*** 2.5–3.8(–5.3) mm long, narrowly lanceolate, mostly smooth, scaberulous distally, hairy on the lower 1/2 of the margins and midveins, the hairs 0.2–0.5 mm long, the callus hairy, apices acuminate, often bidentate, awned, awns 10–30 mm long, straight to flexuous; ***paleas*** 2.2–4.8 mm long, narrowly lanceolate, acuminate; ***anthers*** 0.3–1.2 mm long, purplish. ***Caryopses*** 1.7–2.5 mm long, fusiform, reddish-brown. 2*n* = 20, 40, 60.

##### Distribution.

*Muhlenbergiamicrosperma* occurs in Hawaii, southwestern USA, México, Guatemala, Colombia, Venezuela, Ecuador (including the Galapagos Islands), Peru, and Bolivia (Peterson and Annable 1991).

##### Ecology.

Rocky slopes, rock outcrops, sandy drainages, cliffs, and disturbed roadsides usually in desert scrub vegetation with *Acacia*, *Aristidaadscensionis* L., *Baccharis*, Bombacaceae, Cactaceae, *Dodonaeaviscosa* (L.) Jacq., *Fucraea*, *Heliotropium*, *Heteropogoncontortus* (L.) P. Beauv. ex Roem. & Schult., *Lantana*, *Pitcairnia*, *Prosopis*, *Puya*, *Salvia*, *Schinusmolle* L., and *Schizachyrium*; 1150–3500 m.

##### Comments.

*Muhlenbergiamicrosperma* can sometimes be confused with *M.romaschenkoi* known only from Peru and differs from it by having cleistogamous panicles in the axils of the lower sheaths and shorter, obtuse glumes, 0.4–1.3 mm long (glumes acute to acuminate, 2–2.8 mm long in *M.romaschenkoi*) [Peterson et al. 2018].

In a molecular DNA sequence study, *M.microsperma* forms a strongly supported clade with two other annuals, *M.appressa* C.O. Goodd. and *M.brandegei* C.G. Reeder, all members of M.subg.Muhlenbergia (Fig. 1; Peterson et al. 2010b). In addition, these three species produce cleistogamous spikelets in the axils of the lower culm branches, enclosed by a sheath (Peterson and Annable 1991). Cleistogamous spikelets appear to have evolved twice within *Muhlenbergia*, once in M.subg.Muhlenbergia within the *M.appressa–M.brandegei–M.microsperma* clade and once in M.sect.Pseudosporobolus in *M.cuspidata* (Torr. ex Hook.) Rydb. (Morden and Hatch 1984; Peterson et al. 2010b).

##### Specimens examined.

Guatemala. **Sacatepequez**: Above Pastores, wet thicket, *P.C. Standley 60819* (US); Volcano Agua, near Antigua, shady bank, *A.S. Hitchcock 9130* (US); Antigua, *W.A. Kellerman 7301* (US).

#### ﻿15. Muhlenbergia minutissima

Taxon classificationPlantaePoalesPoaceae

(Steud.) Swallen, Contr. U.S. Natl. Herb. 29(4):207. 1947.

C7010EF7-A52C-531A-92C7-B2A4057794A8

Fig. 12E–H

Agrostis minutissima Steud., Syn. PI. Glumac. 1:171. 1854 Type: U.S.A., New México, 1847, *A. Fendler 986* (holotype: not located; isotypes: F-72158 [image!], MO!, NY-327637!, S-29780 [image!], US-825378!, US-997292!, W-0030511 [image!]). ≡ Sporobolusminutissimus (Steud.) Hitchc, Proc. Biol. Soc. Wash. 41:161. 1928. Basionym.
= Milium microspermum Lag., Gen. Sp. PI. 2. 1816, Type:: México, Habitat in Nova Hispania, *D. Sesse s.n*. (holotype: MA; isotype: US-91019! fragm.). non Muhlenbergiamicrosperma (DC.) Trin. 1824. ≡ Panicummicrospermum (Lag.) E. Fourn., Mexic. Pl 2: 492. 1886. ≡ Sporobolusmicrospermus (Lag.) Hitchc, J. Wash. Acad. Sci. 23(10):453. 1933.
= Vilfa confusa E. Fourn., Mexic. PL 2: 101. 1886. Type: México, Jalicingo, *C.].W. Schiede & Deppe 913* (syntype: US-998282! fragm. ex P); Orizaba, *Botteri 117* (syntypes: P!, US! fragm.); Orizaba, *Schaffner 93* (syntypes: P!, US! fragm. ex P); Orizaba, *Schajfner 125* (syntype: P!); Nevado de Toluca, Sep, *Hahn s.n.* (syntype:: P!); U.S.A., *Hall & Harbour 643* (syntype: P!). ≡ Sporobolusconfusus (E. Fourn.) Vasey, Bull. Torrey Bot. Club 15:293. 1888. ≡ Muhlenbergiaconfusa (E. Fourn.) Swallen, Contr. U.S. Natl. Herb. 29(4): 207. 1947.

##### Description.

Delicate ***annuals*. *Culms*** 5–40 cm tall, erect or spreading, slender, scaberulous to strigulose below the nodes, 0.3–0.7 mm diameter just below the inflorescence; ***internodes*** 8–25 mm long. ***Leaf sheaths*** 4–52 mm long, shorter to longer than the internodes, glabrous or scaberulous, margins hyaline; ***ligules*** 1–2.6 mm long, hyaline, apex irregularly toothed to lacerate, truncate to obtuse, margins entire, sometimes splitting off to form auricles which are not longer than the body of the ligule; ***blades*** 0.5–4.0(–10) cm long, 0.8–2.0 mm wide, flat or involute, short pubescent above and scabrous below. ***Panicles*** 5.0–16.2(–21) cm long, 1.5–6.5 cm wide, open, ovate, nodes 12–35 per inflorescence; ***primary branches*** 8–42 mm long, one or two per node, spreading 25–80° from the rachis; ***pedicels*** 2–7 mm long, slender often capillary, erect. ***Spikelets*** 0.8–1.5 mm long, erect; ***glumes*** 0.5–0.9 mm long, subequal, 1-veined, sparsely short-pilose at least near apex; ***lower glumes*** 0.5–0.8 mm long, obtuse to acute; ***upper glumes*** 0.6–0.9 mm long, obtuse, usually broader than the first glume; ***lemmas*** 0.8–1.5 mm long, lanceolate, with short appressed silky pubescence located along the midvein and margins to glabrous, unawned, apex obtuse to subacute; ***paleas*** 0.8–1.4 mm long, about as long as lemma, with short appressed silky pubescence between the veins or glabrous; ***anthers*** 0.2–0.7 mm long, purplish. ***Caryopses*** 0.6–0.9 mm long, fusiform to elliptic, brownish. 2*n* = 60, 80.

**Figure 12.**
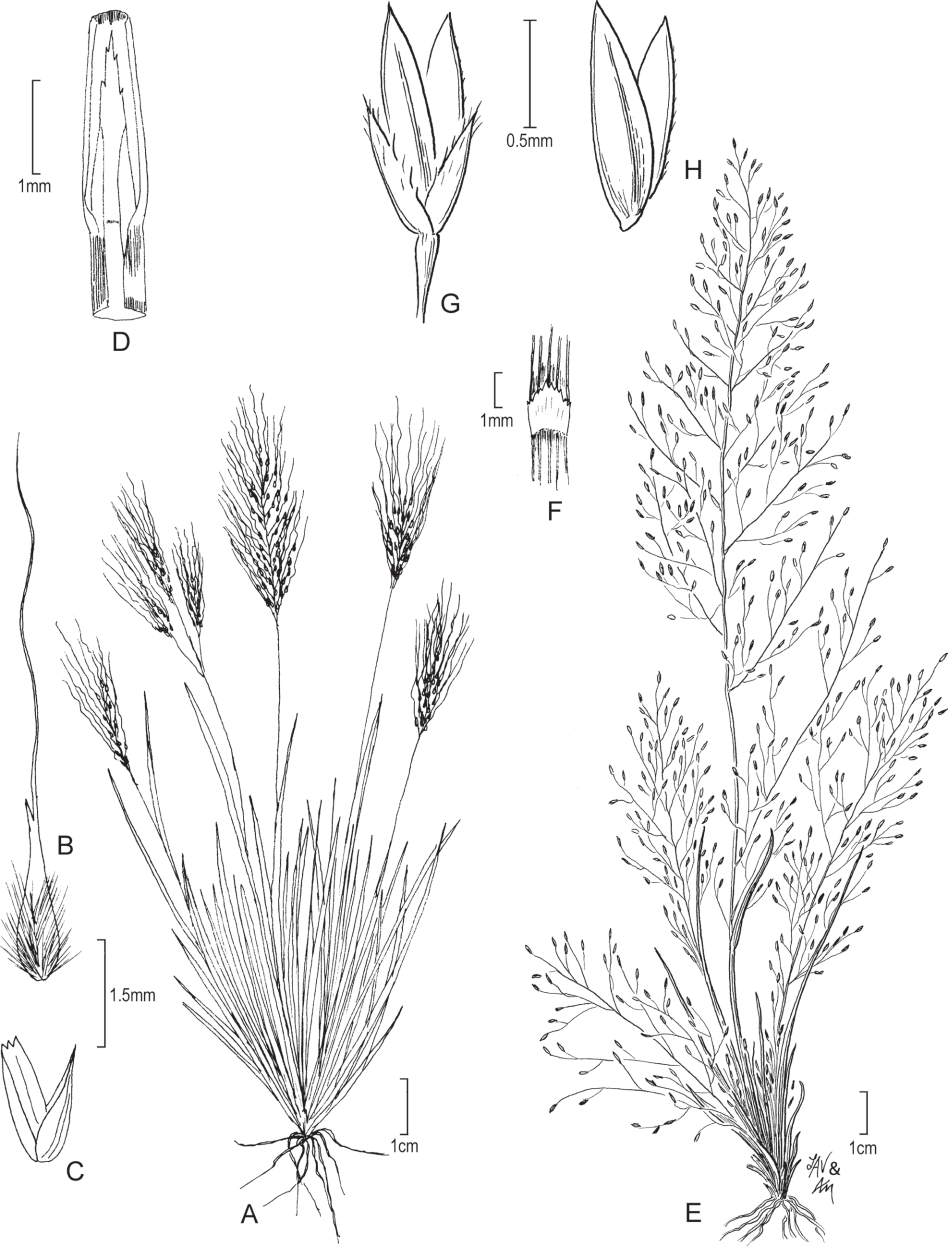
**A–D***Muhlenbergiaperuviana* (P. Beauv.) Steud. **A** habit **B** floret **C** glumes **D** ligule **E–H***Muhlenbergiaminutissima* (Steud.) Swallen **E** habit **F** ligule **G** spikelet **H** floret. **A–D** drawn from *P.M. Peterson & C.R. Annable 4067* (US, WS) **E, G, H** drawn from *P.M. Peterson & C.R. Annable 5601* (US-3182908) **F** drawn from *P.M. Peterson & C.R. Annable 4675* (US, WS).

##### Distribution.

Western North America from central Washington to Montana south to Texas, U.S.A. and throughout México to Guatemala (Peterson and Annable 1991).

##### Ecology.

Sandy and gravelly drainages, rocky slopes, flats, road cuts, and open sites most commonly in yellow pine forests, oak-pine forests with *Arctostaphylos*, thorn scrub forests with *Acacia*, pinyon-juniper woodlands, and oak-gramma grass (*Bouteloua*) savannahs; 1200–3000 m.

##### Comments.

Morphologically, *Muhlenbergiaminutissima* can be separated from other annual species of *Muhlenbergia* in Central America in having sparsely short-pilose glumes near the apex and open, ovate, panicles with pedicels that are longer than the spikelets (2–7 mm long [Peterson and Annable 1991].

Based on DNA sequence analysis, *Muhlenbergiaminutissima* is a member of M.subg.Bealia and shares a most recent common ancestor with *M.sinuosa* Swallen, a species known to occur in Arizona and New Mexico, USA and Chihuahua and Sonora, México (Peterson and Annable 1991; Peterson et al. 2021).

##### Specimens examined.

Guatemala. **Quetzaltenango**: Santa Maria, Volcano Agua, *A.S. Hitchcock 9130* (MICH, US-998503). **Quiché**: *W.A. Archer 3858* (US-1646054). **Totonicapán**: E of San Cristóbal Totonicapán, *Harmon 4570* (ENCB, NY).

#### ﻿16. Muhlenbergia montana

Taxon classificationPlantaePoalesPoaceae

(Nutt.) Hitchc., U.S.D.A. Bull. (1915–23) 772: 145, 147. 1920.

F34E17C0-C68D-506A-957D-DE1ACBE999C2

Fig. 9F–J

Calycodon montanum Nutt., J. Acad. Nat. Sci. Philadelphia ser. 2. 1:186. 1848. Type:United States, New Mexico, Santa Fe Co., in the Rocky Mountains near Santa Fe, *W. Gambel s.n.* (holotype: BM!; isotypes: GH, MO-992590!, PH). Basionym.
= Muhlenbergia gracilis var. enervis Scribn. ex Beal, Grass. N. Amer. 2: 242. 1896. Type: México, Chihuahua, dry ledges, Sierra Madre, 7 Oct 1887, *C.G. Pringle 1413* (holotype: MSC; isotypes: GH-00024024 [image!], US-995814!, UVMVT-024031 [image!], W-1916-27712!). ≡ Muhlenbergiaenervis (Scribn. ex Beal) Hitchc., Contr. U.S. Natl. Herb. 17(3): 302. 1913.
= Muhlenbergia trifida Hack., Repert. Spec. Nov. Regni Veg. 8: 518. 1910. Type: México, Michoacán, vicinity of Morelia, Quinceo, 9 Nov 1909, *Bro. Arséne 3217* (holotype: W-1916-32145!; isotypes: BM, MO-843315!, MPU-026951 [image!], US!, US-86637! fragm.).

##### Description.

Densely caespitose ***perennial*. *Culms*** 10–80(–90) cm tall, erect, terete near base, glabrous below the strictly basal nodes; ***internodes*** mostly glabrous, occasionally glaucous. ***Leaf sheaths*** 2–35 cm long, longer than the lower internode, glabrous to scaberulous, often glaucous, becoming flat, loose and papery, and occasionally spirally twisted near the base; ***ligules*** 4–14(–20) mm long, membranous, decurrent, apex acute to acuminate, often lacerate; ***blades*** 6–30 cm long, 1–2.5(–3) mm wide, flat becoming loosely involute to subfiliform, somewhat stiff, scabrous below and hirsute above. ***Panicles*** 5–25 cm long, (1–)2–6 cm wide, narrow to somewhat open, loosely flowered, not dense; ***primary branches*** 0.5–10 cm long, ascending, appressed or spreading to 40° from the rachises; ***pedicels*** 2–7 mm long, longer than the spikelets, flattened, scabrous, occasionally stiffly reflexed. ***Spikelets*** 3–4.5(–7) mm long, erect, occasionally reflexed; ***glumes*** (1–)1.5–3.2(–4) mm long, ⅓ to ⅔ as long as the lemma, subequal, glabrous to scaberulous above; ***lower glumes*** 1-veined, sometimes mucronate, the mucro less tan 1 mm long; ***upper glumes*** 3-veined, 3-toothed and 3-awned, the teeth (including the awns) ⅓ to the length of the glume, and the awns up to 1.7 mm long, apex truncate to acute; ***lemmas*** 3–4.5 mm long, lanceolate, awned, often greenish or yellowish with dark green or purple mottles, scaberulous above, loosely to densely appressed-pubescent to pilose along the midvein, margins, and proximal. to ⅘, the hairs up to 0.8 mm long, occasionally glabrous, apex acute to acuminate, the awn (2–)6–25 mm long, flexuous; ***paleas*** 3–4.5 mm long, lanceolate, loosely to densely appressed-pubescent to pilose between the veins on the proximal ⅓ to ⅘, apex acute to acuminate, scaberulous; ***anthers*** 1.5–2.3 mm long, purplish. ***Caryopses*** 1.8–2 mm long, fusiform, light brown. 2*n* = 20, 40 (Herrera Arrieta 1998).

##### Distribution.

This species ranges from southwestern USA throughout western México to Guatemala (Peterson et al. 2001).

##### Ecology.

*Muhlenbergiamontana* grows on rocky slopes, dry meadows, ridgetops, and open grasslands, primarily in upland and mountain habitats in pine and oak forests, at elevations of 1400–3000 m.

##### Comments.

*Muhlenbergiamontana* is morphologically similar to *M.quadridentata* but can be separated from the latter in having 3-toothed and 3-awned upper glumes with teeth 1/3 to ½ the length of the glume (the teeth are small <1/6 the length of the glumes in *M.quadridentata*), lemmas that are greenish or yellowish with green mottles or purple mottles (the lemmas are greenish-plumbeous to mottled plumbeous in *M.quadridentata*), and the anthers are usually 1.5–2 mm long (2–2.5 mm long in *M.quadridentata*) [Herrera Arrieta and Peterson 2007, 2018].

*Muhlenbergiamontana* is a member of Muhlenbergiasubg.Clomena, a lineage hypothesized to have originated in the Sierra Madre of México about 5.4 mya (Peterson et al. 2021).

##### Specimens examined.

Guatemala. **Huehuetenango**: La Sierra (Tujimach), across river from San Juan Atitlan, Sierra de los Cuchumatanes. Open upper slopes, *J.A. Steyermark 51989* (US); About Laguna de Ocubila, east of Huehuetenango, dry open oak woods, *P.C. Standley 82722* (US); Chiantla, cerca del cementerio, Llano de San Nicolás, *D.N. Smith 442* (MO)¸ Aldea San Nicolás, Chiantla, *D.N. Smith 491* (MO). **Quetzaltenango**: La Esperanza, lugares secos, esteriles, *M. de Koninck 132* (US). **Quiche**: Between Quiche and San Pedro Jocopilas, on dry rolling hills with pine and oak forest, *P.C. Standley 62453* (US). Mexico. **Chiapas**: Amatenango del Valle: S of the center of Amatenango del Valle, *Alush Shilom Ton 1531*, (ENCB). **San Cristóbalde las Casas**: “El Banco” sobre el libramiento E a San Cristobal, km 4 carr. San Cristobal de Las Casas-Tenejapa, *A. Miranda S. s/n* (MEXU); *D.E. Breedlove & G. Davidse 55174* (CAS) cited in Reeder (1994).

#### ﻿17. Muhlenbergia mucronata

Taxon classificationPlantaePoalesPoaceae

(Kunth) Trin., Gram. Unifl. Sesquifl. 194, t. 5, f. 23. 1824.

C2D1B2C3-EF7F-525F-A86D-0D75064371C1

Fig. 13H

Podosemum mucronatum Kunth, Nov. Gen. Sp. 1:129. 1815 (1816). Type: México, Guanajuato, crescit in mountains prope Cerro de Serna, Santa Rosa et Los Ioares, 1270–1360 hexap (2318-2482 m). Sep, *F.W.H.A. Humboldt & A.J.A. Bonpland s.n.* (holotype: P!; isotypes: BAA-00003945 [image!], BM-000938659 [image!], US-91925! fragm. ex P-Bonpl. y photo). ≡ Agrostismucronata (Kunth) Spreng., Syst. Veg. 1:262. 1825 ≡ Trichochloamucronata (Kunth) Roem. & Schult., Syst. Veg. 2:387. 1817. Basionym.
= Muhlenbergia laxiflora Scribn., Zoë 4:389. 1894. Type: México, Baja California Sur, La Chuparrosa, 17 Oct 1893, *T.S. Brandegee 74* (lectotype designated by A.S. Hitchcock, Contr. U.S. Natl. Herb. 17: 298. 1913: UC-122474!; isolectotypes: NY-00381444 [image!], US! fragm.ex UC).

##### Description.

Densely caespitose ***perennials*. *Culms*** 75–100(–120) cm tall, erect, scabrous or strigulous below the nodes, rounded near base. ***Leaf sheaths*** shorter than internodes, glabrous or scaberulous, purplish in part, rounded below; ***ligules*** (2–)5–8 mm long; ***blades*** 20–40 cm long, 2–3 mm wide, flat or loosely involute, scabrous abaxially, scabrous on ribs adaxially, attenuate into a long apex. ***Panicles*** (5–)10–15(–20) cm long, 1–3(–6) cm wide, oblong-cylindrical, spreading, purple, rarely purplish-green; ***primary branches*** 4–8(–10) cm long, ascending or appressed; ***pedicels*** 1–3 mm long, usually shorter than the spikelets, slender, scabrous below spikelets. ***Spikelets*** (4–)4.5–5 mm long; ***glumes*** 1.5–2 mm long, acute, subequal, scabrous near the apex; ***lemmas*** (4–)4.5–5 mm long, scaberulous between veins, mucronate or shortly awned from 2 minute teeth, the mucro or awn 0.5–1(–2) mm long; ***callus*** hairy, the hairs 0.5–0.7 mm long; ***paleas*** about as long as the lemma, scabrous between the veins, apex acute or acuminate; ***anthers*** 2–2.2 mm long, purplish. ***Caryopses*** 2–2.5 mm long, about 0.5 mm wide, ellipsoid, reddish-brown.

**Figure 13.**
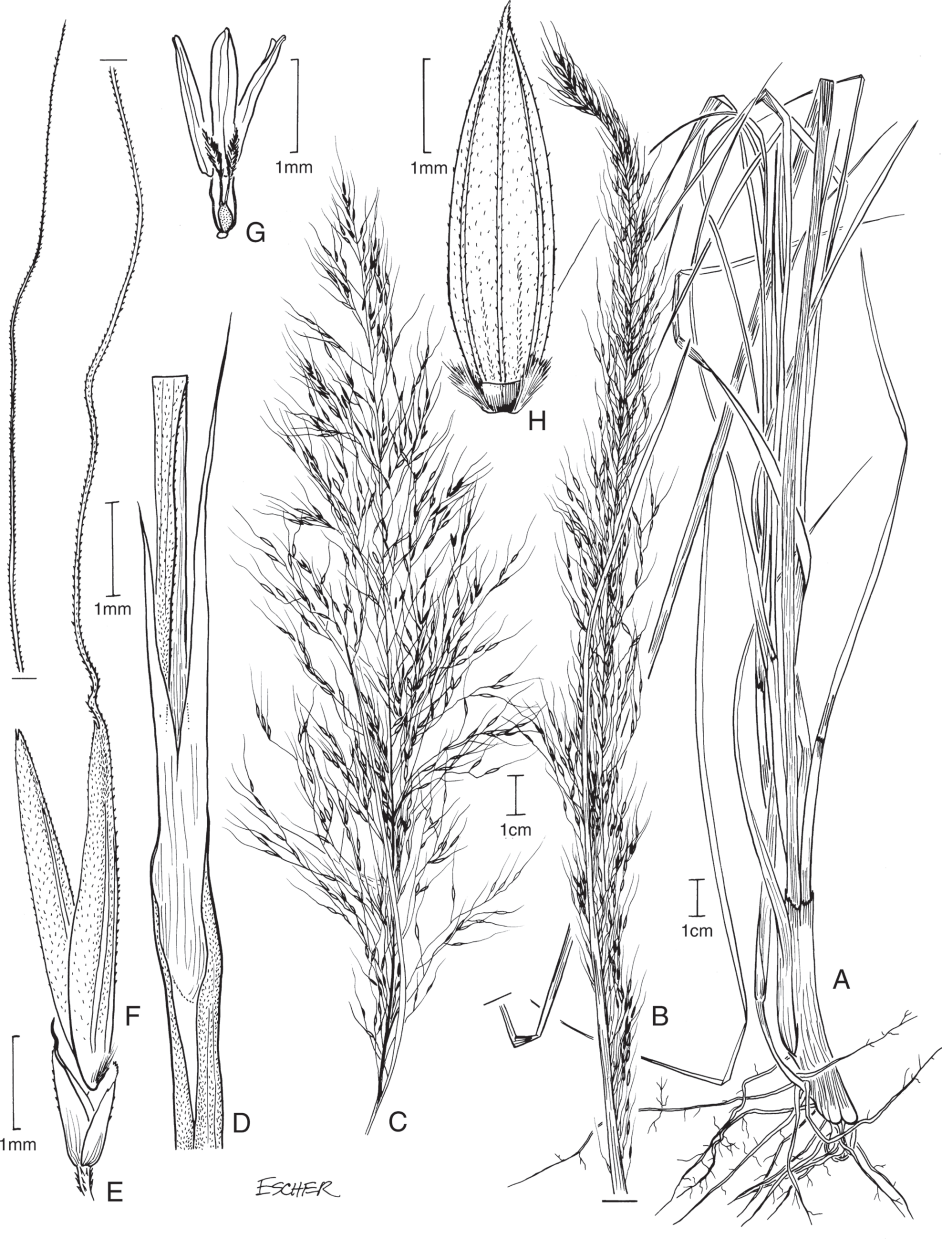
**A–G***Muhlenbergiarigida* (Kunth) Kunth **A** habit **B** inflorescence (narrow) **C** inflorescence (open) **D** ligule **E** glumes **F** floret **G** stamens and pistil **H***Muhlenbergiamucronata* (Kunth) Trin. **H** floret. **A, B** drawn from *P.M. Peterson 9659* (US) **C–I** drawn from *P.M. Peterson*, *C.R. Annable & J. Valdés-Reyna 10876* (ANSM, US) **H** drawn from *P.M. Peterson & C.R. Annable 10778* (US).

##### Distribution.

This Mexican endemic is known from: Baja California and Chihuahua in the north throughout central México to Oaxaca, Veracruz, and Chiapas in the south (Herrera Arrieta and Peterson 2018).

##### Ecology.

*Muhlenbergiamucronata* grows in oak–pine forests at elevations of 1350–2650 m.

##### Comments.

*Muhlenbergiamucronata* is morphologically similar to *M.rigida*, differing from the latter in having mucronate to short-awned lemmas, narrower panicles, and usually shorter pedicels.

*Muhlenbergiamucronata* is a member of M.subg.Trichochloa and pairs with *M.subaristata* Swallen in a recent biogeographical analysis based on plastid and nuclear DNA sequences (Fig. 1; Peterson et al. 2021).

##### Specimens examined.

Mexico. **Chiapas**: **San Cristóbal de las Casas**: 7 km E of San Cristobal las Casas in road to Zontehuitz, *D.E. Breedlove 11153* (US-3113121, US-3113122). **Tenejapa**: along river Chik Ha, barrio of Yashanal, *D.E. Breedlove 11125* (US-3113120). **Zinacantán**: along hwy 190 at Granadilla, *D.E. Breedlove 10607* (US-3113119), *D.E. Breedlove 52324* (CAS) citado en Flora Mesoamercana.

#### ﻿18. Muhlenbergia mutica

Taxon classificationPlantaePoalesPoaceae

(Rupr. ex E. Fourn.) Hitchc., N. Amer. Fl. 17(6): 459. 1935.

70301F49-10E2-5232-85AE-8BCFEF837069

Fig. 14A–I

Epicampes mutica Rupr. ex E. Fourn., Mexic. Pl. 2: 87. Type: México, Veracruz, Mirador, Zacuapan, and Cantaranas, 1840, *H.G. Galeotti 5797* (lectotype, designated by T.R. Soderstrom in Contr. U.S. Natl. Herb. 34(4): 141. 1967: P; isolectotype: US-865973! fragm. ex P). Basionym.
= Epicampes gigantea E. Fourn., Mexic. Pl. 2:88. 1886. Muhlenbergiagigantea (E. Fourn.) Hitchc., N. Amer. Fl. 17(6): 460. 1935. Type: México, Veracruz, Orizaba, Río Blanco, 30 Sep 1886, *E. Bourgeau 3137* (**lectotype, designated here**: P-02265396 [image!]; isolectotypes: G-00099412 [image!], MPU-027109 [image!], P-02265395 [image!]; S14-29388 [image!], US-865978 fragm!, US-865977 fragm!).
= Epicampes bourgeaei E. Fourn., Mexic. Pl. 2:88. 1886. Type: México, Veracruz, Escamala, Refrou D’Orizaba, 26 Aug 1866, *E. Bourgeau 2973* (holotype: P; isotypes: K!, US-A0865984! fragm.).
= Epicampes bourgeaei var. mutica E. Fourn., Mexic. Pl. 2:88. 1886. Type: México, Veracruz, Mirador, Nov 1841, F.M. Liebmann 678 (lectotype: US-207466!, designated by Herrera Arrieta and Peterson, Sida, Bot. Misc. 29: 35. 2007; isolectotypes: K!, US-207465!).
= Epicampes expansa E. Fourn., Mexic. Pl. 2:88. 1886. Type: México, Orizaba, M. Botteri & Sumichrast 104 (holotype: P!; isotype: US-865979! fragm).
= Epicampes laxiuscula E. Fourn., Mexic. Pl. 2:88. 1886. Type: México, Orizaba, M. Botteri 155 (syntype: P?; isosyntypes: BM-000938656 [image!], US-865975! fragm.).
= Epicampes ehrenbergii Mez, Repert. Spec. Nov. Regni Veg. 17: 212. 1921. Type: México, Cuesta de Pinolco, *C. Ehrenberg 1156* (holotype: B?; isotype: US-A0865980! fragm.).
= Muhlenbergia alta Hitchc., N. Amer. Fl. 17(6): 461. 1935. Type: México, Jalisco, hills E of Zapotlán, 25 Sep 1910, *A.S. Hitchcock 7180* (holotype: US-998980!).
= Muhlenbergia magna Hitchc., N. Amer. Fl. 17(6): 460. 1935. Type: México, Jalisco, under cool cliffs of barranca near Guadalajara, 3 Nov 1890, *C.G. Pringle 3335* (holotype: US-825277!; isotypes: BR-0000006883416 [image!], BR-0000006883744 [image!], CM-2820 [image!], E-00531666, F-73213 [image!], G-00099367 [image!], G-00099368 [image!], GOET-006639 [image!], KFTA-0000246 [image!], MEXU-00004527 [image!], MEXU-00004528 [image!], MO-105133!, MO-1837821!, MU-000000018 [image!], MU-000107304 [image!], US-998977!), W-18910001067 [image!].

##### Description.

Strongly caespitose ***perennials***. ***Culms*** 120–300 cm tall, erect, compressed-keeled near the base, glabrous below the nodes to sometimes scaberulous. ***Leaf sheaths*** 12–32 cm long, shorter than internodes, sometimes purplish near base, often changing to brown with age, keels prominent and glabrous, lacking auricles; ***ligules*** (5–)8–15 mm long, membranous, apex lacerate; ***blades*** 35–110 cm long, 5–10 mm wide, flattened, scaberulous adaxially and glabrous abaxially, margins and keels serrate. ***Panicles*** 35–100 long, (8–)15–30 cm wide, purple or brown-purplish, branches ascending or pendulous spreading up to 60° from culm axis; ***primary branches*** 6–25 cm long, usually 15–20 cm long below, naked near the base, pendulous to flexuous; ***pedicels*** 0.2– 2.5 mm long, generally shorter than spikelets, scaberulous. ***Spikelets*** 1.3–2.6(–3) mm long, erect, purple to brownish-purple; ***glumes*** 1.3–2.6(–3) mm long, ovate, generally longer than florets, subequal, 1-veined, often translucent, usually glabrous to scaberulous, apex acute to obtuse; ***lemmas*** 1.3–2.3 mm long, oblong, awnless, rarely mucronate, glabrous; ***paleas*** 1.3–2.3 mm long, glabrous, apex acute to obtuse; ***anthers*** 0.9–1.3 mm long, yellow to purplish. ***Caryopses*** 1–1.3 mm long, fusiform, reddish-brown. 2*n* = 20, 24.

**Figure 14.**
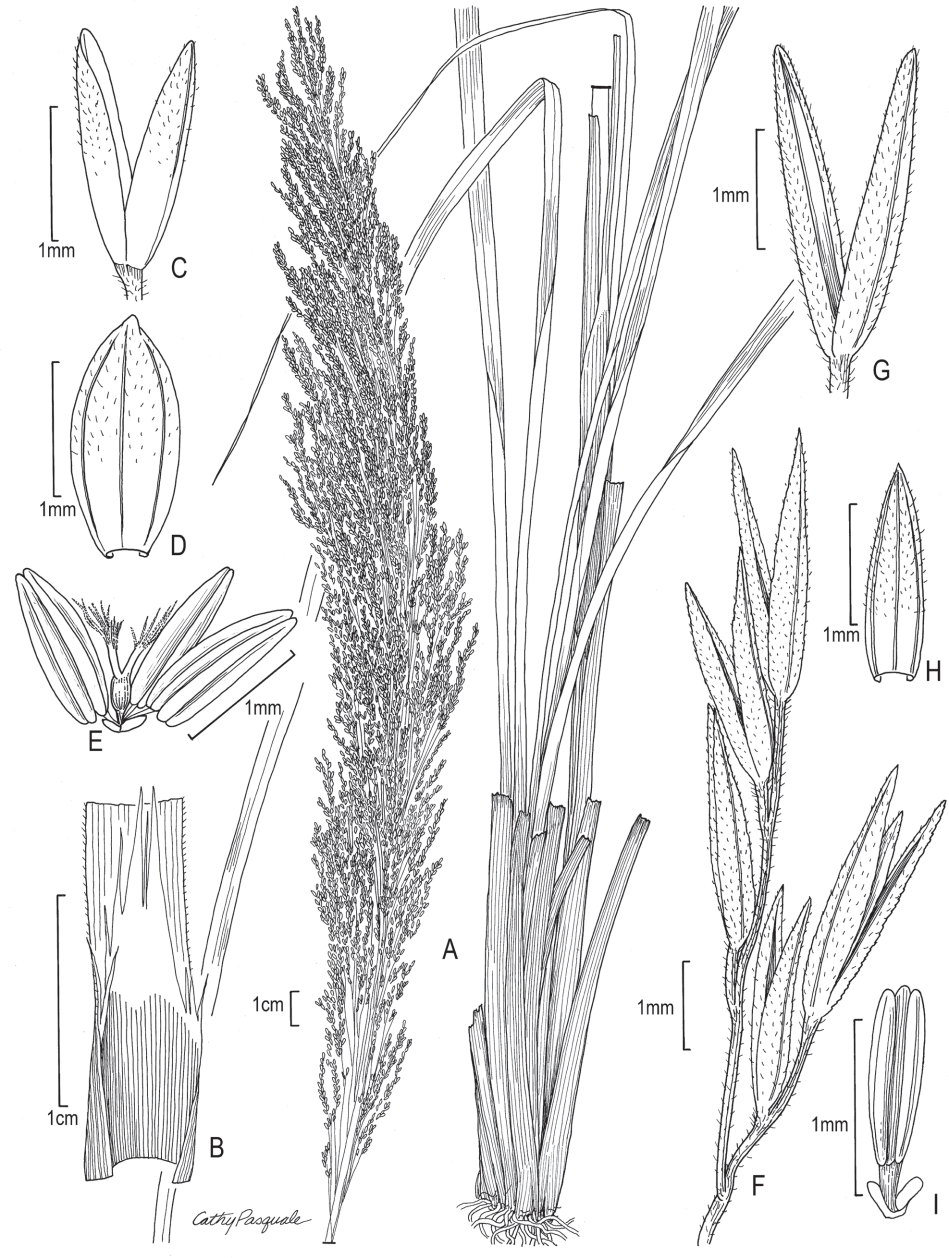
**A–I***Muhlenbergiamutica* (Rupr. ex E. Fourn.) Hitchc. **A** habit **B** ligule **C** glumes **D**. lemma **E** stamens, pistil, and lodicules **F** inflorescence branch **G** glumes **H** lemma **I** stamens and lodicules. **A–E** drawn from *P.M. Peterson & C.R. Annable 6051* (US) **F–I** drawn from *Carl Mez Herbarium 3370* [*Botteri & Sumichrast 104*] (US-1720166).

##### Distribution.

*Muhlenbergiamutica* ranges from Chihuahua, Sinaloa, and Durango, México south to Oaxaca, Veracruz, and Chiapas (Herrera Arrieta and Peterson 2007, 2018).

##### Ecology.

The species grows among pines or pine–oak forests and tropical forests with *Liquidambar*, *Nyssa*, and *Sabal*; 600–2300 m.

##### Comments.

*Muhlenbergiamutica* can be separated morphologically from *M.robusta* in having wider panicles (8–)15–30 cm wide (2–8 cm wide in *M.robusta*) and non-auriculate leaf sheaths [auricles 2–4(–10) mm long in *M.robusta*] (Herrera Arrieta and Peterson 2018). *Muhlenbergiamutica* is a member of M.subg.Trichochloa, and in a recent study was found sister to *M.virletii* (E. Fourn.) Soderstr., another Mexican endemic (Peterson et al. 2021).

Soderstrom (1967) chose to recognize *M.mutica* and *M.gigantea*, stating, “The only character to separate the two is the length of the glumes in relation to the floret. It (*M.mutica*) is most closely related to and doubtfully distinct from *M.gigantea*.” After careful study of the isolectotypes attributed to *M.mutica* and *M.gigantea* (both housed at US), we find no differences in glume length, and they appear to represent specimens of a single species. Therefore, we place *M.gigantea* (younger name) as a synonym of *M.mutica* in our treatment.

##### Specimens examined.

Mexico. **Chiapas. Cintalapa**: 12 km S of’ Mexican hwy 190 near Rizo de Oro, Crest of the Sierra near the microwave station of La mina, *D.E. Breedlove 20641* (ENCB); 23 km W of Las Cruces along road to La Mina Microwave Station, *D.E. Breedlove & G. Davidse 54111* (CAS, MO); Slope, near La Cienega de Leon 30 km N of Las Cruces, *D.E. Breedlove & F. Almeda 48041* (CAS, MO). **Ixtapa**: near Ixtapa, *D.E. Breedlove & G. Davidse 54301* (CAS, MO); Near the Zinacantán Paraje of Muctajoc, *D.E. Breedlove & G. Davidse 54007* (CAS, MO); at Escopetazo, *D.E. Breedlove & G. Davidse 53947* (CAS, MO). **Jitotol**: 10 km N of Jitotol, *D.E. Breedlove 55152* (NY); near Colonia El Laurel, ca. 5 km N of Jitotol, *G. Davidse et al. 29600* (MO). **La Trinitaria**: *E.M. Martínez S. & W.D. Stevens 23903* (MEXU, MO). Paraje of Mahben Chauk, *D.E. Breedlove 7677* (ENCB); Paraje of Yehts ‘Uk’um, *D.E. Breedlove 7504* (ENCB); in the Paraje of Mahosik’, *D.E. Breedlove 14861* (ENCB). **cozingo**: Estación Chajul, reserva Montes Azules, 4 km NE del poblado de Chajul, *S. Sinaca-Colín s/n* (MEXU). Tenejapa: In the Paraje of Mahosik’, *A.S. Ton 1187* (ENCB). Tenejapa: Kulaktik, *A.M. Ton 4570* (ENCB). **San Juan Cancuc**: El Pozo to Oxchuc, *E. Hernández & Sharp X-616* (US). **Tenejapa**: slopes, near Paraje Kulak’tik, *D.E. Breedlove 53057* (CAS, MO); lopes west of Tih Ha’ in the Barrio of Kurus Pilal. Paraje of Mahben Chauk, *D.E. Breedlove 6282* (DS). **Zinacantán**: from Zinacantán paraje of Paste’ to San Lucas, *R.M. Laughlin 2583* (ENCB); ear Paraje Sequentic, *D.E. Breedlove & G. Davidse 53910* (CAS, MO).

#### ﻿19. Muhlenbergia nigra

Taxon classificationPlantaePoalesPoaceae

Hitchc., N. Amer. Fl. 17(6): 468. 1935.

4F2B1408-F751-5166-B50B-31AD18815DDE

Fig. 3F–J

##### Type.

México, México, Nevado de Toluca, cool slopes under pines, 2 Sep 1892, *C.G. Pringle 4211* (holotype: US-746689!; isotypes, F!, MO-2974170!, MSC, US-821929!).

##### Description.

Caespitose ***perennials***. ***Culms*** 45–110 cm tall, erect, rounded near base, pubescent below the nodes, internodes glabrous to scaberulous. ***Leaf sheaths*** 3–17 cm long, shorter than the internodes, glabrous to scaberulous, the basal persistent, papery and flattened with age; ***ligules*** (5–)8–20 mm long, strongly decurrent, occasionally splitting into auricles, membranous to chartaceous above, firmer below near margins, apex truncate to obtuse; ***blades*** 5–25(–35) cm long, 2–3 mm wide, involute, scabrous and short pubescent above and mostly glabrous below. ***Panicles*** 6–15(–17) cm long, 5–10 mm wide, dense, spikelike, erect, exserted and surpassing the blades in height, plumbeous with a hint of green; ***primary branches*** 1–6 mm long, tightly appressed and ascending, imbricate, unexposed; ***pedicels*** 0.1–1.3 mm long, densely hispidulous. ***Spikelets*** (5.3–)6–8 mm long, erect, laterally compressed, plumbeous with a hint of green; ***glumes*** (5.3–)6–8 mm long, linear-elliptic to linear-ovate, usually longer than the lemma, 1-veined, sometimes mucronate or awn-tipped, scabrous along the keel, subequal; apex acuminate, scabrous, the awn up to 1.8 mm long; ***lemmas*** 5–6.5 mm long, broadly lanceolate, the margins involute, scabrous, unawned, mucronate or awned greenish-gray; apex acute, sometimes awn-tipped, the awn up to 1.3 mm long sometimes borne between two minute lobes, the lobes 0.1–0.3 mm long; ***callus*** sparingly pilose, the hairs 0.1–0.3 mm long; ***paleas*** 4.5–6 mm long, shorter than the lemma, scabrous; apex acute; ***anthers*** 2.5–3.2 mm long, grayish-green to whitish-gray. Caryopses 3–3.5 mm long, fusiform, brownish. Chromosome number unknown.

##### Distribution.

*Muhlenbergianigra* occurs in central México south to Guatemala and Costa Rica (Peterson et al. 2001).

##### Ecology.

This species is found in mountain meadows, lava fields, and open pine forests; 2300–4000m.

##### Comments.

*Muhlenbergiacoerulea* (Griseb.) Mez from the Cordillera de los Andes in South America is morphologically similar to *M.nigra*. However, in recent molecular studies *M.nigra* and *M.coerulea*, both members of M.subg.Trichochloa, do not share an immediate common ancestor but lie in a poorly resolved clade with 10 other species in the subgenus (Fig. 1; Peterson et al. 2018, 2021).

##### Specimens examined.

Costa Rica. **San José**: Sabana, south fork of Rio Talarí, *A.S. Weston 12372* (CR, ISC); Pérez Zeledón, sendero a la Sabana los Leones, 700m. S del Puesto, *A. Rodríguez 6461* (INB); Pérez Zeledón, P.N. Parque Nacional Chirripó, Cuenca Chirripó, Cuenca Terraba-Sierpe, sendero a Valle Los Leones, *E. Alfaro 1770* (INB, MO); Pérez Zeledón, Paramo en Las Sabanas Chirrido, *E. Alfaro 424* (INB, MO); Pérez Zeledón: bosques quemados y bosque enano camino al Rio Terbi y Sabana de Los Leones, *J.F. Morales 5174* (INB); Pérez Zeledón, Parque Nacional Chirripó. Del Puesto Los Crestones, 2.5 km al Sur, camino a Sabana Leones, *R. Robles 1784* (INB, MO); Rivas. P.N. Chirripó. Sabana de los Leones, *J. Sánchez el al. 2870* (MO); P.N. Chirripó, Valle de los Conejos, *R. Ocampo 1492* (CR); Parque Nacional Chirripó, Frente a refugio Los Crestones, *G. Vargas et al. 540* (USJ); P.N. Chirripó, Sabana de los Leones, *A. Weston 10114* (CR). Guatemala. **Huehuetenango**: Todos Santos, Cuchumatan, Tuicoy, *R. Flores & M. Véliz 94.4308* (MO); Todos Santos Cuchumatan, camino a la Torre, *M. Véliz et al. 14030* (MO); Sierra de los Cuchumatanes, ca. 28 mi from Huehuetenango, slopes of Cerro Chemal, *J.G. Hanokes 1453* (US); Cerro Chémal, *J.A. Steyermark 50305* (US-1935065, US-2208671). **Quetzaltenango**: Volcan Santa Maria, near summit of mtn., in open somewhat weedy and disturbed meadow above timberline, *J.H. Beaman 4119* (US). **Sacatepequez**: Volcano Agua, open pine woodland on steep northern slopes, *W.E. Harmon 3595* (MO); Santa Maria de Jesús, *M. Véliz et al. 2M.8512* (MO); Acatenango, Volcán de Acatenango, *M. Véliz et al. 10351* (MO); Volcan de Agua, summit of south rim of crater. In open, gravelly soil, *J.H. Beaman 2931* (US); Volcano Agua, *A.S. Hitchcock 9117* (US); Volcan de Agua, *W.A. Kellerman 7416* (US); Antigua, Volcan de Agua, *W.A. Kellerman s.n.* (US); Volcan de Agua, *G. Salas 538* (US).

#### ﻿20. Muhlenbergia orophila

Taxon classificationPlantaePoalesPoaceae

Swallen, Contr. U.S. Natl. Herb.29(9): 408. 1950.

F0FB03FF-2A43-5F77-8749-25ABE4CE788B

Fig. 15F–J

= Muhlenbergia matudae Sohns, J. Washington Acad. Sci. 46(12): 382, f. 32–38. 1956. Type: México, Morelos, Lago de Zempoala, collected en madera humeda, orilla de bosque mixto de pinos y oyamel, 3000 m, 7 Oct 1951, *E. Matuda 25601* (holotype: US-2079186!; isotype: US-2119930!).

##### Type.

Guatemala, Dept. Huehuetenango, Cerro Chémal in alpine meadow, summit of Sierra de los Cuchumantanes, 3700–3800 m, 8 Aug 1942, *J.A. Steyermark 50309* (holotype: F-1202399!; isotypes: F-73211!, G-00099366 [image!], US-132785!, US-132784!, US-1935066!, US-2208672!).

##### Description.

Densely caespitose ***perennials***. ***Culms*** 12–30 cm tall; ***leaf sheaths*** longer than the internodes, glabrous, smooth or scabrous; ***ligules*** 0.3–0.5(–1) mm long, apex truncate and erose; ***blades*** 5–8 cm long, 1–1.6 mm wide, flat or loosely folded to involute, scaberulous, apearing glabrous below with widely-spaced, stiff, short ascending hairs more common towards the apex, margins scabrous towards apex. ***Panicles*** 5–25(–30) cm long, open, diffuse, capillary, the base often partially enclosed by the sheath; ***primary branches*** 2–8 cm long, scabrous, naked below with very few spikelets, spreading 60–90° from the culm axis; ***pedicels*** 4–15 mm long, straight, scabrous; **disarticulation** above the glumes. ***Spikelets*** 3–3.5 mm long, plumbeous to dark-green turning golden brown with age; ***glumes*** 1.5–2.2 mm long, subequal, the lower a little shorter than the upper, 1-veined, apex acute, erose, often mucronate, the mucro less than 0.3 mm long; ***lemmas*** 3–3.5 mm long, lanceolate, 3-veined, puberulous, apex acute, minutely bifid, mucronate or awned between the teeth, the awn up 1.3 mm long, straight or slightly curled near apex, scaberulous; ***callus*** sparsely hairy; ***paleas*** 3–3.6 mm long, often slightly longer than the lemma, 2-veined; ***anthers*** about 1.2–1.6 mm long, purple. ***Caryopses*** not seen.

**Figure 15.**
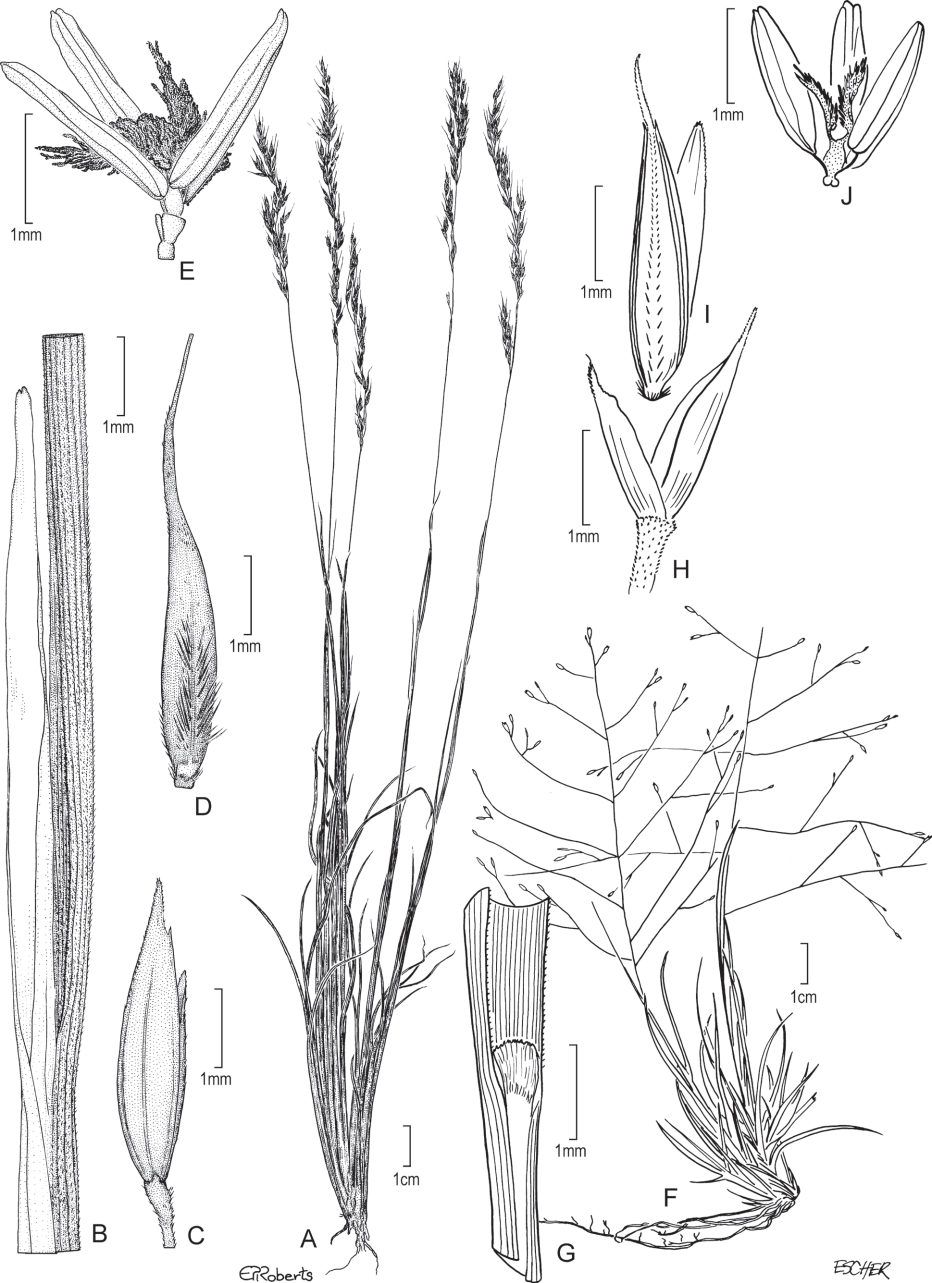
**A–E***Muhlenbergiaquadridentata* (Kunth) Trin. **A** habit **B** ligule **C** glumes **D** floret **E** stamens, pistil, and lodicules **F–J***Muhlenbergiaorophila* Swallen **F** habit **G** ligule **H** glumes **I** floret **J** stamens, pistil, and lodicules. **A–E** drawn from *P.M. Peterson & C.R. Annable 6201* (US) **F–J** drawn from *P.M. Peterson & C.R. Annable 11105* (US).

##### Distribution.

This species has been reported in Chiapas and the type was collected in Guatemala (Villaseñor Ríos 2016). It is also known from central México and has been reported in Ciudad de México, Hidalgo, Morelos, México, Puebla, and Tlaxcala (Espejo Serna et al. 2000; Villaseñor Ríos 2016; Sanchez-Ken 2018).

##### Ecology.

*Muhlenbergiaorophila* is found along creeks and wet areas in pine-fir forests; 3000–3860 m.

##### Comments.

Affinities of *M.orophila* are unknown and it has not yet been included in a DNA molecular study.

##### Specimen examined.

Guatemala. **Huehuetenango**: Chemal, vicinity of Chémal, summit of Sierra de los Cuchumatanes, *J.A. Steyermark 50309* (MO).

#### ﻿21. Muhlenbergia pereilema

Taxon classificationPlantaePoalesPoaceae

P.M. Peterson, Caldasia 31(2): 293. 2009.

AA6D201F-2977-5BAE-9CD4-590CEFCA5168

Fig. 16A–E

Pereilema crinitum J. Presl, Reliq. Haenk. 1(4–5): 233, t. 37, f. a–f. 1830. Type: Panama, *T. Haenke s.n.* (holotype: PR-198058!; isotypes: BR-0000006886257 [image!], HAL-0107173 [image!], LE-TRIN-1519.01!, M, MO-123263!, PR-849!, W-0029492 [image!], W-0029493 [image!], W-18890238189 [image!]). Basionym.
= Pereilema crinitum var. cirratum E. Fourn., Mexic. Pl. 2: 93. 1886. Type: México, Veracruz, Escamella, Orizaba, 24 Oct 1866, E. Bourgeau 3272 (**lectotype, designated here**: P-00751681 [image!]; isolectotypes: L-0062313 [image!], S14-29644 [image!], US-996097! ex P).

##### Description.

Delicate, caespitose, ***annuals***. ***Culms*** 15–90 cm tall, decumbent, rooting from flower nodes, mostly smooth and glabrous below the nodes; internodes usually glabrous to scaberulous. ***Leaf sheaths*** longer than the internodes, smooth or scaberulous; **ligule**s 0.5–1 mm long, membranous, eciliate, irregularly erose; ***blades*** 5–15 cm long, 2–3(–5) mm wide, flat, acuminate, auriculate near base; **auricles** 1–1.5 mm long, falcate. ***Panicles*** 5–20 cm long, 1–3 cm wide, spiciform, linear, continuous or interrupted; ***primary branches*** 0.5–3.5 cm long, appressed, the spikelets arising just above the sterile spikelets or bristles; **bristles** 2–4 mm long, antrorsely scabrous; ***pedicels*** 0.1–0.2 mm long, oblong, arising just above the bristles. ***Spikelets*** 1.5–2.6 mm long, greenish to stramineous, subtended by an involucre of persistent bristles, disarticulation below the glumes; ***glumes*** 0.8–1.5 mm long, equal, narrowly awl-shaped, awned, the awns 1–3 mm long; ***lemmas*** 1.5–2.6 mm long, ovate, keeled, 3-veined, with appressed hairs below, lateral veins close to margins, scabrous, apex acuminate, awned, the awns (15–)20–30 mm long, flexuous, callus hairy, the hairs 0.3–1 mm long; **palea** similar to lemma; ***stamens*** 3, ***anthers*** 0.5–0.7 mm long, yellow; **style** 1. ***Caryopses*** 0.8 mm long, ellipsoid, with adherent pericarp. *2n* = 20 (Pohl and Davidse 1971).

**Figure 16.**
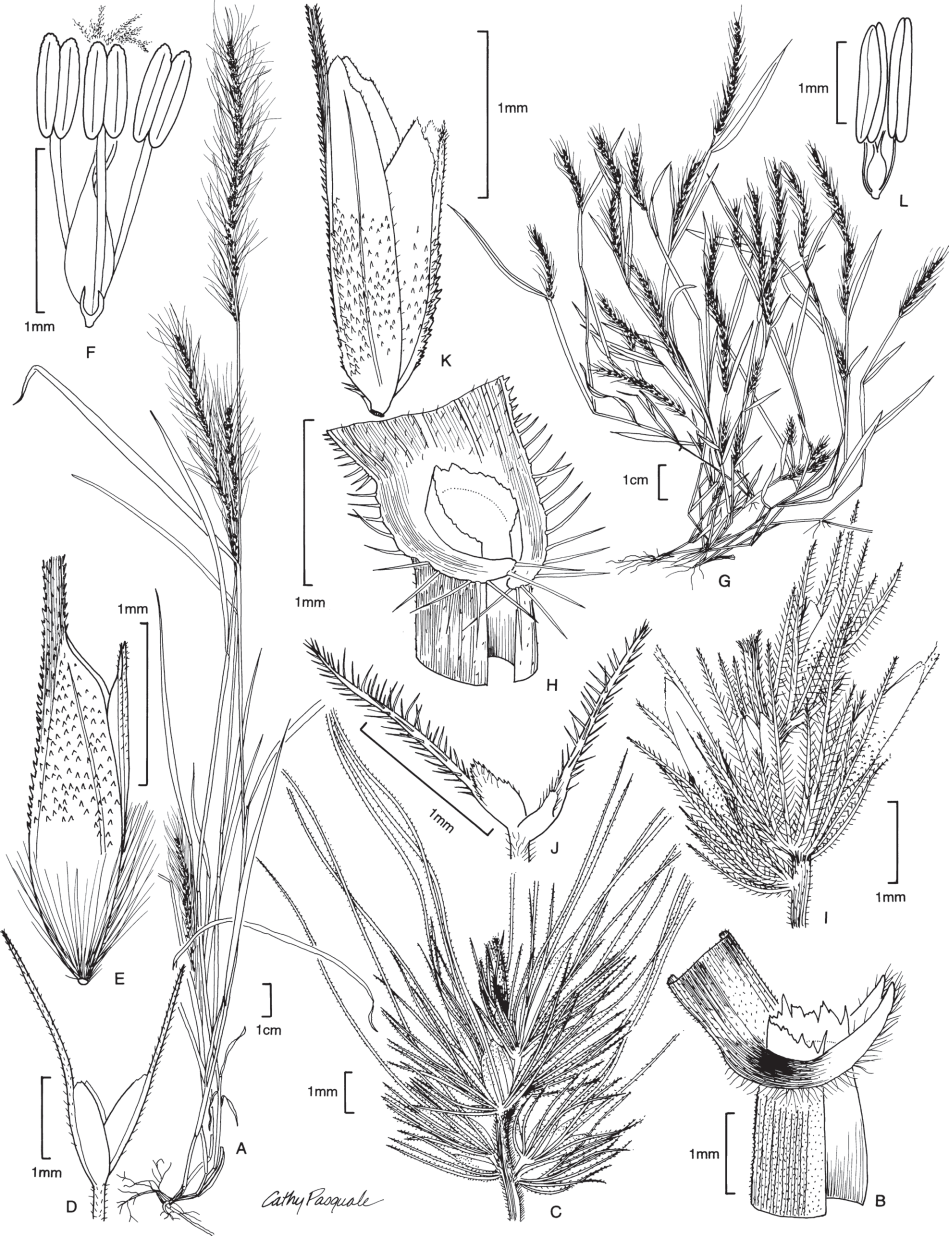
**A–F***Muhlenbergiapereilema* P.M. Peterson **A** habit **B** ligule with auricles **C** portion of the inflorescence **D** glumes **E** floret **F** stamens, pistil, and lodicules **G–L***Muhlenbergiaplumiseta* Columbus **G** habit **H** ligule with auricles **I** portion of the inflorescence **J** bristles **K** floret **L** stamens and pistil. **A–F** drawn from *A.S. Hitchcock 9050* (US) **G–L** drawn from *C.G. Pringle 5962* (US).

##### Distribution.

*Muhlenbergiapereilema* ranges from throughout México to Central (Costa Rica, El Salvador, Guatemala, Honduras, México, Nicaragua, and Panama) and South America (Peterson et al. 2001).

##### Ecology.

This species occurs on grassy slopes and rocky roadsides, ravines, and barrancas in tropical and oak-pine forests; 50–2450 m.

##### Comments.

*Muhlenbergiapereilema* can be separated from *M.plumiseta* in having persistent, scabrous bristles (plumose in *M.plumiseta*) and wider panicles (1–3 cm in *M.pereilema* and 0.2–0.6 cm wide in *M.plumiseta*) [Pohl 1994B; Herrera Arrieta et al. 2010; Herrera Arrieta and Peterson 2018]. This species forms a clade with *M.beyrichiana* Kunth and *M.plumiseta* within M.subg.Muhlenbergia (Peterson et. al. 2021).

##### Specimens examined.

Costa Rica. **Alajuela**: Atenas, Balsa, alrededores de las Escuela Centroamericana de Gandería, *J. Gómez-L 6127* (CR); 1 km S Carrizal, open roadside in coffee plantation, *R.W. Pohl & G. Davidse 11502* (US, CR); La Garita, dam in the canyon of the Rio Grande de Tarcoles, *R.W. Pohl & G. Davidse 11494* (US, CR); 1 km N of Grecia, *R.W. Pohl & G. Davidse 11523* (US, CR). **Cartago**: Roadside N of Puente Negro, N of Orosi, *R.W. Pohl & M. Lucas 13153* (MO, CR); Turrialba, Près de la station de Juan Viñas, *H. Pittier 1759* (CR); San Juan Norte, *R. W. Pohl & G. Davidse 11435* (US, CR); vicinity of Finca Las Concavas, *P.C. Standley 41551* (US). **Heredia**: Orillas de la vía férrea a Heredia, *A.M. Brenes 13284* (CR); Barva, bordes del Río Segundo, *J. León 415* (CR); El Gallito de Heredia, *M. Valerio 540* (CR). **Puntarenas**: Paturage a Boruca, *A. Tonduz 3689* (CR); Savannas de Boruca, *H. Pittier (Tonduz*, *A.) 4450* (US, CR); Boca de Barranca, *M. Montiel s.n.* (USJ); Bord du chemin a Mano de Tigre, *H. Pittier (Tonduz*, *A.) 4627* (US, CR). **San José**: Acosta, camino a Bajo Palma, *J.F. Morales 7439* (INB, MO); Puriscal, Alto La Escalera, a lo largo del camino entre San Ignacio y Guaitil, cuenca del Pirris-Damas. Valle del Candelaria, Alto La Escalera en el camino a Bajo Arias y La Cruz, *J.F. Morales & B. Hammel 6049* (MO, INB, CR); Acosta, Cuenca del Pirris-Damas, Cerros de Caraigres, cabeceras del Río La Mesa, cerca Ceiba Este, *J.F. Morales & J.F. Corrales 6032* (MO, INB); Mora, Colón, le long des chemins dans la vallée du rio Jaris, prés de Pacaca, *H. Pittier 3329* (CR); Mora, Colón, en la orilla del camino de lastre que va de Brasil a Ciudad Colón, *S. Lobo 2309* (CR); Escazú, San Antonio, *D. Santamaría 3648* (INB, MO); Bordes líneas férreas San José-Guadalupe, *A. Tonduz 709* (CR); Hacienda La Esperanza, La Palma, *O. Jiménez 973* (US, CR); Sur un rocher au bord du rio Virilla, sous le pont du F. C. R., près San Juan, *A. Tonduz 17556* (CR); 13 km N of San Isidro de El General along carretera interamericana, *R. W. Pohl & G. Davidse 11626* (US, CR); San Pedro, *W.A. Archer 3888* (US); Tibas, collected along the Río Virilla about 1 km S of Santo Domingo, *J. Taylor 17313* (MO). El Salvador. **Ahuachapán**: Ahuachapán, *S.A. Padilla 134* (US); San Benito, al N de la cima del cerro La Olla, *E. Sandoval & Chinchilla 767* (LAGU, MO). **La Libertad**: Nueva San Salvador, Jardin Botanico La Laguna, *W.G. Berendsohn 1003* (LAGU, MO); Boqueron del Volcán de San Salvador, *L. E. González 1851* (MO, ITIC). **San Salvador**: San Salvador, *S. Calderón 494* (MO); San Martin, *S. Calderón 1902* (US); Cerro el Guayabal, *S. Calderón 1964* (US). Guatemala. **Alta Verapaz**, Coban, *H. von Türckheim 743* (MO); Cobán, *H. von Türckheim 1362* (MO); *H. von Türckheim 1509* (MO, US); Area of mixed forest and clearings, hills about 10 km south of Coban, *L. O. Williams et al. 40051* (US); Finca aved near Coban, *W. Popenoe 903* (US). **Chimaltenango**: along road from Chimaltenango to San Martin Jilotepeque, *P.C. Standley 57894* (US). **Chiquimula**: Volcan Ipala, near Amatillo, bordering lake on top, *J.A. Steyermark 30491* (US). **Huehuetenango**: Thickets and forest in deep canyon of a tributary of Rio Blanco, about 5 km W of Aguacatan, *L.O. Williams et al. 22338* (US). **Jalapa**: vicinity of Jalapa, pine-oak forest, *P.C. Standley 76749* (US). Jutiapa: Volcan Chingo, *J. Donnell Smith 3673* (US). **Guatemala**: Guatemala City, *A.S. Hitchcock 9050* (US); Guatemala City, rocky hill, *A.S. Hitchcock 9031* (US); Guatemala City, barranca north of Guatemala city, *W. Popenoe 733* (US); San Antonio, Las Roces, *Rojas 385* (US). **Santa Rosa**: Chupadero, *J. Donnell Smith 3914* (US); Cerro Redondo, *J. Donnell Smith 6274* (US). **Solola**: Mixed forest area, mountain slopes above Lake Atitlan, about 3–5 km W of Panajachel, *L.O. Williams et al. 25278* (US). Honduras. **El Paraíso**: Yuscarán, la Piedra de Apaguiz, 3.5 km al SE de Danlí, *N.P. Estrada 137* (MO, CR, TEFH); Arauca, Las Manos, 5 km N of Las Manos, near Los Limones, *R.W. Pohl & M. Gabel 13429* (MO, CR); Road to Danlí, near Río San Francisco, *J. R. Swallen 11196* (MO); Road to Yuscaran, *J.R. Swallen 11347* (US). **Francisco Morazán**: Distrito Central, Cerro el Hatillo, 15 km al NE de Tegucigalpa; bosque premontano húmedo, *L.M. Ordóñez 67* (MO); San Antonio de Oriente, quarry above El Zamorano, on road to San Antonio de Oriente, *R.W. Pohl & G. Davidse 12505* (MO, CR); vicinity of El Zamorano, along “Wood Road”, *J.R. Swallen 10821* (US); In pine woods near Piedra Herrada, Mt. Uyuca region, *L.O. Williams 18543* (US); Drainage of the Rio Yeguare, Moist rocky bank near Agua Amarilla, in oak-pine forest, *L.O. Williams & A. Molina R. 14723* (US). **Olancho**: Jutiapa Forest Camp, near Salamá. Pine forest on a steep slope above a stream, *R.W. Pohl & M. Gabel 13749* (MO, CR). Mexico. **Chiapas: Angel Albino Corzo**: slopes of Río Cuxtepec, below Finca Cuxtepec, *D.E. Breedlove & G. Davidse 54676* (MEXU, CAS). Arriaga: 13 km N of Arriaga along Mex. Hwy. 195, *D.E. Breedlove & G. Davidse 54149* (CAS, MO); slope at El Sumidero, 22 km N of Tuxtla Gutierrez, *D.E. Breedlove & P.H. Raven 13387* (DS); along ravines 13 km N of Arriaga along Mexican Highway 195, *D.E. Breedlove 28278* (MO). **Cintalapa**: 23 km W of Las Cruces along road to La Mina Microwave Station, *D.E. Breedlove & G. Davidse 54092* (CAS, MO). Chiapa de Corzo: above El Chorreadero, *D.E. Breedlove & G. Davidse 54032* (MEXU, CAS, MO); 36 km E of Tuxtla Gutierrez, *F.W. Gould & S. Hatch 14374* (ISC, DS); Ixhuatán: 2 km al N of Ishuatan, *G. Davidse et al. 29641* (MEXU, MO). Ixtapa: intersection of Tuxtla Gutiérrez-San Cristobal de las Casas and Villahermosa, *G. Davidse et al. 30104* (MEXU, MO); near Ixtapa, *D.E. Breedlove & G. Davidse 54264* (CAS, MO); along Mex Hwy 190 in the Zinacantan paraje of Muctajoc, *D.E. Breedlove 13819* (DS). **Motozintla**: Motozintla de Mendoza, 25–27 km NE of Huixtla along rd to Motozintla SW of Toliman, *D.E. Breedlove 28593* (CHAPA, MEXU, DS, MO); Ejido Toliman, sobre la carr. Huixtla-Motozintla. Vega de Arroyo, *Gómez et al. 185* (MEXU). **Tenejapa**: In the paraje of Mahosik’, *D.E. Breedlove 16146* (DS). **Villa Corzo**: Above Colonia Vicente Guerrero on road to Finca Cuxtepec, *D.E. Breedlove & L. Strother 46573* (CAS, MO); Above Colonia Vicente Guerrero on road to Finca Cuxtepec, *D.E. Breedlove & G. Davidse 54567* (MEXU, CAS, MO). San Fernando: Parque Nacional del Sumidero, 20–22 km NW of Tuxtla Gutiérrez, *G. Davidse et al. 29767* (MEXU, MO). Nicaragua. Rivas, Volcán Concepción, Isla Ometepe, plantas propias de la lava, *W. Robleto T. 157* (MO, CR). Panama. Coclé, vicinity of Ola, *H. Pittier 5046* (US).

#### ﻿22. Muhlenbergia peruviana

Taxon classificationPlantaePoalesPoaceae

(P. Beauv.) Steud., Nomencl. Bot. (ed. 2) 1:41. 1840.

91C97AF7-37B3-5DFF-A062-5BAF5D8877FC

Fig. 12A–D

Clomena peruviana P. Beauv., Ess. Agrostogr. 28, t. 7, f. 10; t. 3, f. 20. 1812. Agrostisperuviana (P. Beauv.) Spreng., Syst. Veg. 1:262. 1825. Type: Peru, M. Thibaut s.n. (holotype: P!; isotype: E-00373717 [image!]). Basionym.
= Clomena peruviana var. pulvinata Nees, Gramineae 12–13. 1841. Muhlenbergiaperuvianavar.pulvinata (Nees) Nees & E. Mey. ex Kuntze, Revis. Gen. Pl. 3(3):357. 1898. Type: Peru, Lago Titicaca, Apr, *J.F.J. Meyen s.n.* (holotype: B; isotype: US-3376134 fragm. ex B!).
= Muhlenbergia nana Benth., Pl. Hartw. 262. 1846. Type: Ecuador, Mt. Cotopaxi, 1843, *Hartweg 1458* (holotype: K!; isotypes: BAA-1629!, K!, LE!, P!, US-91916 fragm. ex P!, US-995896 fragm. ex P-STEUD & fragm. ex BR!).
= Muhlenbergia pusilla Steud., Syn. Pl. Glumac. 1:177. 1854. Type: México, México, Valley of Toluca, Oct. 1827, J.*L. Berlandier 1141* (holotype: P!; isotypes: BAA-1635!, K!, MO-2974185!, P!, US-1084517!, US-2561239!, US-91910 fragm. ex P!).
= Epicampes bourgeaei E. Fourn., Mexic. Pl. 2: 88. 1886. Type: México, Veracruz, Escamala, Refrou D’Orizaba, 26 Aug 1866, *E. Bourgeau 2973* (holotype: P!; isotype: US-A0865984 fragm ex P!).
= Muhlenbergia bourgeaei E. Fourn., Mexic. Pl. 2:86. 1886. Type: México, Valle de México, Desierto Viejo, 3 Nov 1865, M. Bourgeau 1309 (lectotype: P! designated by Peterson and Annable, Syst. Bot. Monogr. 31:73. 1991; isotype: US-87243 fragm. ex P!). ≡ Epicampesbourgeaei (E. Fourn.) M.E. Jones, Contr. W. Bot. 14:7. 1912, *nom. illeg. hom.*
= Muhlenbergia pulcherrima Scribn. ex Beal, Grass. N. Amer. 2:240. 1896. Type: México, Chihuahua: Sierra Madres, dry ledges of porphyry, 30 Sep 1887, *C.G. Pringle 1416* (holotype: MSC!; isotypes MO-3727978!, NY!, US-995494!, VT!).
= Muhlenbergia peruviana var. elatior Kuntze, Revis. Gen. Pl. 3(2): 357. 1898. Type: Bolivia, Tunarigebirge, 3000 m, May 1892, *Kuntze s.n.* (lectotype: NY! designated by Peterson and Annable, Syst. Bot. Monogr. 31:73. 1991: isotype: fragm. & photo US!).
= Muhlenbergia peruviana var. subcaespitosa Kuntze, Revis. Gen. Pl. 3(3):357. 1898. Type: Bolivia, Tunari Mts., 4600 m, 4 May 1892, *Kuntze s.n.* (lectotype: NY! designated by Peterson and Annable, Syst. Bot. Monogr. 31:73. 1991).
= Muhlenbergia peruviana fo. versicolor Kuntze, Revis. Gen. Pl. 3(3):357. 1898. Type: Bolivia, Tunarigebirge, 3000 m, May 1892, *Kuntze s.n.* (lectotype: NY! designated by Peterson and Annable, Syst. Bot. Monogr. 31:73. 1991; isotype: US fragm. ex NY!).
= Muhlenbergia peruviana fo. viridis Kuntze, Revis. Gen. Pl. 3(3):357. 1898. Type: Bolivia, Puna, 4000 m, 11 Mar 1892, *Kuntze s.n.* (lectotype: NY! designated by Peterson and Annable, Syst. Bot. Monogr. 31:73. 1991).
= Muhlenbergia herzogiana Henrard, Meded. Rijks-Herb. 40:58. 1921. Type: Bolivia, Cordillera de Santa Bonita, Jun 1911, *T. Herzog 2226* (holotype: L!; isotypes: US-87248 fragm. ex L!, US-1161342!, W-1926-23724!).

##### Description.

Tufted ***annuals***. ***Culms*** 3–27 cm tall, erect, glabrous. ***Leaf sheaths*** usually longer than the internodes, smooth or scabridulous; ***ligules*** 1.5–3 mm long, membranous, acute; ***blades*** 1–5 cm long, 0.6–1.5 mm wide, flat to involute, smooth or scabridulous abaxially, sometimes shortly pubescent adaxially. ***Panicles*** 2–8 cm long, 0.3–3.4 cm wide, contracted or open; ***primary branches*** 1–5 cm long, diverging up to 80° from the rachises; ***pedicels*** 0.4–5 mm long, smooth or scabrous. ***Spikelets*** 1.4–4.2 mm long, 1-flowered; ***glumes*** smooth or scabridulous; ***lower glumes*** 0.8–2.8 mm long, narrow to broadly lanceolate, 1-veined, acute, often awn-tipped; ***upper glumes*** 0.9–3 mm long, wider than the lower glumes, lanceolate, 3(2)-veined, truncate to acute, 2- or 3-toothed; ***lemmas*** 1.4–4.2 mm long, ovate, widest near the base, purplish mottled with dark green areas, hairy on the calluses and lower 2/3 of the lemma bodies, hairs to 0.5 mm long, apices acuminate, usually bifid and awned from between the teeth, teeth to 0.5 mm long, awns 3–10 mm long, flexuous, purplish; ***paleas*** 1.3–3.8 mm, narrowly lanceolate, acuminate to subacute; ***anthers*** 0.5–1 mm long, purplish to yellowish. ***Caryopses*** 1–1.6 mm long, fusiform, brownish. 2*n* = 30.

##### Distribution.

*Muhlenbergiaperuviana* occurs in Arizona and New Mexico, U.S.A, throughout México to Guatemala, and then in Argentina, Bolivia, Chile, Ecuador, and Peru (Peterson and Annable 1991).

##### Ecology.

Grassy flats, open gravelly flats, rock outcrops, sandy washes, gravelly drainages, wet or dry meadows, canyons, gravelly or sandy slopes, valleys, shores along lakes, open ridgetops, and disturbed road cuts associated with *Aciachnepulvinata* Benth., *Anatherostipa*, *Berberis*, *Colletiaspinosissima*, *Ephedra*, *Festucaorthophylla* Pilg., *Festuca* ssp., *Jarava*, *Juncus*, *Lepidophyllum*, *Luzula*, *Margyricarpus*, *Monnina*, *Muhlenbergia* ssp., *Nassella*, *Plantago*, *Poa* ssp., *Polylepis*, *Puya*, *Pycnophyllum*, *Salviaoppositiflora* Ruiz & Pav., *Stevia*, *Tagetes*; 3000–4900 m.

##### Comments.

As treated here, *Muhlenbergiaperuviana* includes (as synonyms) what was sometimes identified as *M.pulcherrima* Scribn. ex Beal (southwestern USA and northern México) and *M.pusilla* Steud. (central México to Guatemala). There are many more morphological forms than just these, and since the only chromosome count of this species suggests triploidy (2*n* = 3*x* =30), perhaps this species is apomictic (Reeder 1968). We believe apomixis is occurring in this species but that it is not obligate, and that gene flow takes place sporadically to form intermediates maintained by asexual seed formation (Peterson and Annable 1991).

In a molecular DNA sequence analysis *Muhlenbergiaperuviana* is sister to *M.crispiseta* Hitchc., another annual known only from Texas and north central México, and this pair is embedded in the strongly supported M.subg.Clomena clade (Peterson et al. 2010b, Peterson et al. 2021). Members of M.subg.Clomena possess spikelets with upper glumes that are 3-veined and often 3-toothed and have a densely caespitose habit (Peterson et al. 2010b). *Muhlenbergiaperuviana* can be separated from *M.crispiseta* in having purplish irregularly flexuous, purplish awns (versus sinuous-wavy, crisped and curled, olive-green awns in *M.crispiseta*) and narrow, gradually acuminate lemmas (versus lemmas that are plump near middle) [Peterson and Annable 1991].

##### Specimens examined.

Guatemala. **Huehuetenango**: La Capellania, Sierra de los Cuchumatanes, 1.0 miles NW of La Capellania on hwy 9N and 12.3 miles N of Huehuetenango, *P.M. Peterson & C.R. Annable 4683* (GH, MEXU, MICH, MO, NY, RSA, UC, US, WS); Tojiah, Sierra de los Cuchumatanes, 3 miles SW of Tojiah on hwy 9 N., *P.M. Peterson & C.R. Annable 4698* (GH, MO, NY, RSA, US, WS); Treeless paramo-like plain near Calaveras, *L.O. Williams et al. 21961* (US). **San Marcos**: Tacana, Volcán Tacaná, *M. Véliz et al. 10587* (MO). Mexico. Chiapas: NW de Motozintla de Mendoza on road to El Porvenir, *P.M. Peterson & C.R. Annable 4712* (US); 13 mi NW de Motozintla de Mendoza off road to El Porvenir at top of cumbre below tower, *P.M. Peterson & C.R. Annable 4714* (US).

**Figure 17.**
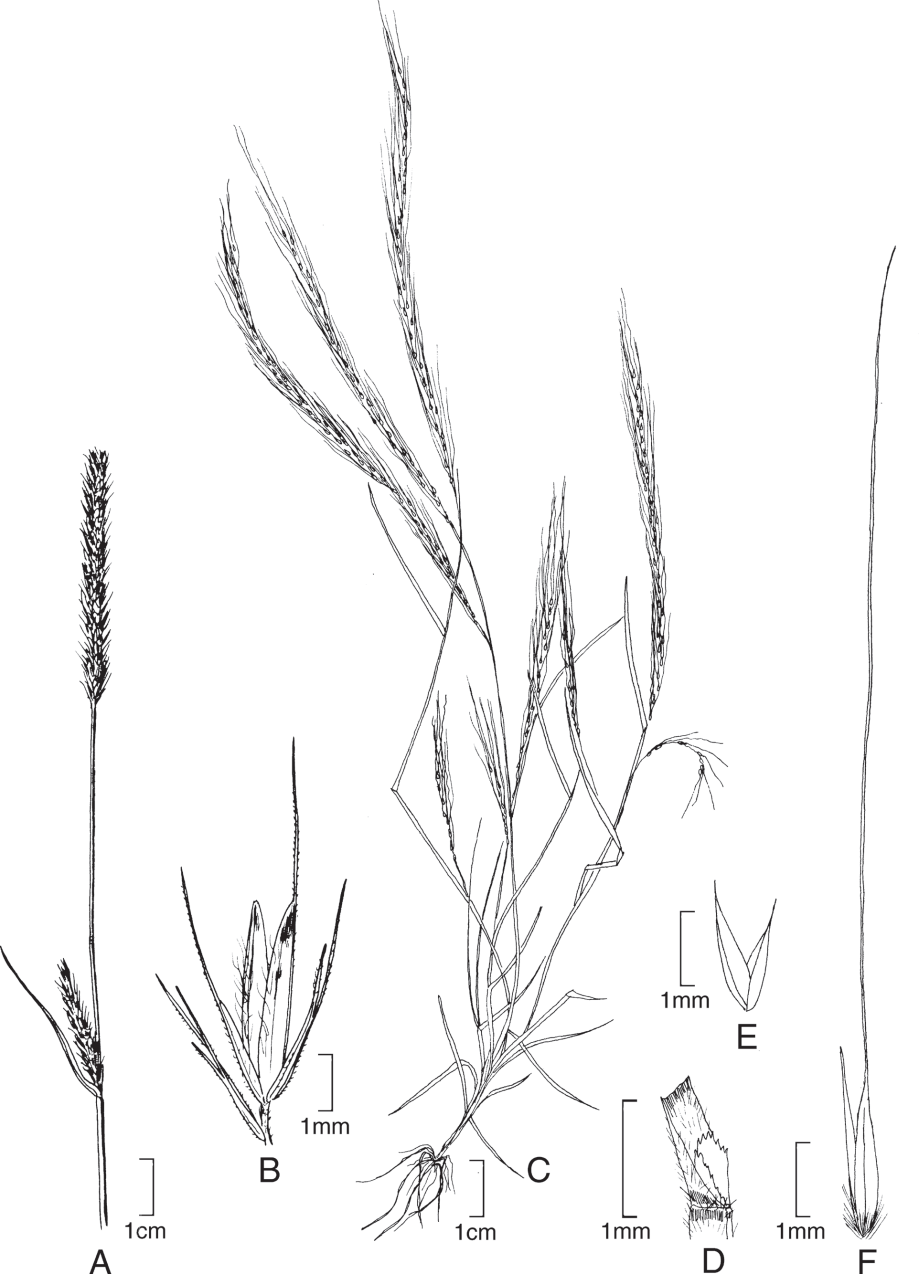
**A, B***Muhlenbergiaphalaroides* (Kunth) P.M. Peterson **A** culm with inflorescence **B** spikelet **C–F***Muhlenbergiatenella* (Kunth) Trin. **C** habit **D** ligule **E** glumes **F** floret. **A, B** drawn from *S. Lægaard 71419* (AAU) **C–F** drawn from *P.M. Peterson & C.R. Annable 4755* (US, WS).

#### ﻿23. Muhlenbergia phalaroides

Taxon classificationPlantaePoalesPoaceae

(Kunth) P.M. Peterson, Caldasia 31(2): 294–296, f. 7 A–B. 2009.

823DDD10-C19A-537B-9653-38BB45F716E0

Fig. 16A–B

Lycurus phalaroides Kunth, Nov. Gen. Sp. (quarto ed.) 1: 142. 1815 (1816). Type: México, Michoacán, near Valladolid, Alberca de Palangeo and Patzcuaro, Sep, *F.W.H.A. Humboldt & A.J.A. Bonpland s.n.* (holotype: P-00669405 [image!]; isotypes: B-W-1630, BM!, BAA-1530!, US-91988 fragm. ex P-BONPL!, US-610837 fragm. ex LE-TRIN!). Basionym.
= Muhlenbergia lycuroides Vasey ex Beal, Grass. N. Amer. 2: 239. 1896. Type: México, Jalisco, Guadalajara, Jul–Oct 1886, *E. Palmer 489* (holotype: MSC; isotypes: GH-00023916 [image!], LE, MEXU, MO-2972929!, NDG-07247 [image!], NY, P-00644181 [image!], P-00644182 [image!], S14-29628 [image!], US-822925!, US-81642!, YU-000898 [image!]).
= Lycurus phleoides var. brevifolius Scribn. ex Beal, Grass. N. Amer. 2: 271. 1896. Type: México, Jalisco, plains of Guadalajara, 23 Oct 1889, *C.G. Pringle 2470* (lectotype: MSC, designated by C. Reeder, Phytologia 57(4): 288. 1985; isolectotypes: BAA!, GH, MEXU, MO-2972926!, NY!, P-00644183 [image!], P-00644184 [image!], US-996049!, W-18900000580 [image!], W-19160029092 [image!]).

##### Description.

***Perennials***, intricately branched near base. ***Culms*** 10–30 cm tall, erect, mostly glabrous, usually decumbent and sprawling below, bent at the pubescent to short pilose nodes; ***internodes*** 0.4–10(–15) cm long, pubescent to short pilose. ***Leaf sheaths*** much shorter than the internodes above, hyaline near the margins, pilose near summit; ***ligules*** 0.4–1 mm long, membranous, apex truncate to deltoid, often erose and lacerate; ***blades*** 0.5–6.5 cm long, 0.5–1.2 mm wide, shorter near the base of culms, flat, folded or loosely involute, lanate above and glabrous or with scattered, short appressed hairs below, margins whitish-thickened, apex navicular, occasionally with a short seta, seta usually less than 2 mm long. ***Panicles*** 1.5–6.5 cm long, 3–8 mm wide, spiciform and spikelike, densely flowered, often interrupted below with only a few spikelets, terminal or axillary; **rachis** lanate to hispid, the short hairs antrorse, appressed; ***primary branches*** 1.5–7 mm long, very short, the spikelets usually in pairs, rarely 1 or 3 per terminal branch, when in pairs the lower short-pedicelled spikelet perfect, staminate or sterile and the upper longer-pedicelled spikelet usually perfect; ***pedicels*** 0.3–1.4 mm long; **disarticulation** usually at the base of the pedicel, each spikelet falling as a unit leaving a small cuplike tip. ***Spikelets*** 3–4 mm long, stramineous with plumbeous mottles, sometimes additionally with purplish mottles; ***glumes*** 1–2.1 mm long, shorter than the lemma, subequal, 1–3-veined; ***lower glumes*** commonly 2 or 3-veined, usually 2-awned, occasionally 1 or 3 awned, the awns 1–3 mm long, equal or subequal, scabrous, recurved; ***upper glumes*** commonly 1-veined, usually 1-awned, the awns 1–2.5 mm long; ***lemmas*** 3–4 mm long, narrowly lanceolate, 3-veined, margins hirsute to lanate and occasionally the lower ½ sparsely hairy, the hairs 0.1–0.3 mm long, apex usually awned, occasionally unawned or mucronate, the awns 1–3 mm long; ***paleas*** 2.8–3.8 mm long, hairy between the veins, the veins occasionally extending as mucros; anthers 1.3–2 mm long, yellowish. ***Caryopses*** 1.7–2 mm long, fusiform, brownish.

##### Distribution.

*Muhlenbergiaphalaroides* ranges from México to South America where it is found in Argentina, Bolivia, Columbia, Ecuador, and Peru (Reeder 1985; Sánchez and Rúgolo de Agrasar 1986; Davidse and Pohl 1994).

##### Ecology.

This species occurs in open grasslands and savannahs on steep rocky slopes flats, and along disturbed irrigation canals in deep clayish-loam to sandy soils associated with *Baccharis*, *Berberis*, *Cheilanthes*, *Condalia*, *Dodonaeaviscosa*, *Eragrostis*, *Jarava ichu Opuntia*, *Muhlenbergiacenchroides*, *M.rigida*, *Nassella*, *Plantago*, *Puya*, and *Sporobolusindicus* (L.) R. Br.; 2800–3500 m.

##### Comments.

*Muhlenbergiaphalaroides* is morphologically similar to *M.phleoides* (Kunth) Columbus known in the southwestern USA and México, and *M.alopecuroides* (Griseb.) P.M. Peterson & Columbus found in the southwestern USA, México, and disjunct in Argentina and Bolivia (Reeder 1985; Davidse and Pohl 1994, Renvoize 1998; Peterson and Giraldo-Cañas 2012). *Muhlenbergiaalopecuroides* differs from *M.phalaroides* in having leaf blades with terminal seta (3–)4–7(–12) mm long and ligules (2–)3–12 mm long whereas *M.phleoides* differs in having auriculate ligules 1–2 mm long (Reeder 1985; Peterson 2003). These morphological differences are perhaps better recognized at the subspecific level but there are no population studies comparing these three species, other than Peterson and Morrone (1997) who investigated populations of only the amphitropical, *M.alopecuroides* [as *Lycurussetosus* (Nutt.) C. Reeder].

*Muhlenbergiaphalaroides* probably lies within M.subg.Pseudosporobolus, although the species has not been included in a DNA-derived phylogeny, aligning with *M.alopecuroides* and *M.phleoides* (Peterson et al. 2021). Many members of this subgenus have narrow panicles, plumbeous spikelets with unawned, mucronate or short-awned lemmas (Peterson et al. 2010b).

##### Specimens examined.

Guatemala. **Quetzaltenango**: Chiquilaja, potreros naturales y secos, *M. de Koninck 63* (US). Mexico. **Chiapas**: **San Cristóbal**: NE edge of San Cristóbal de Las Casas, *D.E. Breedlove & G. Davidse 46039*, *54734* (MEXU). **Teopisca**: N of Teopisca, *D.E. Breedlove & G. Davidse 54768* (MEXU).

#### ﻿24. Muhlenbergia plumbea

Taxon classificationPlantaePoalesPoaceae

(Trin.) Hitchc., Contr. U.S. Natl. Herb. 17(3): 296. 1913.

3CDE7B5E-E3D6-51BD-9A55-575B4E260A65

Fig. 18A–E

Vilfa plumbea Trin., Mém. Acad. Imp. Sci. Saint-Pétersbourg, Sér. 6, Sci. Math., Seconde Pt. Sci. Nat. 6,4(1–2): 98. 1840. Type: México, Mineral del Monte, *Schlechtendal s.n.* (holotype: TRIN-1724.01!; isotype US-557435! fragm. ex LE). ≡ Sporobolusplumbeus (Trin.) Hemsl.. Biol. Cent.-Amer., Bot. 3(19): 546–547. 1885. Basionym.
= Sporobolus pooides Hack., Repert. Spec. Nov. Regni Veg. 10(243–247): 167. 1912[1911]. Type: México, Puebla, Rancho Posada, 2194m, Aug 1910, *F.G. Nicolas 5423* (holotype: W1916-0032028 [image!]; isotype: US-87219! fragm. ex W).

##### Description.

***Perennials*** with slender, scaly rhizomes; **rhizome scales** 7.5–16 mm long, acute often deteriorating with age. ***Culms*** 10–40(–50) cm tall, erect, decumbent near base, little or much branched below, glabrous; ***internodes*** 0.4–6 cm long, glabrous, smooth to nodulose-roughened, the nodes green or purple, constricted. ***Leaf sheaths*** 1–5 cm long, mostly longer than the internodes, glabrous, margins hyaline; ***ligules*** 0.3–0.5(–0.7) mm long, membranous, truncate, decurrent; ***blades*** 2–10(–12) cm long, 1–2.4(–3.0) mm wide, flat or folded, glabrous. ***Panicles*** 4–9(–14) cm long, 0.5–4(–8) cm wide, open, usually well exerted with 6–12 branches; ***primary branches*** mostly 2–8 cm long, ascending and spreading up to 50° from the culm axis, with 3–30 spikelets, widely spaced, naked below, one per node, scabrid; ***pedicels*** 0.5–2 mm long, shorter than the spikelets, scaberulous. ***Spikelets*** 2.5–3.2(–3.5) mm long, plumbeous; ***glumes*** 1–1.6 (–1.8) mm long, shorter than the florets, equal or subequal, the upper often slightly longer than the lower, 1-veined, apex acute; ***lemmas*** 2.2–3(–3.3) mm long, lanceolate to ovate, glabrous or occasionally scabrous, faintly 3-veined, dark green, apex acute; ***paleas*** 2–2.8(–3.1) mm long, about as long as the lemma, glabrous, faintly 2-veined, apex acuminate; ***anthers*** 1.4–2 mm long, dark green or purplish turning yellow with age. ***Caryopses*** 1.4–1.6 mm long, ellipsoid, greenish-brown. 2*n* = 40 (Reeder 1967).

**Figure 18.**
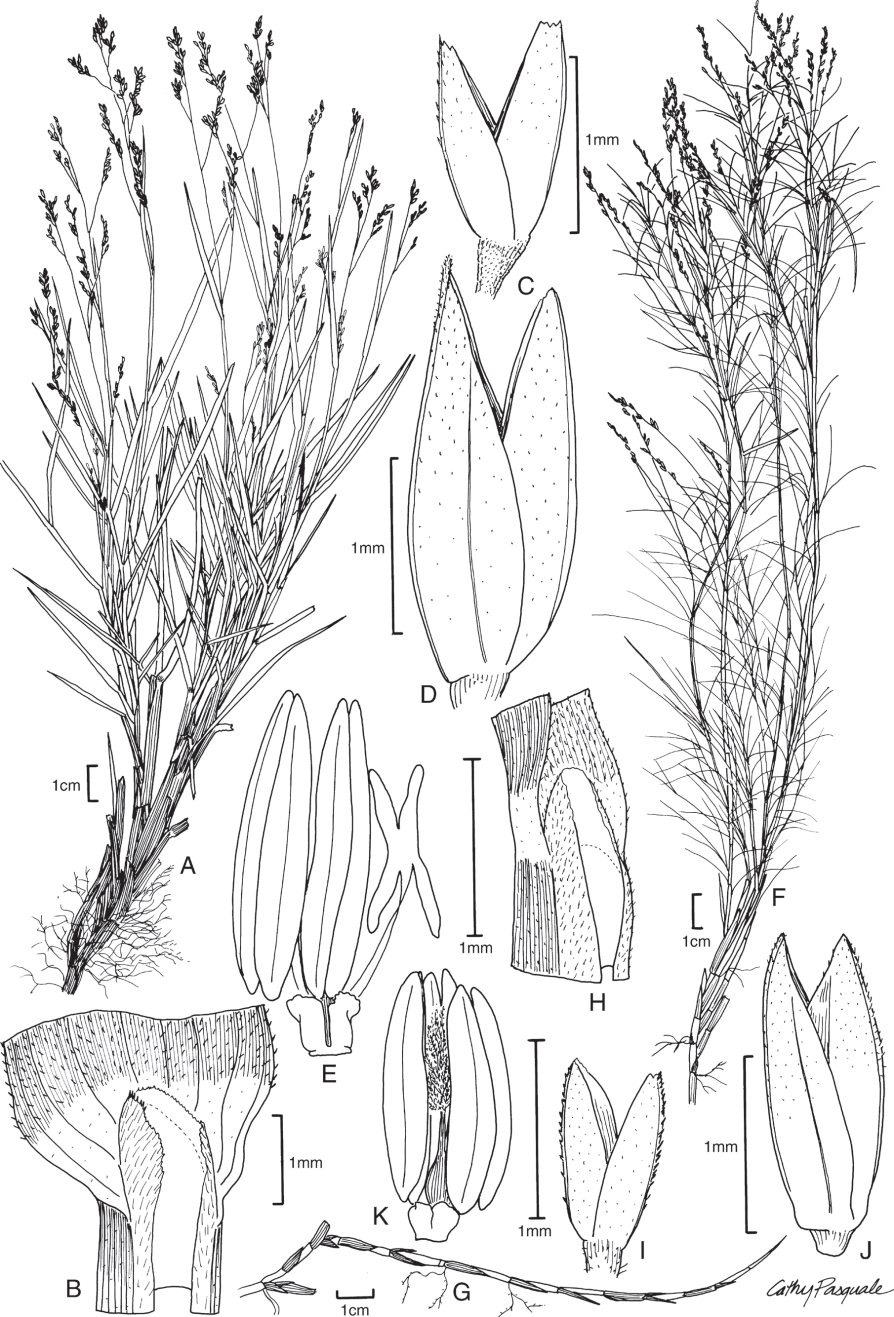
**A–E***Muhlenbergiaplumbea* (Trin.) Hitchc. **A** habit **B** ligule **C** glumes **D** floret **E** stamens and lodicules **F–L***Muhlenbergiautilis* (Torr.) Hitchc. **F** habit **G** rhizome **H** ligule **I** glumes **J** floret **K** stamens, pistil, and lodicules. **A–E** drawn from *C.G. Pringle 9581* (US-396634) **F–L** drawn from *A*,*S. Hitchcock 5652* (US).

##### Distribution.

In Central America *M.plumbea* is known only from a single specimen collected in Guatemala. The species is wide ranging in México having been reported in Baja California, Chihuahua, Ciudad de México, Coahuila, Durango, Guanajuato, Hidalgo, México, Michoacán, Puebla, San Luis Potosí, Sonora, Tlaxcala, and Zacatecas. (Dávila et al. 2018; Sanchez-Ken 2018).

##### Ecology.

This species occurs in wet depressions and alkaline meadows associated with pine and fir forests but is often collected near cultivated fields; 1800–3050m.

##### Comments.

*Muhlenbergiaplumbea* is morphologically similar to *M.utilis*, in Central America known only from Chiapas, the former having longer panicles [4–9(–14) cm versus 1–5 cm] that are usually well exserted (partially included in the upper sheath in *M.utilis*) with longer primary branches (2–8 cm versus 0.2–1.2 cm], longer spikelets [2.5–3.2(–3.5) mm versus 1.4–2.4 mm], and longer anthers [1.4–1.6 mm versus 0.7–1.4 mm].

This species has not been included in a molecular DNA sequence study to date but based on morphology it appears allied with the *M.filiformis–M.ligularis–M.vaginata* clade in M.subg.Bealia or the *M.repens* (J. Presl) Hitchc.–*M.utilis*–*M.villifora* Hitchc. clade in M.subg.Pseudosporobolus (Fig. 1; Peterson et al. 2021).

##### Specimens examined.

Guatemala. Tan abundante como el “Zacachiquin”, 19 Jun 1954, *M. de Koninck 31* (US-2151621).

#### ﻿25. Muhlenbergia plumiseta

Taxon classificationPlantaePoalesPoaceae

Columbus, Aliso 28: 66. 2010.

7409BC80-8719-5101-A012-25D5FE6CE3DA

Fig. 16 G–L

Pereilema ciliatum E. Fourn., Mexic. Pl. 2: 93. 1886. Type: México, region Orizaba, 8 Nov 1866, *E. Bourgeau 3328* (**lectotype, designated here**: P-00751680!; isolectotypes: K-000308948 [image!], L-0062312 [image!], MPU-026837 [image!], US-996083!). ≡ Muhlenbergiaplumosa P. M. Peterson, Amer. J. Bot. 97(9): 1546. 2010, isonym. Basionym.

##### Description.

Delicate, caespitose, ***annuals*. *Culms*** 15–50 cm tall, decumbent, rooting at the lower nodes, smooth and glabrous below the nodes; internodes usually glabrous to scaberulous. **Leaf** s**heaths** shorter than the internodes, scaberulous or smooth; ***ligules*** 0.2–0.4 mm long, membranous, apex truncate, erose; ***blades*** 4–10 cm long, 1.5–3 mm wide, flat, acuminate, auriculate near base, the **auricles** 0.5–1 mm long, clasping, ciliate. ***Panicles*** (2–)3–8 cm long, 0.2–0.6 cm wide, spiciform, linear, continuous; ***primary branches*** 0.2–0.8 cm long, tightly appressed, pilose, ascending, frequently glandular, the spikelets arising just above the sterile spikelets or bristles, the **bristles** 1–4 mm long, plumose, deciduous; ***pedicels*** 0.1–0.2 mm long, arising just above the bristles. ***Spikelets*** 2–3 mm long, stramineous to mottled with gray areas, subtended by an involucre of deciduous bristles; ***glumes*** 1–3 mm long, narrowly awl-shaped, 1-veined, plumose, difficult to separate from the bristles; ***lemmas*** (1.8–)2–3 mm long, lanceolate, scabrous, 3-veined, usually awned, the awns (1–)5–25 mm long, straight or flexuous, callus hairy, the hairs 0.2–0.4 mm long; ***paleas*** (1.8–)2–3 mm long, as long as the lemmas, apex bidentate; ***stamens*** 3, ***anthers*** 0.6–1.4 mm long, yellow or purplish; **styles** 2. ***Caryopses*** about 0.8 mm long, ovoid. 2*n* = 40 (Reeder 1968).

##### Distribution.

*Muhlenbergiaplumiseta* ranges from northwestern México to Guatemala and El Salvador (Peterson et al. 2001).

##### Ecology.

This species occurs on open hillsides, ravines, and margins of forests in tropical deciduous forests and pine forests in shaded, often dry sites; 700–1800 m.

##### Comments.

*Muhlenbergiaplumiseta* can be separated from *M.pereilema* in having deciduous, plumose bristles (bristles not plumose in *M.Pereilema*) subtending the spikelets and spiciform panicles (2–)3–8 cm long, 0.2–0.6 cm wide (10–22 cm long, 1–3 cm wide in *M.Pereilema*) [Herrera Arrieta et al. 2010). This species forms a clade with *M.Beyrichiana* and *M.Pereilema* within M.Subg.Muhlenbergia (Peterson et al. 2021).

##### Specimens examined.

Guatemala. **Huehuetenango**: Rio Selgua, from wooded slopes above Río Selegua, *W.E. Harmon & J.D. Fuentes 4807* (MO). Mexico. **Chiapas**: **Chiapa de Corzo**: above El Chorreadero, *D.E. Breedlove 53812* (CHAPA). **Chicoasén**: Mirador “Manos que imploran”, 10 km al SW de Chicoasén, *A. Reyes G. 911* (MEXU). **Jitotol**: 3 km NE de Jitotol, en la carr. 195, *F.W. Gould 12707* (ENCB, MEXU, US). **Ocozocoautla de Espinosa**: 14.8 mi de Ocozocoautla, en la carr. Méx., *J. Brunker & C. Perino 314* (ENCB). **Tenejapa**: Paraje de Kotol Te´, *D.E. Breedlove 7363* (ENCB, US). **Tuxtla Gutiérrez**: 2 mi S of Tuxtla Gutiérrez along road to Villa Flores, *D.E. Breedlove & P.H. Raven 13334* (US); 11 km al NE de Tuxtla Gutierrez, Cañon del Sumidero, *R. Torres*, *E. Cabrera & M. Huft 6348* (MEXU); 17 km al NE de Tuxtla Gutierrez, Cañon del Sumidero, *R. Torres*, *E. Cabrera & M. Huft 6405* (MEXU). **Zinacantán**: along Mexican Hwy 190 at paraje Sequentic, *D.E. Breedlove 28688* (MEXU).

#### ﻿26. Muhlenbergia quadridentata

Taxon classificationPlantaePoalesPoaceae

(Kunth) Trin., Gram. Unifl. Sesquifl. 194, t. 5b, f. 14. 1824.

74C83480-FA65-52E4-9BB6-89344E5C8E30

Fig. 15A–E

Podosemum quadridentatum Kunth, Nov. Gen. Sp. (quarto ed.) 1:130–131. 1816. Type: México, México, near Toluca, Sep, *F.W.H.A. Humboldt & A.J.A. Bonpland* s.n. (lectotype: P-BONPL!, designated by McVaugh p. 253. 1983; isolectotypes: GH, US-2557456!, US-86634 fragm. ex P!, US-86635!). ≡ Agrostisquadridata (Kunth) Spreng., Syst. Veg. [Sprengel] 1: 263. 1825 (1824). ≡ Muhlenbergiaquadridentata (Kunth) Kunth, Révis. Gramin. 1:64. 1829, isonym. ≡ Trichochloaquadridentata (Kunth) Roem. & Schult., Syst. Veg. 2:388. 1817. ≡ Muhlenbergiavirescenssubsp.quadridentata (Kunth) Y. Herrera, Amer. J. Bot. 81(8):1043. 1994. Basionym.
= Podosemum gracile Kunth, Nov. Gen. Sp. (quarto ed.) 1:131–132. 1816. Type: México, Michoacán, Volcán de Jorullo, Sep, *F.W.H.A. Humboldt & A.J.A. Bonpland s.n.* (holotype: P-BONPL!; isotypes: LE-TRIN-1501.02!, US-86636 fragm. ex P-BONPL!). ≡ Muhlenbergiagracilis (Kunth) Trin., Gram. Unifl. Sesquifl. 193, t. 5a, f. 6. 1824. ≡ Trichochloagracilis (Kunth) Roem. & Schult., Syst. Veg. (ed. 15 bis) 2: 389. 1817. ≡ Muhlenbergiagracilis (Kunth) Kunth, Révis. Gramin. 1:64. 1829, isonym.

##### Description.

Caespitose ***perennials*** with short, stout rhizomes. ***Culms*** 20–70 cm tall, erect, mostly glabrous below the nodes, the nodes basal, flattened, 1 node per culm; ***internodes*** mostly scabrous. ***Leaf sheaths*** 10–30 cm long, shorter than the internodes, scabrous to smooth; **basal *sheaths*** densely pubescent to glabrous abaxially, smooth and shiny adaxially, becoming flattened and usually not spirally twisted with age; ***ligules*** 2–8 mm long, membranous to hyaline above, firm and often brownish with evident veins near the margins below, decurrent, apex acuminate often lacerate; ***blades*** 5–15 cm long, 0.6–2 mm wide, flat or usually tightly involute, scaberulous below, short-spiculate and often villous above, the hairs 0.2–0.5 mm long, usually appressed, the spicules shiny to whitish. ***Panicles*** 5–20 cm long, 0.5–2 cm wide, narrow, loosely-contracted, interrupted below, mostly plumbeous; **central axis** flattened with 2 ribs, scabrous; ***primary branches*** 0.5–5(–6) cm long, appressed and ascending to spreading up to 30° from the rachises; ***pedicels*** 0.5–2 mm long, shorter than the spikelets, scabrous. ***Spikelets*** 3.4–4.7 mm long, mostly plumbeous; ***glumes*** 1.8–4 mm long, shorter to almost as long as the floret, unequal, mostly greenish-plumbeous, scabrous, usually with a few short hairs below; ***lower glumes*** 1.8–2.5 (–3) mm long, 1-veined, apex obtuse to acute, often with 2 small teeth; ***upper glumes*** (3–)3.2–4 mm long, 3-veined, apex truncate, obtuse or acute, often with 3 or 4 small teeth, the teeth less than 1/6 the length of the glumes; ***lemmas*** 3–4.7 mm long, lanceolate, terete, usually awned, greenish-plumbeous to mottled-plumbeous, sparsely pilose near base and margins on lower ½, apex acuminate, scabrous, the awns 1–20 mm long, flexuous, scabrous, greenish-plumbeous; ***paleas*** 2.8–4.3 mm long, shorter than the lemma, pilose on the proximal ½; ***anthers*** 1–2.5 mm long, purple. ***Caryopses*** 1.8–2 mm long, fusiform, brownish. 2*n* = 20.

##### Distribution.

This species is found throughout México in the higher mountains extending to Guatemala (Peterson et al. 2001).

##### Ecology.

*Muhlenbergiaquadridentata* occurs on open to forested slopes derived from calcareous and volcanic rocks, and is associated with *Pinus* ssp., *Abies* sp., *Holodiscusdiscolor* (Pursh) Maxim., *Populustremuloides* Michx., *Pseudostuga*, and *Quercus* ssp.; (1900–) 2500–4100 m.

##### Comments.

The distinction between *M.quadridentata* and *M.virescens* (Kunth) Kunth is minimal and it has been suggested that quite possibly they represent different morphological forms of a single species corresponding to different habitats (McVaugh 1983). Generally, the plumbeous spikeleted forms (*M.quadridentata*) are found above 2500 m whereas the whitish-hyaline to grayish-green forms (*M.virescens*) are found between 1600–2700 m. Even this color distinction can break down since intermediate individuals are not uncommon. One character that seems to be fairly consistent within each species is the presence of hairs at the base of the glumes. In addition to having dull, scabrous glumes, most individuals of *M.quadridentata* have a few short hairs near the base, whereas individuals of *M.virescens* have whitish or stramineous glumes that are glabrous and shiny near the base. Usually the upper glume apices of *M.quadridentata* are truncate with 3 or 4 teeth whereas the glume apices of *M.virescens* are acute and entire (Herrera Arrieta and Peterson 2018).

*Muhlenbergiaquadridentata* is a member of M.subg.Clomena and it pairs with *M.flabellata* in a recent DNA molecular sequence analysis (Peterson et al. 2021). However, *M.virescens* aligns in a clade with *M.montana*, *M.straminea* Hitchc., and *M.curvula* Swallen [the latter two species treated as synonyms of *M.virescens* in Herrera Arrieta and Peterson (2018)].

##### Specimens examined.

Guatemala. **Huehuetenango**: San Juan Ixcoy, Ul-xemal, *M. Véliz 98.689* (MO); Pine-Juniperus woodland near Tojquia, summit of Sierra de los Cuchumatanes, *J.A. Steyermark 50229* (US); Sierra de los Cuchumatanes, between Tojiah and Chemal at km 319.5 on Ruta Nacional 9N, in grassy meadow, *J.H. Beaman 3874* (US). Mexico. **Chiapas**: **Cintalapa**: 1 km al E de Rizo de Oro, *P. Dávila s.n.* (MEXU).

#### ﻿27. Muhlenbergia ramulosa

Taxon classificationPlantaePoalesPoaceae

(Kunth) Swallen, Contr. U.S. Natl. Herb. 29(4): 205. 1947.

E92A8C8E-D135-5079-B1EB-8C6517F979F8

Fig. 19A–D

Vilfa ramulosa Kunth, Nov. Gen. & Sp. 1: 137. 1815. Type: México, Jorulla, *F.W.H.A. Humboldt & A.J.A. Bonpland s.n.* (lectotype: P-HBK! designated in Peterson and Annable, Syst. Bot. Monographs 31: 77. 1991; isolectotypes: B-WILLD!, US! fragm ex P). ≡ Sporobolusramulosus (Kunth) Kunth, Rev. Gram. 1: 68. 1829. Basionym.
= Sporobolus wolfii Vasey, Bull. Torrey Bot. Club 10: 52. 1883. Type: U.S.A., Colorado, Twin Lakes, 1873, *J. Wolf 1077* (holotype: US!; isotypes: MO!, NY!, US!). ≡ Muhlenbergiawolfii (Vasey) Rydberg, Bull. Torrey Bot. Club 32: 600. 1905.

##### Description.

***Annuals***; delicate, slender, often purplish. ***Culms*** (3–)5–25 cm tall, erect or spreading, geniculate, branched at the base, glabrous to minutely scaberulous below the nodes, branching below, striate, 0.2–0.3 mm in diameter just below the inflorescence; internodes 3–40 mm long. ***Leaf sheaths*** 3–30 mm long, usually shorter than the internodes, glabrous to minutely scaberulous, margins hyaline, scaberulous; ***ligules*** 0.2–0.5 mm long, hyaline, apex truncate, ciliate, without lateral lobes (auricles), margins entire; ***blades*** 5–30 cm long, 0.8–1.2 mm wide, involute or flat, minutely puberulent above, glabrous below. ***Panicles*** (1–)2–9 cm long, 0.6–2.7 cm wide, ovoid or deltoid, sparsely flowered; ***primary branches*** (0.5–)1–3.2 cm long, ascending to spreading (open) or closely appressed; ***pedicels*** 1–3 mm long, glabrous or scabrous, rigid. ***Spikelets*** 0.8–1.3 mm long, erect; ***glumes*** 0.4–0.7 mm long, equal, 1-veined, glabrous, whitish, obtuse or subacute, awnless; ***lemmas*** 0.8–1.3 mm long, oval, plump, with mottled with dark greenish-black areas and greenish-white or ochroleucous areas, inflated at maturity, glabrous or appressed-pubescent on margins and midvein, apex acute, awnless; ***paleas*** 0.7–1.3 mm long, oval; ***anthers*** 0.2–0.3 mm long, purplish. ***Caryopses*** 0.5–1 mm long, ellipsoid, brownish to purplish. *n* = 10.

**Figure 19.**
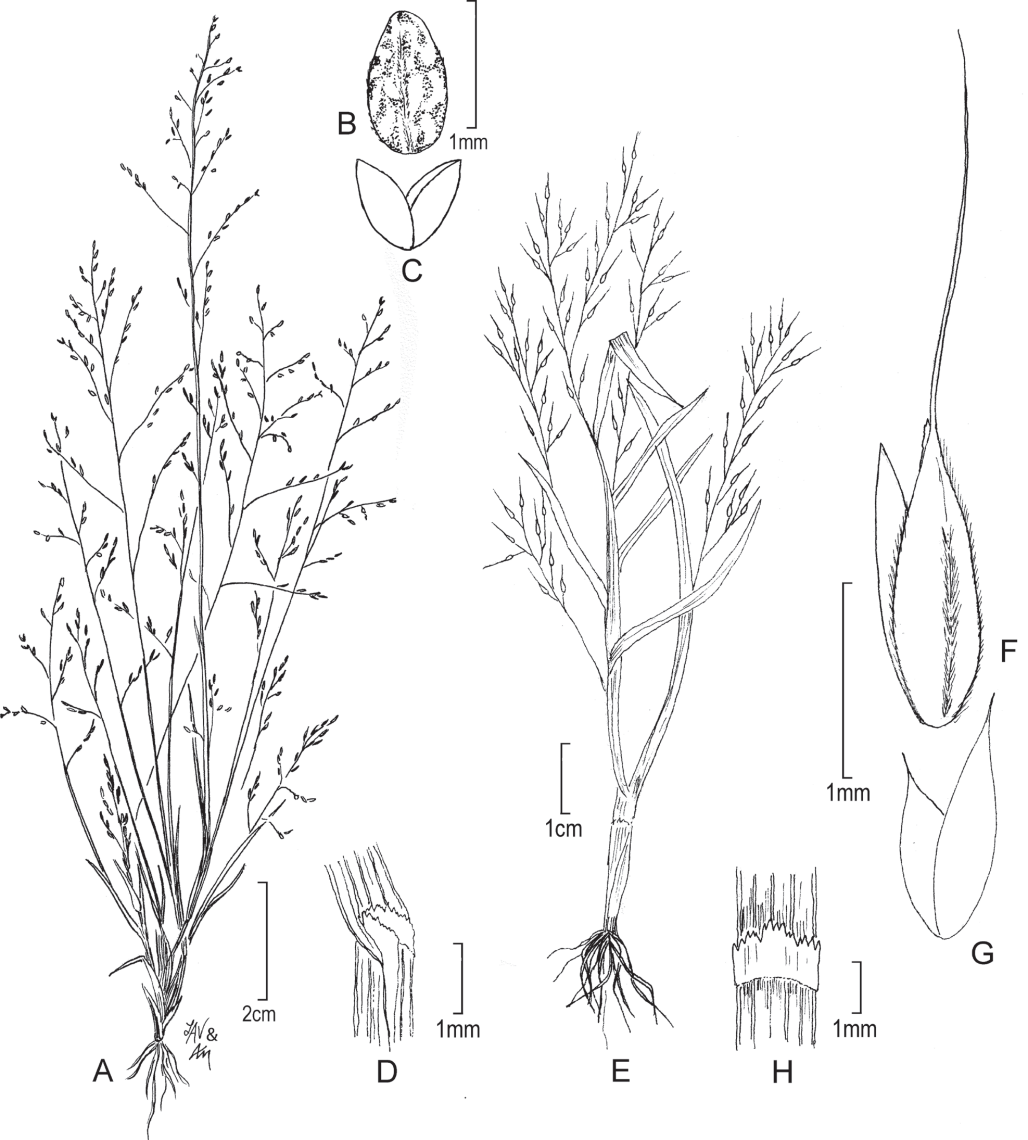
**A–D***Muhlenbergiaramulosa* (Kunth) Swallen **A** habit **B** floret **C** glumes **D** ligule **E–H***Muhlenbergiatenuissima* (J. Presl) Kunth **E** habit **F** floret **G** glumes **H** ligule. **A** drawn from *P.M. Peterson & C.R. Annable 5602* (US-3182911) **B–D** drawn from *P.M. Peterson & C.R. Annable 4661* (US, WS) **E–H** drawn from *P.M. Peterson & C.R. Annable 4751* (US, WS).

##### Distribution.

*Muhlenbergiaramulosa* ranges from the southwestern United States, México, Central America (Guatemala and Costa Rica), and Argentina (Peterson and Annable 1991).

##### Ecology.

It occurs in open pine-oak and tropical forests; 1620–3400 m.

##### Comments.

*Muhlenbergiaramulosa* does not align within the five existing subgenera but is instead sister to the common ancestor shared between the well supported clades of all species in M.subg.Bealia and in M.subg.Trichochloa (Peterson et al. 2021). We place *M.ramulosa* in a separate subgenus, M.subg.Ramulosae P.M. Peterson below.

##### Specimens examined.

Costa Rica. **Cartago**: Oreamuno, Volcán Iraza, devastated area at end of Park Road above Sanitario Duran, growing on ash *R.W. Pohl 14209* (MO); Cordillera Central, lower slopes of Volcan Irazu, 1 km below San Juan de Chicoa, *R.W. Pohl & G. Davidse 11417* (US, CR); Cráter del Volcan Irazú, *O. Jimenez 1151* (US, CR). Guatemala. **Chimaltenango**: Chichavac, *A.F. Skutch 665* (US). **Huehuetenango**: Santa Eulalia, Sierra de los Cuchumatanes, 13 mi NW of Santa Eulalia on road to San Mateo Ixtatán, moist meadow, associates: *Sibbaldia* and *Deschampsia*, *P.M. Peterson & C.R. Annable 4693* (GH, MO, NY, US, WS); meadow at Tojiah on Hwy 9N, *P.M. Peterson & C.R. Annable 4696* (NY, US. WS); 3mi SW of Tojiah on Hwy 9N, *P.M. Peterson & C.R. Annable 4699* (NY, US, WS); Todos Santos Cuchumatan, cerca de la Torre, *J.R. Gálvez et al. 96.5812* (MO); between Tojiah and Chemal at km 319.5 on Ruta Nacional 9N, *J.H. Beaman 3876* (US). **Quetzaltenango**: Cerro Calel, *M. de Koninck* 153 (US). **Sacatepequez**: Volcán de Acatenango, *M. Véliz et al. 10292* (MEXU, MO); Volcano de Agua, *A.S. Hitchcock 9125* (US). Mexico. **Chiapas**: NW de Motzintla de Mendoza on road to El Porvenir, *P.M. Peterson & C.R. Annable 4711* (US, MO); 13 mi NW of Motozintla de Mendoza off road to El Porvenir, *P.M. Peterson & C.R. Annable 4715* (GH, MICH, MO, NY, RSA, UC, US, WS). Sierra Madre, *C.G. Pringle 1425* (MEXU).

#### ﻿28. Muhlenbergia rigida

Taxon classificationPlantaePoalesPoaceae

(Kunth) Kunth, Révis. Gramin. 1: 63. 1829.

D1304184-E2B8-5E13-AF90-91DD0860632F

Fig. 13A–G

Podosemum rigidum Kunth, Nov. Gen. Sp. (quarto ed.) 1: 129. 1816. Type: México, Guanajuato, near Guanajuato, Sep, *F.W.H.A. Humboldt & A.J.A. Bonpland s.n.* (holotype: P!; isotypes: BAA!, US-91920 fragm. ex P!). ≡ Trichochloarigida (Kunth) Roem. & Schult., Syst. Veg. 2: 386. 1817. ≡ Agrostisrigida (Kunth) Spreng., Syst. Veg. 1: 262. 1825. Basionym.
= Podosemum elegans Kunth, Nov. Gen. Sp. (quarto ed.) 1: 130. 1816. Type: Ecuador, Chimborazo, Paramo de las Puntas & Pomallacta, Jun, *F.W.H.A. Humboldt & A.J.A. Bonpland s.n.* (holotype: P!; isotype: BAA!). ≡ Trichochloaelegans (Kunth) Roem. & Schult., Syst. Veg. 2: 387. 1817. ≡ Agrostisquitensis Spreng., Syst. Veg. 1: 262. 1825.
= Podosemum glabratum Kunth, Nov. Gen. Sp. (quarto ed.) 1: 130. 1816. Type: México, Santa Rosa de la Sierra and Cañada de Acabuca, Sep, *F.W.H.A. Humboldt & A.J.A. Bonpland s.n.* (holotype: P-Bonpl!; isotype: US-91921 fragm. ex P-Bonpl!). ≡ Trichochloaglabrata (Kunth) Roem. & Schult., Syst. Veg. 2: 387. 1817. ≡ Agrostisglabrata (Kunth) Spreng., Syst. Veg. 1: 262. 1825.
= Muhlenbergia berlandieri Trin., Mém. Acad. Imp. Sci. Saint-Pétersbourg, Sér. 6, Sci. Math., Seconde Pt. Sci. Nat. 6,4(3-4): 299. 1841. Type: México, Distrito Federal, Mountains near México, Aug 1827, *J.L. Berlandier 676*, *684* (lectotype: LE-TRIN-1487.01! designated by Peterson et al. in PhytoKeys 114: 195. 2018; isolectotypes [all *Berlandier 676*]: COL-000006382 [image!]; P-00644117 [image!], P-00644119 [image!], US-2557457!, US-87241 fragm!, W-239604!, W-0029177 [image!]).
= Muhlenbergia affinis Trin., Mém. Acad. Imp. Sci. Saint-Pétersbourg, Sér. 6, Sci. Math., Seconde Pt. Sci. Nat. 6,4(3-4): 301. 1841. Type: México, México, Toluca, *J.L. Berlandier 1083* (lectotype: P-00644141 [image!] designated by Peterson et al. in PhytoKeys 114: 195. 2018; isolectotypes: G-00099411 [image!], G-00099410 [image!], G-00099409 [image!], LE-TRIN-1485.01 fragm.!, P-00644142 [image!], US-87237 fragm.!). ≡ Podosemumaffine (Trin.) Bush, Amer. Midl. Naturalist 7(2):40. 1921.
= Muhlenbergia phragmitoides Griseb., Abh. Königl. Ges. Wiss. Göttingen 19: 255. 1874. Type: Argentina, Tucumán: Cuesta de Anfama, Sierra de Tucumán, 23 Mar 1872, *P.G. Lorentz 79* (lectotype: GOET-006649 [image!] designated by Peterson et al. in PhytoKeys 114: 195. 2018; isolectotypes: BAA-00002225 [image!], CORD-00004622 [image!], GOET-006648 [image!], SI-002780 [image!], US-91911 fragm. ex GOET!).
= Muhlenbergia elegans var. atroviolacea Kuntze, Revis. Gen. Pl. 3(3): 357. 1898. Type: Bolivia, Cochabamba, 3000 m, 26 Mar 1892, *O. Kuntze s.n.* lectotype: NY-00381485 [image!] designated by Peterson et al. in PhytoKeys 114: 195. 2018.
= Muhlenbergia elegans var. subviridis Kuntze, Revis. Gen. Pl. 3(3): 357. 1898. Type: Bolivia, Tunari Mts, 1600 m, *O. Kuntze* (lectotype: NY-00381486 [image!] designated by Peterson et al. in PhytoKeys 114: 195. 2018).
= Muhlenbergia metcalfei M.E. Jones, Contr. W. Bot. 14: 12. 1912. Type: USA, New Mexico: Grant Co., Santa Rita Mountains, in and around S end of the Black Range, 7000 ft, 9 Oct 1904, *O.B. Metcalf 1485* (holotype: POM-116640!; isotypes: GH-00023980 [image!], MO!, US!).
= Muhlenbergia holwayorum Hitchc., Contr. U.S. Natl. Herb. 24(8): 389. 1927. Type: Bolivia, Sorata, 16 Apr 1920, E.W.D. Holway & M.M. Holway 530 (holotype: US-1108445!).

##### Description.

Densely caespitose ***perennials***. ***Culms*** 40–100 cm tall, stiffly erect, glabrous to scaberulous below the basal, terete nodes, usually 1 node per culm; ***internodes*** mostly glabrous. ***Leaf sheaths*** 2–30 cm long, longer than the internodes, glabrous to scaberulous, rounded near base; ***ligules*** (1–)3–6(–8) mm long, often lacerate, firmer below, strongly decurrent, apex obtuse to acute; ***blades*** 12–35 cm long, 1–3 mm wide, flat or involute, glabrous to scaberulous below and scaberulous to hirsutulous above. ***Panicles*** (4–)10–35 cm long, (2–)3–5(–12) cm wide, loosely contracted to open and lax, sometimes diffuse, reddish-purple; ***primary branches*** 0.4–10 cm long, sometimes capillary, ascending and spreading up to 80° from the rachises; ***pedicels*** 1–10 mm long, mostly longer than the spikelets. ***Spikelets*** 3.5–5 mm long, reddish-purple; ***glumes*** 1–1.7(–2) mm long, much shorter than the floret, about equal, 1-veined, unawned, apex obtuse to subacute, sometimes hirsutulous, rarely mucronate; ***lemmas*** 3.5–5 mm long, narrow lanceolate, scaberulous to scabrous, purple, awned, callus with hairs up to 0.5 mm long, apex acuminate, the awns (8–)10–22 mm long, flexuous; ***paleas*** 3.5–5 mm long, narrow lanceolate, purple, scaberulous, apex acuminate; ***anthers*** 1.7–2.3 mm long, reddish-purple. ***Caryopses*** 2–3.5 mm long, fusiform, brownish. 2*n* = 40, 44.

##### Distribution.

*Muhlenbergiarigida* ranges from Arizona, New Mexico, and southwestern Texas, throughout México, Guatemala, and Honduras to South America where it occurs along the Andes from Columbia, Venezuela, Ecuador, Bolivia, Peru, and Argentina (Peterson et al. 2001).

##### Ecology.

This species occurs on rocky slopes, ravines, and sandy, gravelly slopes derived from granitic and calcareous substrates associated with *Acacia*, *Agave*, *Aristida*, *A.adscensionis*, *Baccharis*, *Berberis*, *Bidens*, *Boutelouacurtipendula* (Michx.) Torr., *Caesalpinia*, *Colletiaspinosissima*, *Cortaderiabifida*, *C.jubata*, *Desmodium*, *Dodonaeaviscosa*, *Ephedra*, *Eragrostis*, *Eucalyptis*, *Eupatorium*, *Festuca*, *Fucaria*, *Hypericum*, *Jarava*, *Krameria*, *Lepechinia*, *Lupinus*, *Lycium*, *Melinusminutiflora*, *Mirabilis*, *Opuntia*, *Paspalum*, *Cenchrusclandestinus*, *Peperomia*, *Puya*, *Salvia*, *Schinusmolle* L., *Schizachyrium*, *Sporobolus*, *Tillandsia*, and *Trichocereus*; 2000–3650 m.

##### Comments.

This species is highly variable and is one of the most common upland bunchgrasses forming almost pure stands in northern México, less common in Peru and South America where it is usually found in smaller populations.

Molecular DNA sequence analysis indicates *M.rigida* lies within Muhlenbergiasubg.Trichochloa and genetically is highly variable (Peterson et al. 2010b; Peterson et al. 2021).

##### Specimens examined.

Guatemala. **Huehuetenango**: upper and off canyon near Huehuetenango, *A.A. Beetle 6* (MEXU). Honduras. **Francisco Morazán**: along road 13 km W of Mateo, *R.W. Pohl 12737* (CR). Mexico. **Chiapas**: **Ococingo**: a 4 km al S de Ejido Benemérito de las Américas camino a Flor de Cacao, *E. Martínez 10785* (ENCB); Arroyo del Rancho Pellizzi, al E de San Cristóbal, *A. Méndez 9158* (CIIDIR, MEXU). **San Cristóbal de las Casas**: 7 km E of San Cristóbal de las Casas, along the road to Zontehuitz, *D.E. Breedlove 11155* (ENCB); NE edge of San Cristobal de las Casas, *D.E. Breedlove 53858* (NY); Northeast edge of San Cristóbal Las Casas, *D.E. Breedlove & G. Davidse 54745* (CAS, MO); East side of San Cristóbal Las Casas, *D.E. Breedlove & G. Davidse 52327* (CAS, MO). **San Fernando**: Sobre cerro ubicado al E de la subestación de CFE, entrada por la zona de extración de arena, carretera Tuxtla-San Fernando, *A. López C. 1359* (HEM, MO). **Tenejapa**: barrio of Yashanal, paraje of Mastab, steep slope along the river of Chik Ha’, *D.E. Breedlove 11125* (ENCB). **Teopisca**: about 15 mi SE of Teopsca on a heavily grazed slope, *J.R. Reeder & C.G. Reeder 2029* (ENCB); S edge of Teopisca, *D.E. Breedlove & P.H. Raven 13097* (ENCB). **Zinacantan**: near paraje Nachij, *D.E. Breedlove 54703* (NY).

#### ﻿29. Muhlenbergia robusta

Taxon classificationPlantaePoalesPoaceae

(E. Fourn.) Hitchc., N. Amer. Fl. 17(6): 462. 1935.

7D743CC1-EF7B-5C91-B619-00A8C0E874D3

Fig. 20A–E

Epicampes robusta E. Fourn., Mexic. Pl. 2:89. 1886. Type: México, Distrito Federal, Santa Fe, 2 Oct 1865, M. Bourgeau 1153 (lectotype: P!, designated by Hitchcock, N. Amer. Fl. 17(6): 462. 1935; isolectotypes: K!, US-999036!, US-999031! fragm., US-90734! fragm.). Basionym.
= Epicampes stricta J. Presl, Reliq. Haenk. 1(4-5):235, t. 39. 1830. Type: México, *T. Haenke s.n.* (holotype: PRC?; isotypes: LE-TRIN-1558.01! fragm., US-865970! fragm.). ≡ Muhlenbergiapresliana Hitchc., N. Amer. Fl. 17(6): 462. 1935b, nom. nov.
= Epicampes berlandieri E. Fourn., Mexic. Pl. 2:89. 1886. Type: México, México, Feb 1839, *J.L. Berlandier 670* (lectotype: P! designated by Hitchcock, N. Amer. Fl. 17(6): 462. 1935; isolectotypes: BM-000938652 [image!], G-00099371 [image!], US-1127013!). ≡ Muhlenbergiafournieriana Hitchc., J. Wash. Acad. Sci. 23(10): 453. 1933.
= Epicampes macrotis Piper, Proc. Biol. Soc. Wash. 18:144. 1905. Type: México, Zacatecas, Sierra Madre Mountains, ca. 40 km W of San Juan Capistrano, 7 Aug 1897, *J.N. Rose 3528* (holotype: US-302505!). ≡ Muhlenbergiamacrotis (Piper) Hitchc., N. Amer. Fl. 17(6): 463. 1935.
= Epicampes minutiflora Mez, Repert. Spec. Nov. Regni Veg. 17:212. 1921. Type: México, Michoacán, near El Canizal, 600m, 15 Jan 1899, *E. Langlassé 750* (isotype: US-386160!). ≡ Muhlenbergiameziana Hitchc., N. Amer. Fl. 17(6): 461. 1935, nom. nov.

##### Description.

Caespitose ***perennials***. ***Culms*** 100–230(–300) cm tall, erect, compressed-keeled near base, glabrous to sometimes pubescent below the nodes; ***internodes*** glabrous. ***Leaf sheaths*** 15–70 cm long, longer than the internodes, glabrous, becoming brownish below, sometimes shredded; ***sheath auricles*** present, (1–)2–4(–10) cm long, linear subulate to broadly triangular, longer above, straight or twisted, firm below; ***ligules*** 2–10(–12) mm long, membranous, lacerate throughout; ***blades*** 40–100 cm long, 4–7 mm wide, folded sometimes involute toward tip, scaberulous above and below, the margins and keel saw-toothed. ***Panicles*** 30–80 cm long, (2–)3–8 cm wide, narrow to loosely contracted, greenish-gray to silvery gray or purplish; ***primary branches*** 1–15(–17) cm long, naked near base, ascending and closely appressed to spreading up to 40° from the rachises; ***pedicels*** 0.3–1.1 mm long, shorter than the spikelets, erect, scaberulous; central axis prominently ribbed, scabrous. ***Spikelets*** (1.8–)2–3 (–3.2) mm long, erect, greenish-gray or purplish; ***glumes*** 1.8–3.2 mm long, usually longer than the floret, subequal, narrowly oblong to elliptic, veinless to indistinctly 1-veined, hyaline to greenish-gray, glabrous to scaberulous, apex acute to obtuse occasionally erose; ***lemmas*** 1.7–2.6 mm long, linear-oblong, unawned or rarely mucronate, greenish to yellowish-brown, glabrous or pubescent with scattered hairs on lower, the hairs up to 0.3 mm long, ***callus*** glabrous or with few hairs, apex acute, the mucro when present up to 1 mm long; ***paleas*** 1.7–2.6 mm long, glabrous to sparingly pilose between the veins on lower, apex acute; ***anthers*** 1.1–2 mm long, purplish. ***Caryopses*** 1.2–1.7 mm long, fusiform, brownish. 2*n* = 40.

**Figure 20.**
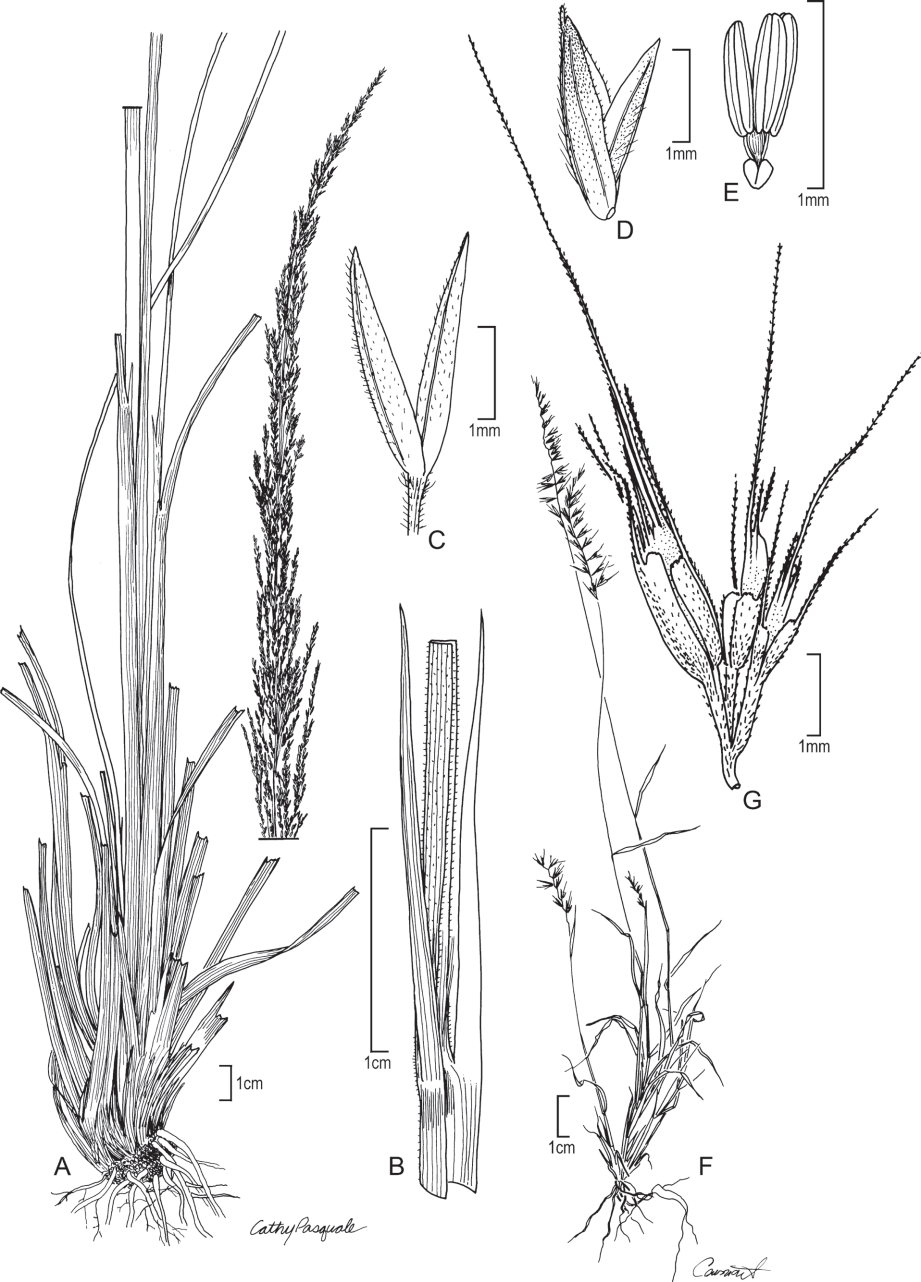
**A–E***Muhlenbergiarobusta* (E. Fourn.) Hitchc. **A** habit **B** ligule **C** glumes **D** floret **E** stamens, pistil, and lodicules **F, G***Muhlenbergiauniseta* (Lag.) Columbus **F** habit **G** primary inflorescence branch with three spikelets. **A–E** drawn from *P.M. Peterson & C.R. Annable 6131* (US) **F, G** drawn from *F.W. Gould 10391* (US-3000113).

##### Distribution.

*Muhlenbergiarobusta* occurs in mountainous areas from Sinaloa and Chihuahua south to Chiapas, Guatemala, Honduras, and Nicaragua in Central America (Peterson et al. 2001).

##### Ecology.

It is found on rocky slopes, along barrancas (canyons), in pine and pine–oak forests, and in tropical deciduous forests; 850–3000 m.

##### Comments.

Morphologically, *M.robusta* is similar to *M.mutica* but can be separated from the latter in having narrow panicles (2–)3–8 cm wide [(8–)15–30 cm wide in *M.mutica*] and auriculate leaf sheaths (not auriculate in *M.mutica*), the auricles 2–4(–10) mm long.

In DNA sequence studies, *Muhlenbergiarobusta* is found to align in a large polytomy with other species in M.subg.Trichochloa (Fig. 1; Peterson et al. 2010b; Peterson et al. 2021).

##### Specimens examined.

Guatemala. **Chimaltenango**: near finca La Alameda, near Chimaltenango, *P.C. Standley 59136* (US); Above Santa Maria, Volcano Agua, *A.S. Hitchcock 9129* (US). **Guatemala**: Guatemala City, 21 km W of Guatemala on CA-1, along road in oak forest, *W.E. Harmon& J.A. Fuentes 4865* (MO); Guatemala City, Eureka, *A.S. Hitchcock 9081* (US); Guatemala City, *A.S. Hitchcock 9035* (US), *9063 (1/2)* (US). **Huehuetenango**: Barranco “Palo Negro” in oak-pine forest about 10 km west of Aguacatan, *L.O. Williams et al. 21844* (US), *21848* (US); km 101 between El Mirador and Chintla, Sierra Cuchumatanes, rocky slopes, *A. Molina 21176* (US). Santa Rosa: Taxisco, Naranjo, *J. Donnell Smith 3932* (US, MO). **Quiche**: Nebaj, NE of Nebaj, *M.J. Metzler 17* (MO). **Sacatepequez**: near Antigua, *P C. Standley 61698* (US); *P.C. Standley 76942* (US). **Santa Rosa**: Taxisco, Naranjo, *Heyde & Lux 3932* (MO). **Sololá**: 5 km W of Patzún, collection from deep ravine covered with pine and oak, *W.E. Harmon & J.D. Dwyer 2646* (MO); Cerca del Lago Atitlán, *M. de Koninck 145* (US). Honduras. **Intibucá**: La Esperanza, Intibucá, Cerro San Cristóbal, bosque mixto premontano húmedo, *T. M. Mejía O. 87* (MO). Mexico. **Chiapas**: Camino de San Cristobal a Ocosingo, 31 km suroeste de Ocosingo, *R. Banda S. et al. 73117* (DS). **Amatenango del Valle**: 14 km SE of Teopisca along hwy. to Comitán, *G. Davidse et al. 29800* (MO). **Angel Albino Corzo**: slopes of Río Cuxtepec below Finca Cuxtepec, *D.E. Breedlove & G. Davidse 54699* (CAS, MO). **Bochil**: 5 km east of Bochil on road to Pichucalco, *D.E. Breedlove & G. Davidse 55164* (CAS, MO). Comitán de Domínguez: 6 km N of Comitán along Mexican hwy 190, 1770 m, *D.E. Breedelove & G. Davidse 54873* (SLPM, CAS, MO); 6 km N of Comitán along Mexican Highway 190, *D.E. Breedlove & G. Davidse 54877* (CAS, MO); 6 km N of Comitán along Mexican Highway 190, *D.E. Breedlove & G. Davidse 54869* (CAS, MO). **Ixtapa**: at Escopetazo, *D.E. Breedlove & G. Davidse 53948* (CAS, MO); along Mexican Highway 190 at the Zinacantán paraje of Muctajoc, *Laughlin 1559* (DS). **La Independencia**: above and SW of La Soledad on road to Las Margaritas, *D.E. Breedlove 53126* (CAS, MO). **La Trinitaria**: 6–7 km S of La Trinitaria, *G. Davidse et al. 29950* (MO); 10 km south of La Trinitaria on Mexican Highway 190, *G. Davidse et al. 55070* (CAS, MO). **Rayón**: 9 miles NW of Pueblo Nuevo Solistahuacá along the road between Rincon Chamula and Rayón, *H. Zuill 609* (DS). near summit of Zontehuitz, *A. Shilom Ton 30* (DS). **Teopizca**: 7 km NW of Teopisca along hwy. to San Cristobal de las Casas, *G. Davidse et al. 29829* (MO); North of Teopisca, *D.E. Breedlove & G. Davidse 54765* (CAS, MO). **Tuxtla Gutiérrez**: 31 km al W de Tuxtla Gtz y 13 km NE de Chiapa de Corzo, 1050 m, *J. C. Soto 13361 con D. Sutton*, *R. Hampshire*, *R. Lira y A. Reyes* (MEXU); km 11.5 de la carr. Tuxtla Gutierrez-Cañon del Sumidero, en el lugar denominado El Zacabastal, *E. Rodríguez s/n* (MEXU); 16 km N of Tuxtla Gutiérrez on road to El Sumidero, *D.E. Breedlove & B.M. Bartholomew 55491* (CAS, MO). **Venustiano Carranza**: 3 miles S of Aguacatenango along the road to Pinola Las Rosas, *D.E. Breedlove & P.H. Raven 13433* (DS). **Villa Corzo**: above Colonia Vincente Guerrero on the road to Finca Cuxtepec, *D.E. Breedlove & G. Davidse 54600* (CAS, MO); near Revolucion Mexicana, *D.E. Breedlove & G. Davidse 54520* (CAS, MO). **Zinacantán**: near Paraje Sequentic [Zequentic], *D.E. Breedlove & G. Davidse 53909* (CAS, MO); Along Mexican Highway 190, 10 miles southeast of the road to Simojovel. Paraje of Granadia, *D. E. Breedlove 7274* (DS); at Kampana Ch’en along Mexican Highway 190, 3 miles west of paraje Navenchauk, *R.M. Laughlin 2279* (DS); along road from Zinacantán center to Ixtapa near Paraje Vo Bits, *D.E. Breedlove 40725* (MO). Nicaragua. **Managua**: Sierra de Managua, *H.A. Garnier 1953* (GH, US).

#### ﻿30. Muhlenbergia setarioides

Taxon classificationPlantaePoalesPoaceae

E. Fourn., Mexic. Pl. 2: 84. 1886.

6FEE910D-34BF-5089-B95E-138DD3861427

Fig. 21F–J

= Muhlenbergia polypogonoides Hack., Ann. K. K. Naturhist. Hofmus. 17: 255. 1902. Type: México, *A. Schmitz 862* (holotype: W-18890124080!; isotypes: US-3412356! fragm. ex W, W-19160029073!

##### Type.

México, Orizaba, Borrego, 14 Nov 1865–1866, *M. Bourgeau 3362* (**lectotype, designated here**: MPU-026952 [image!]; isolectotypes: G-00099164 [image!], GH-0024047 [image!] ex P, K-000308906 [image!], MO-2974294!, US-0091990! ex P & ex LE, S-14-29487 [image!]. ≡ Muhlenbergiasylvaticavar.setarioides (E. Fourn.) Beal, Grass. N. Amer. 2: 249. 1896.

##### Description.

Sprawling ***perennials***, rooting at the lower nodes. ***Culms*** 30–70(–100) cm tall, geniculate, usually glabrous, with many branches, about 1.5 mm thick near base. ***Leaf sheaths*** shorter or longer than internodes, terete, striate, glabrous or scaberulous; ***ligules*** 1.5–3.5 mm long, hyaline, becoming yellowish-brown or brownish, erose; ***blades*** 4–12(–17) cm long, 3–6(–9) mm wide, flat, thin, dark green, scaberulous above, narrowing to the base, central vein whitish, prominent. ***Panicles*** 6–13.5(–15) cm long, 1–2 cm wide, yellowish-brown to greenish, densely flowered, interrupted below; ***primary branches*** 1–3.5 cm long, appressed or ascending, flowered to the base; ***pedicels*** 0.2–1.7 mm long, shorter than the spikelets, hispidulous. ***Spikelets*** (2–)2.5–3 mm long; ***glumes*** 1–2(–2.5) mm long, unequal, 1-veined, green and prominent, apex acute to acuminate; ***lower glumes*** 1–1.5 mm long; ***upper glumes*** 1.5–2(–2.5) mm long; ***lemmas*** (2–)2.5–3 mm long, lanceolate, pale, awned, mottled with dark green spots, the 3-veins green and prominent, scabrous along the veins, pilose over the central vein and margins on the lower 1/3–1/2, the awns 5–14 mm long, somewhat flexuous, purplish; ***paleas*** about as long as the lemma, pilose between the veins on lower ½; ***anthers*** 1–1.2 mm long, yellowish. ***Caryopses*** 1.3–1.5 mm long, narrowly-ellipsoid, dark reddish-brown. 2*n* = 40.

**Figure 21.**
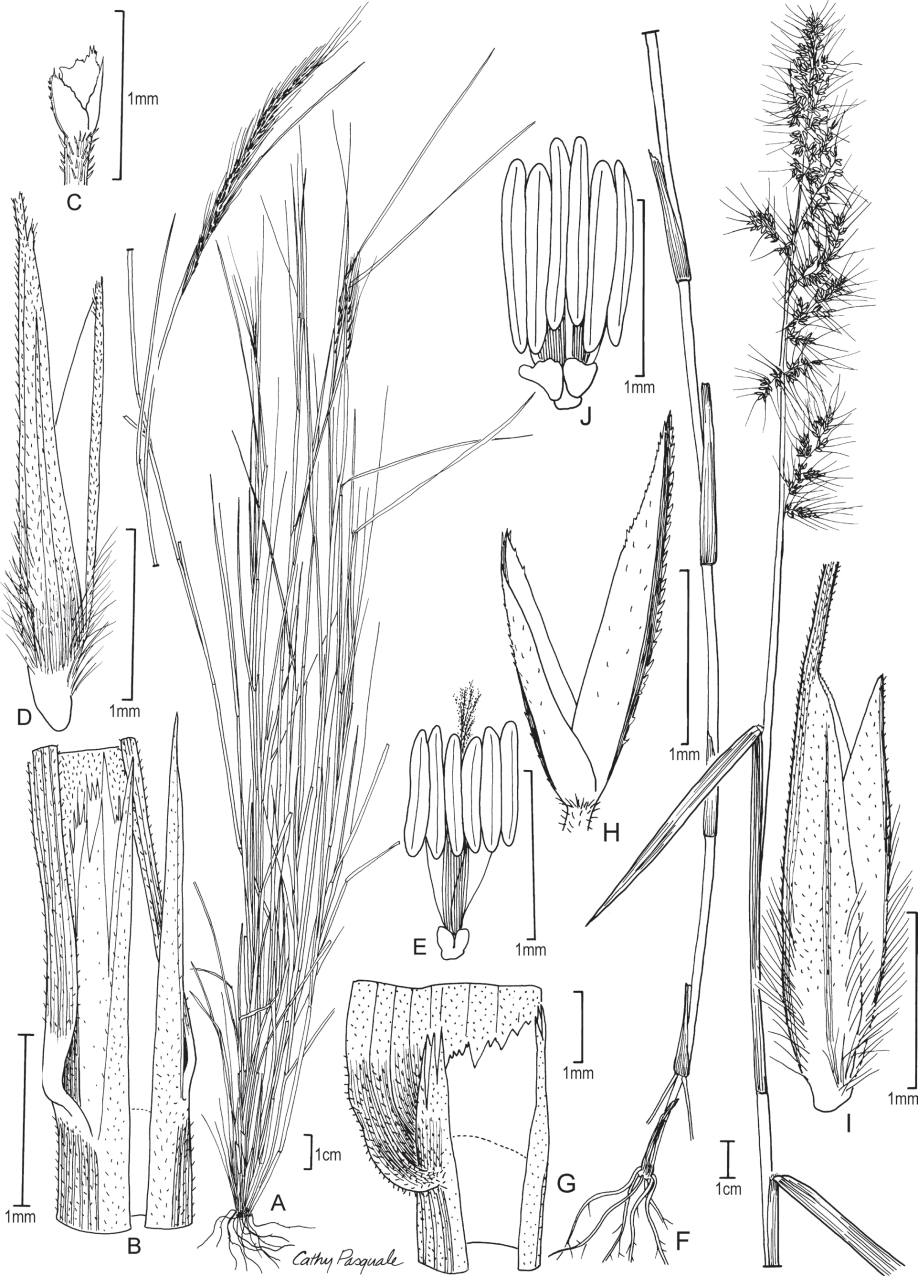
**A–E***Muhlenbergiaspiciformis* Trin. **A** habit **B** ligule **C** glumes **D** floret **E** stamens, pistil, and lodicules **F–J***Muhlenbergiasetarioides* E. Fourn. **F** habit **G** ligule **H** glumes **I** floret **J** stamens, pistil, and lodicules. **A–E** drawn from *P.M. Peterson & C. R. Annable 8361* (US) **F–J** drawn from *D.E. Breedlove 9350* (US).

##### Distribution.

*Muhlenbergiasetarioides* ranges from Puebla, Oaxaca (*Peterson & Annable 9897*, 1.4 mi E of Ayutla on Mex 179), Tlaxcala,Veracruz, and Chiapas in México to Central America from Guatemala to Panama (Sanchez-Ken 2018).

##### Ecology.

This species is found in tropical-wet forests, shaded banks, near cornfields, and along barrancas; 1500–2400 m.

##### Comments.

Morphologically, *M.setarioides* resembles *M.spiciformis* but differs from the latter in having wider leaf blades 3–6(–9) mm wide (1–3 mm wide in *M.spiciformis*), shorter spikelets (2–)2.5–3 mm long (versus 3–4 mm long), acute to acuminate (obtuse to acute in *M.spiciformis*) glumes, upper glumes 1.5–2(–2.5) mm long (versus ≤ 1mm long), and lemmas with awns 5–14 mm long [versus (10–)20–40 mm].

*Muhlenbergiasetarioides* is a member of M.subg.Muhlenbergia and is sister to all remaining species in the subgenus (Fig. 1; Peterson et al. 2021).

##### Specimens examined.

Costa Rica. **Alajuela**: along road below Los Cartagos, 5 km above Carrizal, wet bank of roadside, in shade of herbs, *R.W. Pohl & G. Davidse 11735* (CR, US). **Cartago**: River crossing of Río Reventado between Llano Grande and Tierra Blanca, *R.W. Pohl & M. Lucas 13097* (MO, CR); Cerro de La Carpintera, in potrero, *P.C. Standley 34502* (US). **San José**: along Rio Maria Aguilar, near San José, *P.C. Standley 38976* (US); Vazquez de Coronado, Quebrada Corralillo, 2 km E of Rancho Redondo, moist mossy canyon walls in shade and along roadsides, *R.W. Pohl & G. Davidse 11701* (CR, MO, US); La Palma, wet forest, *P.C. Standley 33006* (US); San Gabriel, orillas de un arroyo, *O. Jiménez 173* (US). El Salvador. **La Libertad**: Volcan San Salvador, near bottom of crater of Volcán San Salvador, *N.C. Fassett 28595* (MO, ITIC, US); Volcan San Salvador, Boquerón de San Salvador, *L.E. González 1852* (MO, ITIC). **San Salvador**: San Salvador, *A.S. Hitchcock 8927* (US). **Sonsonate**: *A. R. Molina et al. 21747* (LAGU). Guatemala. **Escuintla**: San José, *W. A. Kellerman 5115* (MO, US). **Guatemala**: Near San Rafael, *W.A. Kellerman 6239* (US). **Huehuetenango**: Thickets and forest in deep canyon of tributary of Rio Blanco, about 5 km W of Aguacatán, *L.O. Williams*, *A. Molina R. & T.P. Williams 22329* (US). **Quetzaltenango**: Mountains near Santa Maria, just S of Quetzaltenango, *Weatherwax 161 (1679)* (US); Region of Las Nubes, south of San Martin Chile Verde, densely forested barranco, *P.C. Standley 83711* (US); Ravine below Fuentes Georginas, just above Zunil, *J.A. Steyermark 34480* (US); Retalhuleu, *M. De Koninck 226* (US); Mountains above Rio Samala, Sierra Madre Mountains, 2 km west of Zunil, *L. O. Williams*, *A. Molina R. & T.P. Williams 22987* (US). **Sacatepequez**: near Pastores, damp ravine, *P.C. Standley 59936* (US); Volcano Agua, near Antigua, shady bank, *A.S. Hitchcock 9134* (US); Nacimiento del Cangrejal, cuesta de Las Cañas, *A. Molina R. 15439* (US). **San Marcos**: Barrancos 6 mi south and west of town of Tajumulco, northwestern slopes of Volcan Tajumulco, moist thickets in quebrada, *J.A. Steyermark 36594* (US); Barranco Eminencia, above San Rafael Pie de la Cuesta, wet meadow, *P.C. Standley 68464* (US). **Solola**: Mixed forest area, mountains slopes above Lake Atitlan, about 3–5 km west of Panajachel, *L.O. Williams*, *A. Molina R. & T.P. Williams 25264* (US). Mexico. **Chiapas. Motozintla**: along road from Toilman to Niquivil, near Ojo de Agua, *D.E. Breedlove 42616* (MO). **Siltepec**: On the ridge above Siltepec on the road to Huixtla, *D.E. Breedlove & Almeda 58258* (CAS, MO). **Tenejapa**: along trail from Tenejapa center to San Cristóbal de las Casas, in the paraje of Balum K’ anal, *D.E. Breedlove 9350* (ENCB, DS, US); La Punta del Cerro Cruz Ch’en, *A. Shilom Méndez Ton 5088* (MEXU, MO). **Unión Juárez**: En el volcán Tacaná a 500 m al E de Talquián, *E.M. Martínez S. & A Reyes-García 20293* (MO). Panama. **Chiriquí**: Rio Caldera, 1–2 mi above El Boquete, *E.P. Killip 4513* (US).

#### ﻿31. Muhlenbergia spiciformis

Taxon classificationPlantaePoalesPoaceae

Trin., Mém. Acad. Imp. Sci. Saint-Pétersbourg. Sér. 6, Sci. Math., Seconde Pt. Sci. Nat. 6,4(3–4): 288. 1841. (Fig. 14, E-I).

3895CF99-9F94-557D-8F57-B133555362A1

Fig. 21A–E

= Muhlenbergia acutijolia E. Fourn., Mexic. PI. 2:86. 1886. Type: México, Veracruz, Orizaba, 8 Nov 1866,M. Bourgeau 3327 (holotype: P!; isotypes: MO-2974301!, US-87235! fragm! US-2561240!).
= Muhlenbergia parviglumis Vasey, Contr. U.S. Natl. Herb. 3(1):71. 1892. Type: U.S.A., Texas, 1887, *G.C. Nealley s.n.* (holotype: US-81638!; isotype: US-994967!).

##### Type.

México, “Southern México,” *Karwinsky s.n.* (lectotype: W-0002567! designated by Peterson et al. 2007b in J. Bot. Res. Inst. Texas 1(2): 989; isolectotype: LE fragm!).

##### Description.

Caespitose ***perennials***, often short-lived and appearing as annuals. ***Culms*** 25–80 cm tall, erect, slender and wiry, freely branching at the base, strigose to glabrous below the nodes; ***internodes*** mostly glabrous, usually 4–8 nodes per culm. ***Leaf sheaths*** 3.5–12 cm long, shorter than the internodes, scaberulous; ***ligules*** 1–3 mm long, deeply lacerate, margins hyaline, apex acuminate; ***blades*** 2–12 cm long, 1–3 mm wide, flat to involute, hirsutulous to scabrous above and scaberulous below. ***Panicles*** 4–18(–20) cm long, (0.6–)l–2.8 cm wide, narrow, contracted, sometimes interrupted below, loosely flowered; ***primary branches*** 0.6–5 cm long, ascending and appressed occasionally spreading up to 30° from the rachises; ***pedicels*** 0.1–3.0 mm long. ***Spikelets*** 2.8–4 mm long, erect; ***glumes*** 0.3–1.0 mm long, less than 1/2 as long as the lemma, 1-veined, unequal, apex obtuse to acute, sometimes erose; lower glumes shorter than the upper glumes; ***lemmas*** 2.8–4 mm long, narrowly lanceolate, awned, purplish, scabrous roughened, sparsely appressed-pubescent on the calluses and lower 1/4 of the midveins and margins, the hairs less than 0.3 mm long, apex acuminate, the awn (10–)20–40 mm long, straight to flexuous; ***paleas*** 2.6–3.9 mm long, narrowly lanceolate, sparsely pubescent between the veins on the basal 1/3, apex acuminate, scabrous; ***anthers*** 0.9–1.6 mm long, purplish. ***Caryopses*** 2–2.6 mm long, fusiform, brownish. 2*n* = 40.

##### Distribution.

This species ranges from the southwestern United States south to México (Chiapas, Chihuahua, Coahuila, Hidalgo, Nuevo León, Oaxaca, Puebla, Queretaro, San Luis Potosí, Tamaulipas, Tlaxcala, Veracruz) [Peterson et al. 2007b; Sanchez-Ken 2018)].

##### Ecology.

*Muhlenbergiaspiciformis* grows on rocky slopes, cliffs, and calcareous rock.

outcrops, often in thorn-scrub and open woodland communities associated with *Quercus* ssp., *Pinus* ssp., *P.cembroides*, *Juniperusdeppeana*, *Pseudotsugamenziesii*, *Abies*, *Cupressus*, *Agave*, *Ceanothus*, *Acacia*, *Salvia*, *Arbutus*, *Opuntia* and *Fraxinus*; 450–2800 m.

##### Comments.

In addition to being morphologically similar to *M.setarioides* (see earlier comments under *M.setarioides*), *M.spiciformis* can be confused with *M.microsperma* but differs in not having cleistogamous spikelets in the axils of the lower culm branches, panicles narrow, contracted, 0.6–2.8 cm wide (1–6.5 cm wide in *M.microsperma*) primary branches spreading up to 30° from the rachises (primary branches spreading up to 80° from the rachises in *M.microsperma*), and ligules acuminate (truncate to obtuse in *M.microsperma*) [Peterson et al. 2007b].

*Muhlenbergiaspiciformis* is a member of M.subg.Muhlenbergia and is sister to the *M.romaschenkoi–M.tenuifolia* pair in a recent biogeographical study based on DNA sequence analysis (Peterson et al. 2021).

##### Specimens examined.

Mexico. **Chiapas: Comitán de Domínguez**: Laguna Chamula microwave station, 4 km SW of Highway 190 between Comitán and Amatenango del Valle, *D.E. Breedlove & G. Davidse 54857* (CAS, MO). **Jilotol**: 10 km N of Jitotol near Rio Hondo, *D.E. Breedlove & G. Davidse 55151* (CAS, MO). **La Trinitaria**: along Mexican Highway 190, 3 miles south of La Trinitaria, *D.E. Breedlove & P.H. Raven 13235* (DS, US). **San Cristobal de las Casas**: NE edge of San Cristobal de las Casas, *D.E. Breedlove & G. Davidse 54748* (NY, CAS, MO); Northeast edge of San Cristóbal Las Casas, *D.E. Breedlove & G. Davidse 54722* (CAS, MO); W edge of San Cristobal Las Casas, *D.E. Breedlove & G. Davidse 53980* (CAS, MO, US); About 2 miles SE of San Cristóbal, *J.R. Reeder & C.G. Reeder 6066* (MO); Grassy slope of Cerro San Cristóbal, *R.M. Laughlin 1761* (DS).

#### ﻿32. Muhlenbergia tenella

Taxon classificationPlantaePoalesPoaceae

(Kunth) Trin., Gram. Unifl. Sesquifl. p. 192. 1824.

C197AC23-BBEF-5310-9A0C-2CD2DE5E34F1

Fig. 17C–F

Podosemum tenellum Kunth, Nov. Gen. Sp. (quarto ed.) 1: 128. 1816. 1817. Type: México,Veracruz, inter Río Frío et Barranca Honda, *F.W.H.A. Humboldt & A.J.A. Bonpland s.n.* (holotype: P-Bonpl!; isotypes P!, US-91922! fragm. ex P). ≡ Trichochloatenella (Kunth) Roem.& Schult., Syst. Veg. 2: 385. 1817. ≡ Polypogontenellus (Kunth) Spreng., Syst. 1: 243. 1825. ≡ Polypogongracilis (Kunth) Spreng., Syst. 5: 558 (index).1828. Basionym:
= Muhlenbergia sprengelii Trin., Gram. Unifl. Sesquilfl. 189. 1824. Type: México, *F.W.H.A. Humboldt & A.J.A. Bonpland s.n*. (holotype: B-W!). ≡ Arundotenella Spreng., Pl. Min. Cogn. Pug. 2: 6. 1815, *nom. Illeg. hom*.
= Muhlenbergia exilis E. Fourn., Mexic. Pl. 2: 84. 1886. Type: México, Barranca près Cuernavaca, Iturbide, 14 Nov 1865, *E. Bourgeau 1298* (**lectotype, designated here**: P-00644178 [image!]; isolectotypes: G-00099415 [image!], MPU-026954 [image!], P-00644177 [image!], US-87216!).

##### Description.

Slender, delicate ***annuals***, sometimes in small tufts. ***Culms*** 10–30 cm tall, erect or sprawling, glabrous, branching from the lower and middle nodes; 0.3–0.4 mm diameter just below the inflorescence; ***internodes*** 17–36 mm long. ***Leaf sheaths*** 12–37 mm long, mostly shorter than the internodes, glabrous or sparsely pilose especially near the apex and along the margins; ***ligules*** 0.3–0.9 mm long, a ciliate membrane; apex truncate; margin with a tuft of hairs up to 1 mm long; ***blades*** 2–5 cm long, 0.5–2.0 mm wide, flat, often secund or lying to one side of the culm, sparsely appressed pilose pubescent on both surfaces to almost glabrous. ***Panicles*** 3.0–12.6 cm long, 0.5–2.5 cm wide, slender, usually included in the upper sheath, terminal, with 7–12 nodes; ***primary branches*** 2–4.8 mm long, 1 per node, ascending and appressed to the culm axis, bearing spikelets to their base; ***pedicels*** 1–3 mm long, glabrous, to minutely scaberulous, appressed. ***Spikelets*** 1.7–2.7 mm long, overlapping, erect; ***glumes*** 0.5–1.8 mm long, unequal, mostly glabrous, 1-veined, the single greenish vein antrorsely scabrous; apex acute to acuminate, sometimes minutely pubescent, usually mucronate, the mucro up to 1 mm long; ***lower* glume** 0.5–1.3 mm long; ***upper* glume** 0.7–1.8 mm long, narrower; ***lemmas*** 1.7–2.7 mm long, slender, narrow lanceolate, awned, whitish, strongly 3-veined, veins greenish, the appearance of intermediate “veins” actually rows of short barbs on top of folded epidermal ridges, occasionally ciliate on the lateral veins on upper part but mostly glabrous, the awns 12–26 mm long, flexuous; ***callus*** minutely short pubescent; ***paleas*** 1.8–2.8 mm long, a little longer than the lemma, narrow lanceolate, glabrous to minutely antrorsely scabrous; ***anthers*** 0.4–0.5 mm long, yellowish. ***Caryopses*** 1.4–1.6 mm long, narrowly fusiform, light brownish. Cleistogamous spikelets absent. 2*n* = 20.

##### Distribution.

*Muhlenbergiatenella* occurs throughout México ranging to Central America (Belize, Costa Rica, El Salvador, Guatemala, Honduras, Nicaragua, and Panama) and extending into Columbia (Peterson and Annable 1991; Peterson et al. 2001).

##### Ecology.

Individuals of *M.tenella* are usually restricted to perennial wet rocky cliffs, rock walls, and sandy or rocky places along water courses in tropical and subtropical forests and moist pine-oak woodlands; 250–2400 m.

##### Comments.

*Muhlenbergiatenella* can be separated from *M.ciliata* in having long-awned [12–26 mm long versus (1–)5–11(–18) mm long in the latter), mostly eciliate lemmas, appressed panicle branches, secund leaf blade insertion, and its affinity for perennially wet rocky cliffs and rock walls associated with small drainages (Peterson and Annable 1991).

Based on DNA sequence analyses *Muhlenbergiatenella* forms a clade with *M.ciliata* and *M.pectinata* in M.subg.Muhlenbergia (Fig. 1; Peterson et al. 2010b, 2021).

##### Specimens examined.

Belize. **Cayo**: Raspaculo River to Macal River near the junction of both rivers, mixed tropical hardwood forest dominated by secondary species along the side of the river, *T. Hawkins 1292* (MO). Costa Rica. **Alajuela**: Carrillos de Poás, cerca del Río Poás, *A.M. Brenes 17414* (CR); Carrillos de Poás, Old road to Poás, *A.M. Brenes 17390* (CR); San Pedro de San Ramón (orillas del Río Barranca), *A.M. Brenes 21894* (CR); Naranjo, San Juan, *R. Ocampo 764* (CR); Atenas, alrededores de las Oficinas Administrativas, Escuela Centroamericana de Ganadería, *J. Gómez 6102* (CR); La Garita, dam on the Rio Grande de Tarcoles, W of Alajuela, *R.W. Pohl & G. Davidse 11353* (US, CR); Aguacate, *A.S. Oersted 14145* (US), *A.S. Oersted 14147* (US), *A.S. Oersted 14050* (US); Grecia, *E. Anderson 1324* (US). **Cartago**: Turrialba, Terrenos del Instituto Interamericano de Ciencias Agrícolas, J. León 412 (MO); Growing on rocks along the Río Pacuare, between San Rafael and Moravia, *R.W. Pohl & G. Davidse 11476* (CR); Turrialba, Terrenos del IICA, *J. León 1485* (CR), *412* (MO); Navarro, 1 km N of Puente Negro, *R.W. Pohl & G. Davidse 11184* (US, CR); Tres Rios, *H. Pittier 3029* (US, CR). **Guanacaste**: Cañas, Finca La Pacífica, *R. Daubenmire 345* (USJ); Carrillo, *J. A. Echeverría 289* (CR); Nicoya, *R. Ocampo 757* (CR); Santa Rosa National Park, 30 km NW of Liberia, *D. H. Janzen 12267* (MO); Parque Nacional Guanacaste Sector Agua Buena, margen occidental del Río Animas, *A. Chacón et al. 667* (MO, INB, CR); Santa Rosa National Park. Nature trail, forest, *R. Liesner 4313* (MO, CR); La Cruz, Quebrada Costa Rica, Santa Rosa National Park, *E.J. Judziewicz 4286* (MO, CR); Bagaces, 7 km by road N of Bagaces. Quercus-Curtella savanna on volcanic tuff, *R.W. Pohl & M. Lucas 13077* (MO, CR); Deciduous broad-leaf forest with tress to 20 m tall. Between Liberia and Bagaces near the Rio Potrero along the Interamerican hwy, *W. Burger & W. Ramirez 4120* (US, CR). **Heredia**: 5 km al Oeste de San Joaquín de Flores, en un paredón, *M. Montiel s.n.* (CR); vicinity of horseback-riding facility at Tajo de Cariari, ca. 1 km SE of Ciudad Cariari, along Río Virilla, *M. Grayum 4294* (MO, CR); San Vicente, Bosques residuales en las vegas del Río Bermudez, *J.A. González 2485* (CR). **Limón**: Salamanca, Telire, *R. Ocampo 3893* (CR). **Puntarenas**: Montes de Oro, low cliffs along the Río Ciruelas at the Interamerican Hwy, 8 km N of the Puntarenas intersection, *R.W. Pohl & G. Davidse 11288* (CR); Cordillera de Tilarán, Monteverde, cliff edge and descending ridge below Hotel de Montaña, *W. Haber & W. Zuchowski 10893* (CR, MO, INB); Monteverde, 6 km SW Santa Elena on road to Inter American highway. Dry ridge above Río Lagarto, *W. Haber & W. Zuchowski 10155* (INB, MO, CR); Montes de Oro, Quebrada Seca, Cerro Zapotal, Miramar, *L. D. Gómez et al. 23988* (MO); Barranca, 1.5 km S of the Puntarenas intersections on the Carretera Interamericana, *R. W. Pohl & G. Davidse 11346* (CR). **San Jose**: Cul-de-sac at N. end of Calle 9, *Khan et al. 142* (MO, CR); On walls, Museo Nacional, *R.W. Pohl 14168* (MO); Sur un rocher au bord du rio Virilla, sous le pont du F. T. R., près San Juan, *A. Tonduz 17557* (CR); Techados y paredones de San José, *O. Jiménez 50* (CR) ; San Pedro, near Pulpería La Luz, along Avenida Central, *R.W. Pohl & G. Davidse 11248* (US, CR); Acosta, *R. Ocampo 1137* (CR); Sur les mur a San José, *H. Pittier 544* (CR); Sur un rocher au bord d’un ruisseau a La Verbena, pres Alajuelita, *A. Tonduz 9086* (US, CR); Toits des maisons a San Jose, *H. Pittier (Tonduz*, *A.) 3015* (US, CR); vicinity of La Verbena, *P. C. Standley 32288* (US); Between San Pedro de Montes de Oca and Curridabat, *P. C. Standley 32816* (US); San José, *P.C. Standley 41243* (US); Toiss des maisons a San Jose, *A. Tonduz 775* (US); Hills SW of San Jose, *E.W.D. Holway 304* (US), *408* (US); San José, *A.S. Hitchcock 8460* (US), *8501* (US), *8509* (US); San José, *H. Pittier 33* (US, CR); Acosta, San Ignacio, *R.A. Ocampo 792* (CR); Acosta. Bajo Jupa, camino a Bajo Palma, *J.F. Morales 7438* (CR. INB); Cuenca del Pirrís-Damas. Valle del Río Candelaria, Quebrada Guápiles, antes del Soslayo, *J.F. Morales 11629* (INB, MO). El Salvador. **Ahuachapán**: San Benito, al E de la vuelta del río Aguachapio, *E. Sandoval & Chinchilla 793* (MO); El Impossible, by Las Positas river, evergreen tropical moist forest, canopy height ca 30 m., *A. Monro et al. 1912* (MO); El Impossible, bosque el Pacayito, quebrada La Cascada, *W.G. Berendsohn et al. 1332* (LAGU, MO); San Fco. Menéndez, Hda. San Benito, Río Guayapa, *E. Sandoval & Chinchilla 102* (MO); Ahuachapán, *S.A. Padilla 133* (US). **Chalatenango**: La Palma, Recreo Obrero El Refugio, *P. Bernhardt & E.A. Montalvo 41* (MO, ITIC). **La Libertad**: Finca La Giralda, 5 km before Gomasagua, cafetal de sombra orgánica, hasta 12–20 m de alto, dominado por Inga, *A. Monro et al. 3080* (MO). **Morazán**: Arambala, A. P. Sapo, cantón Cumaro, camino a piedra x, *R. A. Carballo & J. Monterrosa 935* (MO); *L. Lara 240* (MHES); *J.F. Morales 14135* (LAGU, MHES); Jocoaitique, río Araute, sector Las Raices, *D. Rodríguez et al. DR-00537* (B, BM, LAGU, MO). **San Miguel**: Finca El Pacayal, Volcán Chinameca, coffee farm, shade (60%) with low diversity of shade trees, canopy to ca. 12–14 m, *A. Monro et al. 2940* (MO). **San Salvador**: vicinity of San Salvador, *P.C. Standley 22410* (MO), *21783* (MO); *19256* (MO); San Salvador, *S. Calderón 497* (MO); San Salvador, *N.L.H. Krauss 1002* (US). **San Vicente**: vicinity of San Vicente, *P.C. Standley 21208* (US). **Sonsonate**: vicinity of Sonsonate, *P.C. Standley 21783* (MO); Coastal plain, rocks along river, *A.S. Hitchcock 8974* (US). **Usulután**: Laguna de Alegría, cerca de la entrada, *D. Williams & R.W. Herrera 361* (MO), *341* (MO), *D. Williams s.n.* (MO); *J. Menjívar et al. 639* (MHES); Laguna de Alegría, *D. Williams s.n.* (MO). Guatemala. **Chiquimula**: Quebrada Shusho, above Chiquimula, *P. C. Standley 74318* (US). **Escuintla**: along Rio Guacalate, *P.C. Standley 58243* (US). **Guatemala**: San Raimundo, along National Hwy. 5, between Salamá and Guatemala City (via El Chol), 2 km past turnoff to San Raimundo (leaving paved road), dry oak forest, *T.B. Croat & D.P. Hannon 63517* (MO); Damp wooded barranca 10 km south of San Raimundo, damp shaded bank, *P.C. Standley 62868* (US); Fiscal, near Guatemala, *W.A. Kellerman 6244* (MO). Santa Rosa: Santa Rosa, *Heyde & Lux 3913* (MO). **Jutiapa**: Hills between Jutiapa and Plan de Urrutia, north of Jutiapa, *P. C. Standley 75531* (US). Santa Rosa: Santa Rosa, *J. Donnell Smith 3913* (US, MO). **Suchitepéquez**: Mazatenango, *Bernoulli 35* (US). Honduras. **Comayagua**: Ojo de Agua, orilla Río Humuya, 30 km N de ciudad Comayagua, bosque de vega tropical rodeado de pinares, *C. Nelson et al. 6830* (MO), *7000* (MO); Chichipates, orilla del Río Yure, bosque tropical de vega; 30 km E Lago Yojoa, pinares y robledales, *C. Nelson et al. 6716* (MO); Rio Yure, unión del río Yure con el Río Humuya, 100 km NO de Ciudad de Comayagua, bosque tropical de vega húmedo, pinares y robledales, *C. Nelson et al. 6113* (MO); Agua Caliente, vaguada de Ríos Chamo y Humuya, 35 km E Lago Yojoa, pinares y robledales, bosque de vega tropical, *C. Nelson et al. 6391* (MO); In stream course of Quebrada Cana, 3 km S and W of Las Flores, *C.L. Johannessen 59* (US). **Copán**: Santa Rosa, bosque de pinos, *M.E. Villena 22* (MO); Santa Rosa, damp shady places, also rock walls, *W.A. Archer 3855* (US); Santa Rosa de Copán, *M.E. Villeda 22* (MO). **Cortés**: Orilla del Río Humuya, 40 km N Santa Cruz de Yojoa, bosque de vega tropical, *C. Nelson et al. 5840* (MO). **El Paraíso**: 9 km S of Yuscaran, *R.W. Pohl & M. Gabel 13441* (MO, CR). **Francisco Morazán**: San Antonio de Oriente, Campus of Escuela Agricola at El Zamorano, *R.W. Pohl 12482* (MO, CR); San Antonio de Oriente, Las Mesas, ca. 5 km E of El Zamorano, near Riachuelo Las Mesas, *R.W. Pohl 12520* (MO, CR); Distrito Central, Parque del Cerro El Picacho, pinares, bosque premontano húmedo, *V.L. Ochoa 83* (MO); Distrito Central, Cerro el Hatillo, 15 km al NE de Tegucigalpa; bosque premontano húmedo, *L.M. Ordóñez 58* (MO): Road toward San Antonio de Oriente, region El Jicarito, above El Zamorano, *P.C. Standley 27477* (MO); San Antonio de Oriente, Zamorano, bosque seco subtropical, *D. Aguilar S. 38* (MO); San Antonio de Oriente, near Las Mesas *L.O. Williams & A. Molina R. 10830* (MO); vicinity of El Zamorano, rocky river banks, Galeras, *J. R. Swallen 10775* (US); bosque mixto entre Cuesta de los Muertos y Monte Obscuro, La Montañita, *L. O. Williams & A. Molina R. 11161* (US); Drainage of the Rio Yaguare, Rivera del Rio Yaguare cerca de Finca San Francisco, Zamoran, *A. Molina 1581* (US); San Antonio del Oriente, moist shady bank, *J.R. Swallen 10917* (US); vicinity of Suyapa. Moist bank along road near Suyapa, *J.R. Swallen 11294* (US); Foothills of Mt. Uyuca, beyond Las Floras, moist open banks, *J.R. Swallen 11297* (US); Carretera a Valle Angeles, 15 km NO de Tega, *L. Trochez 219* (US). **La Paz**: Montaña de Opatoro, 36 km SE de Marcala, bosque húmedo subtropical, *R. Martínez 284* (MO); **Olancho**: Juticalpa, Rio Guayapa, between Jutiapa and Concordia, *R.W. Pohl & M. Gabel 13762* (MO, CR). **Santa Barbara**: Alrededores de Santa Barbara, area de Pino-roble de Rio Ulua, *A. Molina 3789* (US). **Yoro**: Victoria, orilla del Río Sulaco, bosque de vega tropical, *C. Nelson et al. 7217* (MO). Mexico. **Chiapas.** Ocozocuautla, Cañada “El Aguacero”. Selva baja. En las laderas del cañón “La Venta”, *J.J. Ortíz Díaz 1010* (MO). **Acala**: Wooded slope along the Río Grijalva, 10 kilometers south of Mexican Highway 190 along the road to Acala at Nandaburri, *R.M. Laughlin 2813* (DS). **Angel Albino Corzo**: slopes of Río Cuxtepec below Finca Cuxtepec, *D.E. Breedlove & G. Davidse 54700* (CAS, MO); slopes of Río Cuxtepec below Finca Cuxtepec, *D.E. Breedlove & G. Davidse 54675* (CAS, MO). Arriaga: 13 km N of Arriaga along Mex. Hwy. 195, *D. E. Breedlove 28273* (NY). **Chiapa de Corzo**: El Chorreadero 5.6 miles east of Chiapa de Corzo along Mexican Highway 190, *R.M. Laughlin 2608* (DS). Frontera Comalapa: S of Frontera Comalapa on Mex hwy 211 and 18 mi SW of Jtn 190 and 211, small rocky water course which feeds Rio Grijalva, *P.M. Peterson & C.R. Annable 4704* (ARIZ, ENCB, GH, MEXU, MICH, MO, NMC, NY, RSA, TAES, UC, UNLV, US, UTC, WIS, WS); 13 km N of Arriaga along Mex. Hwy. 195, *D.E. Breedlove & G. Davidse 54150* (CAS, MO). Ixtapa: 1 km W of Ixtapa, *F.W. Gould 12715* (US, MO); along Mex 190 in the Zinacantán paraje of Muctajoc, *D.E. Breedlove 13820* (US, DS); near Ixtapa, *D.E. Breedlove & G. Davidse 54265* (CAS, MO); at Escopetazo, *D.E. Breedlove & G. Davidse 54944* (CAS, MO); at Esc opetazo, *D.E. Breedlove & G. Davidse 53944* (CAS, MO). **Ocosingo**: near El Real, E of Ocosingo, *D.E. Breedlove & G. Davidse 56381* (CAS, MO). **Ocozocoautla de Espinoza**: El Aguacero, canyon of the Río La Venta, *G. Davidse et al. 30071* (MO); El Aguacero en el río La Venta, *E. Martínez 22006* (MEXU). **Tonalá**: Cerro Vernal [Cerro Bernal], 21 km S of Tonalá, *G. Davidse et al. 38123* (MO). **Tuxtla Gutiérrez**: El Zapotal, *Ruiz 017* (MEXU); 16 mi W of Pan American Hwy, *Carlson 2066* (MICH). **Villa Corzo**: 65 km S of Mexican Highway 190 on road from Tuxtla Gutiérrez to Nueva Concordia, *D.E. Breedlove & G. Davidse 54464* (CAS, MO); Above Colonia Vincente Guerrero on road to Finca Cuxtepec, *D.E. Breedlove & G. Davidse 54595* (CAS, MO); near Colonia Vicente Guerrero, *D.E. Breedlove 48607* (CAS, MO); 58 km S of Mexican Highway 190 on road to Nueva Concordia, *D.E. Breedlove 37645* (MO). Nicaragua. **Chontales**: Hacienda Veracruz, including Cerro La Batea, pasture on rocky slopes and deciduous forest on basaltic mesas, *W. D. Stevens 23359* (MO), *23309* (MO); Hacienda San Martín, near confluence of Río El Jordán and Río La Pradera; remnant tall evergreen forest, *W.D. Stevens 22862* (MO, CR); Río El Bizcocho, along road from Juigalpa NE toward La Libertad, ca. 17.4 km NE of Río Mayales, at ford of Río El Bizcocho; pastures, gallery forest and steep cliffs S of river, *W.D. Stevens 4033* (MO). **Granada**: shore of Lago de Nicaragua, very dry soil except at water’s edge [Seymour series], *J.T. Atwood 1078* (MO). **Jinotega**: Salto Kayaska, Río Bocay, among rocks along river to top of hills SW of falls, tall evergreen forest on hill, limestone, *W.D. Stevens et al. 16602* (MO). **Managua**: Tipitapa, 22 km N of Managua [Seymour series], *H. Zelaya M. 40A* (MO). **Matagalpa**: Finca La Salvadora, Vuelta del Coyolito, bordeando el Río Yasica; zona boscosa, *D. Castro C. 2217* (MO). **Nueva Segovia**: Ocotal, 3 km W of city; mostly in deep ravine along dry river bed [Seymour series], *J. T. Atwood 758* (MO), *C.E. Nichols 819* (MO). **Rivas**: Southwest of La Virgen and northeast of San Juan del Sur, route 16 where road crossed brook, km 136 [Seymour series], *J.T. Atwood 1174* (MO). Panama. **Chiriqui**: vicinity of Boquete, from Boquete to 3 mi N., second growth, cultivared areas, and roadside, *W.H. Lewis et al. 332* (MO); On rocks along Río Cuvibora ca. 5 miles N of Tolé on road past Alto Caballero, *B. Hammel 6256* (MO); Río Caldera, NW of Boquete, just S of Horqueta, near a small waterfall, volcanic cliff, *P.M. Peterson & C.R. Annable 7375* (MO, US); Cerro Vaca, eastern Chiriqui, in savannas, *H. Pittier 5308* (US); Foothills, vicinity of El Boquete, on bare rock, hilltop, *A.S. Hitchcock 8316* (US). **Cocle**: Valley of the upper Río Mata Ahogado, *P.H. Allen 137* (MO), *141* (MO); Hills south of El Valle de Antón, *P.H. Allen 2806* (MO).

#### ﻿33. Muhlenbergia tenuissima

Taxon classificationPlantaePoalesPoaceae

(J. Presl) Kunth, Enum. Pl. 1:198 (1833).

8B953268-A6A8-590F-AF79-FA1CA630508D

Fig. 19E–H

Podosemum tenuissimum Presl, Rel. Haenk. 1: 230. 1830. Type: Panama or México. *Haenke s.n.* (**lectotype, designated here**: PRC-450957 [image!]; isolectotypes: MO-2974335!, US-91923!, W-0002565!, W-0002566!, W-239298!). Basionym:
= Muhlenbergia nebulosa Scribn. ex Beal, Grasses N. Amer. 2: 247. 1896. Type: México, Jalisco, wet places, hills near Guadalajra, 5 Nov 1889, *C.G. Pringle 2366* (holotype: MSC-11271!; isotypes: BR-0000006883102 [image!], BR-0000006883430 [image!], E-00373720 [image!], F-73212 [image!], GH!, GOET-006642 [image!], IBUG-0179259 [image!], K!, KFTA-0000118 [image!], LL-00370119 [image!], MEXU-00005184 [image!], MEXU-00005183 [image!], UC-122478 [image!], NY!, RSA!, S-G-4200 [image!], SI-002778 [image!], US!, VT!, W-1916-29044!, W-1890-597!).

##### Description.

Delicate, slender, ***annuals***. ***Culms*** 5–30(–38) cm tall, much branched near base and above often sprawling, glabrous or scaberulous below the nodes; 0.1–0.2 mm diameter just below the inflorescence; ***internodes*** 7–30 mm long. ***Leaf sheaths*** 4–20 mm long, glabrous or scaberulous, shorter than the internodes; margins membranous; ***ligules*** 0.6–1.5 mm long, membranous, thin, apex rounded to acute, sometimes lacerate; ***blades*** 1–5 cm long, 0.6–1.4 mm wide, flat or loosely involute, short pubescent above and glabrous to scaberulous below, margins and abaxial midvein often whitish-thickened. ***Panicles*** 6–16 cm long, 1.0–2.5(–6.0) cm wide, open, few-flowered with 9–16 nodes; ***panicle branches*** 0.5–4.0 cm long with ascending and spreading branches up to 70° from the culm axis; ***pedicels*** 1–4 mm long, scabrous, erect, ascending. ***Spikelets*** 1.3–1.9 mm long; ***glumes*** 0.4–0.8 mm long, subequal, subacute, mostly glabrous, 1-veined, minutely scabrous along veins and near apex; ***lower glumes*** 0.4–0.7 mm long, apex sometimes apiculate; ***upper glumes*** 0.6–0.8 mm long, apex sometimes mucronate, the mucro up to 0.2 mm long; ***lemmas*** 1.3–1.8 mm long, lanceolate, very thin, awned, pilose along the margins and midvein, the hairs up to 0.3 mm long, the awn 3–9 mm long, scabrous, mostly straight; callus short pilose; ***paleas*** 1.3–1.9 mm long, as long or slightly longer than the lemma, narrow lanceolate, loosely pilose between the two veins on the proximal 2/3; ***anthers*** 0.5–0.6 mm long, purplish or pale. Caryopses 0.8–1.1 mm long, fusiform, light reddish-brown.

##### Distribution.

*Muhlenbergiatenuissima* ranges from México in Chiapas, Colima, Jalisco, Nayarit, and has been reported in Sonora and Sinaloa (Dávila et al. 2018; Sanchez-Ken 2018). It also extends into Costa Rica, Honduras, Nicaragua, and Panama.

##### Ecology.

This species occurs in tropical forests, savannah grasslands with *Quercus* and *Acacia* often in calcareous derived soils in wet soils, moist depressions, and ephemeral moist flats; 50–1900 m.

##### Comments.

This distinctive species is under collected and confused with other ephemeral annuals. Distinguishing features include lemmas 1.3–1.8 mm long, pilose margins and midvein, apical awns 3–9 mm long, and short anthers 0.5–0.6 mm long (Peterson and Annable 1991).

*Muhlenbergiatenuissima* is a member of M.subg.Pseudosporobolus where it is found to be sister to *M.wrightii* Vasey ex J.M. Coult., although the branches that unite these species are long suggesting a moderate amount of genetic divergence (Peterson et al. 2021).

##### Specimens examined.

Costa Rica. **Guanacaste**: 5 km S of Liberia along the Carretera Interamericana, *R.W. Pohl & G. Davidse 11554* (US); 2 km E of carretera Interamericana on road to Las Animas, *R.W. Pohl & G. Davidse 11530* (CR, UD). Honduras. **Comayagua**: vicinity of Siguatepeque, *P.C. Standley 55871* (US). Morazán: Las Mesas, *J.V. Rodriguez 3683* (US). **Olancho**: tree infested savanna called Sav. Amate, ca. 14 km NE of Catacamas, *C.L. Johannessen 964* (US). **Yoro**: Savanna of Puentecita ca. 8 km N of Yoro, *C. L. Johannessen 707* (US). Mexico. **Chiapas**: **Ixtapa**: near Ixtapa, *D.E. Breedlove & G. Davidse 54331* (CAS, MO). Nicaragua. **Chontales**: Quebrada Niscala, ca. 2.3 km SE of bridge over Quebrada Niscala along road between Acoyapa and Río Oyate; savanna, *D.W. Stevens 19067* (MO). Panama. **Panamá**: Panama City, along road between Panamá and Chepo, *C.W. Dodge et al. 16687* (MO); Juan Diaz, *M.A. Cornman 625* (US); *E.P. Killip 4214* (ARIZ); Agricultural Experiment Station at Matías Hernandez, *H. Pittier 6918* (US); Meadows, Sabana of Panama, Canal Zone, *H. Pittuer 2544* (US); Rio Tecumen, *P.C. Standley 29418* (US); along road between Panama and Chepu, *C.W. Dodge 16687* (US).

#### ﻿34. Muhlenbergia uniseta

Taxon classificationPlantaePoalesPoaceae

(Lag.) Columbus, Aliso 28: 66. 2010.

0F8337F6-2DBE-5BE5-9E2E-57984BD086C9

Fig. 20F, G

Hymenothecium unisetum Lag., Gen. Sp. Pl. 4. 1816. Type: México, cultivated from seed at MA, *D. Sessé & Mociño s.n.* (holotype: MA). ≡ Aegopogongeminiflorusvar.unisetus (Lag.) E. Fourn., Mexic. Pl. 2: 71. 1886. Basionym:
= Lamarckia tenella DC., Cat. Pl. Horti Monsp. 120. 1813. Type: cult. hort. Monsp., *De Candolle s.n.* (**lectotype, designated here**: US-75926! ex MPU; isolectotype: US-75925! ex MPU). ≡ Hymenotheciumtenellum (DC.) Lag., Gen. Sp. Pl. 4. 1816. ≡ Aegopogontenellus (DC.) Trin., Gram. Unifl. Sesquifl. 164. 1824.
= Schellingia tenera Steud., Flora 33: 232. 1850. Type: México, Oaxaca, Cordillera, 1840, *H. Galeotti 5750* (**lectotype, designated here**: P-00745714 [image!]; isolectotypes: P-00745713 [image!], P-00745715 [image!], W-18890117307 [image!], W-0001035 [image!]).

##### Description.

Slender often sprawling, caespitose ***annuals***. ***Culms*** (2–)6–30 cm tall, glabrous below the nodes; ***internodes*** 0.6–6 cm long, glabrous to pilose. ***Leaf sheaths*** mostly 0.5–4.8 cm long, shorter than the internodes, glabrous to sparsely pilose; ***ligules*** 0.6–1.5 mm long, apex mostly truncate, lacerate; ***blades*** 1.5–6 cm long, 0.5–1.5(–1.7) mm wide, flat, scaberulent and pubescent above, smooth beneath. ***Panicles*** 2–6 cm long, 0.5–1.2 cm wide, open, loosely-flowered with racemose branches; ***primary branches*** 3–5 mm long, excluding the awns, one per node; one short-pedicelled spikelet (perfect) with ***pedicels*** 0.2–0.6 mm long and the other two spikelets (staminate or sterile) short-pedicelled, the ***pedicels*** about 0.7–1.5 mm long. ***Spikelets*** 1.5–3.2 mm long, often greenish or purplish; ***glumes*** (1–)1.3–2 mm long, oblong and wider distally, apex deeply notched, entire or mucronate, the mucro 0.2–1 mm long, lobes obtuse or rounded; ***lemmas*** 2.5–3.2 mm long, 3-awned, the central awns 3–8(–11) mm long, lateral awns usually mucronate or awned up to 2 mm long or missing; ***paleas*** 2.2–3 mm long, puberulent, apex 2-mucronate, the mucros less than 0.8 mm long; ***anthers*** 0.5–0.8 mm long, yellowish. ***Caryopses*** 1.1–1.3 mm long, obovoid, light brownish. 2*n* = 20, 60.

##### Distribution.

*Muhlenbergiauniseta* ranges from southern Arizona throughout most of México and Central America (Belize, Costa Rica, El Salvador, Guatemala, Honduras, México, and Panama) [Pohl 1994a; Peterson et al. 2001].

##### Ecology.

*Muhlenbergiauniseta* grows on moist slopes, cliffs, barrancas, canyons, roadsides, and along or near springs usually in shaded areas associated with *Pinus* and *Quercus*; 1300–2860 m.

##### Comments.

*Muhlenbergiauniseta* can be separated morphologically from *M.cenchroides* in having glumes with obtuse or rounded lobes (acute in *M.cenchroides*) that are entire or mucronate (awned 2–4 mm long in *M.cenchroides*) and by being annual (versus perennial).

*Muhlenbergiauniseta* is a member of M.subg.Muhlenbergia and forms a clade with *M.cenchroides* and *M.bryophilus* (Fig. 1; Peterson et al. 2021).

##### Specimens examined.

Costa Rica. **Heredia**: San Isidro, Concepción, Calle Leones, *E. Alfaro 4724* (INB). **San José**: Dota, R.F. Los Santos. Cuenca del Pirris-Damas, Río Blanco, cabeceras Quebrada Vueltas, *J.F. Morales 7088* (MO); vicinity of Santa María de Dota, *P.C. Standley 41720* (US); Aserrí, Cuenca del Pirris-Damas, Cerros Caraigres, Falda S, Quebrada Concha, en el camino viejo a Bijagual, *J.F. Morales 5914* (MO, INB); Aserri, Z.P. Cerros Escazú, Cuenca del Tárcoles, bosques primarios y secundarios en la cima del Cerro Daser (Alto Hierbabuena), *J.F. Morales 6707* (MO,INB); Acosta, Alto Reflis, Falda NE, Fila de Cal, *J.F. Morales 7486* (CR, INB); Desamparados, Tablazo, *J.A. Echeverría 1210* (CR); Entre San Isidro y División, *s.c 3855* (CR); Cord. Talamanca, 14 km S of Division along the Interamericana Hwy., roadside through oak forest, *R.W. Pohl & G. Davidse 11615* (US). El Salvador. **La libertad**: *E. Montalvo et al. 6425* (LAGU). **Santa Ana**: *O. Rohweder 2394* (MO). **Usulután**: *J. Menjívar et al. 3591* (LAGU, MHES, MO); *G. Cerén et al. 3276* (MHES, MO). Guatemala. **Guatemala**: Volcán de Pacaya, above Las Calderas, *P. C. Standley 58340* (US); Volcán de Acatenango, *J. & M. Véliz 93.3425* (MEXU). **Sacatepéquez**: Ciudad Vieja, *H. Bethancourt & M. Véliz 94.4181* (MEXU, MO). **Sololá**: near Sololá, *A. Gentry 6504* (MO); slopes of Volcán de Agua, south of Santa Maria de Jesús, *P.C. Standley 59361* (US). Honduras. **Francisco Morazán**: El Zamorano, North side of Cerro Uyuca, near the farmhouse, *R. W. Pohl & M. Gabel 13413* (CR); On open slopes near Hoya Grande, *L.O. Williams & A. Molina 10994* (US); Open slopes in cloud forest area in mountains above San Juancito, *L.O. Williams & A. Molina 13366* (US); Santa Lucía, camino entre la montañita y Santa Lucía, *J.L. Linares & J. Alana 1901* (MEXU); Escuela Agrícola Panamericana, Faldas de La Montañita, *A. Molina R. 1612A* (US). Mexico. **Chiapas**: **Huixtlán**: 10 km E of Huistan, *D.E. Breedlove & G. Davidse 55169* (MEXU); *D.E. Breedlove & G. Davidse 53853* (MO). **Larráinzar**: NE of Bochil, *D.E. Breedlove 29276* (MEXU). **San Cristóbal de las Casas**: 16 km al NW de San Cristobal de las Casas, sobre el camino a Tenejapa, *E. Cabrera & H. de Cabrera 5733* (MEXU). **Zinacantan**: hwy 190 between paraajes of Nachic and Navenshauk, *D.E. Breedlove 7304* (US). Panama. **Chiriquí**: Large old lava flow ca. 3 km NE of El Hato del Volcán at base of Volcán de Chiriquí (Barú), 1–3 km E of highway, *G. Davidse & W.G. D’Arcy 10386* (MO).

#### ﻿35. Muhlenbergia utilis

Taxon classificationPlantaePoalesPoaceae

(Torr.) Hitchc., J. Wash. Acad. Sci. 23(10): 453. 1933.

86EE4133-6F9C-5977-A4ED-47B0E7B2AB8E

Fig. 18 F–K

Vilfa utilis Torr., Pacif. Railr. Rep. 5(2):365–366. 1857. Type: U.S.A., California, Lost Mountain Spring, from Tejon to the Lost Hills, in stony places, *W.P. Blake s.n.* (holotype: NY-00431757 [image!]; isotypes: GH-00023997 [image!], MO-992072!). ≡ Sporobolusutilis (Torr.) Scribn., Bull. Div. Agrostol., U.S.D.A. 17:171, f. 467. 1899. Basionym.

##### Description.

***Perennials***; with slender, scaly rhizomes. ***Culms*** 7–30 cm tall, erect to decumbent, older plants trailing, up to 1 m long, minutely pubescent to glabrous below the nodes; ***internodes*** mostly smooth to lightly nodulose roughened. ***Leaf sheaths*** 0.3–2.4 cm long, shorter or longer than the internodes, glabrous, margins hyaline; ***ligules*** 0.2–0.8 mm long, membranous, decurrent, apex truncate; ***blades*** 0.5–4.7 cm long, 0.2–1.8 mm wide, involute, sometimes flat, straight or arcuate-spreading, the blades often at right angles to culms, mostly glabrous abaxially and hirsutulous adaxially. ***Panicles*** 1–5 cm long, 0.1–0.4 cm wide, narrow, contracted, interrupted between each branch, partially included in the upper sheaths; ***primary branches*** 0.2–1.2 cm long, appressed, rarely ascending to spreading 30° from the rachises; rachises usually visible between the branches; ***pedicels*** 0.1–1.1 mm long, glabrous. ***Spikelets*** 1.4–2.4 mm long, erect; ***glumes*** 0.5–1.4 mm long, ⅓ to as long as the lemma, subequal, unawned, glabrous, 1-veined, occasionally 2-to 3-veined, yellowish to light green, apex acute; ***lemmas*** 1.3–2.4 mm long, lanceolate, unawned, glabrous or with minute appressed pubescence along the margins and the base, the hairs about 0.1 mm long, green or purplish, apex acute; ***paleas*** 1–2 mm long, lanceolate, glabrous, apex acute; ***anthers*** 0.7–1.4 mm long, yellow to purplish. ***Caryopses*** 0.7–1.2 mm long, ellipsoid to ovoid, brown. 2*n* = 20.

##### Distribution.

*Muhlenbergiautilis* ranges from the southern United States to México in Chiapas, Chihuahua, Durango, Guanajuato, Hidalgo, Jalisco, México, Ciudad de México, Michoacán, Puebla, Querétaro, Sonora, Veracruz, and Zacatecas, and Central America (Guatemala and México) [Herrera Arrieta and Peterson 2018].

##### Ecology.

This species occurs in wet soils along streams, ponds, depressions in grasslands, and alkaline or gypsiferous plains associated with *Quercus* spp.; 200–2500 m.

##### Comments.

Morphologically, *Muhlenbergiautilis* can be confused with *M.vaginata* but the former differs in having slender, scaly rhizomes, (*M.vaginata* without rhizomes but culms decumbent and rooting below can sometimes be mistaken for rhizomes) and unawned lemmas (mucronate in *M.vaginata*).

*Muhlenbergiautilis* is a member of M.subg.Pseudosporobolus and forms a clade with *M.repens* and *M.villiflora* Hitchc. (Fig. 1; Peterson et al. 2021).

##### Specimens examined.

Mexico. **Chiapas**: **San Cristóbal de las Casas**: Marsh at S end of San Cristóbal de las Casas, *D.E. Breedlove 54373* (ENCB, SLPM, CAS, MO).

#### ﻿36. Muhlenbergia vaginata

Taxon classificationPlantaePoalesPoaceae

Swallen, Contr. U.S. Natl. Herb. 29(9): 406. 1950.

6C2DA1AC-5621-5A13-A8A0-74AB87D27E46

##### Type.

Guatemala, San Marcos, road between San Sebastián and San Marcos, 2700–3800 m, 15 Feb 1940, *J.A. Steyermark 35598* (holotype: F-1046643!; isotypes: US-2240531!, US-2236472!).

##### Description.

***Annuals*** or short-lived ***perennials***. ***Culms*** 16–40 cm tall, lax, slender, glabrous, decumbent, rooting at the lower nodes. ***Leaf sheaths*** 0.7–1.6 cm long, shorter than the internodes, glabrous; ***ligules*** 1.3–3 mm long, hyaline, apex acute to obtuse, decurrent; ***blades*** 0.5–3.5 (–4) cm long, 0.6–1.8 (–2) mm wide, mostly cauline, flatted or folded, with navicular apex, glabrous to scabrous. ***Panicles*** 0.5–3 cm long, 3–7 mm wide, frequently partially included in the upper sheath; ***primary branches*** 3–10 mm long, ascending or appressed; ***pedicels*** 0.5–3 mm long, appressed, scabrous. ***Spikelets*** 1.6–2.5 mm long; glumes 0.6–1 mm long, subequal, glabrous, 1-veined, oblong to ovate, light green to green-grayish, apex obtuse, rounded to subacute, occasionally erose; ***lower glumes*** 0.6–0.8 mm long; ***upper glumes*** 0.6–1 mm long; ***lemmas*** 1.6–2.5 mm long, lanceolate, sparsely pubescent below and along the midvein and margins, mottled with olive-green spots, often purplish, apex scabrous, acuminate, sometimes mucronate, the mucro 0.2 mm long; ***paleas*** 1.5–2.5 mm long, lanceolate, glabrous or with a few hairs between the veins; ***anthers*** 0.5–0.8 mm long, purplish. ***Caryopses*** 1–1.2 mm long, ellipsoid to fusiform, brown. *n* = 9.

##### Distribution.

*Muhlenbergiavaginata* ranges from México to Guatemala in Central America (In México it is found in Chiapas, Chihuahua, Ciudad de México, Durango, Hidalgo, Jalisco, México, Michoacán, Morelos, Puebla, Querétaro, Sinaloa, Tlaxcala, and Veracruz (Peterson and Annable 1991).

##### Ecology.

This species is found in wet meadows, wet depressions, open flats, sandy flats along creeks, and rivers in pine or pine–oak woodlands; 1500–3800 m.

##### Comments.

Morphologically, *M.vaginata* can be separated from *M.ligularis* in having contracted, narrow panicles up to 0.7 cm wide (0.4–1.5 cm wide in *M.ligularis*) and culms often rooting at the lower nodes (not rooting at lower nodes in *M.ligularis*) [Peterson and Annable 1991]. *Muhlenbergialigularis* can be separated morphologically from *M.filiformis* in having inconspicuous panicles partly enclosed in the sheath (conspicuous, long-exerted panicles in *M.filiformis*) and leaf blades that are well developed along the entire length of the culm (versus leaf blades most numerous near the base of the culm) [Peterson and Annable 1991].

Based on DNA sequence analysis, *Muhlenbergiavaginata* is in M.subg.Bealia and forms a clade with *M.filiformis* and *M.ligularis* (Fig. 1; Peterson et al. 2021).

##### Specimens examined.

Guatemala. **Huehuetenango**: La Capellania, Sierra de los Cuchumatanes, 3.3 mi NW of La Capellania on hwy 9N and 14.6 mi N of Huehuetenango, *P.M. Peterson & C.R. Annable 4685* (GH, MO, NY, RSA, UC, US, WS); El Mirador, at the summit of the road leading from Huehuetenango to Sierra de los Cuchumatanes, *P.C. Standley 81872* (US); Sierra de los Cuchumatanes. 1.1 mi E of Santa Eulalia on road to San Sebastian Coatan, *P.M. Peterson & C.R. Annable 4690* (US, WS); meadow at Tojah on hwy 9N, *P.M. Peterson & C.R. Annable 4697* (US, WS). **Quetzaltenango**: along eastern side of Rio Somala opposite Santa Maria de Jesus, *J.A. Steyermark 35049* (US). **San Marcos**: San Sebastian, along road between San Sebastian at km 21 and km 8, 8–18 mi NW of San Marcos, 4 km from San Sebastián, NW of San Marcos, *J.A. Steyermark 35598* (MO). **Zinacantán**: near Paraje Nachij, *D.E. Breedlove & G. Davidse 53877* (CAS, MO). Mexico. **Chiapas: Chamula**: large sphagnum bog at Paraje Muk’in ha, *D.E. Breedlove & B. Bartholomew 55504* (SLPM, CAS, MO). **Chenalhó**: edge of pasture 1 mile south of Chenalho Center, *D.E. Breedlove & P.H. Raven 8254* (DS). **Huixtán**: Chilil, SE de San Cristóbal de Las Casas, *M. González et al. 405* (SLPM); 10.5 mi SE of San Cristobal de las Casas, *P.M. Peterson & C.R. Annable 4723* (GH, MO, NY, US, WS).

#### ﻿37. Muhlenbergia versicolor

Taxon classificationPlantaePoalesPoaceae

Swallen, Contr. U.S. Natl. Herb. 29(4): 412. 1950.

8020220F-5593-5F8B-9685-D07E789D962C

Fig. 10A–F

##### Type.

México, Oaxaca, 170 km N of Oaxaca, 13 Dec 1945, *E. Hernandez-Xolocotzi & J.A. Jenkins X-810* (holotype: US-1961991!).

##### Description.

Caespitose ***perennials***. ***Culms*** (80–)100–150 cm tall, erect, with 3 or 4 nodes, strigulose below the nodes; ***internodes*** mostly glabrous. ***Leaf sheaths*** longer than the internodes, basal sheaths compressed but not strongly keeled, the old sheaths brown becoming fibrillose with age, lower sheaths short pilose; ***sheath auricles*** 3–10 mm long, hyaline, wide, becoming frayed at the apex when mature; ***ligules*** (4–)7–22 mm long, hyaline above and firm and brown below; ***blades*** 25–40 cm long, 3–5 mm wide, blades arising near the middle of the culm 12–27 cm long, often shorter than those below, conduplicate or flat, finely scaberulous to scabrous, prominently veined on the upper surface. ***Panicles*** 20–50 cm long, 1.5–6 cm wide, erect, dark green to plumbeous, sometimes tinged in purple; ***primary branches*** 4–12 cm long, ascending or spreading up to 30° from the rachis; ***pedicels*** 1–2.5 mm long. ***Spikelets*** (2.5–)3–3.5 mm long; ***glumes*** (2.5)3–3.5 mm long, subequal, unveined or faintly 1-veined, apex erose-toothed, scabrous, unawned or awned; ***upper glumes*** mostly awned, the awn 1–1.2 mm long; ***lemmas*** 2.5–3(–3.5) mm long, villous on the lower ½ to 2/3, mostly pilose along the margins near the base, the awns 10–15(–25) mm long, flexuous; ***callus*** long-pilose; ***paleas*** equal to the lemmas, moderate to densely pilose between the keels; ***anthers*** up to 1.5 mm long, purple. ***Caryopses*** 1.5 mm long, 0.3 mm wide, fusiform, reddish-brown.

##### Distribution.

*Muhlenbergiaversicolor* ranges from Central México (Colima, Ciudad de México, Guanajuato, Jalisco, México, Michoacán, Morelos, Nayarit, Oaxaca, Puebla, Veracruz) to el Salvador, Guatemala, and Honduras (Sanchez-Ken 2018).

##### Ecology.

This species is mainly found in oak forests, less frequent in pine-oak woodlands, rain forests, and grasslands on slopes and disturbed roadsides associated with *Alnus*, *Salvia*, *Rubus*, and *Thalictrum*; 900–2850 m.

##### Comments.

Morphologically, *Muhlenbergiaversicolor* resembles *M.distichophylla* but differs in having longer lemmas 2.5–3(–3.5) mm long (1.4–2.7 mm long in *M.distichophylla*) and shorter sheath auricles 3–10 mm long (versus 4–26 mm long).

*Muhlenbergiaversicolor* is a member of M.subg.Trichochloa and is found in the clade with *M.breviligula*, *M.maxima*, *M.articulata* Scribn., *M.lehmanniana*, and M.longiglumis Vasey (Fig. 1; Peterson et al. 2021).

##### Specimens examined.

El Salvador. **Santa Ana**: *P. Galán et al. 3920* (LAGU); *R. A. Carballo et al. 965* (LAGU, MO). Guatemala. **Huehuetenango**: San Lorenzo, 7 km S. of San Lorenzo, *W.E. Harmon & J.A. Fuentes 4786* (MO); Aguacatan road, 10 km east of Huehuetenango, *P.C. Standley 82073* (US); Barranco “Palo Negro” about 10 km W of Aguacatan, *L.O. Williams et al. 21850* (US). **Sololá**: Lago Atitlan, *M. De Koninck 146* (US). Honduras. **El Paraíso**: In forest on Mt. Yuscaran, *A. Molina R. 605* (US). **Francisco Morazán**: San Antonio de Oriente, Guayabillas, pine forest of Guayabillas on road to Ojo de Agua, *A.R. Molina 25899* (MO); Distrito Central, Tegucigalpa, alrededores de la Universidad Nacional Autonoma de Honduras, *K.J. Cantarero 48* (MO, TEFH); vicinity of Suyapa, hills above Suyapa, *J.R. Swallen 11277* (US). Mexico. **Chiapas**: **Cintalapa**: Slope, near La Cienega de León 30 km N of Las Cruces, *D.E. Breedlove & F. Almeda 48070* (CAS, MO). **Ixtapa**: Ixtapa, at Escopetazo, *D.E. Breedlove & G. Davidse 53960* (SLPM, CAS, MO); near Ixtapa, *D.E. Breedlove & G. Davidse 54364* (CAS, MO), *D.E. Breedlove & G. Davidse 54363* (CAS, MO). **Motozintla**: SW side of Cerro Mozotal, 11 km NW of the junction of the road to Motozintla along the road to El Porvenir and Siltepec, *D.E. Breedlove & B.M. Bartholomew 55785* (CAS, MO). La Trinitaria, 12 km al S de la Trinitaria, camino a Cd. Cuauhtemoc, *E. Martínez-Salas 23905* (MEXU). *D.E. Breedlove & G. Davidse 54631* (CAS) citada en Flora Mesoamericana. **Teopisca**: Marsh near Teopisca, *D.E. Breedlove & G. Davidse 54813* (CAS, MO); slopes at W edge of Teopisca, *D.E. Breedlove & J.L. Strother 46378* (CAS, MO). **Villa Corzo**: Above Colonia Vincente Guerrero on road to Finca Cuxtepec, *D.E. Breedlove & G. Davidse 54642* (CAS, MO), *D.E. Breedlove & G. Davidse 54631* (CAS, MO).

#### ﻿38. Muhlenbergia xanthodas

Taxon classificationPlantaePoalesPoaceae

Soderst., Contr. U.S. Natl. Herb. 34: 173. 1967.

5DEED452-F4BD-5AD4-B43A-967EDEBCC4EC

Fig. 2F–H

##### Type.

México, Chiapas, collected on rock on Mt. Ovando, 2300 m, 14–18 Nov 1939, *E. Matuda 4003* (holotype: US-1817864!; isotypes: F-64101, F-64108!, GH-00024052 [image!], NY-00381481 [image!], NY-00381482 [image!], US-2075810!).

##### Description.

Densely caespitose ***perennials*. *Culms*** 50–100 cm tall, glabrous. ***Leaf sheaths*** compressed-keeled, glabrous, minutely scabrillose near the collar; ***sheath auricles*** absent; ***ligules*** (3–) 6–13 mm long, delicate, hyaline, frayed with age, scarcely decurrent, becoming somewhat firm at base; ***blades*** 30–50(–70) cm long, 2–4 mm wide, conduplicate, becoming involute towards the apex, scabrous, apically long attenuate, the margins scabrous. ***Panicles*** 20–45(–55) cm long, 2–3(–4) cm wide, erect, golden yellow to yellowish-brown; ***primary branches*** 4–6 (–7) cm long, tightly appressed with spikelets to the base; ***pedicels*** shorter than the spikelets, thin, usually straight, scaberulous and often with a few hairs near the apex. ***Spikelets*** 2–3 mm long; ***glumes*** 2–2.5(–3) mm long, about equal in length, mostly smooth, somewhat lustrous and shining, translucent, unveined, apex acute; ***lemmas*** (2–)2.5–2.9 mm long, as long as the glumes or shorter, mostly glabrous or with a few appressed and very short trichomes at the base of the central vein, the awns (5–)6–20 mm long, golden; ***callus*** short-pilose; ***paleas*** as long as the lemma or a little longer, glabrous; ***anthers*** 1.5–2 mm long. ***Caryopses*** not seen.

##### Distribution.

*Muhlenbergiaxanthodas* is known from Guatemala and Chiapas, México (Sanchez-Ken 2018).

##### Ecology.

This species is found on rocky limestone slopes in deciduous tropical forests; 1500–2300 m.

##### Comments.

Morphologically, *M.xanthodas* is similar to *M.aurea* but differs in having longer spikelets 2–3 mm long (1.7–2.2 mm long in *M.aurea*) and veinless glumes (1-veined in *M.aurea*) with the upper mucronate.

##### Specimens examined.

Guatemala. **Huehuetenango.** Clearings and mixed forest in mountains near El Reposo, about 8 km from Mexican frontier, *L.O. Williams et al.41241* (MEXU); Canyon of Río Seligua, in “El Tapón” near Monos bridge, 40 km, NW of Huehuetenango, *L.O. Williams et al.41263* (MEXU). Mexico. **Chiapas. Altamirano**: 15 km Norte a colonia Puebla Nueva, *A. Pérez M. 221* (MEXU); **Ixtapa**: Intersection of the Tuxtla Gutiérrez-San Cristóbal de las Casas and the Villahermosa highways, *G. Davidse et al. 30101* (MEXU); **San Fernando**: Parque Nacional del Sumidero, 222 km NW of Tuxtla Gutiérrez, along the road to the canyon outlook, *G. Davidse et al. 29764* (MEXU); **Tenejapa**: Ojo del Río Yash zanal, *Alush Méndez Ton 5322* (MEXU); **Tuxtla Gutiérrez**: 18 km NE de Tuxtla Gutiérrez, carr al cañón del Sumidero, *A.J. Zenón*, *Ruíz y Valle # 2* (CIIDIR); Between Escuiplas and Cañada Honda, *Hernández X. & Sharp X-311* (US).

### ﻿Infrageneric classification of the species of *Muhlenbergia* in Central America

#### Muhlenbergia subg. Ramulosae P.M. Peterson, subgen. nov.

Taxon classificationPlantaePoalesPoaceae

59162961-3AB4-5107-AAEC-05E20FE69006

urn:lsid:ipni.org:names:77324812-1

##### Description.

Delicate ***annuals*. *Culms*** (3–)5–25 cm tall, erect or spreading, not rooting at the lower nodes. ***Leaf sheaths*** 3–30 cm long, usually shorter than the internodes; ***ligules*** 0.2–0.5 mm long, hyaline, apex truncate; ***blades*** 0.5–3.0 cm long, 0.8–1.2 mm wide, involute or flat. ***Panicles*** (1–)2–9 cm long, 0.6–2.7 mm wide, exserted, ovoid or deltoid, **branches** loosely contracted, ascending or spreading. ***Spikelets*** 1-flowered; ***glumes*** 0.4–0.7 mm long, shorter than the lemma, 1-veined, glabrous, apex obtuse to subacute; ***lemmas*** 0.8–1.3 mm long, oval, plump, 3-veined, awnless, mottled with greenish-black and greenish-white areas, apex acute. ***Stamens*** 3; ***anthers*** 0.2–0.3 mm long. ***Caryopses*** 0.5–1 mm long, ellipsoid.

### ﻿Species included (monotypic): *M.ramulosa* (Kunth) Swallen.

Muhlenbergiasubg.Bealia (Scribn.) P.M. Peterson: *M.ligularis*, *M.minutissima*.

Muhlenbergiasubg.Clomena (P. Beauv.) Hack.: *M.flabellata*, *M.montana*, *M.peruviana*, *M.quadridentata*.

*Muhlenbergia* subg. *Muhlenbergia: M.cenchroides*, *M.ciliata*, *M.diandra*, *M.diversiglumis*, *M.microsperma*, *M.pereilema*, *M.plumiseta*, *M.setarioides*, *M.spiciformis*, *M.tenella*, *M.uniseta*.

Muhlenbergiasubg.Pseudosporobolus (Parodi) P.M. Peterson: *M.implicata*, *M.phalaroides*, *M.repens*, *M.tenuissima*, *M.utilis*.

Muhlenbergiasubg.Ramulosae P.M. Peterson: *M.ramulosa*.

Muhlenbergiasubg.Trichochloa (P. Beauv.) A. Gray: *M.aurea*, *M.breviligula*, *M.capillaris*, *M.distichophylla*, *M.lehmanniana*, *M.macroura*, *M.mucronata*, *M.mutica*, *M.nigra*, *M.rígida*, *M.robusta*, *M.versicolor*, *M.xanthodas*.

Unplaced: *M.orophila*, *M.plumbea*.

### ﻿Excluded names

*Muhlenbergiabeyrichiana* is not known to occur in Chiapas or Central America and was reported in error (see comments under *M.diandra*) [Dávila et al. 2018], We believe *M.beyrichiana* occurs in Brazil, Ecuador, and Peru. Earlier, it was reported in México and Central America but these are in error (Espejo Serna et al. 2000; Lægaard and Peterson 2001; Peterson et al. 2001, 2018).

*Muhlenbergiaemersleyi* Vasey is not known to occur in Chiapas or Central America but was reported in Dávila et al. (2018) as occurring in Chiapas. We have not seen any specimens of this species from Central America or Chiapas.

*Muhlenbergiafragilis* Swallen is not known to occur in Chiapas or Central America but was reported in Dávila et al. (2018) and Sanchez-Ken (2018).

*Muhlenbergiagrandis* Vasey has been reported for Tabasco by Sanchez-Ken (2018) but we have not seen any specimens of this species from there, and since its distribution is primarily restricted to the Pacific slope of Sonora, Durango, Nayarit, Jalisco, Michoacán, and Queretero at elevations of 800–1600m, it seems unlikely to occur in Tabasco (Soderstrom 1967; Sanchez-Ken 2018). Additionally, *M.grandis* was not treated in Flora Mesoamericana by Reeder (1994).

*Muhlenbergiarepens* (J Presl) Hitchc. is not known to occur in Chiapas or Central America but was reported in Dávila et al. (2018) and Sanchez-Ken (2018).

*Muhlenbergiasinuosa* Swallen is not known to occur in Chiapas or Central America but was reported in Sanchez-Ken (2018).

*Muhlenbergiatarahumara* P.M. Peterson & Columbus is not known to occur in Chiapas or Central America but was reported in Dávila et al. (2018).

*Muhlenbergiatenuifolia* (Kunth) Kunth is not known to occur in Chiapas or Central America but was reported in Dávila et al. (2018) and Sanchez-Ken (2018).

## Supplementary Material

XML Treatment for Muhlenbergia

XML Treatment for Muhlenbergia aurea

XML Treatment for Muhlenbergia breviligula

XML Treatment for Muhlenbergia capillaris

XML Treatment for Muhlenbergia cenchroide

XML Treatment for Muhlenbergia ciliata

XML Treatment for Muhlenbergia diandra

XML Treatment for Muhlenbergia distichophylla

XML Treatment for Muhlenbergia diversiglumis

XML Treatment for Muhlenbergia flabellata

XML Treatment for Muhlenbergia implicata

XML Treatment for Muhlenbergia lehmanniana

XML Treatment for Muhlenbergia ligularis

XML Treatment for Muhlenbergia macroura

XML Treatment for Muhlenbergia microsperma

XML Treatment for Muhlenbergia minutissima

XML Treatment for Muhlenbergia montana

XML Treatment for Muhlenbergia mucronata

XML Treatment for Muhlenbergia mutica

XML Treatment for Muhlenbergia nigra

XML Treatment for Muhlenbergia orophila

XML Treatment for Muhlenbergia pereilema

XML Treatment for Muhlenbergia peruviana

XML Treatment for Muhlenbergia phalaroides

XML Treatment for Muhlenbergia plumbea

XML Treatment for Muhlenbergia plumiseta

XML Treatment for Muhlenbergia quadridentata

XML Treatment for Muhlenbergia ramulosa

XML Treatment for Muhlenbergia rigida

XML Treatment for Muhlenbergia robusta

XML Treatment for Muhlenbergia setarioides

XML Treatment for Muhlenbergia spiciformis

XML Treatment for Muhlenbergia tenella

XML Treatment for Muhlenbergia tenuissima

XML Treatment for Muhlenbergia uniseta

XML Treatment for Muhlenbergia utilis

XML Treatment for Muhlenbergia vaginata

XML Treatment for Muhlenbergia versicolor

XML Treatment for Muhlenbergia xanthodas

XML Treatment for Muhlenbergia subg. Ramulosae P.M. Peterson, subgen. nov.

## ﻿Additional information

### Author ORCIDs

Paul M. Peterson https://orcid.org/0000-0001-9405-5528

Yolanda Herrera Arrieta https://orcid.org/0000-0003-3814-6518

Silvia Lobo Cabezas https://orcid.org/0009-0007-8100-2559

Konstantin Romaschenko https://orcid.org/0000-0002-7248-4193

### Data availability

All of the data that support the findings of this study are available in the main text.
